# CapsNet-MHC predicts peptide-MHC class I binding based on capsule neural networks

**DOI:** 10.1038/s42003-023-04867-2

**Published:** 2023-05-05

**Authors:** Mahmood Kalemati, Saeid Darvishi, Somayyeh Koohi

**Affiliations:** Department of Computer Engineering, Sharif University of Technology, Tehran, Iran

**Keywords:** Machine learning, Bioinformatics

## Abstract

The Major Histocompatibility Complex (MHC) binds to the derived peptides from pathogens to present them to killer T cells on the cell surface. Developing computational methods for accurate, fast, and explainable peptide-MHC binding prediction can facilitate immunotherapies and vaccine development. Various deep learning-based methods rely on separate feature extraction from the peptide and MHC sequences and ignore their pairwise binding information. This paper develops a capsule neural network-based method to efficiently capture the peptide-MHC complex features to predict the peptide-MHC class I binding. Various evaluations confirmed our method outperformance over the alternative methods, while it can provide accurate prediction over less available data. Moreover, for providing precise insights into the results, we explored the essential features that contributed to the prediction. Since the simulation results demonstrated consistency with the experimental studies, we concluded that our method can be utilized for the accurate, rapid, and interpretable peptide-MHC binding prediction to assist biological therapies.

## Abstract (web summary)

A capsule neural network-based method captures pairwise information to accurately predict MHC-I and peptide binding. The comprehensive test of the model indicated both its superiority in performance and interpretability.

## Introduction

Peptide binding to the major histocompatibility complex (MHC) is crucial for presenting them to the adaptive immune system at the cell surface^1, 2^. Thanks to the recognition of the peptide-MHC complex by T cells, their interaction through a search process triggers T cells for initiating an effective cellular immune response ^3^. Hence, an accurate binding prediction of peptides to the MHC is essential for selective epitope identification as a pivotal task in immunotherapy and vaccine development.

There are two main classes of peptide–MHC complexes, each of which is presented on and recognized by different cell types of the immune system. Specifically, peptide-MHC class I complexes are presented on nucleated cells and are recognized by cytotoxic CD8 + T cells, while peptide-MHC class II are displayed on antigen-presenting cells, and make activate CD4 + T cells^3^. Furthermore, for MHC class I, as the main target in this study, locating the open binding groove close to both ends restricts the size of the bounded peptides to almost 8–10 residues, whereas MHC class II incorporates peptides of length 13–25 residues^4–6^. Moreover, the human MHC system, known as the human leukocyte antigen (HLA), includes the high polymorphic gene cluster in the whole genome, affecting the accommodation of residues in the binding groove, and hence, their binding specificity ^7^. Hence, the binding prediction between peptides and their partners attracts intensive research for developing computational methods and tools^8–13^.

According to the prediction algorithm, there are two main classes of computational MHC-peptide binding prediction methods, scoring-based and learning-based methods. The first one utilizes multiple statistical scoring functions to calculate the binding probability scores from the sequence input data. For example, Anthem^8^ adopts five scoring functions and a wrapper feature selection method to choose the best combination set of scoring functions. It should be noted that the existing score-based methods necessitate human intervention for feature selection, while their performance is limited due to their relatively simple sequence scoring functions. Therefore, they cannot efficiently capture the non-linear relations and complicated patterns from the data sequences^13^.

With the recent rapid accumulation of immunopeptidome data, the learning-based methods have shown high performance in MHC-peptide binding prediction from the raw sequence data. As a learning-based method, ACME^9^ proposes a convolutional neural networks (CNNs) architecture for learning the representation of input sequences. For features extraction and motifs capturing, an extra CNN block and an attention module have been added to the initial CNN block, respectively. Besides its simple concatenation model for feeding the final predictor network, this method necessitates manual reconstruction of the attention feature map ^11^. To overcome the latter drawback, DeepAttentionPan^11^ proposes a CNN-based method and three different attention blocks to extract the local and positional information from the input sequences. Despite extracting comprehensive features at the cost of increased complexity, the method cannot efficiently capture the interaction information from the concatenated peptide and MHC feature tensors.

To capture information from the peptide-MHC complex, a recent method, called TranspHLA^12^, takes advantage of the self-attention mechanism. In spite of considering positional and global information by applying a Transformer-based^14^ model, the method cannot extract meaningful local information efficiently, which can be captured with CNNs. On the other hand, HLAB^15^ employs ProtBert Transformer^16^ followed by Bi-LSTM to extract the contextual information from the input sequences. Despite capturing the long-term dependencies, this method relies on employing feature selection algorithms for refining extracted features and applying multiple machine learning models for the final prediction task.

Although the learning-based methods extract various information from the MHC and peptide sequences, they rely on a simple feature fusion model to capture the peptide-MHC complex binding context features^11–13^. Hence, they cannot consider the relationship between peptide and MHC sequences efficiently. Supplementary Note 1 and Supplementary Table S1 provide more details on the existing scoring-based and learning-based methods.

To address mentioned challenges, and especially, to overcome the drawbacks of the simple feature fusion models, we propose applying Capsule Neural networks^17^ (CapsNet) to capture ordered relations from the extracted learned features. Indeed, due to the separate consideration of peptide and MHC sequences in recent studies, simple concatenation could not capture the meaningful relationship between the merged latent vectors. On the other hand, it should be noted that the binding sites of interaction between the peptide and MHC structures are locally clustered, close to the binding pockets of MHC and the peptide anchors^18–20^. Hence, for efficient modeling of peptide-MHC binding, the interaction features should be considered along with the independent representation features from the peptide and MHC sequences.

CapsNet has been introduced to mimic biological neural systems for accurate modeling of hierarchical relationships and providing a stable data representation^17^. It applies special structures constructed by a group of neurons named capsules that accept vectors instead of scalars in conventional neural networks and route their outputs to the higher-order capsules by a mechanism named dynamic routing. In this manner, complicated hidden relationships and dependencies between peptide and MHC representations can be revealed and modeled efficiently. It should be noted that although CapsNet has been employed in the fields of computational biology^21–23^, to the best of our knowledge, its utilization for the peptide-MHC binding prediction has not been evaluated yet.

In this paper, we propose a capsule neural network-based method, named CapsNet-MHC, for predicting the MHC class I-peptide binding. CapsNet-MHC, as a pan-specific method, can accurately predict the binding between MHC allelic variants and peptides with rare sequence lengths. As its main idea, CapsNet-MHC applies capsule neural networks and dynamic routing to efficiently capture the hierarchical relations from the concatenated latent patterns of MHCs and peptides. CapsNet-MHC takes advantage of four major parts including an input encoder, based on the Blosum matrix, for considering frequencies of amino acids and their substitution probabilities, two CNN-Attention blocks for feature extraction, a Capsule neural network for effective feature extraction and fusion of the latent representation of MHC and peptide sequences, and finally, a fully-connected block for predicting binding values. In all, the main contributions of CapsNet-MHC can be summarized as follows:

- Considering hierarchical peptide-MHC complex patterns to avoid information loss in the succeeding integration step
- Proving interpretable results
- Proposing a pooling-free network architecture for capturing binding context information

To evaluate the performance of CapsNet-MHC for MHC-peptide prediction, various datasets have been considered. The comparison results on IEDB’s benchmark datasets confirmed our method outperformance over 9 alternative methods, in terms of the average area under the curve. Moreover, CapsNet-MHC provides better prediction performance than the 9 methods for 23 out of 61 IEDB allele datasets. Considering Anthem datasets, it also provides better performance against state-of-the-art methods in terms of average area under curve (AUC), with a value of 0.98. In all, the extensive tests and comparisons on well-known datasets confirm that CapsNet-MHC can be suitable for binding predicting peptide-MHC class I.

## Results

### Comparative studies

To verify the utility of CapsNet-MHC for predicting the peptide binding to MHC class I, we compared it with some popular and state-of-the-art methods, including PickPocket^10^, NetMHCcons^24^, NetMHCpan 4.0^25^, SMM^26^, NetMHC 4.0^27^, ARB ^28^, SMMPMBEC^28^, IEDB Consensus^28^, and DeepAttentionPan. Furthermore, we compared the prediction performance of CapsNet-MHC against recently developed methods, including ACME, Anthem, TranspHLA, and HLAB.

### Comparing CapsNet-MHC to baselines using IEDB’s datasets

**Fig. 1:**
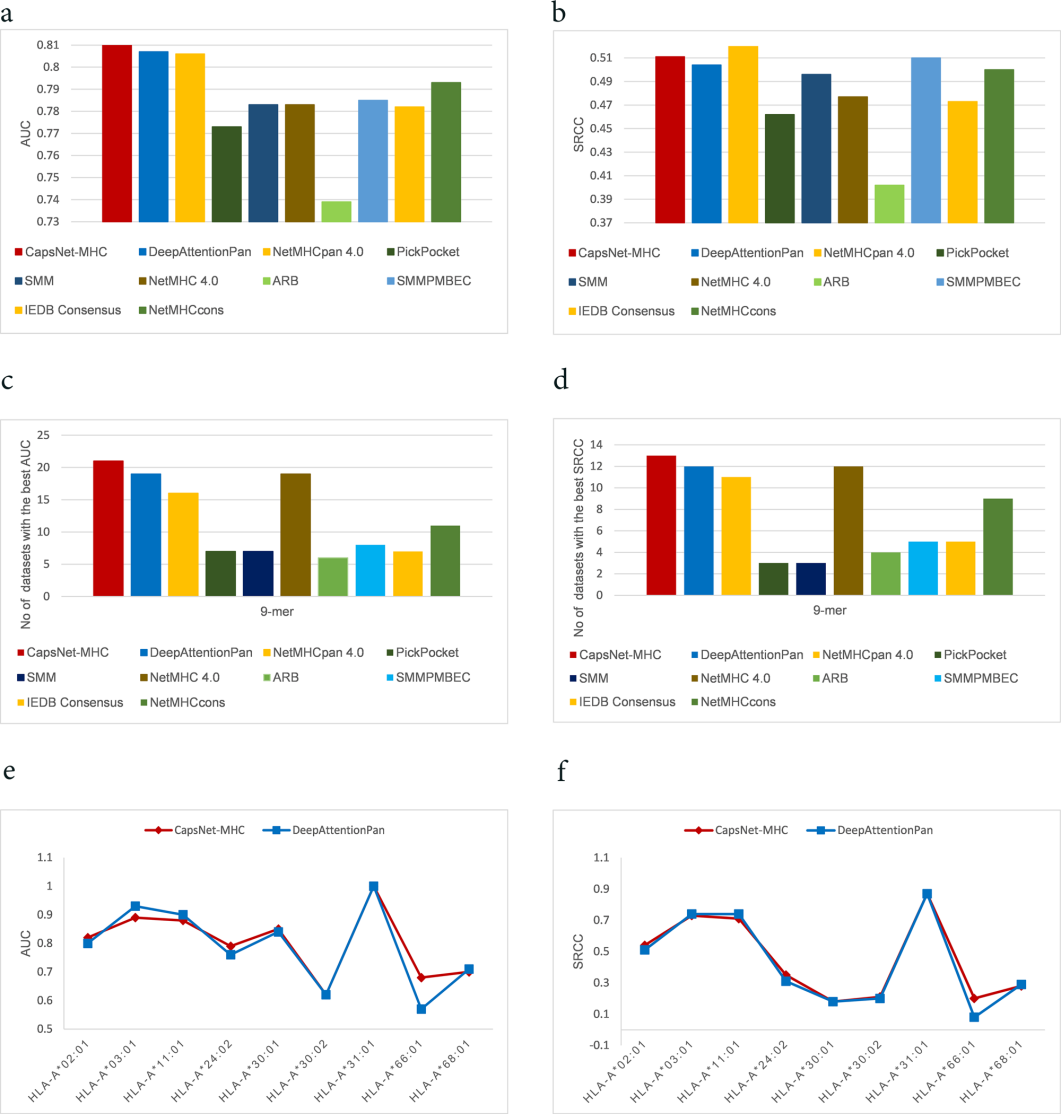
Comparing CapsNet-MHC with baselines over IEDB’s datasets. The average (**a**) AUC and (**b**) SRCC performance metric. The number of benchmark test datasets for which our method provides higher (**c**) AUC and (**d**) SRCC values for the 9-mer peptide length. **e** Prediction accuracy (AUC), and **f** SRCC against DeepAttentionPan for HLA-A alleles.

We evaluated the performance of CapsNet-MHC against baseline methods using IEDB’s datasets, as introduced in Section Methods. The average AUC and SRCC over the 61 test datasets are provided in Fig. 1. According to Fig. 1a, CapsNet-MHC outperforms all baseline methods in terms of AUC, as the main performance metric. Moreover, according to Fig. 1b, the SRCC value for our method is comparable to those of NetMHCPan4.0, while its AUC is slightly larger. Furthermore, CapsNet-MHC outperformed baseline methods in terms of the number of benchmark test datasets for which our method provides higher prediction accuracy. According to Fig. 1c, d, CapsNet-MHC provides better prediction performance, compared to the 9 alternative methods, for 21 out of 61 IEDB allele datasets for the 9-mer peptide length. The detailed performance comparison over the 61 IEDB’s benchmark datasets for all peptide lengths is provided in Supplementary Note 2 and Supplementary Tables S2, S3, and S4. For a comprehensive evaluation of CapsNet-MHC, we compared its prediction accuracy against DeepAttentionPan. According to Fig. 1e, f, CapsNet-MHC outperforms DeepAttentionPan for four alleles, and delivers an equal performance in terms of AUC and SRCC for two alleles. Summarizing the above discussion, we conclude that our proposed method provides better prediction accuracy for both performance metrics.

### Comparing CapsNet-MHC to state-of-the-art methods using Anthem’s datasets

**Fig. 2:**
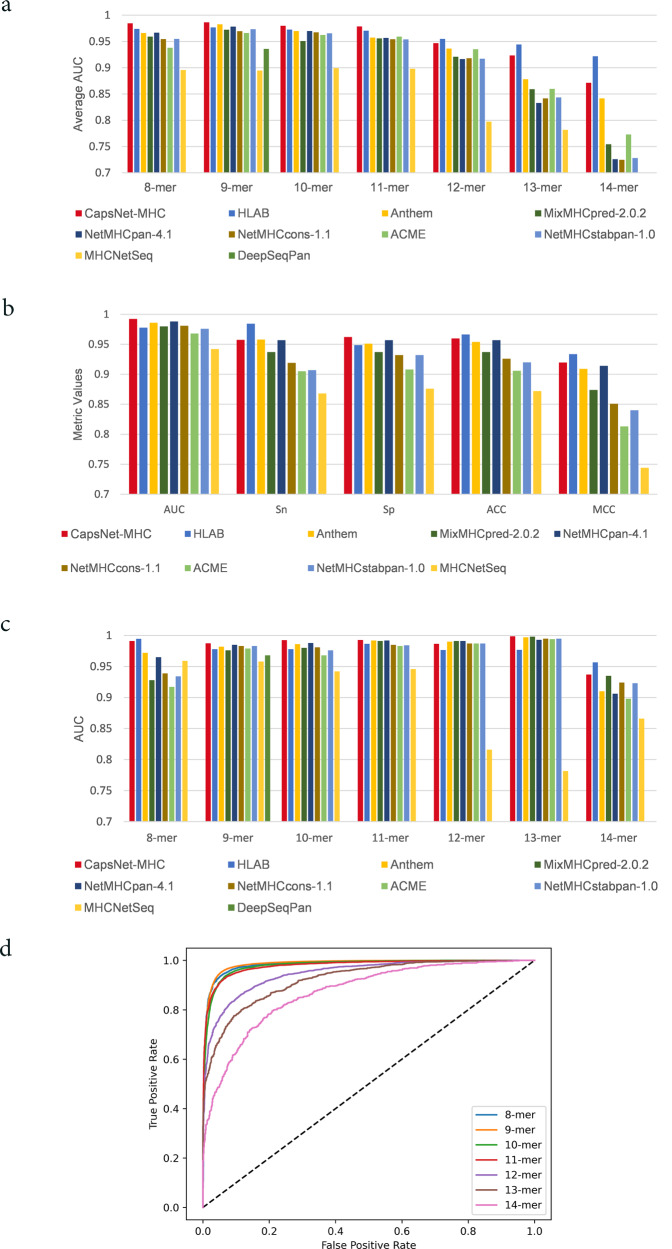
Comparing CapsNet-MHC with state-of-the-art methods over Anthem’s datasets. (**a**) AUC metric for all the HLA I alleles, (**b**) AUC, ACC, Sn, Sp, MCC for predicting the k-mer peptides binding to a specific (HLA-A*01:01) allele, (**c**) AUC values for predicting the k-mer peptides binding to a specific (HLA-A*01:01) allele, (**d**) ROC curves for all k-mer.

We evaluated our method and compared it against state-of-the-art methods for peptide binding prediction using Anthem’s datasets, as introduced in Section Methods. For this purpose, we compared CapsNet-MHC with four recently published methods, including ACME, Anthem, TranspHLA, and HLAB, along with six well-known methods, including MixMHCpred-2.0.2^29^, NetMHCpan-4.1 ^30^, NetMHCcons-1.1, NetMHCstabpan-1.0^31^, MHCNetSeq^32^, and DeepSeqPan^33^. Figure 2a represents the performance evaluation of CapsNet-MHC over the independent test sets of all the HLA-I alleles for various sizes of k-mer (i.e., 8 ≤ *k* ≤ 14), using the AUC metric. According to Fig. 2a, CapsNet-MHC outperforms all alternative methods for 4 out of 7 sizes of k-mer (i.e., 8–11), and achieves the second rank for k-mer of size 12 to 14. For more clarification of superiority of CapsNet-MHC, the full distribution of AUC values for HLAB and CapsNet-MHC for various sizes of peptide k-mer (i.e., 8 ≤ *k* ≤ 14) is represented in Supplementary Fig. S1. Overall, our method outperformed all state-of-the-art methods for all the HLA I alleles for various peptide lengths. The detailed data for the CapsNet-MHC performance comparison with the alternative studies on Anthem’s independent test sets of all HLA-I alleles for various sizes of k-mer is provided in Supplementary Note 3 and Supplementary Table S5. Furthermore, the full distributions of ACC, MCC, Specificity, and Sensitivity values for CapsNet-MHC are illustrated in Supplementary Fig. S2.

For allele-specific evaluation, we provided the comparison of CapsNet-MHC against state-of-the-art methods for a specific allotype HLA-A*01:01 using five performance metrics, AUC, ACC, MCC, Sn, and Sp. As shown in Fig. 2b, CapsNet-MHC provides prediction outperformance over the alternative methods for HLA-A*01:01 and 10-mer peptides. Moreover, according to Fig. 2c, our method outperforms the existing methods for HLA-A*01:01 and 4 out of 7 sizes of k-mer (i.e., 9, 10, 11, and 13) peptides. Hence, we can conclude that besides its superiority as a pan-specific method (as shown in Fig. 2a), CapsNet-MHC can be utilized as an allele-specific method for peptide binding prediction to MHC Class I. For a more detailed evaluation of CapsNet-MHC applicability as a binary classification task, we provided the receiver operating characteristic (ROC) curves for binding prediction of peptides to the MHC. According to Fig. 2d, the area under ROC curves for k-mer sizes of 8 to 11 are close to 0.98. Hence, we can conclude that our proposed method can be utilized for all peptide lengths and MHC Class I alleles.

We compared CapsNet-MHC against a recently developed method, called TranspHLA, which performs training and evaluation over an almost doubled data volume, compared to the HLAB, and CapsNet-MHC, while the MHC’s C alleles are excluded. Indeed, for delivering acceptable accuracy prediction, the transformation-based methods rely on training over massive data points. Based on the reported results^12^, Supplementary Fig. S3a compares the AUC values of CapsNet-MHC against those of TranspHLA, while HLAB’s results are included for more clear comparisons. According to this figure, CapsNet-MHC outperformed both state-of-the-art methods, TranspHLA and HLAB. For a fair evaluation, we compared CapsNet-MHC with TranspHLA for both independent and external test sets, addressed by TranspHLA. Supplementary Figure S3b, c represents the average values of two performance metrics, AUC and ACC, over the independent and external datasets, respectively. According to these figures, our method provides comparable prediction accuracies for both test sets in terms of the AUC and ACC metrics, respectively.

### Interpretability studies and biological insights

**Fig. 3:**
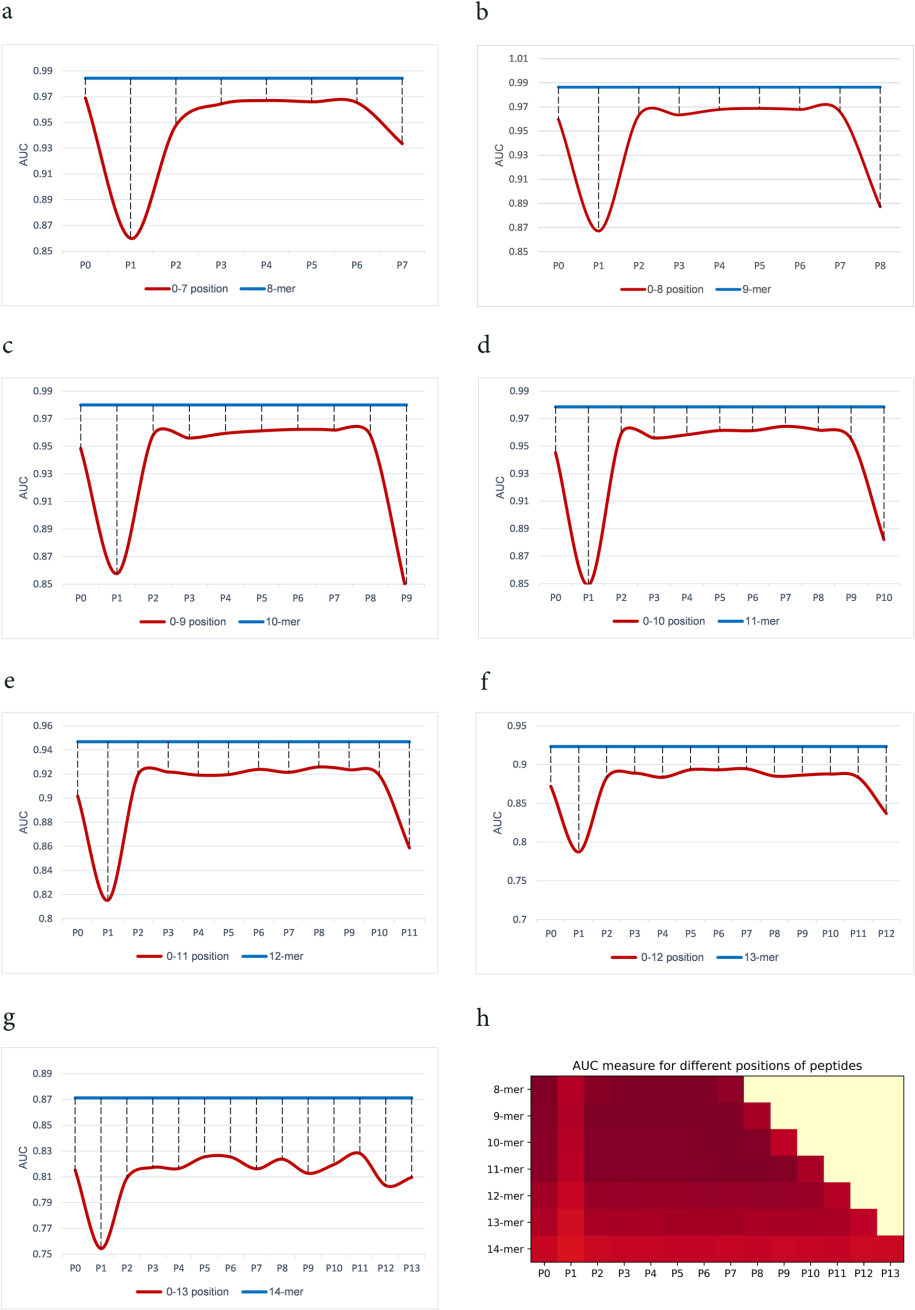
Permutation feature importance analysis. **a**–**g** Permutation feature importance scores in terms of the distance values between AUC for the shuffled and unchanged values for various positions of peptide k-mer (i.e., 8 ≤ *k* ≤ 14)). **h** Permutation feature importance heatmap for all sizes of peptide k-mer.

Model’s interpretability helps human experts to understand and interpret the method and the provided results for realistic scenarios. To represent the interpretability of CapsNet-MHC, we take advantage of the permutation feature importance algorithm^34, 35^ which represents more important and useful features for prediction tasks over Anthem’s benchmark datasets. Based on this algorithm, we followed three steps. First, we shuffled the values for each position of the peptide sequences data, while the values for other positions are unchanged. Then, the peptide binding prediction is performed by the proposed method for the shuffled data. Finally, we calculated the feature importance scores using the distance values between the prediction scores for the shuffled and unshuffled data. Figure 3a–g represents the feature importance scores in terms of the distance values between AUC for the shuffled and unchanged values for various positions of peptide k-mer (i.e., 8 ≤ *k* ≤ 14), respectively. Moreover, Fig. 3h provides a heatmap for permutation importance feature scores for all sizes of k-mer. According to Fig. 3, position 1 (P1), as well as the last available position for each k-mer represent the most important and useful features for peptide binding prediction. These results are consistent with the contribution of anchor positions for peptide binding to MHC Class I.

**Fig. 4:**
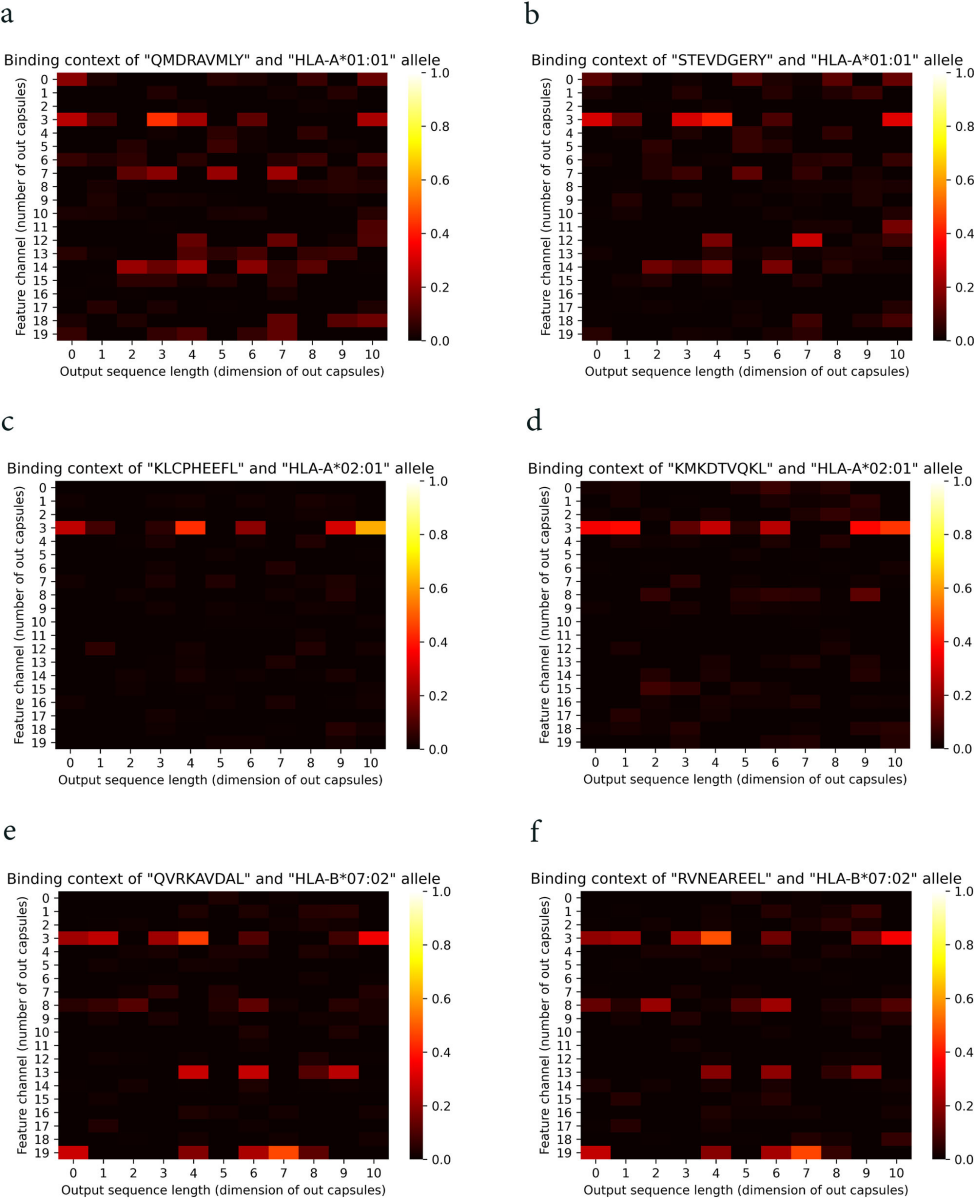
Contribution of capsules for learning the binding features. **a**, **b** Heatmaps for the HLA-A*01:01 allele and the peptides QMDRAVMLY, and STEVDGERY, respectively. **c**, **d** Heatmaps for the HLA-A*02:01 allele and the peptides KLCPHEEFL, and KMKDTVQKL, respectively. **e**, **f** Heatmaps for the HLA-B*07:02 allele and the peptides QVRKAVDAL, and RVNEAREEL, respectively.

As follows, we explore the capability of CapsNet-MHC to capture the binding features using dynamic routing, and extract important binding regions from the peptide-MHC complex. For this purpose, we visualized the outputs from capsules for peptide-HLA complexes, including HLA-A*01:01, HLA-A*02:01, and HLA-B*07:02 alleles and various peptide pairs. From the heatmaps, as shown in Fig. 4a–f, the overall learned pattern for each peptide-HLA complex for each individual HLA allele (the total 2D heatmaps) are almost identical, while the partial patterns for each capsule (i.e., feature vectors in each row) are slightly different. For example, according to Fig. 4a, b, the captured binding patterns for different peptides (i.e., “QMDRAVMLY” and “STEVDGERY”) are slightly different for an individual HLA allele (i.e., HLA-A*01:01). Therefore, we can suggest the network to learn the patterns hierarchically and dominantly based on the HLA alleles. This conclusion is consistent with the capability of capsules to capture the hierarchical features using dynamic routing and its equivariance and invariance properties.

Furthermore, according to Fig. 4a–f, we can conclude that for learning the peptide-MHC binding features, the contributions of one or more capsules are more than others for each individual HLA. For example, capsules 3, 7, 12, and 14, listed on the vertical axis, contain feature vectors with greater values close to 1.0 (bright colors) compared to the other capsules. Therefore, it can be concluded that some parts of the peptide-HLA complex have more contribution to the binding prediction. This finding is consistent with the contribution of specific sites of HLA and peptides, known as binding domains, to the peptide binding to MHC.

Moreover, for more clarification of CapsNet-MHC interpretability, we investigated the capsule’s output for peptide-HLA complexes consisting of individual peptides and different HLA alleles. For this purpose, we visualized the capsules’ outputs for peptide-HLA complexes, including the peptide “APRNKIGTL” and two HLA alleles HLA-A*01:01 and HLA-B*07:02. From the heatmaps, as shown in Supplementary Fig. S4a, b, the overall learned pattern for each peptide-HLA complex (i.e., total 2D heatmaps) is almost identical to that of each individual peptide, while the partial patterns for each capsule (i.e., feature vectors in each row) are slightly different from those of individual peptide. The aforementioned conclusions clarify capsules contribution to the learning of binding patterns, and provide useful insights into the interpretability of CapsNet-MHC.

**Fig. 5:**
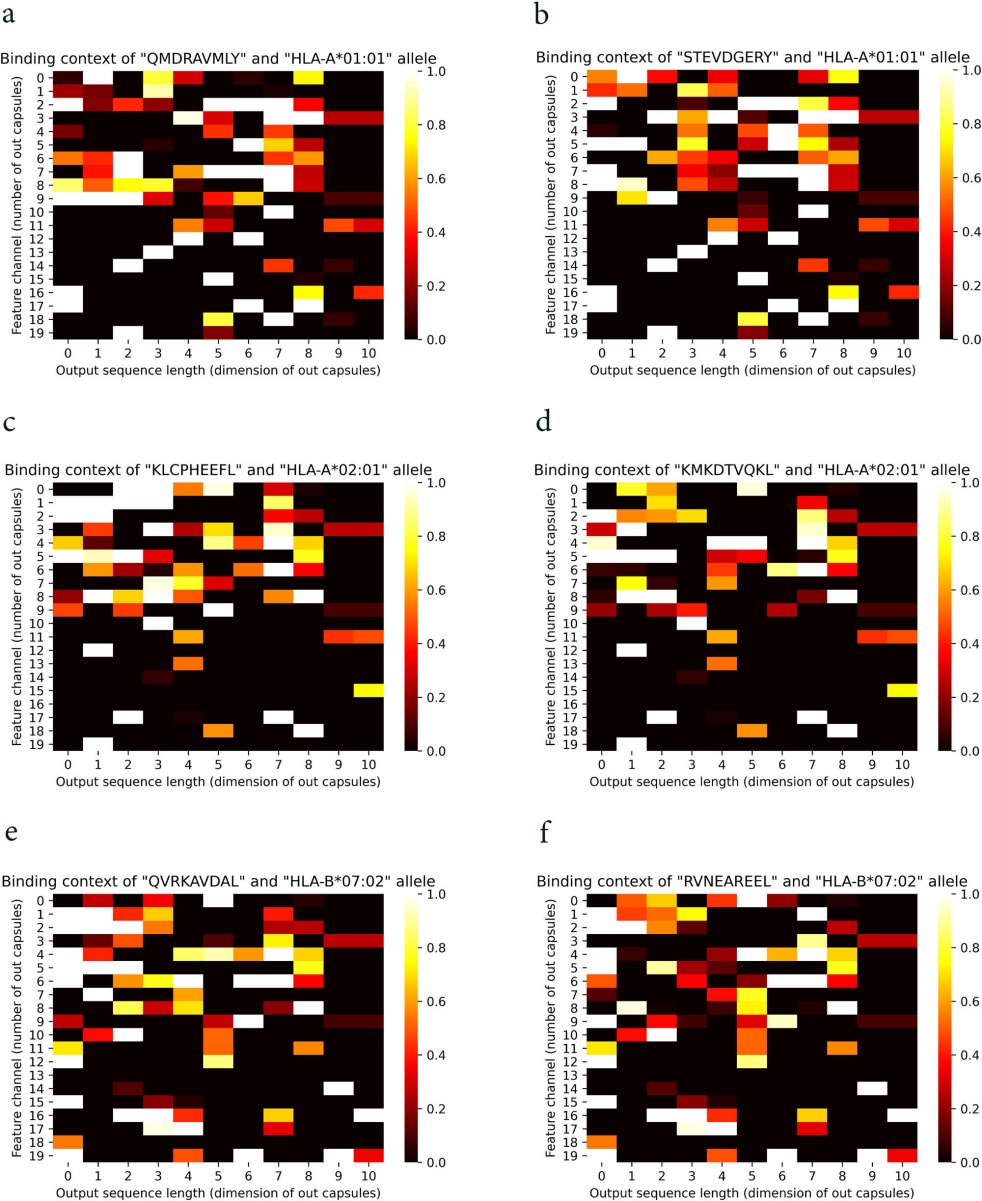
Contribution of a simple concatenation layer for learning the binding features. **a**, **b** Heatmaps for the HLA-A*01:01 allele and the peptides QMDRAVMLY and STEVDGERY, respectively. **c**, **d** Heatmaps for the HLA-A*02:01 allele and the peptides KLCPHEEFL and KMKDTVQKL, respectively. **e**, **f** Heatmaps for the HLA-B*07:02 allele and the peptides QVRKAVDAL and RVNEAREEL, respectively.

**Fig. 6:**
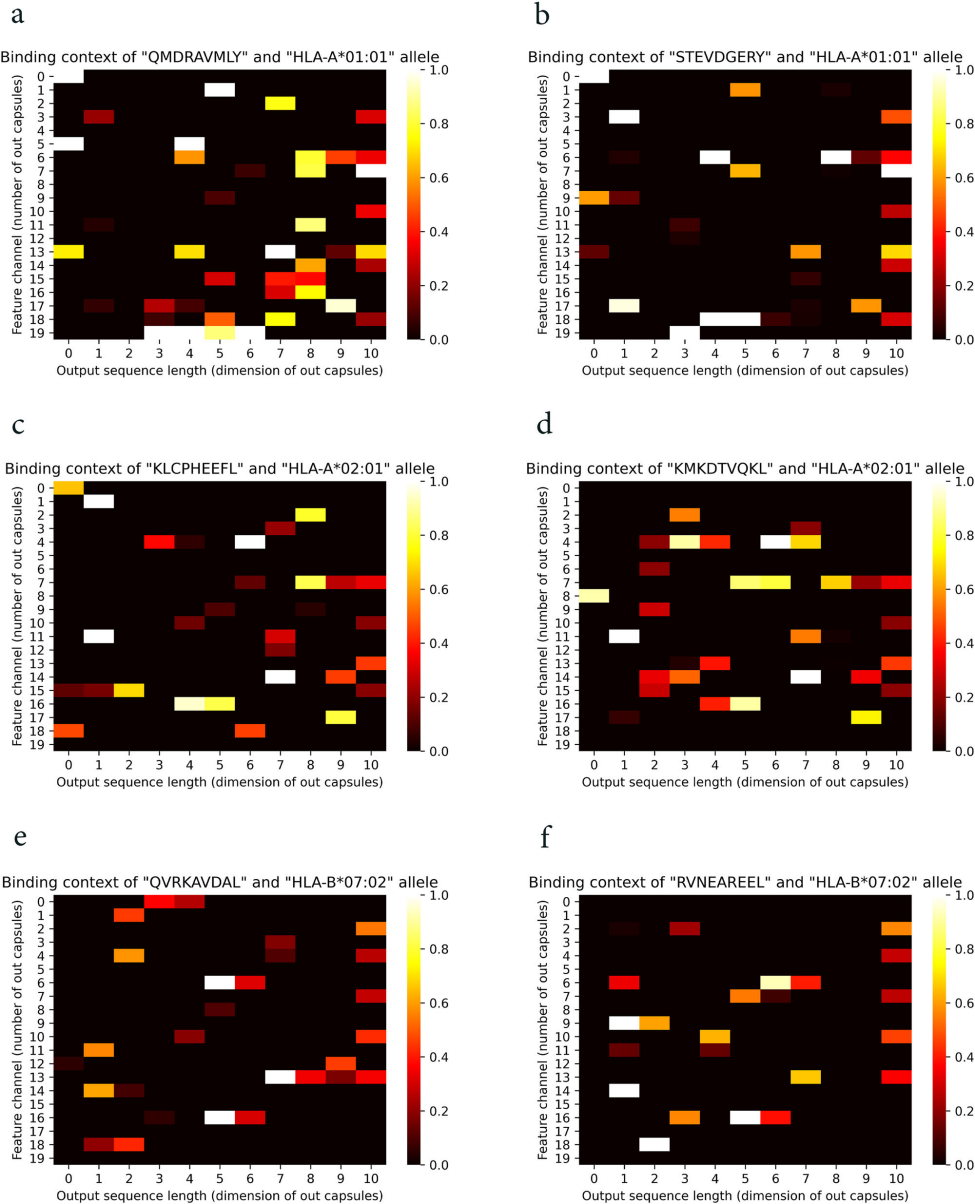
Contribution of CNNs for learning the binding features. **a**, **b** Heatmaps for the HLA-A*01:01 allele and the peptides QMDRAVMLY and STEVDGERY, respectively. **c**, **d** Heatmaps for the HLA-A*02:01 allele and the peptides KLCPHEEFL and KMKDTVQKL, respectively. **e**, **f** Heatmaps for the HLA-B*07:02 allele and the peptides QVRKAVDAL and RVNEAREEL, respectively.

Furthermore, to show why and how the fusion works using CapsNet in the binding feature extraction step of the model, we provided comparisons between CapsNet-based networks against a simple concatenation layer and CNN-based binding feature extractors. To this end, we utilized a conventional concatenation layer in CapsNet-MHC architecture and considered DeepAttentionPan architecture which utilized CNNs in the binding feature extractor step. The heatmap for learned features for the simple concatenation layer and CNN layer for specific alleles and peptides are shown in Figs. 5 and 6. It should be noted that, the experiments for the simple concatenation layer and CNNs conducted over the same alleles and peptides mentioned for CapsNet in Fig. 4. According to Fig. 5a–f, as expected, the heatmap for the concatenation layer cannot provide meaningful partial and overall learned patterns for the paired peptide-HLA complex. In these figures, the overall patterns are almost identical presenting the simple merging of peptide and MHC representations. On the other hand, according to Fig. 6a–f, although the heatmap for CNN-based binding feature extractor can learn some partial patterns, it cannot provide overall patterns from the peptide-MHC molecules. For example, the neurons in the last dimension (i.e., dimension 10) have learned almost identical patterns which can indicate they have learned a specific allele.

From a biological perspective, CapsNet has the ability to learn partial and overall patterns, while the CNN-based binding feature extractor is capable of learning some partial patterns for peptide-MHC complex molecules. On the other hand, a simple concatenation layer architecture is unable to provide useful learned binding features from the peptide-MHC complex molecules for the prediction task. These findings align with CapsNet’s ability to provide partial and overall patterns using capsules and dynamic routing processes for feature fusion tasks.

### Ablation studies for evaluating the contribution of model components

For evaluating the contribution of CapsNet to the prediction performance, we conducted ablation studies, as follows.

**Fig. 7:**
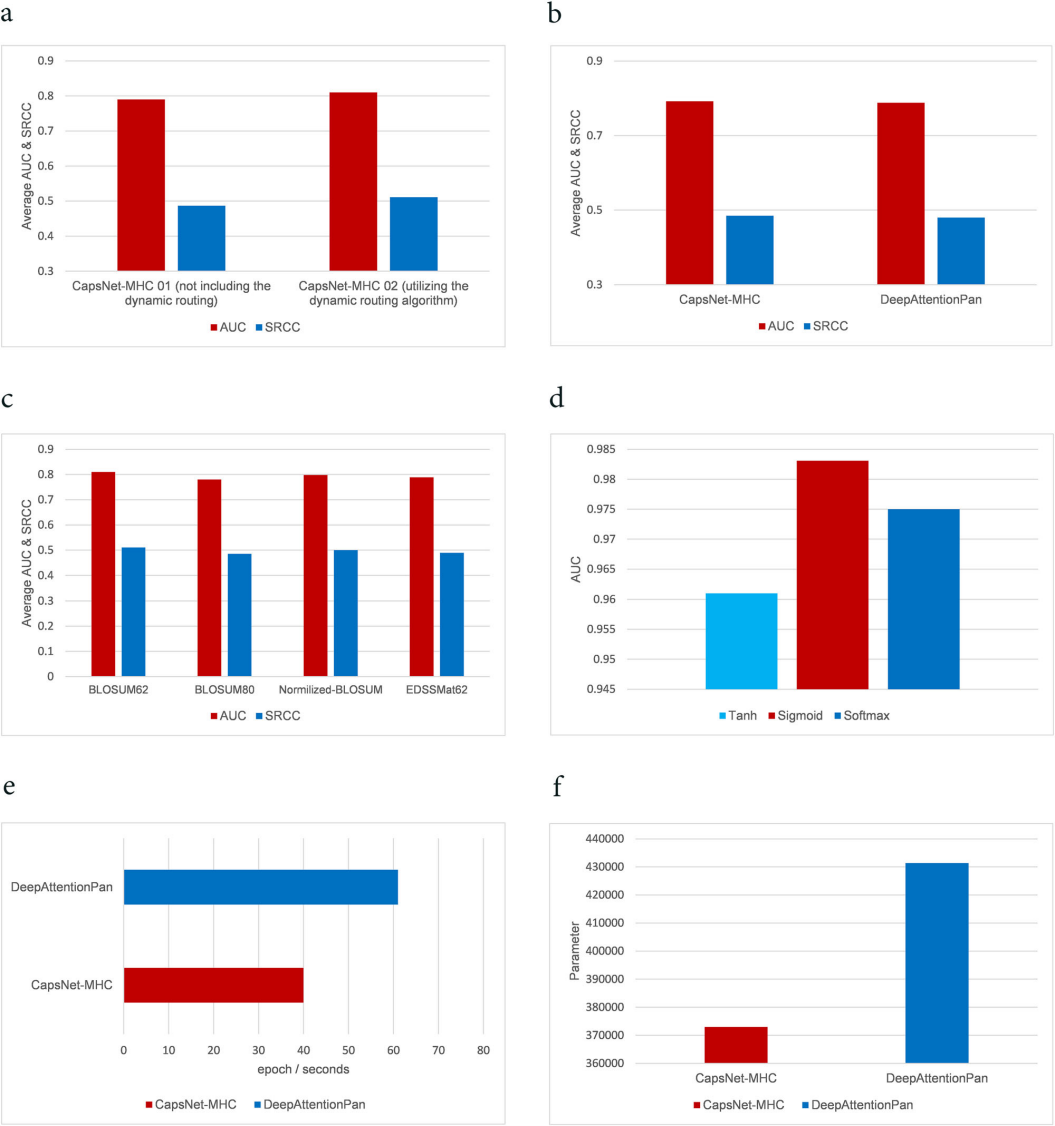
Ablation studies. **a** Comparison between CapsNet-MHC 01 (not including the dynamic routing) and CapsNet-MHC 02 (utilizing the dynamic routing algorithm), **b** Prediction performance comparison between CapsNet-MHC and DeepAttentionPan in the case of small data training set. **c** Prediction accuracy comparison of various encoding schemes. **d** Prediction accuracy comparison of various activation functions. Comparing CapsNet-MHC and DeepAttentionPan in terms of (**e**) run time and (**f**) the number of parameters.

For investigating the impact of dynamic routing process on the prediction performance, we considered two separate architectures, called CapsNet-MHC1.0, and CapsNet-MHC2.0. The applied blocks for both architectures are identical, except that the second one includes the dynamic routing algorithm. According to Fig. 7a, CapsNet-MHC2.0 outperformed CapsNet-MHC1.0 in terms of both AUC and SRCC metrics. Specifically, CapsNet-MHC2.0 improved prediction performance by 0.02 and 0.024 in terms of the AUC and SRCC, respectively, for IEDB’s benchmark datasets. Hence, we can conclude that dynamic routing, as a parallel attention mechanism, can help to recognize the overlapped patterns, as reported in CapsNet^17^.

For evaluating the efficiency of capsules in the case of small training datasets, we conducted an experiment with 60% of available data from the IEDB’s dataset. To this end, we re-trained and re-evaluated CapsNet-MHC. According to Fig. 7b, CapsNet-MHC outperformed a recently published method, DeepAttentionPan, in terms of both AUC and SRCC values. Therefore, we can conclude that CapsNet-MHC can be a practical method for training new predictive models for datasets with limited available data.

For a more detailed analysis of encoding choices, we trained and evaluated CapsNet-MHC with four widely-used encoding schemes for the input sequence, including Blosum62, Blosum80, normalized Blosum, and EDDSSMat62 ^36^. According to Fig. 7c, Blosum62 provides better AUC and SRCC values, compared to the alternative encoding schemes over benchmark test sets. The later achievement is due to the fact that Blosum62 delivers meaningful features of the biological relationship for the peptide and MHC sequences encoding.

For a classification task, the activation function maps the outputs to the range of [0, 1]. We considered three different configurations, each of which utilizes a different activation function, including Tanh, Sigmoid, and Softmax. According to the presented results in Fig. 7d, utilizing the Sigmoid activation function results in better prediction performance, as compared to the Softmax and Tanh.

To evaluate the impact of utilizing CapsNet, we compared the performance of CapsNet-MHC with that of DeepAttentionPan, in terms of prediction accuracy and network complexity. According to Fig. 1, our method outperformed DeepAttentionPan in terms of all accuracy metrics over the IEDB’s datasets. Furthermore, according to Fig. 7e and f, CapsNet-MHC reduced the runtime and the number of network parameters. Specifically, CapsNet-MHC delivered 30% smaller training and inferring time in terms of the seconds/epoch, and 13% smaller number of network parameters. Hence, CapsNet-MHC proposed a practical method for peptide binding prediction to the MHC class I in a fast and accurate fashion.

### Effect of utilizing transformer architectures and structural features

To provide an efficient encoding and a distributed representation for protein sequences, protein language models and transformer architectures have been widely utilized for various representation learning and prediction tasks. Recently, transformer architectures have been adopted in peptide-MHC binding prediction methods to perform accurate representation of MHC and peptide sequences for prediction task^12, 15^. To investigate the transformers adoption in the CapsNet-MHC, we employed two recently published transformers called ESM-1 and ESM-2. ESM-1 transformer is a deep contextual language model which can be employed for representation learning from the individual protein sequences for diverse protein tasks, such as homology detection, prediction of protein structure, residue–residue contacts, and mutational effect^37^. On the other hand, ESM-2 transformer is a general-purpose protein language model for predicting the structure, function, and other protein properties from the sequences^38^. While we utilized ESM-1 and ESM-2 in the encoder part of the model, the corresponding architecture for ESM-1 and ESM-2 named as CapsNet-MHC-ESM1 and CapsNet-MHC-ESM2, respectively.

**Fig. 8:**
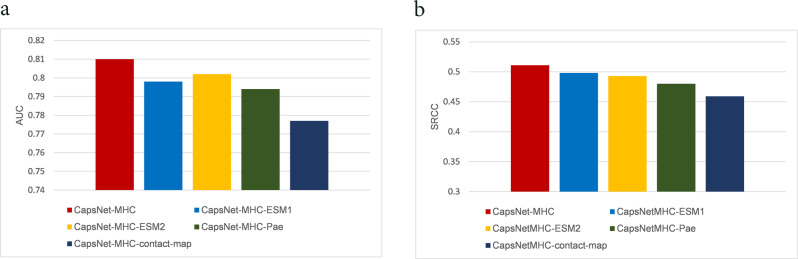
Comparing CapsNet-MHC with its four transformer-based versions over IEDB’s datasets. The average (**a**) AUC and (**b**) SRCC performance metrics.

Furthermore, we utilized structural features taking advantage of ESMFold to provide an end-to-end structure prediction directly from the sequence of a MHC and peptide^38^. ESMFold has been designed based on language models trained over a large database of protein sequences, and so, provides atomic resolution prediction of protein structure in the form of contact maps^38^. In this regard, we utilized contact maps for MHC and peptide sequences predicted by ESMFold in the encoder part of the model, and so, the corresponding architecture is named as CapsNet-MHC-contact-map. Moreover, we employed the structural-related features PAE (predicted aligned error), provided by ESMFold, as the fourth extra representation for MHC and peptide sequences for CapsNet-MHC. In this manner, the corresponding architecture is named as CapsNet-MHC-Pae. The average AUC and SRCC for CapsNet-MHC, and its four transformer-based versions including CapsNet-MHC-ESM1, CapsNet-MHC-ESM2, CapsNet-MHC-Pae, and CapsNet-MHC-contact-map over the 61 test datasets of IEDB are provided in Fig. 8. According to Fig. 8a, b, CapsNet-MHC outperforms all transformer-based versions in terms of AUC, and SRCC performance metrics. CapsNet-MHC-ESM2 and CapsNet-MHC-ESM1 provide the next better AUC and SRCC, respectively. The detailed performance comparison over the 61 IEDB’s benchmark datasets for all peptide lengths is provided in Supplementary Note 4 and Supplementary Table S6.

**Fig. 9:**
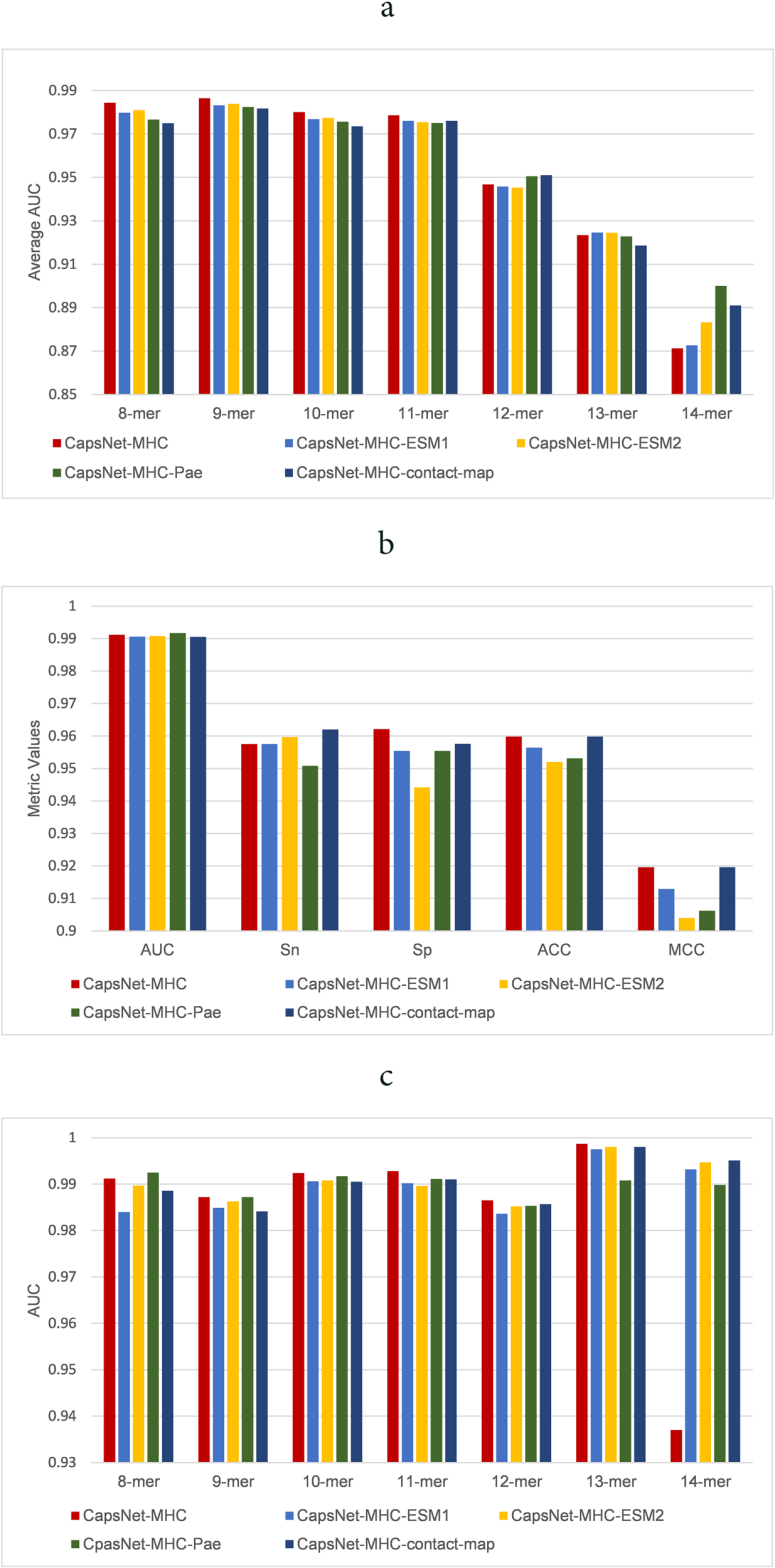
Comparing CapsNet-MHC with its four transformer-based versions over Anthem’s datasets. (**a**) AUC metric for all the HLA I alleles, (**b**) AUC, ACC, Sn, Sp, and MCC for predicting the k-mer peptides binding to a specific (HLA-A*01:01) allele, (**c**) AUC values for predicting the k-mer peptides binding to a specific (HLA-A*01:01) allele.

For more investigation, we compared CapsNet-MHC against its four transformer-based versions using Anthem’s datasets. Figure 9a represents the performance evaluation of all versions of CapsNet-MHC over the independent test sets of all the HLA-I alleles for various sizes of k-mer (i.e., 8 ≤ *k* ≤ 14), using the AUC metric. According to Fig. 9a, CapsNet-MHC outperforms all alternative transformer-based versions for four out of seven sizes of k-mer (i.e., 8–11). Moreover, CapsNet-MHC-contact-map, CapsNet-MHC-ESM1, and CapsNet-MHC-Pae provide better AUC for 12-mer, 13-mer, and 14-mer peptides, respectively. Hence, we can conclude that utilizing structural and structural-related features extracted from the transformers can provide better performance for peptide-MHC binding prediction for the peptides with small data and longer lengths in the pan-specific setting. These results are consistent with the capability of transformer for providing efficient representations where trained over a large database of protein sequences. The detailed data for the CapsNet-MHC performance comparison with its four transformer-based versions using Anthem’s independent test sets of all HLA-I alleles for various sizes of k-mer is provided in Supplementary Note 5 and Supplementary Table S7.

For allele-specific evaluation, we provided the comparison of CapsNet-MHC against its four transformer-based versions using five performance metrics, AUC, ACC, MCC, Sn, and Sp. As shown in Fig. 9b, CapsNet-MHC-Pae and CapsNet-MHC-contact-map which utilized structural-related and structural features, respectively, provide prediction outperformance over other versions of CapsNet-MHC for HLA-A*01:01 in terms of AUC and Sn, respectively. Furthermore, CapsNet-MHC-contact-map provides equal performance with CapsNet-MHC in terms of ACC and MCC. Moreover, according to Fig. 9c, for the specific allotype HLA-A*01:01, CapsNet-MHC-Pae and CapsNet-MHC-contact-map outperform CapsNet-MHC for 8-mer and 14-mer peptides, respectively. In all, we can conclude that utilizing structural (i.e., contact map) features can provide better performance for peptide-MHC binding prediction in the allele-specific setting for rare and longer peptide sequence data which provide more structural features.

## Discussion

Providing a precise, explainable, and rapid method for the peptide binding prediction to the MHC is important for initiating a suitable immune response, facilitating immunotherapy, and speeding up vaccine development. In this paper, we have proposed a method for peptide-MHC class I binding prediction which provides fully-automated feature extraction from the raw sequences, unlike the scoring-based methods, and taking advantage of CapsNet, considers the interaction and binding features efficiently and explanatorily, unlike the alternative learning-based methods.

Specifically, we applied widely-used CNNs along with the attention mechanisms for feature extraction from the encoded peptide and MHC sequences, using Blosum62 input encoding method. To feed the learned features into the final prediction network, a Capsule neural network was adopted to efficiently capture the binding features from the learned features. The various evaluations over multiple datasets demonstrated the superiority of CapsNet-MHC, compared to the alternative tools for peptide-MHC binding prediction. Specifically, CapsNet-MHC outperformed all baselines and recently published state-of-the-art HLAB and TranspHLA over IEDB’s and Anthem’s datasets.

Considering runtime evaluation, it should be noted that the superior deep learning-based methods rely on utilizing complex neural networks to capture the complicated patterns, and so, the training time may not be affordable for realistic scenarios, especially in the case that re-training is required for new experimental datasets. For example, TranspHLA necessitated a high memory usage (i.e., 92 GB) for the transformer architecture to train and extract the informative feature from the peptide and MHC sequences in the form of multiple encoder blocks. On the other hand, as a simpler architecture, DeepAttentionPan applied CNNs for the binding context feature extraction, while it cannot efficiently extract the interaction relationship of the peptide-MHC complex features. As discussed in the previous section, CapsNet-MHC provides superior performance with a smaller training time, as well as a reduced number of network parameters. Furthermore, the proposed method, compared to the alternative ones, enables model training with the smaller available dataset. Specifically, as demonstrated by the simulation results, CapsNet-MHC can be applied for re-training over smaller datasets without considerable degradation of prediction accuracy. In all, our method can be utilized as a rapid tool for accurate peptide binding to the MHC class I.

Besides providing accuracy and speed improvements, it is worth noting that the resultant predictions of the proposed deep learning-based method are consistent with the experimental studies, and so, they are interpretable and explainable by domain experts. To provide more data insights, we applied a feature importance algorithm to explain highly effective biological information in the prediction task. Various conducted evaluations over the studied datasets illustrated the prediction consistency with the contribution of anchor positions for peptide binding to MHC Class I. As a result, besides the accurate prediction and fast training, CapsNet-MHC provides interpretable and explainable decisions to help the domain experts for a more precise analysis of the results.

Furthermore, we leveraged distributed representations and structural features by utilizing multiple transformer architectures, including ESM-1, ESM-2, and ESMFold. Our evaluations over benchmark datasets have demonstrated that the use of structural features and distributed representations extracted from transformers has enhanced the performance of peptide-MHC complex binding prediction, particularly for rare peptides and peptides with longer lengths. Therefore, incorporating these features can be beneficial in scenarios with limited available data, particularly for longer peptides.

In all, CapsNet-MHC outperforms various popular and state-of-the-art methods for predicting peptide binding to the MHC class I. Our proposed method provides superior prediction accuracy, compared to the state-of-the-art methods, while performing explainable results. Moreover, the training of CapsNet-MHC is performed rapidly, while it can also be trained over less available data without considerable degradation of the prediction accuracy, compared to the baseline methods.

## Methods

### Datasets

We evaluated the performance of CapsNet-MHC for the peptide binding prediction to MHC class I using two widely-used groups of datasets, including public IEDB datasets and the datasets retrieved from the study Anthem, as explained in more detail in the following.

### IEDB’s datasets

For evaluating CapsNet-MHC and comparing it with the alternative methods, using IEDB’s dataset^39^, the training datasets called BD2013 and the test datasets called IEDB’s weekly benchmark dataset are downloaded from http://tools.iedb.org/main/datasets/ and http://tools.iedb.org/auto_bench/mhci/weekly/, respectively. Moreover, the resultant performance metrics are compared against those of various well-known methods, including DeepAttentionPan, NetMHCpan 4.0, PickPocket, SMM, NetMHC 4.0, ARB, SMMPMBEC, IEDB Consensus, and NetMHCcons. More detailed explanations of IEDB’s datasets are provided in Supplementary Note 6 and Supplementary Table S8.

### Anthem’s datasets

For a more comprehensive evaluation of CapsNet-MHC, Anthem’s datasets are downloaded from https://github.com/17shutao/Anthem/tree/master/Dataset. Considering Anthem’s dataset, the achieved performance metrics are compared with those of alternative methods, including TranspHLA, Anthem, ACME, NetMHCPan-4.1, HLAB, MixMHCpred-2.0.2, NetMHCcons-1.1, NetMHCstabpan-1.0, DeepSeqPan, and MHCNetSeq. More detailed explanations of Anthem’s datasets are provided in Supplementary Note 6 and Supplementary Table S8.

### Implementation details

The method was implemented using the popular Python library PyTorch on NVIDIA’s GeForce GTX 1080 with 11 GB of available memory. For evaluating CapsNet-MHC, we applied five-fold cross-validation at the training step. To this end, the training set is split into five nearly equal parts, where four parts are used for the training, and the remained part is used for the model evaluation. To ensure that all parts of the datasets are included in evaluating the proposed model, the training and evaluation phases are repeated in five iterations. Finally, the model is evaluated using an independent test set to demonstrate its prediction capability for any unseen data. In this manner, the average prediction accuracy of various iterations is considered as the final result. Supplementary Note 7 and Supplementary Table S9 illustrate various parameter settings for CapsNet-MHC.

### Evaluation metrics

We evaluated the prediction performance of CapsNet-MHC using various widely-used evaluation metrics in prior studies for the peptide binding prediction to MHC class I. These metrics include Accuracy (ACC), Spearman’s rank correlation coefficient (SRCC), Matthews correlation coefficient (MCC), Sensitivity (Sn), Specificity (Sp), and F1 score, which are calculated by (Eqs. 1–6), respectively.

1 $${Accuracy}=\frac{{TP}+{TN}}{{TP}+{TN}+{FP}+{FN}},$$

where, TP, TN, FP, and FN are the numbers of true positives, true negatives, false positives, and false negatives, respectively.

2 $${SRCC}=1-\frac{6\sum {d}_{i}^{2}}{n\left({n}^{2}-1\right)},$$

where, *d*_*i*_ and n denote the difference between the two ranks of each observation and the number of observations, respectively.

3 $${MCC}=\frac{\left({TP}\times {TN}\right)-({FN}\times {FP})}{\sqrt{({TP}+{FN})\times ({TN}+{FP})\times ({TP}+{FP})\times ({TN}+{FN})}}$$

4 $${S}_{n}=\frac{{TP}}{{TP}+{FN}}$$

5 $${S}_{p}=\frac{{TN}}{{TN}+{FP}}$$

6 $$F1\,{score}=\frac{2\times {Precision}\times {Recall}}{{Percision}+{Recall}},$$

where Precision and Recall are calculated by Eqs. (7) and, 8, respectively.

7 $${Precision}=\frac{{TP}}{{TP}+{FP}}$$

8 $${Recall}=\frac{{TP}}{{TP}+{FN}}$$

In addition to all aforementioned metrics, we included the area under the ROC curve (AUC), as a performance evaluation metric.

### CapsNet-MHC method

**Fig. 10:**
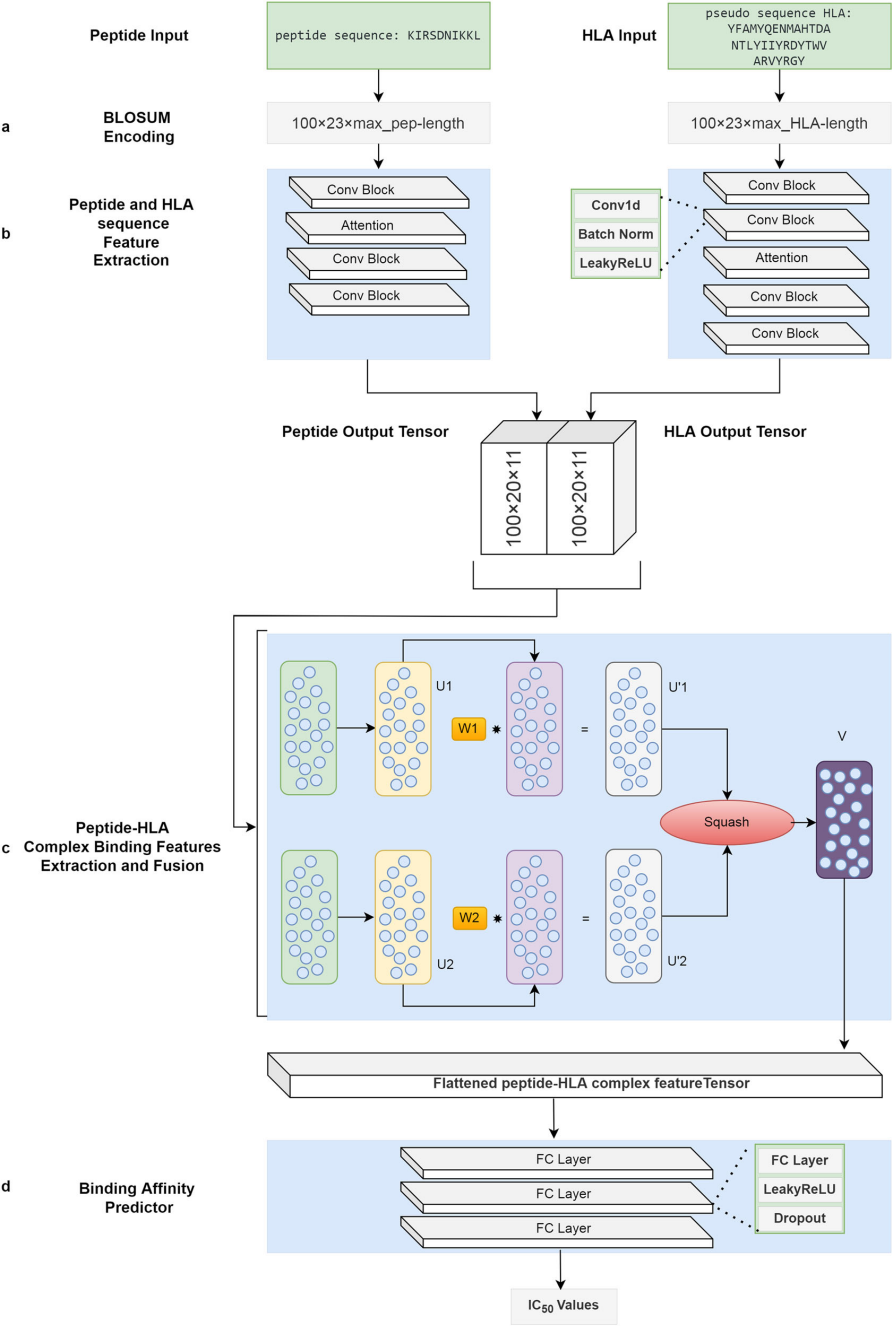
Building blocks of CapsNet-MHC. **a** Inputs encoding with Blosum matrix considering frequencies of amino acids and their substitution probabilities, **b** feature extraction by two CNN-Attention blocks, **c** feature fusion of the latent representation of MHC and peptide sequences taking advantage of a Capsule neural network, and finally, **d** extraction of predicting binding values by a fully-connected block. W, U, and U’ denote the weights matrices, input vectors to the primary capsules, and transformed vectors, respectively.

As shown in Fig. 10, the proposed method, called CapsNet-MHC, is built upon four major units: a) data encoder, b) feature extractor, c) binding dependencies extractor, and d) binding predictor, which are explained as follows.

a) Data encoder. As the first step, we use the amino acid sequence presentation of peptides and MHCs as the method’s inputs. We encode and embed each peptide and MHC sequence using the Blosum62 (Blocks Substitution Matrix) with 23 rows and columns. Assuming lengths of 15 and 385 for the peptide and MHC sequences, respectively, the peptides and MHC sequences are encoded to the 23 × 15 and 23 × 385 matrices, respectively. Moreover, assuming a batch size of 100 for feeding the encoded sequences into the model, the size of the peptide and MHC input tensors would be 100 × 23 × 15 and 100 × 23 × 385, respectively. It should be noted that the proper batch size is chosen based on the grid search for tuning the model.

b) Feature extractor. As the second step, for extracting local patterns and positional dependencies from the encoded peptide and MHC tensors, two separate CNN-Attention modules are utilized. The feature extractor for peptide sequences includes a CNN block followed by an attention layer and two CNN blocks. On the other hand, the feature extractor for MHC sequences includes two CNN blocks followed by an attention layer and two CNN blocks. The two output tensors with the size of 100 × 20 × 11 are passed into the next step. It should be noted that these modules are adopted from DeepAttentionPan with the key difference that the input and output tensors are in different shapes.

c) Binding dependencies extractor. As the key building block of CapsNet-MHC, for extracting the hierarchical binding dependencies from the peptides and MHC latent features, a CapsuleNet ^17^ is utilized. To this end, two separate primary capsules are applied to the peptide and MHC latent tensors, provided by the previous step. Specifically, the primary capsule includes a vector *u*_*i*_ (where *i* = 0, 1), delivered by the feature extractor for peptide and MHC feature vectors. Through the affine transformation, the input from the low-level capsules *u*_*i*_ is passed to the high-level capsules once being multiplied by the weights matrices. Equation (9) demonstrates the conditional transformed vectors ${\hat{u}}_{{i|j}}$ where *W* and *u* denote the weights matrices and input vectors to the primary capsules, respectively ^17^.

9 $${\hat{u}}_{i{{{{{\rm{|}}}}}}j}={W}_{{ij}}{u}_{i}$$

In this step, through the weight matrix multiplication, the spatial dependencies are encoded and the high-level features are extracted from the input vectors. Afterward, the transformed vectors ${\hat{u}}_{{i|j}}$ are multiplied by the weights learned by the dynamic routing algorithm, and so, the weighted sum of the input vectors can be calculated, as formulated in Eq. (10). In this equation, *c*_*ij*_ denotes the network weights learned by the dynamic routing algorithm.

10 $${s}_{j}={\sum }_{i}{c}_{{ij}}{\hat{u}}_{{j|i}}$$

Finally, by applying the activation function, the high-level capsule *v*_*j*_ is calculated according to Eq. (11). In this equation, *v*_*j*_ and *s*_*j*_ are the output provided by the non-linearity unit, and the one delivered by the earlier step, respectively. Moreover, the left and the right sides of the equation stand for the additional squashing, and the unit scaling of the output vector, respectively. Indeed, for information compression and reusing through the next step of CapsNet-MHC, the squash vector nonlinearity has been applied.

11 $${v}_{j}=\frac{{{{{{\rm{||}}}}}}{s}_{j}{{{{{\rm{|}}}}}}{{{{{{\rm{|}}}}}}}^{2}}{1+{{{{{\rm{||}}}}}}{s}_{j}{{{{{\rm{|}}}}}}{{{{{{\rm{|}}}}}}}^{2}}\frac{{s}_{j}}{{{{{{\rm{||}}}}}}{s}_{j}{{{{{\rm{||}}}}}}}$$

d) Binding predictor. As the final step, a three-layer fully-connected block followed by an output layer is utilized to predict the binding values between the peptide and MHC sequences.

### Statistics and reproducibility

We performed computational experiments for comparative studies of our method using K-fold cross-validation, and these experiments were repeated at least three times to ensure robustness of our results. For interpretability studies and biological insights, we utilized the seaborn library to graph charts and analyze the results.

### Reporting summary

Further information on research design is available in the Nature Portfolio Reporting Summary linked to this article.

## Supplementary information

Supplementary Information

Description of Additional Supplementary Files

Supplementary Data 1

Supplementary Data 2

Supplementary Data 3

Supplementary Data 4

Supplementary Data 5

Supplementary Data 6

Reporting Summary

## Data Availability

The datasets are available at https://github.com/s7776d/CapsNet-MHC/tree/main/dataset. Source data for graphs and charts can be found in Supplementary Data 1–6.

## Code availability

The code is available at https://github.com/s7776d/CapsNet-MHC.
