# Supplementary material for: CapsNet-MHC predicts peptide-MHC class I binding based on capsule neural networks
Source: Commun Biol. 2023 May 5;6:492. doi: 10.1038/s42003-023-04867-2 (PMC10162658; doi:10.1038/s42003-023-04867-2)
Supplement: Supplementary file 1 — Supplementary Information [file 42003_2023_4867_MOESM1_ESM.pdf]

CapsNet-MHC predicts peptide-MHC class I binding based on capsule neural networks

Supplementary Materials

Mahmood Kalemati[1], Saeid Darvishi[1] and Somayyeh Koohi[1*]

[1]Department of Computer Engineering, Sharif University of Technology, Tehran, Iran

[*]Corresponding author

E-mail: koohi@sharif.edu

Contents

Supplementary Note 1. Existing methods and tools for peptide-MHC class I binding prediction 1

Supplementary Note 2. The detailed results for IEDB's datasets 5

Supplementary Note 3. The detailed results for Anthem's datasets..15

Supplementary Note 4. The detailed results for transformers over IEDB's dataset 77

Supplementary Note 5. The detailed results for transformers over Anthem's dataset 80

Supplementary Note 6. The summarized information for benchmark datasets 119

Supplementary Note 7. Network parameter settings..120

Supplementary Figure 1. Full distribution of AUC values of HLAB and CapsNet-MHC, for various sizes of peptide k-mer (8≤k≤14)..121

Supplementary Figure 2. Full distribution of ACC, MCC, Specificity, and Sensitivity values of CapsNet-MHC, for various sizes of peptide k-mer (8≤k≤14)...122

Supplementary Figure 3. Comparing prediction performance of CapsNet-MHC and TranspHLA 123

Supplementary Figure 4. Contribution of Capsules for learning the binding features................................124

Supplementary References 125

Supplementary Note 1. Existing methods and tools for peptide-MHC class I binding prediction

Various methods and tools for the peptide-MHC binding prediction have been reviewed in the main manuscript. It should be noted that these methods can be categorized into allele-specific and pan-specific, where the method is trained for each MHC allotype, and a single method is trained on various MHC allotypes data, respectively1. Supplementary Table S1 provides summarized information for some of these methods, as well as some other peptide-MHC class I binding prediction methods and tools. For each

method and tool, the type of method (pan-specific/ allele-specific), the network architecture, the input encoding method, and their pros/cons have been shown.

Supplementary Table S1. More details for various peptide-MHC class I binding prediction methods and tools

Ref.	Method	Method Type	Network Architecture	Encoding/Embedding	More details
2	DeepHLApan: A Deep Learning Approach for Neoantigen Prediction Considering Both HLA-Peptide Binding and Immunogenicity	Pan-Specific	RNN (BiGRU) + Attention	One-hot	Able to process the variable-length sequences, and capture the long-term dependencies<br>Applies a computationally expensive BiGRU-attention architecture for training the binding model
3	MHCSeqNet: a deep neural network model for universal MHC binding prediction	Pan-Specific	RNN (GRU)	One-hot<br>Skip-Gram Model4	Able to perform binding prediction on peptides of any length and various MHC class I alleles taking advantage of a GRU-based architecture<br>Despite capturing the long-range dependencies, cannot extract valuable local information from the input sequences
5	ACME: pan-specific peptide–MHC class I binding prediction through attention-based deep neural networks	Pan-Specific	CNN + Attention	blocks substitution matrix (BLOSUM)	Able to extract important local features from the input sequences taking advantage of CNN blocks<br>Relies on manual reconstruction of the attention feature map6
7	DeepSeqPan, a novel deep convolutional neural network model for pan-specific class I HLA-peptide binding affinity prediction	Pan-Specific	CNN	One-hot	Able to learn local patterns from the input sequences<br>Limited to employ peptide sequences with 9 amino acid length

8	Peptide-Major Histocompatibility Complex Class I Binding Prediction Based on Deep Learning With Novel Feature	Allele-Specific	CNN	A 2D matrix represents the order of the sequence, hydropathy index, polarity, and sequence length	Employs various valuable physicochemical and sequence-based information<br>Relies on feature selection and engineering to construct the input matrix
9	IConMHC: a deep learning convolutional neural network model to predict peptide and MHC-I binding affinity	Pan-Specific	CNN	3D Matrix (48 × 9 × 19) 9 for peptide, 48 for MHC and 19 for the PCA component of amino acid interactions	Employs valuable physical and chemical interaction features<br>Takes advantage of PCA algorithm to capture principal component of amino acid interactions, which may lead to loss of some important information
10	MHCherryPan: a novel model to predict the binding affinity of pan-specific class I HLA-peptide	Pan-Specific	CNN + RNN (BiLSTM)	BLOSUM	Captures important local information and long-term dependencies from the input sequences<br>Unable to employ all the length of HLA (MHC) sequences, which may lead to loss of valuable sequence information
5, 6	Deep learning pan-specific model for interpretable MHC-I peptide binding prediction with improved attention mechanism	Pan-Specific	CNN + Attention	BLOSUM	Able to extract the important local and positional information from the input sequences<br>Unable to model and extract the interaction information from the learned feature tensors
11	OnionMHC: A deep learning model for peptide — HLA-A*02:01 binding predictions using both structure and sequence feature sets	Pan-Specific	CNN + RNN (LSTM)	One-hot + BLOSUM	Employs both structural and binding affinity information<br>Unable to employ whole length of HLA sequences, which may lead to loss of important information

12	A Pan-Specific GRU-Based Recurrent Neural Network for Predicting HLA-I-Binding Peptides	Pan-Specific	RNN (GRU)	one auto-embedding layer for HLA-peptide sequences embedding	Able to perform prediction for the various lengths of peptides and for all the HLA-I alleles<br>Applies a computationally expensive architecture to learn the long-term dependencies from the input sequences
13	Anthem: a user customised tool for fast and accurate prediction of binding between peptides and HLA class I molecules	Pan-Specific	Aggregating one-dependence estimators (AODE) algorithm14	Amino acid frequency (AAF) score+ WebLogo-based sequence conservation score15 + Position-specific scoring matrix (PSSM) + Position weight matrix (PWM) + BLOSUM	Prepares a new comprehensive dataset collected from the various public sources for training and evaluating the models<br>Relies on a wrapper feature selection method, and employing multiple scoring functions
16	A transformer-based model to predict peptide–HLA class I binding and optimize mutated peptides for vaccine design	Pan-Specific	A Transformer-based architecture utilizing multi-head self-attention mechanism	A Transformer-based model for positional and amino-acid embedding	Considers positional and global information of input sequences by applying a Transformer-based model<br>Utilizing a transformer-based architecture makes the model more complicated in terms of the number of parameters
17	HLAB: learning the BiLSTM features from the ProtBert-encoded proteins for the class I HLA-peptide binding prediction	Pan-Specific	ProtBert + BiLSTM+ multiple ML classifier	A ProtBert-BiLSTM model	Able to capture the long-term dependencies from the ProtBert-encoded sequences using a BiLSTM model<br>Relies on employing feature selection and dimensionality reduction algorithms for refining features and applying multiple machine learning models

Supplementary Note 2. The detailed results for IEDB's datasets

In this section, we provide detailed evaluation results of CapsNet-MHC over IEDB's datasets. For this purpose, we compare our method against 12 alternative methods, including DeepAttentionPan5, NetMHCpan 2.81, NetMHCpan 3.01, NetMHCpan 4.018, PickPocket19, SMM20, ANN 3.421, ANN 4.021, ARB22, SMMPMBEC22, IEDB Consensus22, and NetMHCcons23. Supplementary Tables S2, S3, and S4 provide detailed comparison results for these Pan-specific, Allele-specific, and Ensemble methods using AUC and SRCC metrics over 61 IEDB's benchmark datasets, respectively. It should be noted that the last row of Supplementary Table S2 (as shown in red color), represent the number of benchmark datasets for which CapsNet-MHC provides higher prediction accuracy, in terms of AUC and SRCC.

Supplementary Table S2. Detailed results for pan-specific methods over IEDB's dataset

					CapsNet-MHC		DeepAttentionPan		NetMHCpan 2.8		NetMHCpan 3.0		NetMHCpan 4.0		PickPocket	
HLA	IEDB ref	Measure	Length	Count	AUC	SRCC	AUC	SRCC	AUC	SRCC	AUC	SRCC	AUC	SRCC	AUC	SRCC
A*02:01	1026371	t1/2	9	34	0.74	0.41	0.74	0.4	0.75	0.42	0.74	0.42	0.74	0.42	0.72	0.45
A*02:01	1026371	t1/2	10	10	0.54	0.1	0.5	0.07	0.54	0.14	0.5	0.12	0.54	0.15	0.5	-0.03
B*07:02	1026371	t1/2	9	33	0.97	0.84	0.96	0.81	0.93	0.81	0.97	0.82	0.97	0.82	0.85	0.56
B*07:02	1026371	t1/2	10	19	0.9	0.7	0.9	0.71	0.88	0.69	0.9	0.68	0.9	0.64	0.8	0.61
A*02:01	1026371	binary	9	341	0.87	0.5	0.84	0.46	0.89	0.52	0.89	0.52	0.89	0.52	0.87	0.5
A*02:01	1026371	ic50	9	22	0.76	0.41	0.69	0.3	0.72	0.36	0.72	0.43	0.71	0.4	0.71	0.39
A*02:01	1026371	t1/2	9	22	0.78	0.49	0.79	0.44	0.73	0.43	0.73	0.46	0.71	0.41	0.66	0.39
A*24:02	1026371	binary	9	346	0.83	0.36	0.83	0.37	0.85	0.39	0.85	0.38	0.85	0.39	0.82	0.36
A*24:02	1026371	ic50	9	19	0.74	0.33	0.69	0.25	0.63	0.18	0.7	0.27	0.69	0.26	0.8	0.45
A*30:01	1026371	binary	9	347	0.85	0.18	0.84	0.18	0.81	0.16	0.8	0.16	0.83	0.17	0.75	0.13
A*30:02	1026371	binary	9	360	0.75	0.4	0.73	0.37	0.77	0.42	0.78	0.45	0.78	0.45	0.75	0.4
A*30:02	1026371	ic50	9	56	0.56	0.12	0.58	0.11	0.48	0.01	0.49	-0.02	0.5	-0.01	0.42	-0.1
A*30:02	1026371	t1/2	9	56	0.56	0.1	0.57	0.12	0.5	0.05	0.48	0.03	0.5	0.05	0.47	-0.01
A*68:01	1026371	binary	9	436	0.89	0.41	0.9	0.41	0.87	0.38	0.86	0.37	0.85	0.36	0.88	0.4
A*68:01	1026371	ic50	9	35	0.84	0.65	0.87	0.65	0.84	0.63	0.84	0.6	0.85	0.61	0.86	0.64
A*68:01	1026371	t1/2	9	35	0.37	-0.21	0.36	-0.19	0.32	-0.32	0.38	-0.2	0.38	-0.22	0.24	-0.46
B*07:02	1026371	binary	9	288	0.85	0.31	0.86	0.32	0.87	0.32	0.86	0.32	0.87	0.33	0.85	0.31
A*02:01	1027079	binary	9	18	0.82	0.49	0.82	0.49	0.82	0.49	0.82	0.49	0.85	0.54	0.82	0.49
A*02:01	1027471	binary	9	43	0.84	0.37	0.82	0.35	0.84	0.38	0.81	0.34	0.81	0.34	0.83	0.37
A*02:01	1027588	binary	9	18	0.84	0.58	0.86	0.6	0.83	0.56	0.84	0.58	0.83	0.56	0.75	0.42
C*03:03	1028228	ic50	9	10	0.77	0.53	0.96	0.95	0.68	0.68	0.84	0.89	0.84	0.84	0.64	0.47
A*02:01	1028285	t1/2	9	135	0.82	0.71	0.76	0.54	0.76	0.52	0.77	0.54	0.8	0.59	0.82	0.59
A*02:01	1028285	t1/2	10	36	0.8	0.4	0.83	0.73	0.78	0.61	0.8	0.67	0.8	0.67	0.74	0.55
A*02:01	1028285	t1/2	11	35	0.88	0.71	0.59	0.15	0.7	0.34	0.68	0.36	0.72	0.45	0.8	0.53
A*11:01	1028287	t1/2	9	177	0.9	0.56	0.9	0.74	0.9	0.76	0.9	0.77	0.91	0.79	0.81	0.56
A*02:01	1028553	ic50	9	22	0.91	0.65	0.9	0.55	0.92	0.62	0.85	0.59	0.85	0.57	0.83	0.58

B*07:02	1028553	ic50	9	22	0.92	0.73	0.88	0.66	0.89	0.66	0.89	0.67	0.88	0.73	0.89	0.62
A*02:01	1028554	ic50	9	44	0.91	0.65	0.86	0.65	0.89	0.7	0.88	0.71	0.88	0.72	0.88	0.51
B*07:02	1028554	ic50	9	52	0.92	0.73	0.87	0.61	0.77	0.62	0.79	0.63	0.8	0.66	0.74	0.7
B*35:01	1028554	ic50	9	56	0.91	0.63	0.72	0.35	0.68	0.36	0.67	0.4	0.66	0.36	0.7	0.28
B*44:03	1028554	ic50	9	46	0.67	0.59	0.67	0.58	0.61	0.46	0.64	0.6	0.58	0.56	0.64	0.43
B*57:01	1028554	ic50	9	53	0.84	0.47	0.84	0.49	0.86	0.62	0.87	0.64	0.88	0.6	0.85	0.44
B*27:03	315174	binary	9	10	0.79	0.5	0.92	0.71	0.92	0.71	0.83	0.57	0.92	0.71	0.88	0.64
A*02:01	1028790	ic50	10	2	-	1	-	1	-	1	-	1	-	1	-	1
A*02:03	1028790	ic50	10	2	-	-1	-	-1	-	-1	-	-1	-	-1	-	-1
A*02:06	1028790	ic50	10	2	-	1	-	1	-	1	-	1	-	1	-	1
A*68:02	1028790	ic50	10	2	-	1	-	1	-	1	-	1	-	1	-	1
A*02:01	1028928	binary	9	11	1	0.67	1	0.67	0.94	0.6	0.94	0.6	0.94	0.6	0.92	0.56
B*07:02	1028928	binary	9	11	1	0.5	1	0.5	1	0.5	1	0.5	1	0.5	1	0.5
C*03:04	315209	t1/2	9	14	1	0.94	0.98	0.9	0.91	0.78	1	0.88	1	0.86	0.87	0.72
B*57:01	1029061	ic50	9	17	1	0.68	1	0.65	0.97	0.6	1	0.58	0.97	0.56	0.97	0.72
B*27:04	1029125	binary	9	21	0.95	0.73	1	0.82	0.94	0.72	0.99	0.8	0.99	0.8	0.83	0.53
B*27:05	1029125	binary	9	21	0.96	0.75	0.97	0.77	0.96	0.75	0.95	0.73	0.94	0.72	0.91	0.67
B*27:06	1029125	binary	9	21	0.81	0.52	0.79	0.49	0.75	0.42	0.77	0.45	0.73	0.39	0.8	0.5
B*15:02	1027131	binary	9	14	1	0.71	1	0.71	1	0.71	1	0.71	1	0.71	1	0.71
A*02:01	1029824	binary	9	77	0.53	0.05	0.53	0.05	0.55	0.07	0.57	0.1	0.56	0.09	0.55	0.08
B*38:01	1029957	ic50	9	4	-	0.8	-	0.8	-	1	-	1	-	1	-	0.8
B*39:06	1029957	ic50	9	33	0.71	0.42	0.76	0.5	0.81	0.59	0.79	0.55	0.71	0.4	0.75	0.54
A*31:01	315312	binary	9	8	1	0.87	1	0.87	0.88	0.65	0.94	0.76	1	0.87	0.88	0.65
A*66:01	315312	binary	9	16	0.68	0.2	0.57	0.08	0.54	0.04	0.54	0.04	0.54	0.04	0.68	0.2
A*03:01	1031253	ic50	9	14	0.89	0.73	0.93	0.76	0.93	0.78	0.93	0.77	0.96	0.8	0.96	0.71
B*07:02	1031253	ic50	9	13	1	0.97	1	0.97	1	0,97	1	0.97	1	0.98	1	0.87
B*27:05	1031253	ic50	9	12	0.57	0.47	0.54	0.39	0.63	0,59	0.63	0.57	0.57	0.48	0.6	0.49
B*27:05	1031959	binary	8	97	0.45	-0.09	0.44	0.09	0.49	-0.02	0.49	-0.02	49	-0.02	0.49	-0.02
B*27:05	1031959	binary	9	4810	0.61	0.17	0.61	0.17	0.6	0.16	-	-	-	-	0.59	0.15

A*02:01	1033071	ic50	9	102	0.87	0.82	0.87	0.82	0.87	0.81	0.87	0.82	0.87	0.81	0.84	0.78
A*02:01	1033071	ic50	10	65	0.89	0.81	0.88	0.78	0.87	0.82	0.91	0.85	0.91	0.85	0.87	0.81
A*02:01	1031072	ic50	9	24	1	0.83	1	0.83	0.99	0.82	0.99	0.82	1	0.86	0.97	0.77
A*02:01	1031072	ic50	10	16	0.9	0.82	0.9	0.84	0.95	0.85	0.92	0.84	0.95	0.88	0.82	0.71
A*02:01	1033576	binary	9	187	0.59	0.07	0.59	0.08	0.6	0.09	0.61	0.09	0.61	0.09	0.62	0.1
A*02:01	1031894	ic50	9	3	1	0.5	1	0.5	1	0.5	1	0.5	1	0.5	1	0.5
			-	-	23	17	19	17	14	8	14	7	21	16	8	6

Supplementary Table S3. Detailed results for Allele-specific methods over IEDB's dataset

					SMM		ANN 3.4		ANN 4.0		ARB		SMMPMBEC	
HLA	IED ref	Measure	Length	Count	AUC	SRCC	AUC	SRCC	AUC	SRCC	AUC	SRCC	AUC	SRCC
A*02:01	1026371	t1/2	9	34	0.77	0.45	0.76	0.45	0.74	0.41	0.75	0.45	0.76	0.45
A*02:01	1026371	t1/2	10	10	0.5	0.07	0.54	0.15	0.46	0.05	0.42	-0.01	0.5	0.04
B*07:02	1026371	t1/2	9	33	0.93	0.72	0.93	0.77	0.9	0.72	0.72	0.44	0.93	0.74
B*07:02	1026371	t1/2	10	19	0.82	0.61	0.83	0.63	0.95	0.74	0.77	0.54	0.83	0.68
A*02:01	1026371	binary	9	341	0.88	0.51	0.87	0.49	0.89	0.53	0.88	0.51	0.88	0.51
A*02:01	1026371	ic50	9	22	0.68	0.37	0.63	0.29	0.68	0.37	0.74	0.45	0.66	0.32
A*02:01	1026371	t1/2	9	22	0.74	0.37	0.67	0.29	0.74	0.38	0.69	0.46	0.72	0.33
A*24:02	1026371	binary	9	346	0.82	0.36	0.85	0.38	0.85	0.38	0.83	0.37	0.82	0.35
A*24:02	1026371	ic50	9	19	0.76	0.39	0.61	0.19	0.63	0.22	0.45	0.03	0.77	0.36
A*30:01	1026371	binary	9	347	0.79	0.15	0.77	0.14	0.82	0.17	0.71	0.11	0.78	0.15
A*30:02	1026371	binary	9	360	0.73	0.36	0.75	0.4	0.75	0.4	0.65	0.25	0.72	0.35
A*30:02	1026371	ic50	9	56	0.57	0.12	0.6	0.13	0.54	0.03	0.66	0.27	0.55	0.07
A*30:02	1026371	t1/2	9	56	0.5	0.07	0.55	0.19	0.48	0.07	0.52	0.15	0.53	0.12
A*68:01	1026371	binary	9	436	0.86	0.37	0.87	0.38	0.89	0.4	0.78	0.34	0.88	0.39
A*68:01	1026371	ic50	9	35	0.79	0.62	0.84	0.65	0.85	0.68	0.77	0.53	0.83	0.63
A*68:01	1026371	t1/2	9	35	0.25	-0.42	0.27	-0.41	0.28	-0.4	0.31	-0.39	0.25	-0.43
B*07:02	1026371	binary	9	288	0.88	0.33	0.88	0.33	0.87	0.33	0.85	0.31	0.88	0.33
A*02:01	1027079	binary	9	18	0.77	0.42	0.78	0.44	78	0.44	0.82	0.49	0.77	0.42
A*02:01	1027471	binary	9	43	0.83	0.36	0.81	0.34	0.84	0.37	0.81	0.35	0.83	0.36
A*02:01	1027588	binary	9	18	0.84	0.58	0.82	0.55	0.86	0.6	0.75	0.43	0.86	0.6
C*03:03	1028228	ic50	9	10	0.68	0.46	0.68	0.46	0.76	0.61	-	-	0.76	0.67
A*02:01	1028285	t1/2	9	135	0.79	0.56	0.74	0.51	0.75	0.52	0.79	0.49	0.78	0.55
A*02:01	1028285	t1/2	10	36	0.82	0.59	0.75	0.54	0.8	0.66	0.86	0.73	0.81	0.59
A*02:01	1028285	t1/2	11	35	0.81	0.47	0.78	0.39	0.57	0.14	0.66	0.12	0.76	0.31
A*11:01	1028287	t1/2	9	177	0.89	0.74	0.9	0.74	0.91	0.76	0.85	0.64	0.9	0.75
A*02:01	1028553	ic50	9	22	0.85	0.59	0.91	0.7	0.92	0.63	0.9	0.69	0.83	0.62

B*07:02	1028553	ic50	9	22	0.89	0.62	0.92	0.76	0.89	0.65	0.88	0.55	0.88	0.62
A*02:01	1028554	ic50	9	44	0.89	0.58	0.82	0.62	0.81	0.6	0.76	0.51	0.86	0.55
B*07:02	1028554	ic50	9	52	0.85	0.66	0.88	0.7	0.83	0.67	0.76	0.65	0.86	0.7
B*35:01	1028554	ic50	9	56	0.59	0.21	0.57	0.27	0.69	0.27	0.64	0.26	0.53	0.2
B*44:03	1028554	ic50	9	46	0.75	0.47	0.65	0.56	0.74	0.65	0.56	0.25	0.76	0.55
B*57:01	1028554	ic50	9	53	0.77	0.33	0.94	0.52	0.96	0.53	0.63	0.12	0.77	0.29
B*27:03	315174	binary	9	10	-	-	-	-	-	-	-	-	0.5	Nan
A*02:01	1028790	ic50	10	2	-	1	-	1	-	1	-	1	-	1
A*02:03	1028790	ic50	10	2	-	1	-	-1	-	-1	-	-1	-	1
A*02:06	1028790	ic50	10	2	-	1	-	1	-	1	-	1	-	1
A*68:02	1028790	ic50	10	2	-	1	-	1	-	1	-	1	-	1
A*02:01	1028928	binary	9	11	0.94	0.6	0.94	0.6	0.94	0.6	0.94	0.6	0.94	0.6
B*07:02	1028928	binary	9	11	1	0.5	1	0.5	1	0.5	1	0.5	1	0.5
C*03:04	315209	t1/2	9	14	-	-	-	-	-	-	-	-	-	-
B*57:01	1029061	ic50	9	17	0.97	0.64	0.87	0.64	0.83	0.6	0.6	0.12	0.97	0.65
B*27:04	1029125	binary	9	21	-	-	-	-	-	-	-	-	-	-
B*27:05	1029125	binary	9	21	0.91	0.67	0.94	0.72	0.96	0.75	0.88	0.62	0.96	0.75
B*27:06	1029125	binary	9	21	-	-	-	-	-	-	-	-	-	-
B*15:02	1027131	binary	9	14	0.91	0.58	1	0.71	1	0.71	1	0.72	1	0.71
A*02:01	1029824	binary	9	77	0.57	0.1	0.57	0.11	0.58	0.12	0.52	0.04	0.57	0.1
B*38:01	1029957	ic50	9	4	-	0.8	-	0.8	-	1	-	0	-	0.8
B*39:06	1029957	ic50	9	33	-	-	-	-	-	-	-	-	-	-
A*31:01	315312	binary	9	8	0.88	0.65	0.88	0.65	0.88	0.65	0.81	0.55	0.88	0.65
A*66:01	315312	binary	9	16	0.68	0.2	0.68	0.2	0.25	-0.29	-	-	1	0.57
A*03:01	1031253	ic50	9	14	0.87	0.69	0.89	0.73	0.96	0.79	0.71	0.47	0.91	0.75
B*07:02	1031253	ic50	9	13	1	0.96	1	0.96	1	0.98	1	0.89	1	0.97
B*27:05	1031253	ic50	9	12	0.63	0.44	0.69	0.6	0.69	0.58	0.6	0.46	0.6	0.46
B*27:05	1031959	binary	8	97	0.46	-0.07	0.47	-0.06	0.48	-0.04	0.47	-0.04	0.46	-0.06
B*27:05	1031959	binary	9	4810	0.61	0.17	0.61	0.17	0.61	0.17	0.59	0.15	0.6	0.17

A*02:01	1033071	ic50	9	102	0.86	0.8	0.86	0.81	0.87	0.82	0.84	0.79	0.86	0.8
A*02:01	1033071	ic50	10	65	0.88	0.81	0.9	0.84	0.91	0.86	0.86	0.79	0.89	0.82
A*02:01	1031072	ic50	9	24	1	0.85	1	0.88	1	0.88	1	0.81	1	0.86
A*02:01	1031072	ic50	10	16	0.87	0.72	0.87	0.76	0.92	0.84	0.69	0.61	0.87	0.71
A*02:01	1033576	binary	9	187	0.6	0.09	0.61	0.1	0.65	0.12	0.54	0.04	0.6	0.09
A*02:01	1031894	ic50	9	3	1	0.5	1	0.5	1	0.5	1	0.5	1	0.5
			-	-	7	7	11	11	20	17	7	8	8	9

Supplementary Table S4. Detailed results for Ensemble methods over IEDB's dataset

					IEDB Consensus		NetMHCcons	
HLA	IED ref	Measure	Length	Count	AUC	SRCC	AUC	SRCC
A*02:01	1026371	t1/2	9	34	0.75	0.42	0.76	0.45
A*02:01	1026371	t1/2	10	10	0.52	0.11	0.5	0.09
B*07:02	1026371	t1/2	9	33	0.92	0.72	0.92	0.77
B*07:02	1026371	t1/2	10	19	0.83	0.64	0.85	0.66
A*02:01	1026371	binary	9	341	0.89	0.53	0.89	0.52
A*02:01	1026371	ic50	9	22	0.59	0.2	0.69	0.34
A*02:01	1026371	t1/2	9	22	0.67	0.24	0.71	0.39
A*24:02	1026371	binary	9	346	0.84	0.38	0.85	0.39
A*24:02	1026371	ic50	9	19	0.75	0.35	0.62	0.17
A*30:01	1026371	binary	9	347	0.73	0.12	0.79	0.15
A*30:02	1026371	binary	9	360	0.75	0.4	0.77	0.43
A*30:02	1026371	ic50	9	56	0.57	0.08	0.53	0.05
A*30:02	1026371	t1/2	9	56	0.48	0.07	0.5	0.09
A*68:01	1026371	binary	9	436	0.88	0.39	0.87	0.39
A*68:01	1026371	ic50	9	35	0.82	0.66	0.85	0.65
A*68:01	1026371	t1/2	9	35	0.26	-0.42	0.29	-0.35
B*07:02	1026371	binary	9	288	0.87	0.32	0.87	0.33
A*02:01	1027079	binary	9	18	0.75	0.4	0.82	0.49
A*02:01	1027471	binary	9	43	0.82	0.35	0.83	0.36
A*02:01	1027588	binary	9	18	0.83	0.56	0.82	0.54
C*03:03	1028228	ic50	9	10	0.68	0.46	0.68	0.56
A*02:01	1028285	t1/2	9	135	0.78	0.57	0.76	0.53
A*02:01	1028285	t1/2	10	36	0.81	0.59	0.78	0.6
A*02:01	1028285	t1/2	11	35	0.82	0.47	0.76	0.41
A*11:01	1028287	t1/2	9	177	0.89	0.73	0.9	0.76

A*02:01	1028553	ic50	9	22	0.85	0.58	0.92	0.64
B*07:02	1028553	ic50	9	22	0.88	0.67	0.91	0.72
A*02:01	1028554	ic50	9	44	0.84	0.5	0.89	0.69
B*07:02	1028554	ic50	9	52	0.82	0.59	0.86	0.73
B*35:01	1028554	ic50	9	56	0.7	0.29	0.64	0.36
B*44:03	1028554	ic50	9	46	0.84	0.56	0.64	0.55
B*57:01	1028554	ic50	9	53	0.77	0.33	0.92	0.56
B*27:03	315174	binary	9	10	-	-	0.92	0.71
A*02:01	1028790	ic50	10	2	-	1	-	1
A*02:03	1028790	ic50	10	2	-	-1	-	-1
A*02:06	1028790	ic50	10	2	-	1	-	1
A*68:02	1028790	ic50	10	2	-	1	-	1
A*02:01	1028928	binary	9	11	0.94	0.6	0.94	0.6
B*07:02	1028928	binary	9	11	1	0.52	1	0.5
C*03:04	315209	t1/2	9	14	-	-	0.91	0.78
B*57:01	1029061	ic50	9	17	0.9	0.62	0.9	0.57
B*27:04	1029125	binary	9	21	-	-	0.94	0.72
B*27:05	1029125	binary	9	21	0.91	0.68	0.96	0.75
B*27:06	1029125	binary	9	21	-	-	0.75	0.42
B*15:02	1027131	binary	9	14	0.97	0.67	1	0.71
A*02:01	1029824	binary	9	77	0.58	0.12	0.56	0.09
B*38:01	1029957	ic50	9	4	-	1	-	1
B*39:06	1029957	ic50	9	33	-	-	0.81	0.59
A*31:01	315312	binary	9	8	0.88	0.65	0.88	0.65
A*66:01	315312	binary	9	16	0.68	0.2	0.61	0.12
A*03:01	1031253	ic50	9	14	0.9	0.73	0.91	0.76
B*07:02	1031253	ic50	9	13	1	0.97	1	0.97
B*27:05	1031253	ic50	9	12	0.64	0.49	0.66	0.62
B*27:05	1031959	binary	8	97	0.45	-0.08	0.47	-0.04

B*27:05	1031959	binary	9	4810	0.6	0.16	0.6	0.17
A*02:01	1033071	ic50	9	102	0.85	0.81	0.87	0.81
A*02:01	1033071	ic50	10	65	0.88	0.85	0.89	0.83
A*02:01	1031072	ic50	9	24	1	0.86	1	0.85
A*02:01	1031072	ic50	10	16	0.87	0.81	0.87	0.77
A*02:01	1033576	binary	9	187	0.63	0.11	0.6	0..08
A*02:01	1031894	ic50	9	3	1	0.87	1	0.5
			-	-	7	8	11	12

Supplementary Note 3. The detailed results for Anthem's datasets

In this section, we provide detailed information for evaluating CapsNet-MHC over Anthem's datasets13. To this end, we compare our proposed method against 8 alternative methods, including HLAB17, Anthem13, ACME5, NetMHCpan-4.124, MHCNetSeq3, NetMHCcons-1.123, NetMHCstabpan-1.025, and MixMHCpred-2.0.226. Supplementary Table S5 provides detailed comparison results in terms of AUC, Sensitivity, Specificity, Accuracy, and MCC metrics over Anthems' datasets, for various peptide lengths and MHC (HLA) alleles.

Supplementary Table S5. Performance comparison of CapsNet-MHC against HLAB, Anthem, MixMHCpred 2.0.1, NetMHCpan 4.1, NetMHCcons 1.1, NetMHCstabpan 1.0, MHCNetSeq, and ACME on the independent test dataset

Length	HLA-I	Tool	AUC	Sensitivity	Specificity	Accuracy	MCC
8	HLA-A*01:01	HLAB	0.9945	0.9552	0.9559	0.9556	0.9111
		CapsNet-MHC	0.9912	0.9851	0.9265	0.9556	0.9127
		Anthem	0.972	0.845	0.948	0.896	0.797
		NetMHCpan-4.1	0.965	0.913	0.925	0.919	0.84
		MHCNetSeq	0.959	0.925	0.928	0.927	0.855
		NetMHCcons-1.1	0.939	0.867	0.887	0.877	0.755
		NetMHCstabpan-1.0	0.934	0.858	0.888	0.873	0.747
		MixMHCpred-2.0.2	0.928	0.86	0.884	0.872	0.744
		ACME	0.917	0.857	0.881	0.869	0.738
	HLA-A*02:01	CapsNet-MHC	0.9245	0.8599	0.8924	0.8762	0.7527
		HLAB	0.9188	0.8862	0.8797	0.873	0.746
		Anthem	0.911	0.806	0.902	0.854	0.712
		ACME	0.911	0.85	0.85	0.85	0.7
		NetMHCcons-1.1	0.907	0.823	0.878	0.85	0.702
		NetMHCstabpan-1.0	0.903	0.826	0.868	0.847	0.695
		MixMHCpred-2.0.2	0.901	0.842	0.845	0.844	0.688
		NetMHCpan-4.1	0.901	0.823	0.877	0.85	0.701
		MHCNetSeq	0.887	0.798	0.871	0.835	0.672
	HLA-A*03:01	HLAB	0.9825	0.9474	0.9483	0.9478	0.8956
		NetMHCpan-4.1	0.976	0.965	0.963	0.964	0.928
		NetMHCcons-1.1	0.975	0.963	0.974	0.968	0.937
		NetMHCstabpan-1.0	0.975	0.965	0.967	0.966	0.932
		CapsNet-MHC	0.9749	0.9298	0.9138	0.9217	0.8436
		MixMHCpred-2.0.2	0.972	0.965	0.907	0.936	0.874
		Anthem	0.968	0.947	0.93	0.939	0.878
		ACME	0.963	0.942	0.959	0.951	0.902
		MHCNetSeq	0.947	0.931	0.895	0.913	0.827

HLA-A*11:01	MHCNetSeq	0.979	0.935	0.971	0.953	0.91
	NetMHCstabpan-1.0	0.974	0.959	0.918	0.938	0.884
	NetMHCcons-1.1	0.972	0.941	0.918	0.929	0.865
	NetMHCpan-4.1	0.968	0.9	0.935	0.918	0.837
	HLAB	0.9575	0.8824	0.8333	0.8571	0.7157
	ACME	0.956	0.894	0.929	0.912	0.826
	CapsNet-MHC	0.9542	0.7647	0.8889	0.8286	0.6601
	Anthem	0.934	0.788	0.918	0.853	0.715
	MixMHCpred-2.0.2	0.915	0.888	0.882	0.885	0.773
HLA-A*24:02	NetMHCpan-4.1	0.972	0.959	0.918	0.939	0.879
	NetMHCcons-1.1	0.969	0.939	0.923	0.931	0.862
	NetMHCstabpan-1.0	0.969	0.948	0.905	0.926	0.853
	CapsNet-MHC	0.9641	0.9091	0.8667	0.8871	0.7761
	HLAB	0.9611	0.8409	0.9333	0.8876	0.7782
	MixMHCpred-2.0.2	0.961	0.914	0.927	0.92	0.841
	Anthem	0.959	0.85	0.929	0.889	0.785
	ACME	0.959	0.902	0.914	0.908	0.818
	MHCNetSeq	0.94	0.886	0.898	0.892	0.785
HLA-A*29:02	CapsNet-MHC	0.989	0.975	0.9268	0.9506	0.9024
	HLAB	0.9847	0.9669	0.9396	0.9532	0.9067
	NetMHCpan-4.1	0.982	0.99	0.945	0.968	0.936
	NetMHCcons-1.1	0.982	0.972	0.942	0.958	0.916
	NetMHCstabpan-1.0	0.979	0.967	0.94	0.954	0.909
	ACME	0.973	0.93	0.935	0.932	0.865
	MixMHCpred-2.0.2	0.969	0.91	0.963	0.936	0.874
	Anthem	0.96	0.92	0.895	0.907	0.818
	MHCNetSeq	0.941	0.875	0.878	0.876	0.754
HLA-B*07:02	NetMHCpan-4.1	0.986	0.944	0.959	0.952	0.903
	HLAB	0.9847	0.9669	0.9396	0.9532	0.9068
	CapsNet-MHC	0.9829	0.9337	0.956	0.9449	0.89
	NetMHCcons-1.1	0.981	0.942	0.941	0.941	0.883
	NetMHCstabpan-1.0	0.981	0.936	0.949	0.943	0.885
	MixMHCpred-2.0.2	0.98	0.924	0.944	0.934	0.869
	ACME	0.98	0.942	0.945	0.944	0.887
	MHCNetSeq	0.98	0.918	0.963	0.941	0.883
	Anthem	0.975	0.944	0.955	0.949	0.898
HLA-B*08:01	CapsNet-MHC	0.9952	0.9697	0.9612	0.9654	0.9309
	NetMHCpan-4.1	0.994	0.982	0.977	0.98	0.959
	HLAB	0.9912	0.9913	0.9655	0.9784	0.9571
	Anthem	0.99	0.951	0.959	0.955	0.91
	NetMHCcons-1.1	0.99	0.974	0.965	0.97	0.939
	NetMHCstabpan-1.0	0.989	0.976	0.961	0.968	0.936
	MixMHCpred-2.0.2	0.987	0.937	0.954	0.945	0.891

	ACME	0.984	0.977	0.958	0.967	0.935
	MHCNetSeq	0.979	0.954	0.933	0.943	0.887
HLA-B*13:02	CapsNet-MHC	1	1	1	1	1
	HLAB	1	1	1	1	1
	NetMHCpan-4.1	0.994	0.982	0.977	0.98	0.959
	NetMHCstabpan-1.0	0.995	1	0.993	0.996	0.993
	NetMHCcons-1.1	0.994	1	0.993	0.996	0.993
	MixMHCpred-2.0.2	0.986	0.922	0.993	0.957	0.918
	ACME	0.978	0.993	0.957	0.975	0.951
	MHCNetSeq	0.971	0.964	0.907	0.936	0.876
	Anthem	0.956	0.857	0.999	0.928	0.864
HLA-B*14:02	NetMHCpan-4.1	0.996	0.993	0.983	0.988	0.976
	Anthem	0.995	0.924	0.975	0.95	0.901
	HLAB	0.9938	0.9811	0.9813	0.9812	0.9624
	NetMHCstabpan-1.0	0.993	0.979	0.975	0.977	0.955
	CapsNet-MHC	0.9913	0.9811	0.9533	0.9671	0.9347
	NetMHCcons-1.1	0.991	0.978	0.97	0.974	0.948
	MixMHCpred-2.0.2	0.989	0.932	0.966	0.949	0.899
	ACME	0.984	0.949	0.949	0.949	0.898
	MHCNetSeq	0.957	0.962	0.862	0.912	0.829
HLA-B*15:01	NetMHCpan-4.1	0.985	0.942	0.959	0.95	0.901
	MHCNetSeq	0.985	0.944	0.942	0.943	0.886
	Anthem	0.981	0.92	0.954	0.937	0.874
	MixMHCpred-2.0.2	0.981	0.95	0.933	0.941	0.883
	CapsNet-MHC	0.978	0.9643	0.9381	0.9511	0.9026
	NetMHCstabpan-1.0	0.975	0.923	0.944	0.934	0.868
	HLAB	0.9745	0.9375	0.885	0.9111	0.8234
	NetMHCcons-1.1	0.973	0.912	0.945	0.928	0.857
	ACME	0.973	0.921	0.961	0.941	0.883
HLA-B*18:01	Anthem	0.991	0.96	0.987	0.973	0.948
	ACME	0.991	0.983	0.991	0.987	0.974
	NetMHCcons-1.1	0.989	0.981	0.988	0.984	0.969
	CapsNet-MHC	0.9877	0.981	0.9434	0.9621	0.9248
	MixMHCpred-2.0.2	0.987	0.971	0.983	0.977	0.954
	NetMHCpan-4.1	0.987	0.977	0.991	0.984	0.969
	NetMHCstabpan-1.0	0.987	0.976	0.98	0.978	0.956
	HLAB	0.9745	0.9375	0.885	0.9111	0.8234
	MHCNetSeq	0.984	0.972	0.95	0.961	0.923
HLA-B*18:03	CapsNet-MHC	1	1	0.9545	0.9767	0.9545
	HLAB	1	1	0.9545	0.9767	0.9545
	MixMHCpred-2.0.2	0.999	1	0.981	0.99	0.981
	NetMHCstabpan-1.0	0.998	1	0.995	0.998	0.995
	NetMHCpan-4.1	0.997	1	0.995	0.998	0.995

	NetMHCcons-1.1	0.997	1	0.995	0.998	0.995
	ACME	0.996	0.99	0.986	0.988	0.976
	MHCNetSeq	0.994	1	0.928	0.964	0.932
	Anthem	0.978	0.933	0.984	0.958	0.919
HLA-B*27:05	CapsNet-MHC	0.8673	0.7821	0.7962	0.7891	0.5783
	Anthem	0.832	0.697	0.778	0.738	0.478
	HLAB	0.8317	0.8013	0.758	0.7796	0.5597
	MHCNetSeq	0.727	0.608	0.737	0.673	0.348
	NetMHCstabpan-1.0	0.723	0.588	0.768	0.678	0.363
	NetMHCcons-1.1	0.717	0.588	0.74	0.664	0.334
	MixMHCpred-2.0.2	0.716	0.578	0.753	0.665	0.339
	NetMHCpan-4.1	0.713	0.623	0.733	0.678	0.358
	ACME	0.704	0.573	0.783	0.678	0.367
HLA-B*27:09	HLAB	0.9875	0.95	0.9048	0.9268	0.8548
	CapsNet-MHC	0.9333	0.85	0.8095	0.8293	0.6595
	Anthem	0.907	0.75	0.881	0.816	0.647
	NetMHCpan-4.1	0.837	0.86	0.765	0.812	0.632
	MixMHCpred-2.0.2	0.79	0.7	0.79	0.745	0.495
	NetMHCcons-1.1	0.678	0.765	0.63	0.698	0.405
	NetMHCstabpan-1.0	0.676	0.77	0.62	0.695	0.399
	MHCNetSeq	0.559	0.55	0.6	0.575	0.156
	ACME	0.558	0.685	0.51	0.598	0.201
HLA-B*35:01	HLAB	0.9512	0.8261	0.8936	0.8602	0.7217
	CapsNet-MHC	0.95	0.8261	0.8936	0.8602	0.7217
	Anthem	0.927	0.822	0.911	0.867	0.737
	MHCNetSeq	0.912	0.913	0.839	0.876	0.755
	NetMHCpan-4.1	0.911	0.819	0.907	0.863	0.729
	MixMHCpred-2.0.2	0.897	0.802	0.885	0.844	0.693
	NetMHCstabpan-1.0	0.895	0.808	0.902	0.856	0.714
	NetMHCcons-1.1	0.894	0.811	0.885	0.848	0.698
	ACME	0.891	0.792	0.9	0.846	0.697
HLA-B*37:01	CapsNet-MHC	0.9993	0.9918	0.9756	0.9837	0.9675
	NetMHCpan-4.1	0.996	0.996	0.989	0.993	0.985
	NetMHCstabpan-1.0	0.996	0.988	0.972	0.98	0.961
	NetMHCcons-1.1	0.993	0.975	0.965	0.97	0.94
	HLAB	0.9923	0.9918	0.9837	0.9878	0.9755
	Anthem	0.992	0.928	0.985	0.957	0.915
	ACME	0.99	0.959	0.956	0.957	0.915
	MixMHCpred-2.0.2	0.942	0.875	0.859	0.867	0.735
	MHCNetSeq	0.873	0.79	0.813	0.802	0.604
HLA-B*39:01	CapsNet-MHC	0.9987	0.8889	1	0.9455	0.896
	NetMHCcons-1.1	0.992	0.981	0.974	0.978	0.956
	ACME	0.992	1	0.967	0.983	0.968

	NetMHCpan-4.1	0.99	0.97	0.959	0.964	0.931
	NetMHCstabpan-1.0	0.988	0.963	0.959	0.961	0.923
	MHCNetSeq	0.982	0.941	0.937	0.939	0.879
	HLAB	0.9683	0.8148	0.9643	0.8909	0.7896
	MixMHCpred-2.0.2	0.956	0.874	0.911	0.892	0.787
	Anthem	0.945	0.778	0.976	0.877	0.769
HLA-B*39:24	CapsNet-MHC	1	0.92	1	0.9608	0.9243
	HLAB	1	1	1	1	1
	MixMHCpred-2.0.2	0.998	1	0.988	0.994	0.988
	NetMHCpan-4.1	0.992	0.976	0.968	0.972	0.944
	Anthem	0.991	0.984	0.962	0.973	0.948
	NetMHCcons-1.1	0.99	1	0.972	0.986	0.973
	NetMHCstabpan-1.0	0.99	1	0.968	0.984	0.969
	ACME	0.985	0.992	0.956	0.974	0.95
	MHCNetSeq	0.973	0.932	0.924	0.928	0.856
HLA-B*40:01	NetMHCpan-4.1	0.995	0.995	0.976	0.986	0.972
	CapsNet-MHC	0.9935	1	0.9545	0.9767	0.9545
	Anthem	0.993	0.99	0.97	0.98	0.962
	NetMHCcons-1.1	0.991	0.986	0.962	0.974	0.948
	NetMHCstabpan-1.0	0.991	0.995	0.962	0.979	0.959
	ACME	0.991	0.981	0.957	0.969	0.939
	HLAB	0.9848	1	0.9545	0.9767	0.9545
	MixMHCpred-2.0.2	0.982	0.957	0.947	0.952	0.907
	MHCNetSeq	0.965	0.919	0.928	0.924	0.85
HLA-B*40:02	CapsNet-MHC	0.9923	0.9632	0.96	0.9616	0.9232
	Anthem	0.989	0.957	0.967	0.962	0.923
	MixMHCpred-2.0.2	0.989	0.957	0.969	0.963	0.926
	NetMHCpan-4.1	0.985	0.965	0.952	0.958	0.917
	HLAB	0.9829	0.9766	0.8867	0.9316	0.8667
	NetMHCcons-1.1	0.98	0.953	0.942	0.947	0.895
	NetMHCstabpan-1.0	0.978	0.949	0.928	0.938	0.878
	ACME	0.976	0.948	0.924	0.936	0.873
	MHCNetSeq	0.971	0.98	0.788	0.884	0.782
HLA-B*44:02	CapsNet-MHC	0.9966	0.931	0.9667	0.9492	0.8987
	HLAB	0.9954	0.931	1	0.9661	0.9343
	MixMHCpred-2.0.2	0.991	0.969	0.969	0.969	0.938
	NetMHCpan-4.1	0.989	0.976	0.955	0.966	0.931
	NetMHCcons-1.1	0.987	0.963	0.966	0.964	0.928
	NetMHCstabpan-1.0	0.986	0.938	0.962	0.95	0.901
	ACME	0.978	0.935	0.931	0.933	0.866
	Anthem	0.97	0.848	0.947	0.898	0.8
	MHCNetSeq	0.952	0.938	0.907	0.923	0.847
HLA-B*44:03	MHCNetSeq	0.989	0.947	0.971	0.959	0.918

	NetMHCpan-4.1	0.986	0.957	0.971	0.964	0.929
	NetMHCcons-1.1	0.983	0.961	0.971	0.966	0.933
	CapsNet-MHC	0.9815	0.9643	0.931	0.9474	0.8953
	HLAB	0.9729	0.9643	0.8621	0.9123	0.8294
	ACME	0.978	0.957	0.971	0.964	0.929
	Anthem	0.976	0.964	0.941	0.953	0.907
	NetMHCstabpan-1.0	0.973	0.961	0.979	0.969	0.94
	MixMHCpred-2.0.2	0.965	0.957	0.971	0.964	0.929
HLA-B*46:01	CapsNet-MHC	0.9762	1	0.8571	0.9268	0.8633
	MixMHCpred-2.0.2	0.975	0.9	0.945	0.923	0.847
	NetMHCpan-4.1	0.97	0.96	0.915	0.938	0.876
	HLAB	0.9667	1	0.9048	0.9512	0.9069
	MHCNetSeq	0.956	0.89	0.925	0.908	0.817
	NetMHCstabpan-1.0	0.952	0.945	0.895	0.92	0.843
	NetMHCcons-1.1	0.944	0.935	0.875	0.905	0.815
	ACME	0.923	0.87	0.855	0.863	0.728
	Anthem	0.92	0.82	0.884	0.852	0.712
HLA-B*49:01	CapsNet-MHC	0.9985	0.9766	0.9845	0.9805	0.9611
	NetMHCpan-4.1	0.997	0.992	0.985	0.988	0.978
	Anthem	0.993	0.977	0.976	0.976	0.952
	NetMHCcons-1.1	0.993	0.975	0.986	0.981	0.961
	NetMHCstabpan-1.0	0.993	0.98	0.978	0.979	0.959
	HLAB	0.9918	0.9766	0.9922	0.9844	0.969
	ACME	0.989	0.978	0.966	0.972	0.944
	MixMHCpred-2.0.2	0.988	0.965	0.972	0.969	0.937
	MHCNetSeq	0.97	0.977	0.848	0.912	0.832
HLA-B*51:01	NetMHCpan-4.1	0.988	0.966	0.967	0.967	0.933
	CapsNet-MHC	0.9856	1	0.9444	0.9714	0.9444
	Anthem	0.98	0.936	0.952	0.944	0.888
	NetMHCcons-1.1	0.974	0.939	0.95	0.944	0.889
	HLAB	0.9732	0.9638	0.9414	0.9526	0.9054
	MixMHCpred-2.0.2	0.972	0.939	0.95	0.944	0.889
	NetMHCstabpan-1.0	0.972	0.937	0.942	0.939	0.879
	ACME	0.948	0.873	0.934	0.904	0.81
	MHCNetSeq	0.943	0.873	0.902	0.888	0.776
HLA-B*51:08	CapsNet-MHC	1	1	0.9444	0.9714	0.9444
	MixMHCpred-2.0.2	0.998	0.994	0.982	0.988	0.977
	NetMHCpan-4.1	0.997	1	0.982	0.991	0.983
	Anthem	0.987	0.976	0.985	0.981	0.962
	NetMHCcons-1.1	0.984	0.971	0.959	0.965	0.93
	NetMHCstabpan-1.0	0.984	0.988	0.959	0.974	0.948
	MHCNetSeq	0.976	1	0.353	0.677	0.461
	HLAB	0.9739	1	0.9444	0.9714	0.9444

	ACME	0.969	0.935	0.959	0.947	0.895
HLA-B*52:01	Anthem	0.982	0.942	0.985	0.964	0.928
	HLAB	0.9727	0.9737	0.974	0.9739	0.9477
	NetMHCpan-4.1	0.972	0.965	0.955	0.96	0.92
	MixMHCpred-2.0.2	0.971	0.927	0.957	0.942	0.885
	CapsNet-MHC	0.9694	0.9605	0.961	0.9608	0.9216
	NetMHCstabpan-1.0	0.965	0.922	0.946	0.934	0.869
	NetMHCcons-1.1	0.957	0.929	0.931	0.93	0.861
	ACME	0.915	0.842	0.874	0.858	0.717
	MHCNetSeq	0.883	0.856	0.781	0.819	0.641
HLA-B*54:01	HLAB	0.9823	0.8	1	0.902	0.8191
	CapsNet-MHC	0.9508	0.84	1	0.9216	0.8532
	ACME	0.908	0.856	0.944	0.9	0.804
	Anthem	0.904	0.792	0.962	0.877	0.767
	NetMHCpan-4.1	0.896	0.84	0.972	0.906	0.82
	MixMHCpred-2.0.2	0.892	0.84	0.96	0.9	0.806
	NetMHCcons-1.1	0.87	0.84	0.98	0.91	0.829
	NetMHCstabpan-1.0	0.86	0.84	0.984	0.912	0.833
	MHCNetSeq	0.788	0.744	0.732	0.738	0.477
HLA-B*57:01	HLAB	0.9571	0.8642	0.9268	0.9018	0.8058
	CapsNet-MHC	0.9429	0.8765	0.939	0.9018	0.8046
	Anthem	0.923	0.81	0.933	0.871	0.749
	MHCNetSeq	0.909	0.812	0.875	0.844	0.692
	NetMHCpan-4.1	0.893	0.805	0.874	0.84	0.682
	ACME	0.884	0.77	0.922	0.847	0.701
	NetMHCcons-1.1	0.875	0.78	0.815	0.798	0.597
	NetMHCstabpan-1.0	0.87	0.766	0.84	0.802	0.608
	MixMHCpred-2.0.2	0.863	0.779	0.907	0.843	0.693
HLA-B*57:03	CapsNet-MHC	0.9722	0.9298	0.9483	0.9391	0.8784
	HLAB	0.9595	0.9649	0.9138	0.9391	0.8795
	Anthem	0.958	0.895	0.924	0.909	0.82
	NetMHCpan-4.1	0.927	0.872	0.868	0.87	0.741
	ACME	0.905	0.8	0.89	0.845	0.692
	NetMHCstabpan-1.0	0.871	0.81	0.789	0.8	0.602
	NetMHCcons-1.1	0.867	0.83	0.77	0.8	0.602
	MHCNetSeq	0.858	0.777	0.807	0.792	0.585
HLA-B*58:01	HLAB	0.9938	0.9481	1	0.9742	0.9496
	Anthem	0.988	0.945	0.936	0.941	0.882
	CapsNet-MHC	0.9875	0.961	0.9359	0.9484	0.8971
	ACME	0.973	0.943	0.949	0.946	0.892
	NetMHCpan-4.1	0.971	0.914	0.929	0.921	0.844
	MHCNetSeq	0.969	0.891	0.949	0.92	0.842
	MixMHCpred-2.0.2	0.964	0.893	0.909	0.902	0.804

	NetMHCcons-1.1	0.96	0.904	0.923	0.914	0.827
	NetMHCstabpan-1.0	0.959	0.903	0.925	0.914	0.828
HLA-C*01:02	CapsNet-MHC	0.9758	0.963	0.8909	0.9266	0.8556
	NetMHCpan-4.1	0.969	0.937	0.935	0.936	0.873
	HLAB	0.964	0.963	0.9091	0.9358	0.8729
	NetMHCcons-1.1	0.963	0.903	0.946	0.925	0.851
	Anthem	0.956	0.855	0.924	0.89	0.783
	NetMHCstabpan-1.0	0.954	0.917	0.902	0.909	0.819
	MixMHCpred-2.0.2	0.941	0.9	0.839	0.869	0.741
	MHCNetSeq	0.761	0.833	0.485	0.659	0.339
HLA-C*02:02	Anthem	0.989	0.933	0.95	0.942	0.886
	CapsNet-MHC	0.9833	0.9167	0.92	0.9184	0.8367
	MixMHCpred-2.0.2	0.98	0.958	0.983	0.971	0.942
	HLAB	0.9583	1	0.92	0.9592	0.9215
	NetMHCcons-1.1	0.948	0.871	0.917	0.894	0.79
	NetMHCstabpan-1.0	0.946	0.921	0.863	0.892	0.786
	NetMHCpan-4.1	0.943	0.896	0.875	0.886	0.772
	MHCNetSeq	0.857	0.762	0.746	0.754	0.51
HLA-C*03:03	CapsNet-MHC	0.9723	0.9412	0.9143	0.9275	0.8555
	NetMHCstabpan-1.0	0.958	0.924	0.944	0.934	0.868
	NetMHCpan-4.1	0.957	0.924	0.938	0.931	0.862
	MixMHCpred-2.0.2	0.956	0.909	0.968	0.938	0.878
	NetMHCcons-1.1	0.953	0.918	0.935	0.926	0.854
	HLAB	0.9462	0.9412	0.8857	0.913	0.8276
	Anthem	0.945	0.865	0.954	0.909	0.823
	MHCNetSeq	0.944	0.938	0.891	0.915	0.831
HLA-C*03:04	CapsNet-MHC	0.997	0.9825	0.9655	0.9739	0.948
	MixMHCpred-2.0.2	0.995	0.96	0.979	0.969	0.939
	Anthem	0.993	0.951	0.962	0.957	0.914
	NetMHCpan-4.1	0.992	0.963	0.965	0.964	0.928
	HLAB	0.9903	0.9649	0.931	0.9478	0.8962
	NetMHCcons-1.1	0.986	0.965	0.951	0.958	0.916
	NetMHCstabpan-1.0	0.986	0.963	0.949	0.956	0.913
	MHCNetSeq	0.971	0.926	0.9	0.913	0.827
HLA-C*04:01	CapsNet-MHC	0.9746	0.8671	0.9497	0.9085	0.8197
	Anthem	0.961	0.866	0.955	0.91	0.824
	MixMHCpred-2.0.2	0.954	0.897	0.916	0.906	0.813
	NetMHCpan-4.1	0.953	0.878	0.927	0.902	0.805
	HLAB	0.9434	0.8797	0.956	0.918	0.8383
	NetMHCstabpan-1.0	0.939	0.849	0.91	0.879	0.76
	NetMHCcons-1.1	0.923	0.841	0.864	0.853	0.706
	MHCNetSeq	0.85	0.74	0.823	0.782	0.566
HLA-C*05:01	CapsNet-MHC	0.9981	0.9931	0.9448	0.9689	0.9388

	Anthem	0.997	0.976	0.983	0.98	0.959
	MixMHCpred-2.0.2	0.997	0.973	0.987	0.98	0.96
	NetMHCpan-4.1	0.997	0.989	0.98	0.984	0.969
	NetMHCstabpan-1.0	0.996	0.983	0.974	0.979	0.958
	NetMHCcons-1.1	0.995	0.985	0.969	0.977	0.954
	HLAB	0.9902	0.9861	0.9517	0.9689	0.9383
	MHCNetSeq	0.927	0.993	0.451	0.722	0.528
HLA-C*06:02	HLAB	0.981	0.9714	0.9444	0.9577	0.9159
	MixMHCpred-2.0.2	0.981	0.943	0.983	0.963	0.927
	CapsNet-MHC	0.9762	0.8286	0.9167	0.8732	0.7488
	NetMHCcons-1.1	0.97	0.943	0.911	0.927	0.855
	NetMHCpan-4.1	0.968	0.903	0.917	0.91	0.821
	Anthem	0.958	0.829	0.95	0.889	0.79
	NetMHCstabpan-1.0	0.957	0.914	0.871	0.893	0.788
	MHCNetSeq	0.734	0.729	0.711	0.72	0.442
HLA-C*07:01	CapsNet-MHC	0.9947	0.9778	0.9384	0.956	0.913
	NetMHCpan-4.1	0.988	0.958	0.973	0.966	0.932
	NetMHCstabpan-1.0	0.987	0.98	0.949	0.965	0.93
	NetMHCcons-1.1	0.98	0.962	0.92	0.941	0.884
	MixMHCpred-2.0.2	0.969	0.942	0.902	0.922	0.846
	Anthem	0.965	0.924	0.913	0.919	0.838
	HLAB	0.9367	0.9778	0.8696	0.9231	0.8515
	MHCNetSeq	0.845	0.785	0.78	0.782	0.567
HLA-C*07:02	HLAB	0.9997	0.9811	0.9815	0.9813	0.9626
	Anthem	0.996	0.977	0.969	0.973	0.947
	CapsNet-MHC	0.9986	0.9811	0.963	0.972	0.9441
	NetMHCpan-4.1	0.996	0.964	0.977	0.971	0.942
	MixMHCpred-2.0.2	0.995	0.994	0.973	0.984	0.968
	NetMHCcons-1.1	0.994	0.975	0.966	0.971	0.942
	NetMHCstabpan-1.0	0.99	0.96	0.956	0.958	0.918
	MHCNetSeq	0.948	0.876	0.855	0.865	0.731
HLA-C*07:04	HLAB	1	1	1	1	1
	NetMHCpan-4.1	0.993	0.995	0.989	0.992	0.985
	NetMHCstabpan-1.0	0.988	1	0.974	0.987	0.975
	CapsNet-MHC	0.9816	1	0.95	0.9744	0.95
	Anthem	0.979	0.853	0.948	0.9	0.806
	NetMHCcons-1.1	0.979	0.989	0.958	0.974	0.949
	MHCNetSeq	0.737	0.81	0.584	0.697	0.407
HLA-C*08:02	CapsNet-MHC	1	1	0.9939	0.9969	0.9939
	MixMHCpred-2.0.2	0.999	0.992	0.989	0.991	0.981
	Anthem	0.998	0.985	0.985	0.985	0.97
	NetMHCpan-4.1	0.998	1	0.988	0.994	0.988
	HLAB	0.997	0.9691	1	0.9846	0.9697

	NetMHCcons-1.1	0.995	0.979	0.975	0.977	0.954
	NetMHCstabpan-1.0	0.995	0.976	0.971	0.974	0.948
	MHCNetSeq	0.899	0.944	0.661	0.803	0.632
HLA-C*12:03	MixMHCpred-2.0.2	0.992	0.967	0.96	0.963	0.927
	CapsNet-MHC	0.9871	1	0.9032	0.9508	0.9062
	NetMHCpan-4.1	0.981	0.98	0.943	0.962	0.925
	NetMHCcons-1.1	0.976	0.967	0.927	0.947	0.896
	NetMHCstabpan-1.0	0.97	0.95	0.92	0.935	0.871
	Anthem	0.969	0.926	0.955	0.941	0.883
	HLAB	0.9634	1	0.8065	0.9016	0.8198
	MHCNetSeq	0.767	0.726	0.707	0.717	0.435
HLA-C*14:02	MixMHCpred-2.0.2	1	0.998	0.995	0.996	0.993
	CapsNet-MHC	0.9998	1	0.9694	0.9846	0.9697
	Anthem	0.999	0.988	0.996	0.992	0.983
	NetMHCpan-4.1	0.999	0.997	0.993	0.995	0.99
	HLAB	0.9977	0.9897	0.9898	0.9897	0.9795
	NetMHCcons-1.1	0.994	0.988	0.965	0.976	0.953
	NetMHCstabpan-1.0	0.993	0.995	0.956	0.975	0.952
	MHCNetSeq	0.603	0.691	0.503	0.597	0.197
HLA-C*15:02	CapsNet-MHC	0.9936	1	0.925	0.962	0.9268
	NetMHCpan-4.1	0.992	1	0.964	0.982	0.965
	MixMHCpred-2.0.2	0.99	0.949	0.951	0.95	0.9
	Anthem	0.989	0.923	0.957	0.94	0.882
	HLAB	0.9878	1	0.925	0.962	0.9268
	NetMHCcons-1.1	0.984	0.982	0.956	0.969	0.939
	NetMHCstabpan-1.0	0.983	0.99	0.941	0.965	0.932
	MHCNetSeq	0.873	0.816	0.841	0.828	0.658
HLA-C*16:01	CapsNet-MHC	0.9892	0.9806	0.9103	0.9453	0.8929
	NetMHCpan-4.1	0.988	0.969	0.941	0.955	0.911
	Anthem	0.987	0.942	0.958	0.95	0.9
	MixMHCpred-2.0.2	0.984	0.936	0.946	0.942	0.883
	HLAB	0.9796	0.9613	0.9423	0.9518	0.9037
	NetMHCstabpan-1.0	0.949	0.936	0.875	0.906	0.813
	NetMHCcons-1.1	0.947	0.929	0.873	0.901	0.804
	MHCNetSeq	0.844	0.884	0.477	0.68	0.395
HLA-C*17:01	MixMHCpred-2.0.2	0.988	0.964	0.964	0.964	0.929
	NetMHCpan-4.1	0.981	0.96	0.94	0.95	0.901
	Anthem	0.972	0.808	0.946	0.877	0.762
	NetMHCstabpan-1.0	0.971	0.932	0.932	0.932	0.865
	CapsNet-MHC	0.9499	1	0.8077	0.902	0.8204
	NetMHCcons-1.1	0.948	0.912	0.924	0.918	0.838
	HLAB	0.9477	0.96	0.8077	0.8824	0.7749
	MHCNetSeq	0.778	0.724	0.76	0.742	0.491

9	HLA-A*01:01	CapsNet-MHC	0.9872	0.9406	0.9589	0.9497	0.8996
		NetMHCpan-4.1	0.985	0.952	0.954	0.953	0.906
		NetMHCcons-1.1	0.983	0.941	0.946	0.944	0.887
		NetMHCstabpan-1.0	0.983	0.935	0.949	0.942	0.884
		Anthem	0.982	0.938	0.959	0.949	0.897
		ACME	0.979	0.934	0.942	0.938	0.877
		HLAB	0.978	0.9416	0.9522	0.9469	0.8938
		MixMHCpred-2.0.2	0.976	0.937	0.943	0.94	0.88
		DeepSeqPan	0.968	0.906	0.929	0.918	0.836
		MHCNetSeq	0.958	0.898	0.904	0.901	0.802
	HLA-A*02:01	CapsNet-MHC	0.964	0.9088	0.9085	0.9086	0.8173
		HLAB	0.9599	0.9005	0.9183	0.9094	0.819
		Anthem	0.956	0.877	0.92	0.899	0.799
		NetMHCpan-4.1	0.956	0.89	0.906	0.898	0.796
		NetMHCstabpan-1.0	0.956	0.892	0.903	0.897	0.795
		NetMHCcons-1.1	0.955	0.887	0.907	0.897	0.794
		ACME	0.951	0.891	0.911	0.901	0.803
		DeepSeqPan	0.944	0.875	0.894	0.885	0.77
		MixMHCpred-2.0.2	0.943	0.87	0.89	0.88	0.76
		MHCNetSeq	0.938	0.875	0.902	0.888	0.777
	HLA-A*02:02	CapsNet-MHC	0.9659	0.9106	0.901	0.9058	0.8116
		HLAB	0.9645	0.9238	0.9175	0.9207	0.8413
		MixMHCpred-2.0.2	0.963	0.931	0.895	0.913	0.827
		ACME	0.961	0.943	0.896	0.919	0.839
		Anthem	0.952	0.951	0.864	0.907	0.818
		NetMHCstabpan-1.0	0.951	0.91	0.877	0.894	0.789
		NetMHCpan-4.1	0.95	0.92	0.879	0.9	0.801
		NetMHCcons-1.1	0.95	0.894	0.887	0.891	0.782
		DeepSeqPan	0.941	0.897	0.871	0.884	0.769
		MHCNetSeq	0.905	0.853	0.862	0.858	0.716
	HLA-A*02:03	CapsNet-MHC	0.9778	0.9522	0.9046	0.9284	0.8578
		ACME	0.977	0.941	0.918	0.93	0.859
		NetMHCstabpan-1.0	0.973	0.94	0.907	0.924	0.847
		NetMHCpan-4.1	0.972	0.936	0.904	0.92	0.842
		NetMHCcons-1.1	0.972	0.936	0.909	0.922	0.845
		HLAB	0.9716	0.9599	0.9123	0.9361	0.8732
		Anthem	0.97	0.946	0.88	0.913	0.828
		DeepSeqPan	0.965	0.919	0.899	0.909	0.818
		MHCNetSeq	0.949	0.895	0.885	0.89	0.78
		MixMHCpred-2.0.2	0.948	0.888	0.864	0.876	0.752
	HLA-A*02:04	CapsNet-MHC	0.9881	0.9493	0.9386	0.9439	0.8879
		MixMHCpred-2.0.2	0.986	0.937	0.949	0.943	0.886
		NetMHCpan-4.1	0.986	0.955	0.955	0.955	0.91

	MHCNetSeq	0.986	0.95	0.955	0.952	0.904
	NetMHCcons-1.1	0.983	0.958	0.952	0.955	0.91
	NetMHCstabpan-1.0	0.983	0.959	0.946	0.953	0.906
	ACME	0.982	0.946	0.948	0.947	0.894
	Anthem	0.98	0.917	0.962	0.939	0.88
	HLAB	0.976	0.9457	0.9458	0.9458	0.8915
	DeepSeqPan	0.975	0.94	0.935	0.938	0.875
HLA-B*07:02	CapsNet-MHC	0.9888	0.9576	0.9532	0.9554	0.9108
	NetMHCpan-4.1	0.986	0.956	0.956	0.956	0.912
	NetMHCcons-1.1	0.986	0.956	0.956	0.956	0.911
	NetMHCstabpan-1.0	0.986	0.955	0.959	0.957	0.914
	HLAB	0.9847	0.9586	0.9396	0.9491	0.8983
	ACME	0.984	0.949	0.958	0.954	0.907
	Anthem	0.983	0.942	0.954	0.948	0.896
	MHCNetSeq	0.979	0.939	0.942	0.941	0.881
	DeepSeqPan	0.979	0.938	0.935	0.936	0.873
	MixMHCpred-2.0.2	0.978	0.937	0.939	0.938	0.876
HLA-B*08:01	CapsNet-MHC	0.9881	0.947	0.9489	0.9479	0.8959
	NetMHCpan-4.1	0.987	0.956	0.956	0.955	0.911
	NetMHCcons-1.1	0.985	0.953	0.952	0.953	0.905
	NetMHCstabpan-1.0	0.985	0.953	0.95	0.952	0.904
	HLAB	0.9848	0.9394	0.9603	0.9498	0.8999
	ACME	0.983	0.947	0.95	0.948	0.897
	Anthem	0.98	0.93	0.942	0.936	0.871
	MixMHCpred-2.0.2	0.974	0.923	0.927	0.925	0.85
	MHCNetSeq	0.974	0.921	0.944	0.933	0.866
	DeepSeqPan	0.97	0.924	0.915	0.92	0.84
HLA-B*13:02	CapsNet-MHC	0.9965	0.9851	0.9735	0.9793	0.9586
	Anthem	0.996	0.982	0.978	0.98	0.96
	HLAB	0.9957	0.9751	0.9818	0.9785	0.9569
	MixMHCpred-2.0.2	0.995	0.977	0.976	0.976	0.953
	NetMHCpan-4.1	0.991	0.964	0.955	0.96	0.92
	NetMHCstabpan-1.0	0.968	0.931	0.894	0.912	0.826
	NetMHCcons-1.1	0.967	0.932	0.892	0.912	0.825
	MHCNetSeq	0.93	0.883	0.851	0.867	0.735
	ACME	0.859	0.774	0.775	0.775	0.549
HLA-B*14:01	CapsNet-MHC	1	1	1	1	1
	HLAB	1	1	1	1	1
	MixMHCpred-2.0.2	1	1	1	1	1
	Anthem	0.997	1	0.984	0.992	0.985
	NetMHCstabpan-1.0	0.992	0.958	0.963	0.961	0.922
	NetMHCcons-1.1	0.991	0.963	0.963	0.963	0.927
	NetMHCpan-4.1	0.99	0.942	0.979	0.961	0.922

	ACME	0.989	0.953	0.968	0.961	0.923
	MHCNetSeq	0.982	0.937	0.968	0.953	0.907
	DeepSeqPan	0.981	0.947	0.963	0.955	0.912
HLA-B*14:02	CapsNet-MHC	0.9914	0.974	0.9497	0.9619	0.924
	Anthem	0.987	0.965	0.935	0.95	0.9
	HLAB	0.9858	0.9832	0.9314	0.9573	0.9158
	NetMHCpan-4.1	0.979	0.976	0.928	0.952	0.906
	NetMHCstabpan-1.0	0.978	0.96	0.934	0.947	0.894
	NetMHCcons-1.1	0.97	0.963	0.908	0.935	0.873
	MixMHCpred-2.0.2	0.952	0.902	0.86	0.881	0.763
	ACME	0.936	0.868	0.859	0.864	0.728
	DeepSeqPan	0.909	0.857	0.815	0.836	0.672
	MHCNetSeq	0.887	0.787	0.798	0.793	0.586
HLA-B*15:01	CapsNet-MHC	0.9876	0.9552	0.9485	0.9518	0.9036
	Anthem	0.985	0.95	0.94	0.945	0.89
	NetMHCstabpan-1.0	0.983	0.947	0.95	0.949	0.897
	NetMHCpan-4.1	0.982	0.951	0.942	0.946	0.893
	MixMHCpred-2.0.2	0.981	0.942	0.934	0.938	0.876
	NetMHCcons-1.1	0.981	0.943	0.946	0.944	0.889
	HLAB	0.9805	0.9971	0.4579	0.7274	0.5402
	ACME	0.98	0.948	0.943	0.945	0.891
	MHCNetSeq	0.973	0.938	0.936	0.937	0.874
	DeepSeqPan	0.966	0.922	0.905	0.914	0.827
HLA-B*15:02	CapsNet-MHC	0.979	0.9623	0.9444	0.9533	0.9067
	Anthem	0.979	0.954	0.956	0.955	0.911
	HLAB	0.9766	0.9811	0.9444	0.9626	0.9259
	MixMHCpred-2.0.2	0.975	0.941	0.955	0.948	0.897
	NetMHCcons-1.1	0.97	0.962	0.973	0.968	0.936
	MHCNetSeq	0.97	0.951	0.902	0.926	0.855
	NetMHCpan-4.1	0.969	0.958	0.97	0.964	0.929
	NetMHCstabpan-1.0	0.969	0.96	0.96	0.96	0.921
	ACME	0.969	0.956	0.953	0.955	0.91
	DeepSeqPan	0.926	0.887	0.874	0.88	0.761
HLA-B*15:03	CapsNet-MHC	0.9821	0.9878	0.8675	0.9273	0.861
	NetMHCcons-1.1	0.979	0.934	0.934	0.934	0.869
	ACME	0.979	0.965	0.935	0.95	0.901
	NetMHCpan-4.1	0.974	0.918	0.934	0.926	0.853
	NetMHCstabpan-1.0	0.974	0.919	0.929	0.924	0.849
	Anthem	0.973	0.902	0.915	0.909	0.819
	HLAB	0.9691	0.8049	0.9518	0.8788	0.7655
	DeepSeqPan	0.969	0.941	0.943	0.942	0.884
	MixMHCpred-2.0.2	0.919	0.896	0.828	0.862	0.727
	MHCNetSeq	0.797	0.7	0.777	0.738	0.483

HLA-B*15:09	CapsNet-MHC	0.9978	0.7619	1	0.8837	0.7879
	ACME	0.996	0.99	0.981	0.986	0.972
	MixMHCpred-2.0.2	0.994	1	0.976	0.988	0.977
	NetMHCpan-4.1	0.994	1	0.976	0.988	0.976
	NetMHCcons-1.1	0.992	1	0.971	0.986	0.972
	HLAB	0.9913	0.8571	1	0.9302	0.8685
	NetMHCstabpan-1.0	0.99	0.995	0.971	0.983	0.967
	DeepSeqPan	0.978	0.962	0.924	0.943	0.887
	Anthem	0.974	0.743	0.965	0.854	0.728
	MHCNetSeq	0.927	0.867	0.862	0.864	0.73
HLA-B*15:11	Anthem	0.989	0.966	0.978	0.972	0.943
	MHCNetSeq	0.98	0.942	0.926	0.934	0.868
	CapsNet-MHC	0.9773	0.9514	0.914	0.9326	0.8659
	NetMHCpan-4.1	0.97	0.933	0.922	0.927	0.855
	ACME	0.969	0.939	0.927	0.933	0.865
	HLAB	0.9609	0.8973	0.9409	0.9191	0.839
	NetMHCstabpan-1.0	0.965	0.923	0.922	0.923	0.846
	NetMHCcons-1.1	0.962	0.918	0.91	0.914	0.828
HLA-B*15:17	MixMHCpred-2.0.2	0.969	0.94	0.902	0.921	0.844
	Anthem	0.957	0.913	0.877	0.895	0.792
	ACME	0.953	0.907	0.885	0.896	0.793
	NetMHCpan-4.1	0.948	0.913	0.88	0.896	0.794
	NetMHCcons-1.1	0.944	0.898	0.881	0.889	0.78
	NetMHCstabpan-1.0	0.944	0.903	0.883	0.893	0.787
	CapsNet-MHC	0.9405	0.9091	0.85	0.8794	0.7602
	DeepSeqPan	0.908	0.842	0.847	0.845	0.691
	HLAB	0.9037	0.9192	0.82	0.8693	0.7426
	MHCNetSeq	0.827	0.738	0.833	0.786	0.575
HLA-B*15:18	CapsNet-MHC	0.9979	0.9954	0.987	0.988	0.976
	HLAB	0.9955	0.9954	0.9862	0.9908	0.9816
	Anthem	0.995	0.989	0.9862	0.9908	0.9816
	MixMHCpred-2.0.2	0.995	0.994	0.993	0.994	0.988
	NetMHCpan-4.1	0.991	0.981	0.96	0.971	0.942
	NetMHCcons-1.1	0.986	0.972	0.961	0.967	0.933
	NetMHCstabpan-1.0	0.986	0.971	0.964	0.967	0.935
	ACME	0.981	0.969	0.952	0.96	0.921
	MHCNetSeq	0.789	0.702	0.723	0.712	0.425
HLA-B*15:42	MixMHCpred-2.0.2	0.983	0.931	0.948	0.94	0.879
	CapsNet-MHC	0.9816	0.9437	0.8472	0.8951	0.7942
	Anthem	0.972	0.921	0.903	0.912	0.827
	HLAB	0.9475	0.9155	0.9167	0.9161	0.8322
	NetMHCstabpan-1.0	0.941	0.884	0.887	0.886	0.772
	NetMHCpan-4.1	0.93	0.876	0.882	0.879	0.758

	NetMHCcons-1.1	0.879	0.856	0.773	0.815	0.633
	DeepSeqPan	0.865	0.797	0.792	0.795	0.59
	ACME	0.805	0.834	0.711	0.773	0.55
HLA-B*18:01	ACME	0.99	0.953	0.965	0.959	0.918
	CapsNet-MHC	0.989	0.9615	0.9472	0.9544	0.9089
	NetMHCpan-4.1	0.988	0.953	0.954	0.953	0.906
	NetMHCcons-1.1	0.988	0.956	0.956	0.956	0.911
	NetMHCstabpan-1.0	0.987	0.951	0.952	0.952	0.904
	Anthem	0.985	0.93	0.955	0.943	0.886
	MixMHCpred-2.0.2	0.978	0.924	0.928	0.926	0.852
	HLAB	0.9772	0.9591	0.952	0.9556	0.9112
	DeepSeqPan	0.972	0.92	0.926	0.923	0.846
	MHCNetSeq	0.971	0.918	0.932	0.925	0.851
HLA-B*18:03	Anthem	0.999	0.988	0.982	0.985	0.97
	MixMHCpred-2.0.2	0.999	1	0.991	0.995	0.991
	NetMHCpan-4.1	0.99	0.96	0.981	0.97	0.941
	ACME	0.987	0.947	0.947	0.947	0.895
	NetMHCcons-1.1	0.984	0.969	0.95	0.96	0.92
	NetMHCstabpan-1.0	0.983	0.963	0.938	0.95	0.901
	MHCNetSeq	0.978	0.938	0.956	0.947	0.894
	CapsNet-MHC	0.965	0.8438	0.9091	0.8769	0.755
	HLAB	0.9337	1	0.8485	0.9231	0.8566
HLA-B*27:01	MixMHCpred-2.0.2	0.997	0.991	0.983	0.988	0.975
	NetMHCpan-4.1	0.997	0.993	0.981	0.987	0.975
	Anthem	0.996	0.993	0.976	0.984	0.969
	ACME	0.995	0.986	0.971	0.979	0.958
	MHCNetSeq	0.995	0.984	0.969	0.977	0.953
	CapsNet-MHC	0.9945	0.9974	0.9683	0.9828	0.9661
	NetMHCcons-1.1	0.989	0.966	0.943	0.955	0.91
	NetMHCstabpan-1.0	0.989	0.961	0.944	0.953	0.906
	HLAB	0.9886	0.9894	0.9683	0.9789	0.9579
	DeepSeqPan	0.878	0.826	0.79	0.808	0.616
HLA-B*27:02	CapsNet-MHC	0.9976	0.9883	0.9922	0.9903	0.9806
	MixMHCpred-2.0.2	0.996	0.982	0.984	0.983	0.967
	HLAB	0.9952	0.9922	0.9882	0.9903	0.9806
	Anthem	0.994	0.983	0.984	0.983	0.967
	NetMHCpan-4.1	0.993	0.981	0.978	0.98	0.959
	NetMHCstabpan-1.0	0.991	0.985	0.971	0.978	0.956
	MHCNetSeq	0.991	0.97	0.971	0.971	0.941
	ACME	0.989	0.978	0.965	0.972	0.943
	NetMHCcons-1.1	0.988	0.968	0.959	0.963	0.927
	DeepSeqPan	0.969	0.939	0.91	0.924	0.85
HLA-B*27:03	MHCNetSeq	0.996	0.981	0.974	0.978	0.956

	CapsNet-MHC	0.993	0.9608	0.9903	0.9756	0.9516
	HLAB	0.9882	0.9804	0.9806	0.9805	0.961
	MixMHCpred-2.0.2	0.988	0.937	0.974	0.956	0.912
	NetMHCpan-4.1	0.986	0.952	0.96	0.956	0.913
	Anthem	0.985	0.924	0.979	0.952	0.905
	ACME	0.972	0.921	0.97	0.945	0.891
	NetMHCcons-1.1	0.968	0.931	0.981	0.956	0.914
	NetMHCstabpan-1.0	0.968	0.931	0.982	0.957	0.915
	DeepSeqPan	0.951	0.881	0.911	0.896	0.793
HLA-B*27:04	CapsNet-MHC	0.9958	0.9758	0.9783	0.9771	0.9542
	MixMHCpred-2.0.2	0.995	0.976	0.965	0.971	0.941
	Anthem	0.993	0.973	0.969	0.971	0.943
	HLAB	0.9928	0.9807	0.9735	0.9771	0.9542
	NetMHCpan-4.1	0.99	0.963	0.96	0.961	0.923
	NetMHCstabpan-1.0	0.989	0.965	0.951	0.958	0.917
	NetMHCcons-1.1	0.988	0.957	0.951	0.954	0.909
	ACME	0.987	0.948	0.955	0.951	0.903
	MHCNetSeq	0.977	0.939	0.946	0.943	0.885
	DeepSeqPan	0.973	0.925	0.947	0.936	0.872
HLA-B*27:05	CapsNet-MHC	0.9367	0.8485	0.8815	0.865	0.7303
	HLAB	0.9365	0.8273	0.9167	0.872	0.747
	Anthem	0.913	0.81	0.856	0.833	0.667
	MixMHCpred-2.0.2	0.878	0.783	0.792	0.788	0.576
	NetMHCpan-4.1	0.862	0.748	0.795	0.772	0.544
	ACME	0.861	0.741	0.808	0.774	0.549
	NetMHCcons-1.1	0.857	0.729	0.806	0.767	0.536
	NetMHCstabpan-1.0	0.857	0.728	0.809	0.768	0.538
	DeepSeqPan	0.83	0.706	0.801	0.753	0.509
	MHCNetSeq	0.81	0.706	0.809	0.757	0.517
HLA-B*27:06	CapsNet-MHC	0.9947	0.9691	0.9755	0.9723	0.9446
	Anthem	0.992	0.953	0.968	0.961	0.922
	MixMHCpred-2.0.2	0.992	0.949	0.97	0.96	0.92
	MHCNetSeq	0.989	0.954	0.948	0.951	0.903
	HLAB	0.9833	0.9691	0.9387	0.9538	0.9081
	NetMHCpan-4.1	0.981	0.922	0.973	0.947	0.896
	ACME	0.981	0.945	0.956	0.951	0.901
	NetMHCstabpan-1.0	0.978	0.94	0.946	0.943	0.887
	NetMHCcons-1.1	0.976	0.937	0.943	0.94	0.88
	DeepSeqPan	0.965	0.927	0.933	0.93	0.86
HLA-B*27:07	MixMHCpred-2.0.2	0.999	0.989	0.989	0.989	0.979
	NetMHCpan-4.1	0.997	0.996	0.986	0.991	0.982
	CapsNet-MHC	0.9964	1	0.9865	0.9932	0.9865
	Anthem	0.995	0.991	0.982	0.987	0.973

	ACME	0.995	0.984	0.976	0.98	0.96
	HLAB	0.9944	0.9864	0.9865	0.9865	0.9729
	MHCNetSeq	0.994	0.987	0.969	0.978	0.956
	NetMHCstabpan-1.0	0.992	0.967	0.968	0.968	0.935
	NetMHCcons-1.1	0.991	0.97	0.956	0.963	0.926
HLA-B*27:08	CapsNet-MHC	0.9989	0.9947	0.9947	0.9947	0.9894
	HLAB	0.9977	0.9947	0.9947	0.9947	0.9894
	Anthem	0.995	0.989	0.98	0.985	0.97
	MixMHCpred-2.0.2	0.995	0.988	0.985	0.986	0.972
	NetMHCpan-4.1	0.992	0.989	0.972	0.98	0.961
	NetMHCstabpan-1.0	0.99	0.981	0.965	0.973	0.947
	NetMHCcons-1.1	0.989	0.992	0.957	0.975	0.95
	ACME	0.988	0.97	0.963	0.966	0.933
HLA-B*27:09	HLAB	0.9853	0.9066	0.9705	0.9386	0.8789
	CapsNet-MHC	0.9849	0.923	0.9476	0.9353	0.8709
	Anthem	0.97	0.894	0.927	0.91	0.822
	NetMHCpan-4.1	0.953	0.877	0.898	0.888	0.776
	MixMHCpred-2.0.2	0.947	0.872	0.874	0.873	0.746
	NetMHCcons-1.1	0.912	0.776	0.907	0.841	0.689
	NetMHCstabpan-1.0	0.912	0.772	0.913	0.843	0.693
	ACME	0.91	0.765	0.901	0.833	0.673
	MHCNetSeq	0.899	0.8	0.858	0.829	0.66
HLA-B*27:20	MixMHCpred-2.0.2	0.999	1	0.995	0.997	0.995
	NetMHCcons-1.1	0.995	0.995	0.979	0.987	0.975
	NetMHCstabpan-1.0	0.995	0.995	0.979	0.987	0.974
	NetMHCpan-4.1	0.994	0.984	0.979	0.982	0.964
	CapsNet-MHC	0.9921	0.8421	0.95	0.8974	0.7984
	DeepSeqPan	0.98	0.974	0.926	0.95	0.903
	ACME	0.978	0.958	0.931	0.945	0.891
	Anthem	0.977	0.884	0.98	0.932	0.871
	HLAB	0.9763	0.8947	0.95	0.9231	0.847
	MHCNetSeq	0.867	0.805	0.821	0.813	0.631
HLA-B*35:01	CapsNet-MHC	0.9839	0.9479	0.94	0.944	0.8879
	NetMHCpan-4.1	0.978	0.948	0.941	0.944	0.889
	HLAB	0.97	0.9387	0.9424	0.9406	0.8812
	NetMHCstabpan-1.0	0.977	0.947	0.937	0.942	0.884
	Anthem	0.976	0.936	0.937	0.937	0.873
	NetMHCcons-1.1	0.976	0.941	0.937	0.939	0.878
	ACME	0.975	0.945	0.931	0.938	0.877
	MixMHCpred-2.0.2	0.962	0.914	0.894	0.904	0.808
	DeepSeqPan	0.943	0.888	0.866	0.877	0.753
	MHCNetSeq	0.942	0.859	0.894	0.876	0.753
HLA-B*35:03	CapsNet-MHC	0.997	0.9836	0.97	0.9768	0.9537

	Anthem	0.995	0.972	0.964	0.968	0.936
	HLAB	0.9947	0.9823	0.9755	0.9789	0.9578
	MixMHCpred-2.0.2	0.994	0.96	0.968	0.964	0.928
	NetMHCpan-4.1	0.994	0.975	0.962	0.969	0.937
	NetMHCstabpan-1.0	0.993	0.972	0.96	0.966	0.932
	NetMHCcons-1.1	0.992	0.967	0.959	0.963	0.926
	ACME	0.991	0.967	0.957	0.962	0.924
	DeepSeqPan	0.981	0.927	0.944	0.936	0.872
	MHCNetSeq	0.973	0.931	0.92	0.925	0.851
HLA-B*35:08	CapsNet-MHC	0.9853	0.9433	0.9538	0.9486	0.8972
	Anthem	0.983	0.92	0.955	0.937	0.876
	NetMHCpan-4.1	0.981	0.964	0.952	0.958	0.917
	HLAB	0.9802	0.9124	0.959	0.9357	0.8724
	ACME	0.978	0.958	0.943	0.95	0.901
	NetMHCcons-1.1	0.977	0.952	0.951	0.951	0.903
	NetMHCstabpan-1.0	0.977	0.951	0.947	0.949	0.899
	MixMHCpred-2.0.2	0.963	0.882	0.911	0.897	0.795
	MHCNetSeq	0.958	0.883	0.906	0.894	0.789
	DeepSeqPan	0.944	0.883	0.88	0.882	0.764
HLA-B*37:01	CapsNet-MHC	0.9964	0.984	0.9753	0.9797	0.9594
	Anthem	0.995	0.977	0.978	0.978	0.955
	HLAB	0.9911	0.9753	0.9739	0.9746	0.9492
	NetMHCpan-4.1	0.991	0.991	0.968	0.979	0.959
	NetMHCstabpan-1.0	0.991	0.97	0.961	0.966	0.932
	NetMHCcons-1.1	0.99	0.964	0.957	0.961	0.922
	ACME	0.988	0.96	0.957	0.959	0.918
	MHCNetSeq	0.963	0.91	0.916	0.913	0.826
	DeepSeqPan	0.961	0.925	0.926	0.925	0.85
	MixMHCpred-2.0.2	0.953	0.922	0.873	0.897	0.796
HLA-B*38:01	Anthem	0.998	0.992	0.988	0.99	0.98
	MixMHCpred-2.0.2	0.998	0.988	0.989	0.989	0.978
	CapsNet-MHC	0.9971	0.991	0.9821	0.9865	0.9731
	NetMHCpan-4.1	0.997	0.987	0.981	0.985	0.969
	NetMHCcons-1.1	0.996	0.983	0.972	0.978	0.955
	ACME	0.995	0.98	0.972	0.976	0.952
	HLAB	0.9944	0.9859	0.9859	0.9859	0.9718
	NetMHCstabpan-1.0	0.993	0.972	0.966	0.969	0.938
	MHCNetSeq	0.986	0.953	0.943	0.948	0.896
	DeepSeqPan	0.979	0.936	0.922	0.929	0.859
HLA-B*39:01	CapsNet-MHC	0.9949	0.9672	0.9717	0.9694	0.9389
	NetMHCpan-4.1	0.989	0.963	0.951	0.957	0.915
	Anthem	0.988	0.944	0.954	0.949	0.898
	HLAB	0.9862	0.9717	0.9717	0.9717	0.915

	ACME	0.985	0.96	0.943	0.952	0.9434
	NetMHCcons-1.1	0.983	0.942	0.934	0.938	0.876
	NetMHCstabpan-1.0	0.983	0.945	0.934	0.94	0.879
	MixMHCpred-2.0.2	0.981	0.932	0.938	0.935	0.87
	MHCNetSeq	0.976	0.929	0.933	0.931	0.862
	DeepSeqPan	0.946	0.889	0.862	0.875	0.751
HLA-B*39:06	CapsNet-MHC	0.9974	0.9905	0.9779	0.9842	0.9685
	HLAB	0.997	0.9937	0.9716	0.9826	0.9655
	Anthem	0.996	0.984	0.984	0.984	0.968
	MixMHCpred-2.0.2	0.996	0.981	0.981	0.981	0.962
	NetMHCpan-4.1	0.993	0.968	0.965	0.966	0.933
	ACME	0.97	0.936	0.916	0.926	0.852
	NetMHCstabpan-1.0	0.96	0.913	0.896	0.905	0.81
	NetMHCcons-1.1	0.958	0.91	0.894	0.902	0.804
	MHCNetSeq	0.939	0.88	0.871	0.875	0.751
HLA-B*39:24	CapsNet-MHC	0.9925	0.9794	0.9184	0.9487	0.8992
	MixMHCpred-2.0.2	0.989	0.958	0.975	0.967	0.933
	MHCNetSeq	0.989	0.966	0.958	0.962	0.924
	Anthem	0.988	0.959	0.975	0.967	0.934
	NetMHCpan-4.1	0.988	0.978	0.961	0.97	0.94
	ACME	0.984	0.968	0.944	0.956	0.913
	NetMHCstabpan-1.0	0.981	0.975	0.922	0.948	0.898
	NetMHCcons-1.1	0.978	0.964	0.92	0.942	0.885
	HLAB	0.977	0.9588	0.9286	0.9436	0.8876
HLA-C*01:02	CapsNet-MHC	0.9975	0.9885	0.9696	0.979	0.9583
	NetMHCpan-4.1	0.996	0.98	0.971	0.979	0.9581
	HLAB	0.9923	0.9771	0.981	0.976	0.952
	NetMHCcons-1.1	0.99	0.968	0.955	0.962	0.924
	Anthem	0.989	0.937	0.961	0.949	0.898
	MixMHCpred-2.0.2	0.989	0.947	0.947	0.947	0.894
	NetMHCstabpan-1.0	0.987	0.95	0.941	0.945	0.891
	MHCNetSeq	0.873	0.789	0.799	0.794	0.589
HLA-C*02:02	CapsNet-MHC	0.9872	0.9748	0.9313	0.953	0.9069
	Anthem	0.98	0.959	0.913	0.936	0.873
	HLAB	0.9728	0.968	0.9361	0.9521	0.9046
	NetMHCpan-4.1	0.971	0.953	0.911	0.932	0.865
	NetMHCstabpan-1.0	0.97	0.955	0.915	0.935	0.871
	NetMHCcons-1.1	0.964	0.948	0.896	0.922	0.845
	MixMHCpred-2.0.2	0.95	0.901	0.862	0.881	0.764
	MHCNetSeq	0.855	0.781	0.807	0.794	0.588
HLA-C*03:03	Anthem	0.985	0.961	0.94	0.95	0.901
	CapsNet-MHC	0.9842	0.962	0.9399	0.951	0.9022
	NetMHCpan-4.1	0.97	0.94	0.921	0.93	0.861

	NetMHCstabpan-1.0	0.97	0.937	0.917	0.927	0.855
	MixMHCpred-2.0.2	0.967	0.916	0.906	0.911	0.822
	NetMHCcons-1.1	0.964	0.932	0.9	0.916	0.832
	HLAB	0.9512	0.9979	0.7703	0.884	0.7888
	DeepSeqPan	0.904	0.821	0.833	0.827	0.655
	MHCNetSeq	0.867	0.804	0.778	0.79	0.582
HLA-C*03:04	CapsNet-MHC	0.9931	0.987	0.9479	0.9674	0.9356
	Anthem	0.988	0.975	0.937	0.956	0.913
	NetMHCpan-4.1	0.978	0.968	0.934	0.951	0.902
	NetMHCcons-1.1	0.978	0.974	0.928	0.951	0.904
	NetMHCstabpan-1.0	0.978	0.969	0.93	0.95	0.9
	MixMHCpred-2.0.2	0.973	0.925	0.914	0.919	0.839
	HLAB	0.9707	0.9467	0.9657	0.9562	0.9125
	MHCNetSeq	0.854	0.781	0.765	0.773	0.546
HLA-C*04:01	CapsNet-MHC	0.9761	0.9315	0.9414	0.9365	0.873
	HLAB	0.9701	0.9167	0.947	0.9319	0.8641
	Anthem	0.962	0.909	0.914	0.912	0.823
	NetMHCpan-4.1	0.956	0.913	0.909	0.911	0.822
	NetMHCstabpan-1.0	0.952	0.912	0.899	0.905	0.81
	NetMHCcons-1.1	0.942	0.894	0.871	0.882	0.765
	MixMHCpred-2.0.2	0.919	0.857	0.839	0.848	0.696
	DeepSeqPan	0.843	0.782	0.758	0.77	0.541
	MHCNetSeq	0.553	0.46	0.75	0.605	0.219
HLA-C*05:01	CapsNet-MHC	0.9928	0.9714	0.9578	0.9646	0.9292
	Anthem	0.991	0.952	0.957	0.955	0.91
	NetMHCpan-4.1	0.99	0.958	0.95	0.954	0.908
	NetMHCstabpan-1.0	0.987	0.962	0.944	0.953	0.906
	MixMHCpred-2.0.2	0.986	0.942	0.93	0.936	0.872
	NetMHCcons-1.1	0.986	0.959	0.938	0.949	0.898
	HLAB	0.9746	0.9768	0.9496	0.9632	0.9267
	DeepSeqPan	0.953	0.892	0.878	0.885	0.77
	MHCNetSeq	0.918	0.847	0.858	0.853	0.705
HLA-C*06:02	CapsNet-MHC	0.9829	0.9738	0.9223	0.948	0.8973
	Anthem	0.981	0.949	0.927	0.938	0.876
	HLAB	0.9772	0.9611	0.9322	0.9467	0.8937
	NetMHCpan-4.1	0.976	0.951	0.93	0.94	0.881
	NetMHCstabpan-1.0	0.972	0.938	0.923	0.93	0.861
	NetMHCcons-1.1	0.967	0.934	0.906	0.92	0.841
	MixMHCpred-2.0.2	0.956	0.881	0.893	0.887	0.774
	DeepSeqPan	0.864	0.786	0.776	0.781	0.563
	MHCNetSeq	0.771	0.684	0.78	0.732	0.466
HLA-C*07:01	CapsNet-MHC	0.9847	0.9564	0.9537	0.955	0.9101
	Anthem	0.975	0.933	0.924	0.929	0.857

	HLAB	0.9708	0.9606	0.9368	0.9487	0.8977
	NetMHCpan-4.1	0.97	0.956	0.925	0.94	0.881
	NetMHCstabpan-1.0	0.966	0.943	0.911	0.927	0.855
	NetMHCcons-1.1	0.957	0.919	0.892	0.906	0.812
	MixMHCpred-2.0.2	0.914	0.855	0.82	0.837	0.676
	DeepSeqPan	0.855	0.775	0.792	0.784	0.568
	MHCNetSeq	0.847	0.764	0.762	0.763	0.526
HLA-C*07:02	CapsNet-MHC	0.9857	0.9695	0.9197	0.9446	0.8903
	Anthem	0.982	0.953	0.921	0.937	0.875
	NetMHCpan-4.1	0.974	0.937	0.916	0.926	0.853
	NetMHCstabpan-1.0	0.97	0.915	0.919	0.917	0.834
	NetMHCcons-1.1	0.967	0.917	0.901	0.909	0.818
	MixMHCpred-2.0.2	0.954	0.891	0.886	0.888	0.777
	HLAB	0.9493	0.8328	0.9358	0.8843	0.7728
	DeepSeqPan	0.839	0.745	0.773	0.759	0.519
	MHCNetSeq	0.802	0.721	0.81	0.765	0.532
HLA-C*07:04	CapsNet-MHC	0.9887	0.9615	0.9524	0.9569	0.9139
	Anthem	0.98	0.936	0.929	0.933	0.866
	HLAB	0.9781	0.9615	0.8352	0.8983	0.803
	NetMHCpan-4.1	0.973	0.96	0.91	0.935	0.872
	NetMHCstabpan-1.0	0.962	0.947	0.895	0.921	0.844
	NetMHCcons-1.1	0.904	0.85	0.817	0.833	0.667
	MHCNetSeq	0.651	0.578	0.664	0.621	0.243
HLA-C*08:02	CapsNet-MHC	0.9916	0.9796	0.9542	0.9669	0.9341
	Anthem	0.987	0.967	0.937	0.952	0.905
	HLAB	0.9799	0.9593	0.9613	0.9603	0.9206
	NetMHCpan-4.1	0.977	0.969	0.924	0.946	0.894
	NetMHCstabpan-1.0	0.977	0.957	0.921	0.939	0.878
	NetMHCcons-1.1	0.964	0.934	0.874	0.904	0.809
	MixMHCpred-2.0.2	0.944	0.883	0.857	0.87	0.741
	DeepSeqPan	0.927	0.876	0.825	0.851	0.703
	MHCNetSeq	0.801	0.739	0.74	0.74	0.48
HLA-C*12:03	CapsNet-MHC	0.9913	0.976	0.9468	0.9614	0.9232
	Anthem	0.989	0.96	0.948	0.954	0.908
	HLAB	0.9845	0.9867	0.9069	0.9467	0.8964
	NetMHCcons-1.1	0.981	0.941	0.926	0.933	0.867
	NetMHCpan-4.1	0.98	0.935	0.921	0.928	0.856
	MixMHCpred-2.0.2	0.976	0.926	0.915	0.921	0.841
	NetMHCstabpan-1.0	0.972	0.93	0.91	0.92	0.841
	DeepSeqPan	0.917	0.872	0.844	0.858	0.716
	MHCNetSeq	0.798	0.719	0.756	0.738	0.476
HLA-C*14:02	NetMHCpan-4.1	0.996	0.984	0.979	0.981	0.962
	CapsNet-MHC	0.995	0.9891	0.9673	0.9782	0.9566

		Anthem	0.993	0.968	0.967	0.968	0.935
		NetMHCstabpan-1.0	0.993	0.973	0.96	0.967	0.934
		MixMHCpred-2.0.2	0.992	0.952	0.965	0.958	0.917
		NetMHCcons-1.1	0.992	0.974	0.962	0.968	0.936
		HLAB	0.9918	0.9836	0.9619	0.9727	0.9457
		DeepSeqPan	0.939	0.883	0.851	0.867	0.735
		MHCNetSeq	0.528	0.502	0.501	0.502	0.003
	HLA-C*15:02	CapsNet-MHC	0.9902	0.9806	0.9503	0.9654	0.9313
		MixMHCpred-2.0.2	0.989	0.956	0.96	0.958	0.917
		Anthem	0.988	0.959	0.956	0.958	0.915
		NetMHCpan-4.1	0.988	0.964	0.954	0.959	0.918
		NetMHCcons-1.1	0.984	0.973	0.931	0.952	0.905
		NetMHCstabpan-1.0	0.98	0.946	0.935	0.94	0.881
		HLAB	0.9769	0.964	0.9392	0.9516	0.9035
		DeepSeqPan	0.948	0.883	0.895	0.889	0.778
		MHCNetSeq	0.926	0.832	0.871	0.852	0.705
	HLA-C*16:01	CapsNet-MHC	0.9907	0.9847	0.9266	0.9556	0.9128
		Anthem	0.989	0.972	0.933	0.953	0.907
		HLAB	0.9831	0.9449	0.9572	0.951	0.9021
		NetMHCpan-4.1	0.98	0.961	0.914	0.938	0.876
		NetMHCstabpan-1.0	0.967	0.934	0.91	0.922	0.844
		NetMHCcons-1.1	0.966	0.935	0.906	0.92	0.841
		MixMHCpred-2.0.2	0.96	0.872	0.903	0.888	0.776
		MHCNetSeq	0.689	0.628	0.682	0.655	0.311
	HLA-C*17:01	CapsNet-MHC	0.9849	0.9286	0.9529	0.9408	0.8819
		Anthem	0.983	0.905	0.958	0.931	0.864
		NetMHCpan-4.1	0.979	0.919	0.941	0.93	0.861
		NetMHCstabpan-1.0	0.969	0.92	0.919	0.92	0.839
		MixMHCpred-2.0.2	0.962	0.905	0.948	0.927	0.854
		HLAB	0.9605	0.9167	0.9176	0.9172	0.8343
		NetMHCcons-1.1	0.938	0.864	0.924	0.894	0.79
		MHCNetSeq	0.855	0.831	0.775	0.803	0.607
10	HLA-A*01:01	CapsNet-MHC	0.9924	0.9575	0.9621	0.9598	0.9196
		NetMHCpan-4.1	0.988	0.957	0.957	0.957	0.914
		Anthem	0.986	0.958	0.951	0.954	0.909
		NetMHCcons-1.1	0.981	0.919	0.932	0.926	0.851
		MixMHCpred-2.0.2	0.98	0.937	0.937	0.937	0.874
		HLAB	0.9779	0.9843	0.9487	0.9665	0.9336
		NetMHCstabpan-1.0	0.976	0.907	0.932	0.92	0.84
		ACME	0.968	0.905	0.908	0.906	0.813
		MHCNetSeq	0.942	0.868	0.876	0.872	0.744
	HLA-A*02:01	CapsNet-MHC	0.9584	0.9178	0.8925	0.9051	0.8106
		HLAB	0.9573	0.9754	0.7075	0.8414	0.7088

	Anthem	0.948	0.888	0.885	0.886	0.773
	NetMHCcons-1.1	0.943	0.868	0.887	0.878	0.756
	NetMHCstabpan-1.0	0.942	0.874	0.876	0.875	0.75
	NetMHCpan-4.1	0.933	0.868	0.877	0.872	0.744
	ACME	0.931	0.864	0.888	0.876	0.753
	MixMHCpred-2.0.2	0.919	0.845	0.848	0.846	0.693
	MHCNetSeq	0.9	0.819	0.872	0.845	0.691
HLA-A*02:02	CapsNet-MHC	0.9675	0.94	0.9104	0.9252	0.8508
	MixMHCpred-2.0.2	0.965	0.94	0.907	0.923	0.846
	HLAB	0.9572	0.95	0.8856	0.9177	0.8372
	Anthem	0.946	0.959	0.846	0.902	0.811
	NetMHCcons-1.1	0.932	0.867	0.865	0.866	0.732
	NetMHCstabpan-1.0	0.928	0.871	0.864	0.868	0.736
	ACME	0.927	0.893	0.839	0.866	0.734
	NetMHCpan-4.1	0.913	0.855	0.841	0.848	0.697
	MHCNetSeq	0.783	0.696	0.809	0.752	0.508
HLA-A*02:03	HLAB	0.9765	0.95	0.9315	0.9407	0.8816
	CapsNet-MHC	0.9737	0.9469	0.919	0.9392	0.8662
	Anthem	0.964	0.934	0.885	0.91	0.821
	NetMHCcons-1.1	0.959	0.908	0.885	0.896	0.793
	NetMHCstabpan-1.0	0.956	0.903	0.884	0.894	0.788
	ACME	0.955	0.908	0.871	0.89	0.78
	NetMHCpan-4.1	0.945	0.891	0.87	0.88	0.761
	MixMHCpred-2.0.2	0.903	0.792	0.854	0.823	0.648
	MHCNetSeq	0.891	0.818	0.845	0.832	0.664
HLA-A*02:04	HLAB	0.997	0.9355	1	0.9683	0.9383
	CapsNet-MHC	0.995	0.9677	1	0.9841	0.9687
	NetMHCpan-4.1	0.994	0.971	0.984	0.978	0.955
	MixMHCpred-2.0.2	0.992	0.968	0.981	0.974	0.949
	NetMHCcons-1.1	0.99	0.971	0.971	0.971	0.942
	NetMHCstabpan-1.0	0.99	0.968	0.968	0.968	0.936
	ACME	0.988	0.942	0.974	0.958	0.917
	MHCNetSeq	0.988	0.938	0.971	0.955	0.91
	Anthem	0.965	0.819	0.967	0.893	0.797
HLA-A*02:05	NetMHCcons-1.1	0.987	0.982	0.965	0.973	0.947
	NetMHCpan-4.1	0.986	0.973	0.961	0.967	0.935
	NetMHCstabpan-1.0	0.986	0.978	0.96	0.969	0.938
	ACME	0.985	0.969	0.953	0.961	0.923
	HLAB	0.984	0.9733	0.9211	0.947	0.8953
	MHCNetSeq	0.976	0.921	0.92	0.921	0.842
	Anthem	0.975	0.872	0.952	0.912	0.828
	CapsNet-MHC	0.9747	0.9733	0.8684	0.9205	0.8459
	MixMHCpred-2.0.2	0.955	0.878	0.9	0.889	0.78

HLA-A*02:06	HLAB	0.9636	0.9671	0.8879	0.904	0.8575
	CapsNet-MHC	0.9615	0.9437	0.8645	0.9274	0.8106
	Anthem	0.951	0.918	0.87	0.894	0.789
	NetMHCstabpan-1.0	0.949	0.9	0.869	0.885	0.77
	NetMHCcons-1.1	0.948	0.916	0.856	0.886	0.774
	NetMHCpan-4.1	0.937	0.852	0.874	0.863	0.727
	ACME	0.937	0.903	0.847	0.875	0.752
	MHCNetSeq	0.847	0.751	0.843	0.797	0.597
	MixMHCpred-2.0.2	0.784	0.731	0.717	0.724	0.449
HLA-A*02:07	HLAB	0.9713	0.8913	0.9462	0.9189	0.839
	CapsNet-MHC	0.9712	0.9022	0.957	0.9297	0.8607
	MixMHCpred-2.0.2	0.97	0.928	0.944	0.936	0.872
	MHCNetSeq	0.963	0.906	0.934	0.92	0.84
	Anthem	0.956	0.87	0.967	0.918	0.84
	NetMHCpan-4.1	0.956	0.895	0.947	0.921	0.844
	NetMHCcons-1.1	0.942	0.889	0.904	0.897	0.794
	NetMHCstabpan-1.0	0.939	0.897	0.897	0.897	0.794
	ACME	0.896	0.821	0.866	0.843	0.689
HLA-A*02:17	CapsNet-MHC	1	1	0.9355	0.9672	0.9365
	MixMHCpred-2.0.2	0.995	0.997	0.99	0.993	0.987
	NetMHCstabpan-1.0	0.991	0.99	0.967	0.978	0.957
	HLAB	0.9903	0.9	1	0.9508	0.9059
	NetMHCcons-1.1	0.99	0.99	0.97	0.98	0.96
	NetMHCpan-4.1	0.986	0.987	0.967	0.977	0.954
	Anthem	0.985	0.973	0.953	0.963	0.928
	ACME	0.98	0.99	0.933	0.962	0.925
	MHCNetSeq	0.977	1	0.954	0.976	0.955
HLA-A*03:01	HLAB	0.977	0.9577	0.9296	0.9436	0.8876
	NetMHCcons-1.1	0.977	0.946	0.927	0.936	0.872
	CapsNet-MHC	0.9753	0.9597	0.9215	0.9406	0.8818
	NetMHCpan-4.1	0.975	0.934	0.929	0.931	0.863
	NetMHCstabpan-1.0	0.974	0.926	0.928	0.927	0.855
	ACME	0.973	0.934	0.944	0.939	0.878
	Anthem	0.972	0.95	0.921	0.935	0.872
	MixMHCpred-2.0.2	0.948	0.876	0.89	0.883	0.766
	MHCNetSeq	0.914	0.825	0.882	0.854	0.709
HLA-A*11:01	CapsNet-MHC	0.9848	0.9694	0.9202	0.9448	0.8906
	NetMHCcons-1.1	0.983	0.955	0.927	0.941	0.883
	NetMHCpan-4.1	0.982	0.952	0.93	0.941	0.882
	Anthem	0.981	0.953	0.926	0.939	0.879
	NetMHCstabpan-1.0	0.981	0.946	0.93	0.938	0.876
	ACME	0.981	0.956	0.939	0.948	0.896
	HLAB	0.9748	0.9575	0.9423	0.9499	0.8999

	MHCNetSeq	0.948	0.876	0.91	0.893	0.786
	MixMHCpred-2.0.2	0.946	0.869	0.879	0.874	0.749
HLA-A*23:01	CapsNet-MHC	0.9868	0.9322	0.9496	0.9409	0.882
	NetMHCcons-1.1	0.982	0.959	0.935	0.947	0.894
	NetMHCpan-4.1	0.981	0.962	0.952	0.957	0.914
	HLAB	0.9784	0.9322	0.9412	0.9367	0.8734
	NetMHCstabpan-1.0	0.978	0.944	0.929	0.936	0.873
	ACME	0.974	0.958	0.948	0.953	0.906
	Anthem	0.968	0.868	0.957	0.912	0.828
	MixMHCpred-2.0.2	0.96	0.921	0.899	0.91	0.821
	MHCNetSeq	0.957	0.895	0.911	0.903	0.807
HLA-A*24:02	CapsNet-MHC	0.9924	0.9651	0.9594	0.9623	0.9245
	HLAB	0.9913	0.968	0.9594	0.9637	0.9275
	NetMHCcons-1.1	0.99	0.965	0.944	0.955	0.91
	Anthem	0.989	0.956	0.947	0.952	0.904
	NetMHCstabpan-1.0	0.989	0.962	0.942	0.952	0.904
	MixMHCpred-2.0.2	0.988	0.951	0.942	0.946	0.893
	NetMHCpan-4.1	0.988	0.956	0.953	0.955	0.91
	ACME	0.986	0.947	0.95	0.949	0.897
	MHCNetSeq	0.984	0.947	0.94	0.943	0.887
HLA-A*24:06	CapsNet-MHC	1	1	1	1	1
	HLAB	1	1	1	1	1
	MixMHCpred-2.0.2	1	1	0.995	0.997	0.995
	MHCNetSeq	0.999	1	0.995	0.997	0.995
	NetMHCpan-4.1	0.996	1	0.985	0.992	0.986
	NetMHCstabpan-1.0	0.995	1	0.98	0.99	0.981
	NetMHCcons-1.1	0.994	1	0.98	0.99	0.981
	Anthem	0.981	0.93	0.974	0.952	0.906
HLA-A*26:01	CapsNet-MHC	0.986	0.9385	0.9697	0.9542	0.9088
	ACME	0.985	0.972	0.963	0.968	0.935
	HLAB	0.9801	0.9385	0.9697	0.9542	0.9088
	NetMHCcons-1.1	0.979	0.926	0.943	0.935	0.87
	NetMHCpan-4.1	0.977	0.923	0.958	0.941	0.882
	Anthem	0.974	0.941	0.944	0.943	0.886
	NetMHCstabpan-1.0	0.967	0.905	0.926	0.915	0.832
	MixMHCpred-2.0.2	0.938	0.897	0.897	0.897	0.794
	MHCNetSeq	0.924	0.859	0.917	0.888	0.777
HLA-A*29:02	CapsNet-MHC	0.9837	0.9592	0.9472	0.9532	0.9064
	MixMHCpred-2.0.2	0.98	0.942	0.934	0.938	0.876
	HLAB	0.977	0.9224	0.9512	0.9369	0.8741
	Anthem	0.977	0.92	0.951	0.936	0.871
	NetMHCpan-4.1	0.977	0.931	0.947	0.939	0.878
	NetMHCcons-1.1	0.976	0.93	0.948	0.939	0.878

	ACME	0.972	0.937	0.939	0.938	0.877
	NetMHCstabpan-1.0	0.971	0.925	0.932	0.928	0.857
	MHCNetSeq	0.932	0.855	0.918	0.887	0.775
HLA-A*30:01	Anthem	0.923	0.831	0.858	0.844	0.692
	ACME	0.919	0.831	0.862	0.846	0.698
	NetMHCcons-1.1	0.891	0.887	0.774	0.831	0.667
	NetMHCpan-4.1	0.856	0.834	0.823	0.828	0.659
	CapsNet-MHC	0.8442	0.7949	0.775	0.7848	0.5699
	NetMHCstabpan-1.0	0.827	0.772	0.759	0.765	0.532
	HLAB	0.8038	0.8718	0.45	0.6582	0.3541
	MixMHCpred-2.0.2	0.785	0.728	0.728	0.728	0.459
	MHCNetSeq	0.666	0.559	0.79	0.674	0.359
HLA-A*30:02	MixMHCpred-2.0.2	0.988	0.972	0.971	0.971	0.943
	NetMHCcons-1.1	0.982	0.966	0.955	0.961	0.922
	CapsNet-MHC	0.9781	1	0.9167	0.958	0.9194
	ACME	0.978	0.962	0.945	0.954	0.907
	Anthem	0.976	0.924	0.95	0.937	0.875
	NetMHCpan-4.1	0.972	0.935	0.951	0.943	0.886
	NetMHCstabpan-1.0	0.967	0.92	0.925	0.922	0.846
	HLAB	0.9577	0.9014	0.9444	0.9231	0.8468
	MHCNetSeq	0.792	0.68	0.875	0.778	0.568
HLA-A*31:01	CapsNet-MHC	0.9769	0.9532	0.9025	0.9278	0.8568
	HLAB	0.968	0.9447	0.8941	0.9193	0.8397
	Anthem	0.957	0.929	0.888	0.908	0.818
	NetMHCpan-4.1	0.951	0.899	0.879	0.889	0.779
	NetMHCcons-1.1	0.95	0.899	0.881	0.89	0.781
	NetMHCstabpan-1.0	0.95	0.9	0.883	0.892	0.783
	ACME	0.945	0.899	0.877	0.888	0.776
	MixMHCpred-2.0.2	0.869	0.789	0.806	0.798	0.596
	MHCNetSeq	0.849	0.764	0.832	0.799	0.599
HLA-A*32:01	ACME	0.964	0.915	0.915	0.915	0.83
	NetMHCcons-1.1	0.959	0.901	0.913	0.907	0.814
	Anthem	0.957	0.863	0.942	0.902	0.807
	NetMHCpan-4.1	0.956	0.921	0.947	0.934	0.868
	CapsNet-MHC	0.9518	0.8783	0.8966	0.8874	0.775
	NetMHCstabpan-1.0	0.943	0.888	0.909	0.898	0.798
	MixMHCpred-2.0.2	0.939	0.893	0.901	0.896	0.793
	HLAB	0.9366	0.8783	0.9224	0.9004	0.8016
	MHCNetSeq	0.918	0.861	0.914	0.887	0.776
HLA-A*33:01	HLAB	0.9824	1	0.9242	0.962	0.9267
	MixMHCpred-2.0.2	0.981	0.959	0.936	0.947	0.895
	CapsNet-MHC	0.9749	0.9924	0.8939	0.943	0.8903
	Anthem	0.972	0.946	0.913	0.93	0.861

	NetMHCcons-1.1	0.962	0.94	0.895	0.917	0.836
	NetMHCstabpan-1.0	0.962	0.941	0.902	0.921	0.844
	ACME	0.959	0.912	0.91	0.911	0.822
	NetMHCpan-4.1	0.957	0.916	0.907	0.911	0.823
	MHCNetSeq	0.852	0.774	0.827	0.8	0.602
HLA-A*68:01	HLAB	0.9845	0.8056	0.9751	0.8904	0.7922
	CapsNet-MHC	0.9838	0.9833	0.9086	0.9459	0.8943
	Anthem	0.974	0.943	0.924	0.934	0.867
	ACME	0.973	0.953	0.913	0.934	0.867
	NetMHCcons-1.1	0.97	0.942	0.913	0.927	0.855
	NetMHCpan-4.1	0.969	0.945	0.91	0.927	0.855
	NetMHCstabpan-1.0	0.966	0.928	0.907	0.917	0.835
	MixMHCpred-2.0.2	0.9	0.807	0.859	0.833	0.667
	MHCNetSeq	0.837	0.758	0.822	0.79	0.582
HLA-A*68:02	CapsNet-MHC	0.9512	0.8944	0.898	0.8962	0.7924
	HLAB	0.9436	0.901	0.9046	0.9028	0.8056
	Anthem	0.931	0.871	0.883	0.877	0.755
	NetMHCcons-1.1	0.92	0.86	0.865	0.863	0.726
	ACME	0.92	0.858	0.898	0.878	0.756
	NetMHCstabpan-1.0	0.918	0.86	0.862	0.861	0.722
	NetMHCpan-4.1	0.916	0.846	0.874	0.86	0.72
	MixMHCpred-2.0.2	0.885	0.79	0.846	0.818	0.638
	MHCNetSeq	0.779	0.673	0.762	0.718	0.438
HLA-A*69:01	CapsNet-MHC	0.9954	0.96	0.9231	0.9412	0.8831
	NetMHCpan-4.1	0.985	0.968	0.952	0.96	0.921
	HLAB	0.9815	0.96	0.9231	0.9412	0.8831
	Anthem	0.975	0.936	0.925	0.93	0.862
	NetMHCcons-1.1	0.965	0.916	0.912	0.914	0.829
	ACME	0.964	0.92	0.916	0.918	0.837
	NetMHCstabpan-1.0	0.959	0.92	0.904	0.912	0.825
	MixMHCpred-2.0.2	0.944	0.88	0.88	0.88	0.762
	MHCNetSeq	0.898	0.8	0.9	0.85	0.705
HLA-B*07:02	CapsNet-MHC	0.9924	0.972	0.9572	0.9646	0.9293
	NetMHCcons-1.1	0.989	0.963	0.962	0.962	0.925
	NetMHCstabpan-1.0	0.989	0.959	0.959	0.959	0.918
	Anthem	0.988	0.959	0.955	0.957	0.914
	NetMHCpan-4.1	0.988	0.96	0.955	0.957	0.914
	ACME	0.988	0.959	0.958	0.959	0.918
	HLAB	0.9854	0.972	0.959	0.9655	0.9311
	MixMHCpred-2.0.2	0.984	0.942	0.939	0.941	0.882
	MHCNetSeq	0.981	0.932	0.946	0.939	0.878
HLA-B*08:01	ACME	0.983	0.98	0.945	0.962	0.926
	NetMHCcons-1.1	0.976	0.953	0.932	0.942	0.885

	NetMHCpan-4.1	0.968	0.931	0.91	0.92	0.841
	Anthem	0.965	0.893	0.924	0.908	0.819
	NetMHCstabpan-1.0	0.961	0.919	0.9	0.91	0.82
	CapsNet-MHC	0.9559	0.8986	0.8857	0.8921	0.7843
	HLAB	0.9484	0.942	0.8571	0.8993	0.8017
	MHCNetSeq	0.917	0.855	0.848	0.852	0.704
	MixMHCpred-2.0.2	0.879	0.839	0.777	0.808	0.618
HLA-B*13:02	CapsNet-MHC	1	0.9524	1	0.9767	0.9544
	HLAB	0.987	0.9048	0.9545	0.9302	0.8612
	NetMHCpan-4.1	0.986	0.976	0.943	0.959	0.923
	NetMHCcons-1.1	0.979	0.933	0.971	0.952	0.906
	NetMHCstabpan-1.0	0.97	0.919	0.947	0.934	0.868
	Anthem	0.966	0.886	0.94	0.913	0.829
	MixMHCpred-2.0.2	0.942	0.891	0.924	0.907	0.816
	MHCNetSeq	0.922	0.886	0.891	0.888	0.778
	ACME	0.882	0.862	0.829	0.845	0.693
HLA-B*14:02	NetMHCpan-4.1	0.994	0.997	0.984	0.991	0.982
	CapsNet-MHC	0.991	0.9744	0.925	0.9494	0.8999
	NetMHCstabpan-1.0	0.99	1	0.974	0.987	0.975
	NetMHCcons-1.1	0.985	0.992	0.954	0.973	0.947
	Anthem	0.972	0.892	0.963	0.928	0.859
	ACME	0.968	0.949	0.926	0.937	0.875
	MixMHCpred-2.0.2	0.961	0.91	0.928	0.919	0.839
	HLAB	0.9391	0.8974	0.825	0.8608	0.7238
	MHCNetSeq	0.881	0.874	0.797	0.836	0.676
HLA-B*15:01	CapsNet-MHC	0.9892	0.9757	0.9462	0.9609	0.9222
	Anthem	0.986	0.964	0.943	0.954	0.908
	NetMHCpan-4.1	0.984	0.96	0.953	0.956	0.913
	NetMHCcons-1.1	0.983	0.943	0.947	0.945	0.891
	NetMHCstabpan-1.0	0.982	0.944	0.941	0.943	0.885
	MixMHCpred-2.0.2	0.981	0.942	0.933	0.938	0.875
	ACME	0.981	0.956	0.94	0.948	0.896
	HLAB	0.9808	0.967	0.9444	0.9557	0.9116
	MHCNetSeq	0.97	0.923	0.934	0.928	0.857
HLA-B*18:01	NetMHCpan-4.1	0.994	1	0.964	0.982	0.965
	CapsNet-MHC	0.9939	0.9762	0.9302	0.9529	0.9069
	NetMHCcons-1.1	0.994	0.981	0.974	0.977	0.955
	NetMHCstabpan-1.0	0.993	0.99	0.966	0.978	0.957
	ACME	0.992	0.986	0.959	0.972	0.946
	HLAB	0.9889	1	0.907	0.9529	0.91
	Anthem	0.986	0.948	0.957	0.952	0.905
	MixMHCpred-2.0.2	0.979	0.926	0.933	0.93	0.861
	MHCNetSeq	0.969	0.933	0.924	0.929	0.858

HLA-B*27:01	CapsNet-MHC	0.9988	1	0.9788	0.9894	0.979
	HLAB	0.9984	1	0.9577	0.9788	0.9584
	MixMHCpred-2.0.2	0.998	0.992	0.98	0.986	0.972
	Anthem	0.996	0.995	0.975	0.985	0.97
	NetMHCpan-4.1	0.995	0.993	0.979	0.986	0.972
	ACME	0.99	0.977	0.957	0.967	0.934
	MHCNetSeq	0.99	0.995	0.915	0.955	0.912
	NetMHCcons-1.1	0.987	0.971	0.944	0.958	0.916
	NetMHCstabpan-1.0	0.986	0.973	0.941	0.957	0.914
HLA-B*27:02	CapsNet-MHC	0.9978	0.9779	0.989	0.9835	0.967
	HLAB	0.9953	0.9613	0.9945	0.978	0.9564
	MixMHCpred-2.0.2	0.995	0.972	0.99	0.981	0.963
	Anthem	0.994	0.962	0.986	0.974	0.949
	NetMHCpan-4.1	0.993	0.98	0.978	0.979	0.958
	NetMHCstabpan-1.0	0.99	0.967	0.971	0.969	0.938
	ACME	0.987	0.972	0.95	0.962	0.923
	MHCNetSeq	0.987	0.972	0.914	0.943	0.888
	NetMHCcons-1.1	0.986	0.97	0.955	0.963	0.926
HLA-B*27:03	CapsNet-MHC	0.9951	0.9787	0.9792	0.9789	0.9579
	MHCNetSeq	0.994	0.974	0.972	0.973	0.947
	HLAB	0.9898	0.9574	0.9792	0.9684	0.937
	MixMHCpred-2.0.2	0.982	0.957	0.992	0.975	0.949
	NetMHCpan-4.1	0.982	0.947	0.964	0.955	0.911
	Anthem	0.977	0.949	0.983	0.966	0.932
	NetMHCcons-1.1	0.972	0.934	0.964	0.949	0.899
	NetMHCstabpan-1.0	0.972	0.938	0.964	0.951	0.903
	ACME	0.971	0.932	0.947	0.94	0.879
HLA-B*27:04	HLAB	0.9962	0.9167	1	0.9587	0.9204
	CapsNet-MHC	0.9883	0.9333	1	0.9669	0.9359
	NetMHCpan-4.1	0.985	0.937	0.967	0.952	0.904
	ACME	0.983	0.927	0.957	0.942	0.884
	NetMHCcons-1.1	0.979	0.933	0.975	0.954	0.909
	NetMHCstabpan-1.0	0.978	0.933	0.977	0.955	0.911
	Anthem	0.971	0.913	0.968	0.941	0.883
	MixMHCpred-2.0.2	0.964	0.931	0.98	0.956	0.913
	MHCNetSeq	0.961	0.933	0.945	0.939	0.879
HLA-B*27:05	CapsNet-MHC	0.9064	0.7882	0.8665	0.8274	0.6568
	HLAB	0.8971	0.8041	0.8371	0.8206	0.6415
	Anthem	0.865	0.725	0.836	0.78	0.565
	NetMHCcons-1.1	0.816	0.688	0.782	0.735	0.472
	NetMHCstabpan-1.0	0.816	0.677	0.784	0.731	0.465
	MixMHCpred-2.0.2	0.814	0.695	0.807	0.751	0.505
	NetMHCpan-4.1	0.807	0.676	0.796	0.736	0.477

	ACME	0.791	0.655	0.827	0.741	0.489
	MHCNetSeq	0.784	0.665	0.807	0.736	0.478
HLA-B*27:06	CapsNet-MHC	0.9969	0.94	0.9804	0.9604	0.9215
	MHCNetSeq	0.994	0.98	0.93	0.955	0.912
	HLAB	0.9902	0.96	0.9412	0.9505	0.9012
	MixMHCpred-2.0.2	0.99	0.964	0.976	0.97	0.94
	NetMHCpan-4.1	0.985	0.932	0.948	0.94	0.88
	ACME	0.98	0.93	0.938	0.934	0.869
	Anthem	0.976	0.932	0.987	0.959	0.921
	NetMHCcons-1.1	0.969	0.904	0.932	0.918	0.837
	NetMHCstabpan-1.0	0.969	0.908	0.938	0.923	0.847
HLA-B*27:07	HLAB	0.9989	1	0.9905	0.9952	0.9905
	MixMHCpred-2.0.2	0.998	0.987	0.987	0.988	0.975
	CapsNet-MHC	0.9963	0.9904	0.9905	0.9904	0.9809
	Anthem	0.996	0.969	0.986	0.978	0.956
	NetMHCpan-4.1	0.994	0.988	0.977	0.983	0.966
	MHCNetSeq	0.994	1	0.955	0.977	0.956
	ACME	0.992	0.986	0.975	0.98	0.961
	NetMHCstabpan-1.0	0.989	0.982	0.965	0.974	0.948
	NetMHCcons-1.1	0.987	0.988	0.951	0.97	0.94
HLA-B*27:08	CapsNet-MHC	0.9994	0.9906	0.972	0.9812	0.9631
	HLAB	0.9993	1	0.9626	0.9812	0.9626
	MixMHCpred-2.0.2	0.999	0.993	0.988	0.99	0.98
	Anthem	0.995	0.976	0.982	0.979	0.957
	NetMHCstabpan-1.0	0.995	0.988	0.972	0.98	0.96
	NetMHCpan-4.1	0.993	0.975	0.967	0.971	0.943
	NetMHCcons-1.1	0.993	0.978	0.969	0.973	0.947
	ACME	0.99	0.982	0.959	0.97	0.941
HLA-B*27:09	CapsNet-MHC	0.9879	0.9273	0.9502	0.9388	0.8778
	Anthem	0.969	0.887	0.94	0.914	0.829
	NetMHCpan-4.1	0.955	0.887	0.926	0.907	0.815
	MixMHCpred-2.0.2	0.953	0.871	0.94	0.906	0.814
	HLAB	0.9512	0.9682	0.905	0.9365	0.8748
	NetMHCcons-1.1	0.948	0.864	0.922	0.893	0.787
	NetMHCstabpan-1.0	0.948	0.861	0.927	0.894	0.79
	MHCNetSeq	0.948	0.88	0.918	0.899	0.799
	ACME	0.94	0.863	0.929	0.895	0.793
HLA-B*35:01	CapsNet-MHC	0.9695	0.9355	0.8964	0.9159	0.8325
	Anthem	0.969	0.929	0.931	0.93	0.861
	NetMHCpan-4.1	0.969	0.932	0.956	0.944	0.888
	NetMHCcons-1.1	0.964	0.922	0.939	0.931	0.861
	NetMHCstabpan-1.0	0.964	0.917	0.935	0.926	0.852
	HLAB	0.9636	0.9355	0.9214	0.9284	0.857

	ACME	0.963	0.927	0.94	0.933	0.867
	MixMHCpred-2.0.2	0.961	0.918	0.925	0.921	0.843
	MHCNetSeq	0.919	0.816	0.867	0.842	0.685
HLA-B*35:03	NetMHCpan-4.1	0.997	0.998	0.986	0.992	0.984
	CapsNet-MHC	0.9927	0.9388	0.94	0.9394	0.8788
	ACME	0.987	0.961	0.947	0.954	0.909
	HLAB	0.9833	0.9592	0.96	0.9596	0.9192
	Anthem	0.978	0.922	0.952	0.937	0.875
	NetMHCcons-1.1	0.977	0.904	0.968	0.936	0.874
	NetMHCstabpan-1.0	0.974	0.902	0.953	0.928	0.857
	MixMHCpred-2.0.2	0.967	0.935	0.953	0.944	0.888
	MHCNetSeq	0.929	0.835	0.92	0.878	0.759
HLA-B*35:08	HLAB	0.9919	0.9565	1	0.9785	0.9579
	CapsNet-MHC	0.9769	0.9565	0.9787	0.9677	0.9357
	NetMHCpan-4.1	0.976	0.957	0.98	0.968	0.937
	ACME	0.969	0.926	0.963	0.945	0.89
	Anthem	0.963	0.913	0.949	0.931	0.864
	NetMHCcons-1.1	0.963	0.917	0.937	0.927	0.855
	NetMHCstabpan-1.0	0.963	0.913	0.939	0.926	0.853
	MixMHCpred-2.0.2	0.946	0.872	0.904	0.888	0.777
	MHCNetSeq	0.939	0.885	0.891	0.888	0.777
HLA-B*37:01	CapsNet-MHC	0.9983	0.9877	0.939	0.9632	0.9275
	HLAB	0.9983	0.9259	0.9878	0.9571	0.9158
	NetMHCpan-4.1	0.993	0.996	0.979	0.988	0.976
	NetMHCcons-1.1	0.99	0.973	0.973	0.973	0.946
	NetMHCstabpan-1.0	0.989	0.98	0.977	0.978	0.957
	Anthem	0.986	0.953	0.956	0.955	0.91
	ACME	0.984	0.962	0.972	0.967	0.933
	MixMHCpred-2.0.2	0.93	0.906	0.847	0.877	0.755
	MHCNetSeq	0.923	0.856	0.878	0.867	0.734
HLA-B*39:01	HLAB	0.9998	1	0.9808	0.9903	0.9808
	CapsNet-MHC	0.9989	0.9804	0.9615	0.9709	0.9419
	NetMHCcons-1.1	0.998	0.988	0.971	0.979	0.959
	NetMHCstabpan-1.0	0.998	0.988	0.976	0.982	0.965
	Anthem	0.997	0.965	0.974	0.969	0.939
	MixMHCpred-2.0.2	0.997	0.973	0.986	0.979	0.96
	NetMHCpan-4.1	0.997	0.982	0.994	0.988	0.977
	ACME	0.997	0.98	0.974	0.978	0.955
	MHCNetSeq	0.997	0.98	0.982	0.981	0.963
HLA-B*40:01	CapsNet-MHC	0.9977	0.9823	0.9648	0.9735	0.9472
	NetMHCcons-1.1	0.995	0.972	0.978	0.975	0.951
	ACME	0.995	0.988	0.968	0.978	0.956
	Anthem	0.994	0.959	0.968	0.964	0.927

	HLAB	0.9937	0.969	0.9604	0.9647	0.9294
	NetMHCpan-4.1	0.993	0.968	0.971	0.969	0.939
	NetMHCstabpan-1.0	0.992	0.966	0.976	0.971	0.942
	MixMHCpred-2.0.2	0.985	0.956	0.947	0.951	0.903
	MHCNetSeq	0.981	0.942	0.963	0.953	0.906
HLA-B*40:02	CapsNet-MHC	0.995	0.978	0.9615	0.9697	0.9396
	MixMHCpred-2.0.2	0.992	0.964	0.963	0.963	0.926
	Anthem	0.991	0.97	0.967	0.969	0.938
	NetMHCcons-1.1	0.991	0.97	0.973	0.971	0.943
	NetMHCpan-4.1	0.99	0.984	0.964	0.974	0.948
	NetMHCstabpan-1.0	0.989	0.967	0.964	0.966	0.932
	ACME	0.989	0.967	0.959	0.963	0.926
	HLAB	0.9886	0.978	0.9643	0.9711	0.9423
	MHCNetSeq	0.968	0.915	0.92	0.917	0.835
HLA-B*41:01	ACME	0.994	0.963	0.981	0.972	0.945
	HLAB	0.9926	0.9375	1	0.9697	0.941
	NetMHCpan-4.1	0.99	0.944	0.981	0.963	0.926
	CapsNet-MHC	0.989	0.9375	1	0.9697	0.941
	NetMHCstabpan-1.0	0.983	0.938	0.988	0.963	0.926
	MixMHCpred-2.0.2	0.982	0.925	0.975	0.95	0.902
	NetMHCcons-1.1	0.977	0.938	0.981	0.96	0.92
	MHCNetSeq	0.969	0.95	0.938	0.944	0.889
	Anthem	0.929	0.862	0.991	0.927	0.861
HLA-B*44:02	NetMHCpan-4.1	0.993	0.976	0.967	0.971	0.942
	CapsNet-MHC	0.9892	0.9684	0.9419	0.9552	0.9106
	Anthem	0.988	0.955	0.946	0.95	0.901
	NetMHCcons-1.1	0.988	0.937	0.957	0.947	0.894
	ACME	0.985	0.946	0.954	0.95	0.901
	HLAB	0.9848	0.9539	0.954	0.9539	0.9079
	NetMHCstabpan-1.0	0.984	0.922	0.961	0.942	0.884
	MixMHCpred-2.0.2	0.983	0.93	0.932	0.931	0.863
	MHCNetSeq	0.963	0.899	0.926	0.912	0.825
HLA-B*44:03	CapsNet-MHC	0.994	0.9835	0.9671	0.9753	0.9507
	NetMHCpan-4.1	0.994	0.978	0.976	0.977	0.954
	NetMHCcons-1.1	0.994	0.98	0.97	0.975	0.95
	ACME	0.994	0.98	0.971	0.976	0.951
	HLAB	0.9922	0.9808	0.9753	0.9781	0.9561
	Anthem	0.991	0.959	0.964	0.961	0.923
	NetMHCstabpan-1.0	0.991	0.972	0.956	0.964	0.928
	MixMHCpred-2.0.2	0.99	0.954	0.945	0.95	0.9
	MHCNetSeq	0.98	0.946	0.96	0.953	0.906
HLA-B*44:27	CapsNet-MHC	1	1	1	1	1
	HLAB	1	1	1	1	1

	MixMHCpred-2.0.2	0.999	1	0.993	0.996	0.993
	NetMHCstabpan-1.0	0.999	1	0.993	0.996	0.993
	NetMHCcons-1.1	0.998	0.993	0.993	0.993	0.986
	MHCNetSeq	0.998	0.986	0.993	0.989	0.979
	ACME	0.997	0.986	0.986	0.986	0.972
	NetMHCpan-4.1	0.992	1	0.986	0.993	0.987
	Anthem	0.978	0.886	0.983	0.935	0.875
HLA-B*45:01	HLAB	0.9803	0.9444	0.8624	0.9032	0.8093
	CapsNet-MHC	0.9774	0.9537	0.9174	0.9355	0.8716
	Anthem	0.971	0.911	0.932	0.921	0.844
	NetMHCpan-4.1	0.966	0.934	0.95	0.942	0.885
	NetMHCcons-1.1	0.966	0.909	0.946	0.928	0.857
	NetMHCstabpan-1.0	0.959	0.911	0.952	0.931	0.864
	ACME	0.959	0.919	0.935	0.927	0.855
	MixMHCpred-2.0.2	0.924	0.861	0.835	0.848	0.698
	MHCNetSeq	0.835	0.741	0.824	0.783	0.569
HLA-B*46:01	MHCNetSeq	0.978	0.946	0.941	0.943	0.887
	Anthem	0.971	0.934	0.965	0.95	0.9
	MixMHCpred-2.0.2	0.966	0.946	0.981	0.963	0.927
	HLAB	0.9578	0.7763	0.9481	0.8627	0.7359
	CapsNet-MHC	0.9498	0.9079	0.8961	0.902	0.804
	NetMHCstabpan-1.0	0.946	0.908	0.921	0.914	0.829
	NetMHCpan-4.1	0.945	0.915	0.918	0.916	0.833
	NetMHCcons-1.1	0.945	0.903	0.918	0.911	0.822
	ACME	0.927	0.851	0.864	0.858	0.717
HLA-B*49:01	NetMHCpan-4.1	0.995	0.995	0.974	0.985	0.969
	Anthem	0.986	0.939	0.963	0.95	0.902
	NetMHCcons-1.1	0.984	0.953	0.974	0.964	0.927
	ACME	0.984	0.963	0.964	0.964	0.927
	NetMHCstabpan-1.0	0.983	0.953	0.964	0.958	0.917
	CapsNet-MHC	0.9828	0.9346	0.9259	0.9302	0.8605
	HLAB	0.9792	0.9813	0.9444	0.9628	0.9262
	MixMHCpred-2.0.2	0.967	0.906	0.942	0.924	0.849
	MHCNetSeq	0.942	0.927	0.87	0.899	0.799
HLA-B*50:01	CapsNet-MHC	0.9974	0.9767	0.9773	0.977	0.954
	NetMHCpan-4.1	0.985	0.975	0.947	0.96	0.922
	NetMHCstabpan-1.0	0.981	0.963	0.958	0.96	0.921
	NetMHCcons-1.1	0.98	0.961	0.953	0.957	0.914
	HLAB	0.9797	0.9535	0.9545	0.954	0.908
	Anthem	0.975	0.884	0.95	0.917	0.836
	MixMHCpred-2.0.2	0.97	0.916	0.928	0.922	0.845
	ACME	0.968	0.944	0.935	0.939	0.879
	MHCNetSeq	0.956	0.902	0.895	0.899	0.799

HLA-B*51:01	HLAB	0.9683	0.9024	0.9636	0.9331	0.8678
	CapsNet-MHC	0.9668	0.9207	0.9333	0.9271	0.8542
	Anthem	0.938	0.826	0.918	0.872	0.747
	NetMHCcons-1.1	0.87	0.79	0.754	0.772	0.545
	NetMHCpan-4.1	0.869	0.779	0.781	0.78	0.563
	NetMHCstabpan-1.0	0.865	0.786	0.749	0.767	0.536
	MixMHCpred-2.0.2	0.823	0.685	0.829	0.757	0.519
	ACME	0.792	0.665	0.81	0.738	0.481
	MHCNetSeq	0.746	0.685	0.715	0.7	0.402
HLA-B*53:01	MixMHCpred-2.0.2	0.994	1	0.978	0.989	0.978
	NetMHCcons-1.1	0.986	0.974	0.949	0.962	0.924
	Anthem	0.983	0.895	0.972	0.934	0.87
	NetMHCpan-4.1	0.981	0.956	0.939	0.948	0.896
	CapsNet-MHC	0.9794	0.9661	0.9167	0.9412	0.8835
	NetMHCstabpan-1.0	0.977	0.934	0.929	0.931	0.863
	HLAB	0.9675	0.8983	0.9667	0.9328	0.8674
	ACME	0.961	0.908	0.942	0.926	0.852
	MHCNetSeq	0.761	0.686	0.732	0.709	0.42
HLA-B*54:01	HLAB	0.9729	0.9242	0.9552	0.9398	0.8801
	CapsNet-MHC	0.9711	0.8485	0.9851	0.9173	0.8422
	ACME	0.956	0.912	0.94	0.926	0.852
	NetMHCstabpan-1.0	0.947	0.929	0.915	0.922	0.845
	NetMHCcons-1.1	0.944	0.925	0.92	0.923	0.846
	Anthem	0.943	0.824	0.954	0.889	0.786
	NetMHCpan-4.1	0.939	0.888	0.908	0.898	0.796
	MixMHCpred-2.0.2	0.932	0.865	0.9	0.883	0.766
	MHCNetSeq	0.735	0.706	0.753	0.73	0.461
HLA-B*56:01	HLAB	1	0.8333	1	0.9178	0.8468
	MixMHCpred-2.0.2	0.999	1	0.986	0.993	0.987
	NetMHCpan-4.1	0.999	0.997	0.992	0.994	0.989
	NetMHCcons-1.1	0.999	1	0.989	0.994	0.989
	NetMHCstabpan-1.0	0.999	1	0.994	0.997	0.995
	CapsNet-MHC	0.9985	1	0.9189	0.9589	0.921
	ACME	0.997	1	0.986	0.993	0.987
	Anthem	0.995	0.994	0.979	0.987	0.974
	MHCNetSeq	0.987	0.969	0.964	0.966	0.933
HLA-B*57:01	CapsNet-MHC	0.9764	0.9171	0.9659	0.9415	0.8841
	Anthem	0.965	0.881	0.962	0.921	0.846
	NetMHCcons-1.1	0.963	0.894	0.949	0.922	0.844
	NetMHCstabpan-1.0	0.963	0.888	0.952	0.92	0.841
	MixMHCpred-2.0.2	0.959	0.892	0.952	0.922	0.845
	MHCNetSeq	0.959	0.908	0.938	0.924	0.847
	NetMHCpan-4.1	0.958	0.893	0.936	0.915	0.83

	HLAB	0.9578	0.935	0.9026	0.9188	0.838
	ACME	0.947	0.887	0.934	0.911	0.822
HLA-B*57:03	NetMHCpan-4.1	0.984	0.946	0.956	0.951	0.902
	NetMHCcons-1.1	0.982	0.942	0.966	0.954	0.909
	NetMHCstabpan-1.0	0.982	0.944	0.966	0.955	0.91
	CapsNet-MHC	0.9818	0.9539	0.9541	0.954	0.908
	Anthem	0.978	0.945	0.971	0.958	0.916
	MHCNetSeq	0.974	0.927	0.954	0.94	0.881
	HLAB	0.9652	0.8848	0.9541	0.9195	0.8411
	ACME	0.965	0.911	0.933	0.922	0.845
HLA-B*58:01	NetMHCcons-1.1	0.976	0.944	0.916	0.93	0.861
	CapsNet-MHC	0.9737	0.9293	0.9296	0.9295	0.8589
	NetMHCpan-4.1	0.971	0.916	0.929	0.923	0.846
	Anthem	0.97	0.901	0.929	0.915	0.831
	NetMHCstabpan-1.0	0.965	0.895	0.918	0.906	0.813
	ACME	0.965	0.901	0.936	0.919	0.838
	HLAB	0.9572	0.899	0.9296	0.9144	0.8291
	MixMHCpred-2.0.2	0.941	0.869	0.921	0.895	0.791
	MHCNetSeq	0.909	0.848	0.952	0.9	0.804
HLA-C*01:02	NetMHCstabpan-1.0	0.982	0.951	0.976	0.964	0.927
	CapsNet-MHC	0.9813	0.9512	0.8795	0.9152	0.8326
	Anthem	0.978	0.922	0.916	0.919	0.838
	NetMHCpan-4.1	0.977	0.952	0.954	0.953	0.906
	NetMHCcons-1.1	0.972	0.947	0.949	0.948	0.896
	MixMHCpred-2.0.2	0.965	0.897	0.903	0.9	0.801
	HLAB	0.9506	0.9634	0.8554	0.9091	0.8232
	MHCNetSeq	0.844	0.75	0.782	0.766	0.533
HLA-C*02:02	CapsNet-MHC	0.9875	0.9675	0.9113	0.9393	0.88
	NetMHCpan-4.1	0.986	0.964	0.953	0.959	0.918
	Anthem	0.982	0.93	0.932	0.931	0.863
	NetMHCstabpan-1.0	0.981	0.923	0.943	0.933	0.867
	NetMHCcons-1.1	0.976	0.922	0.936	0.929	0.859
	HLAB	0.9679	0.9268	0.9032	0.915	0.8302
	MixMHCpred-2.0.2	0.966	0.911	0.911	0.911	0.822
	MHCNetSeq	0.864	0.789	0.781	0.785	0.572
HLA-C*03:03	CapsNet-MHC	0.9435	0.8732	0.9444	0.9091	0.8201
	HLAB	0.9435	0.9014	0.9722	0.9371	0.8762
	NetMHCpan-4.1	0.936	0.876	0.975	0.926	0.855
	Anthem	0.929	0.837	0.938	0.887	0.779
	MixMHCpred-2.0.2	0.928	0.876	0.918	0.897	0.795
	NetMHCstabpan-1.0	0.925	0.86	0.945	0.902	0.809
	NetMHCcons-1.1	0.923	0.867	0.924	0.895	0.794
	MHCNetSeq	0.877	0.803	0.814	0.809	0.617

HLA-C*03:04	CapsNet-MHC	0.9872	0.939	0.9036	0.9212	0.843
	NetMHCpan-4.1	0.984	0.94	0.943	0.941	0.883
	NetMHCcons-1.1	0.983	0.944	0.944	0.944	0.888
	NetMHCstabpan-1.0	0.983	0.942	0.943	0.943	0.886
	HLAB	0.9827	0.8902	0.9639	0.9273	0.8567
	MixMHCpred-2.0.2	0.979	0.947	0.939	0.943	0.887
	Anthem	0.976	0.897	0.969	0.933	0.869
	MHCNetSeq	0.897	0.844	0.802	0.823	0.649
HLA-C*04:01	CapsNet-MHC	0.9695	0.8961	0.9397	0.9179	0.8366
	HLAB	0.9598	0.7619	0.9914	0.8769	0.7742
	Anthem	0.953	0.839	0.942	0.891	0.786
	NetMHCpan-4.1	0.925	0.866	0.915	0.89	0.782
	NetMHCcons-1.1	0.916	0.845	0.888	0.866	0.734
	NetMHCstabpan-1.0	0.916	0.839	0.882	0.86	0.722
	MixMHCpred-2.0.2	0.911	0.833	0.897	0.865	0.732
	MHCNetSeq	0.678	0.582	0.71	0.646	0.295
HLA-C*05:01	CapsNet-MHC	0.9784	0.9068	0.9748	0.9409	0.8838
	HLAB	0.9771	0.8983	0.9832	0.9409	0.885
	Anthem	0.968	0.864	0.956	0.91	0.824
	NetMHCcons-1.1	0.967	0.936	0.954	0.945	0.889
	NetMHCpan-4.1	0.966	0.931	0.945	0.938	0.876
	NetMHCstabpan-1.0	0.965	0.935	0.951	0.943	0.886
	MixMHCpred-2.0.2	0.96	0.922	0.903	0.913	0.826
	MHCNetSeq	0.886	0.864	0.753	0.808	0.621
HLA-C*06:02	CapsNet-MHC	0.9491	0.866	0.9184	0.8923	0.7856
	HLAB	0.9427	0.9072	0.8878	0.8974	0.7951
	Anthem	0.925	0.792	0.925	0.858	0.724
	NetMHCpan-4.1	0.917	0.833	0.93	0.881	0.767
	NetMHCstabpan-1.0	0.915	0.831	0.898	0.864	0.732
	MixMHCpred-2.0.2	0.905	0.833	0.862	0.847	0.696
	NetMHCcons-1.1	0.905	0.838	0.876	0.857	0.716
	MHCNetSeq	0.79	0.702	0.776	0.739	0.482
HLA-C*07:01	CapsNet-MHC	0.99	0.975	0.963	0.9689	0.938
	NetMHCpan-4.1	0.987	0.954	0.954	0.954	0.908
	HLAB	0.9849	0.975	0.9506	0.9627	0.9258
	Anthem	0.978	0.9	0.94	0.92	0.841
	NetMHCstabpan-1.0	0.972	0.914	0.912	0.913	0.827
	NetMHCcons-1.1	0.96	0.933	0.87	0.901	0.805
	MixMHCpred-2.0.2	0.872	0.828	0.806	0.817	0.634
	MHCNetSeq	0.861	0.773	0.83	0.801	0.604
HLA-C*07:02	NetMHCpan-4.1	0.975	0.92	0.944	0.932	0.865
	CapsNet-MHC	0.9744	0.9455	0.9107	0.9279	0.8564
	HLAB	0.9698	0.8909	0.9464	0.9189	0.839

		Anthem	0.951	0.858	0.912	0.885	0.773
		NetMHCstabpan-1.0	0.949	0.884	0.9	0.892	0.784
		NetMHCcons-1.1	0.946	0.925	0.856	0.891	0.784
		MixMHCpred-2.0.2	0.916	0.862	0.829	0.845	0.693
		MHCNetSeq	0.87	0.812	0.813	0.813	0.626
	HLA-C*07:04	NetMHCpan-4.1	0.996	1	0.978	0.989	0.979
		NetMHCstabpan-1.0	0.988	0.985	0.957	0.97	0.941
		Anthem	0.985	0.932	0.945	0.938	0.877
		CapsNet-MHC	0.9735	1	0.8788	0.9385	0.8838
		NetMHCcons-1.1	0.971	0.95	0.931	0.941	0.882
		HLAB	0.9538	0.9688	0.8485	0.9077	0.8219
		MHCNetSeq	0.563	0.506	0.688	0.597	0.203
	HLA-C*08:02	CapsNet-MHC	0.9927	0.9643	0.9646	0.9644	0.9289
		NetMHCpan-4.1	0.989	0.987	0.969	0.978	0.956
		HLAB	0.9885	0.9732	0.9381	0.9556	0.9117
		Anthem	0.987	0.971	0.954	0.963	0.926
		NetMHCstabpan-1.0	0.985	0.943	0.949	0.946	0.892
		NetMHCcons-1.1	0.983	0.94	0.954	0.947	0.895
		MixMHCpred-2.0.2	0.969	0.906	0.927	0.916	0.833
		MHCNetSeq	0.92	0.84	0.87	0.855	0.711
	HLA-C*14:02	MixMHCpred-2.0.2	0.997	0.984	0.987	0.986	0.972
		NetMHCpan-4.1	0.997	0.995	0.983	0.989	0.978
		NetMHCcons-1.1	0.995	0.991	0.976	0.984	0.967
		NetMHCstabpan-1.0	0.994	0.986	0.969	0.977	0.955
		CapsNet-MHC	0.9925	1	0.9538	0.9767	0.9545
		Anthem	0.99	0.978	0.977	0.978	0.955
		HLAB	0.9899	0.9531	0.9846	0.969	0.9384
		MHCNetSeq	0.669	0.671	0.622	0.646	0.293
	HLA-C*16:01	NetMHCpan-4.1	0.974	0.942	0.922	0.932	0.864
		NetMHCcons-1.1	0.971	0.946	0.905	0.925	0.852
		NetMHCstabpan-1.0	0.971	0.957	0.898	0.927	0.856
		Anthem	0.961	0.791	0.937	0.864	0.739
		CapsNet-MHC	0.9598	0.913	0.8298	0.871	0.7449
		MixMHCpred-2.0.2	0.953	0.898	0.88	0.889	0.78
		HLAB	0.9431	0.9348	0.8511	0.8925	0.788
		MHCNetSeq	0.752	0.822	0.615	0.719	0.453
11	HLA-A*01:01	CapsNet-MHC	0.9928	0.974	0.9397	0.9568	0.9142
		Anthem	0.992	0.957	0.954	0.955	0.911
		NetMHCpan-4.1	0.992	0.965	0.959	0.962	0.924
		MixMHCpred-2.0.2	0.991	0.954	0.953	0.954	0.907
		HLAB	0.9864	0.9177	0.9655	0.9417	0.8844
		NetMHCcons-1.1	0.985	0.928	0.945	0.936	0.873
		NetMHCstabpan-1.0	0.984	0.926	0.941	0.934	0.867

	ACME	0.983	0.93	0.944	0.937	0.874
	MHCNetSeq	0.946	0.896	0.872	0.884	0.768
HLA-A*02:01	CapsNet-MHC	0.9612	0.9163	0.8807	0.8985	0.7975
	Anthem	0.95	0.88	0.89	0.885	0.771
	HLAB	0.9317	0.9044	0.8887	0.8965	0.7931
	MixMHCpred-2.0.2	0.931	0.872	0.86	0.866	0.732
	NetMHCcons-1.1	0.93	0.855	0.921	0.887	0.777
	NetMHCstabpan-1.0	0.927	0.852	0.913	0.882	0.767
	ACME	0.925	0.862	0.882	0.872	0.744
	NetMHCpan-4.1	0.921	0.863	0.89	0.876	0.753
	MHCNetSeq	0.893	0.807	0.839	0.823	0.646
HLA-A*02:03	CapsNet-MHC	0.9387	0.8077	0.9259	0.8679	0.74
	HLAB	0.9288	0.8462	0.9259	0.8868	0.7754
	NetMHCpan-4.1	0.921	0.885	0.915	0.9	0.801
	ACME	0.912	0.885	0.885	0.885	0.77
	NetMHCcons-1.1	0.906	0.873	0.831	0.852	0.711
	NetMHCstabpan-1.0	0.87	0.769	0.866	0.817	0.64
	Anthem	0.868	0.785	0.904	0.844	0.696
	MixMHCpred-2.0.2	0.843	0.788	0.843	0.815	0.632
	MHCNetSeq	0.811	0.754	0.846	0.8	0.604
HLA-A*02:04	HLAB	0.9891	0.9655	0.9667	0.9661	0.9322
	MixMHCpred-2.0.2	0.981	0.914	0.966	0.94	0.881
	CapsNet-MHC	0.977	0.8966	0.9667	0.9322	0.8662
	ACME	0.965	0.918	0.931	0.924	0.849
	NetMHCpan-4.1	0.954	0.904	0.925	0.914	0.829
	NetMHCstabpan-1.0	0.952	0.914	0.942	0.928	0.857
	MHCNetSeq	0.952	0.911	0.935	0.923	0.846
	NetMHCcons-1.1	0.95	0.907	0.938	0.923	0.847
	Anthem	0.93	0.786	0.974	0.88	0.774
HLA-A*02:05	MHCNetSeq	0.985	0.971	0.923	0.947	0.897
	NetMHCpan-4.1	0.984	0.976	0.953	0.965	0.932
	NetMHCcons-1.1	0.984	1	0.959	0.979	0.962
	CapsNet-MHC	0.9837	1	0.9444	0.9714	0.9444
	NetMHCstabpan-1.0	0.983	0.976	0.971	0.974	0.949
	ACME	0.983	0.982	0.953	0.968	0.937
	HLAB	0.9804	0.9412	0.9444	0.9429	0.8856
	Anthem	0.962	0.894	0.955	0.925	0.853
	MixMHCpred-2.0.2	0.954	0.882	0.906	0.894	0.79
HLA-A*02:07	CapsNet-MHC	0.9771	0.9211	0.9481	0.9346	0.8696
	NetMHCpan-4.1	0.96	0.9	0.954	0.927	0.855
	MixMHCpred-2.0.2	0.958	0.907	0.974	0.94	0.882
	HLAB	0.9564	0.8947	0.9481	0.9216	0.8442
	MHCNetSeq	0.953	0.913	0.914	0.914	0.828

	Anthem	0.948	0.887	0.971	0.929	0.862
	NetMHCcons-1.1	0.94	0.896	0.912	0.904	0.808
	NetMHCstabpan-1.0	0.937	0.891	0.911	0.901	0.802
	ACME	0.915	0.88	0.865	0.872	0.746
HLA-A*03:01	NetMHCpan-4.1	0.982	0.959	0.963	0.961	0.921
	CapsNet-MHC	0.9786	0.9636	0.9639	0.9637	0.9275
	NetMHCcons-1.1	0.978	0.946	0.967	0.957	0.913
	NetMHCstabpan-1.0	0.977	0.955	0.955	0.955	0.91
	Anthem	0.976	0.957	0.938	0.947	0.895
	ACME	0.975	0.943	0.941	0.942	0.884
	HLAB	0.9733	0.9455	0.9578	0.9517	0.9034
	MixMHCpred-2.0.2	0.969	0.928	0.932	0.93	0.861
	MHCNetSeq	0.926	0.848	0.919	0.884	0.77
HLA-A*11:01	CapsNet-MHC	0.9822	0.9614	0.9359	0.9486	0.8975
	HLAB	0.98	0.9571	0.9786	0.9679	0.936
	NetMHCpan-4.1	0.978	0.955	0.963	0.959	0.918
	NetMHCcons-1.1	0.976	0.948	0.968	0.958	0.917
	NetMHCstabpan-1.0	0.976	0.945	0.97	0.958	0.916
	Anthem	0.975	0.95	0.955	0.952	0.905
	MixMHCpred-2.0.2	0.973	0.934	0.942	0.938	0.877
	ACME	0.973	0.955	0.957	0.956	0.913
	MHCNetSeq	0.959	0.914	0.928	0.921	0.843
HLA-A*23:01	CapsNet-MHC	1	1	1	1	1
	HLAB	1	1	1	1	1
	MixMHCpred-2.0.2	0.999	0.985	1	0.992	0.985
	NetMHCpan-4.1	0.999	1	0.996	0.998	0.996
	ACME	0.999	1	0.989	0.994	0.989
	NetMHCcons-1.1	0.997	0.985	0.981	0.983	0.966
	NetMHCstabpan-1.0	0.997	1	0.973	0.987	0.974
	Anthem	0.991	0.954	0.991	0.972	0.946
	MHCNetSeq	0.991	0.954	0.973	0.964	0.928
HLA-A*24:02	CapsNet-MHC	0.9949	0.9624	0.984	0.9732	0.9466
	HLAB	0.9947	0.9677	0.9733	0.9705	0.941
	Anthem	0.99	0.938	0.962	0.95	0.9
	NetMHCpan-4.1	0.99	0.97	0.955	0.963	0.925
	NetMHCstabpan-1.0	0.99	0.971	0.956	0.964	0.928
	NetMHCcons-1.1	0.989	0.974	0.953	0.964	0.928
	MixMHCpred-2.0.2	0.985	0.949	0.932	0.94	0.881
	ACME	0.985	0.945	0.96	0.953	0.906
	MHCNetSeq	0.955	0.893	0.9	0.897	0.794
HLA-A*24:06	CapsNet-MHC	0.9553	0.7895	0.95	0.8718	0.7514
	HLAB	0.9395	0.8421	0.95	0.8974	0.7948
	NetMHCpan-4.1	0.938	0.89	0.953	0.921	0.845

	MixMHCpred-2.0.2	0.936	0.895	0.973	0.934	0.871
	NetMHCstabpan-1.0	0.933	0.879	0.916	0.897	0.797
	NetMHCcons-1.1	0.932	0.863	0.937	0.9	0.804
	Anthem	0.918	0.81	0.943	0.877	0.763
	MHCNetSeq	0.912	0.895	0.853	0.874	0.749
HLA-A*29:02	CapsNet-MHC	0.9914	0.9468	0.9579	0.9524	0.9048
	HLAB	0.9823	0.9574	0.9579	0.9577	0.9153
	MixMHCpred-2.0.2	0.979	0.94	0.978	0.959	0.919
	Anthem	0.973	0.919	0.958	0.939	0.878
	NetMHCcons-1.1	0.973	0.939	0.96	0.95	0.9
	NetMHCpan-4.1	0.972	0.946	0.955	0.95	0.901
	NetMHCstabpan-1.0	0.97	0.935	0.955	0.945	0.891
	ACME	0.968	0.942	0.947	0.944	0.888
	MHCNetSeq	0.945	0.894	0.89	0.892	0.783
HLA-A*31:01	CapsNet-MHC	0.988	0.9848	0.8209	0.9023	0.816
	MixMHCpred-2.0.2	0.986	0.95	0.948	0.949	0.899
	Anthem	0.984	0.967	0.914	0.94	0.882
	NetMHCstabpan-1.0	0.981	0.958	0.926	0.942	0.884
	NetMHCcons-1.1	0.978	0.941	0.941	0.941	0.882
	NetMHCpan-4.1	0.974	0.938	0.932	0.935	0.87
	ACME	0.967	0.911	0.932	0.921	0.843
	HLAB	0.9664	0.9545	0.8955	0.9248	0.8512
	MHCNetSeq	0.931	0.858	0.886	0.872	0.745
HLA-A*32:01	NetMHCstabpan-1.0	0.989	0.96	0.968	0.964	0.928
	CapsNet-MHC	0.9878	0.94	0.9608	0.9505	0.9011
	NetMHCcons-1.1	0.987	0.962	0.954	0.958	0.916
	MixMHCpred-2.0.2	0.985	0.932	0.978	0.955	0.911
	NetMHCpan-4.1	0.982	0.938	0.974	0.956	0.913
	ACME	0.976	0.944	0.964	0.954	0.909
	HLAB	0.9745	0.92	0.9804	0.9505	0.9025
	Anthem	0.953	0.864	0.97	0.917	0.84
	MHCNetSeq	0.95	0.908	0.926	0.917	0.835
HLA-A*68:01	CapsNet-MHC	1	1	0.9273	0.9633	0.9292
	NetMHCstabpan-1.0	0.999	0.998	0.989	0.993	0.987
	HLAB	0.9987	1	0.9455	0.9725	0.9464
	NetMHCpan-4.1	0.998	0.996	0.994	0.995	0.991
	NetMHCcons-1.1	0.998	0.992	0.989	0.991	0.982
	ACME	0.996	0.994	0.978	0.986	0.973
	Anthem	0.995	0.985	0.974	0.98	0.96
	MixMHCpred-2.0.2	0.994	0.991	0.954	0.972	0.946
	MHCNetSeq	0.913	0.867	0.828	0.847	0.695
HLA-A*68:02	CapsNet-MHC	0.9294	0.8429	0.9155	0.8794	0.7606
	HLAB	0.9274	0.8714	0.8592	0.8652	0.7306

	Anthem	0.889	0.8	0.829	0.815	0.63
	MHCNetSeq	0.88	0.807	0.78	0.794	0.591
	MixMHCpred-2.0.2	0.872	0.74	0.886	0.813	0.634
	NetMHCstabpan-1.0	0.851	0.756	0.869	0.812	0.63
	NetMHCcons-1.1	0.839	0.742	0.867	0.804	0.614
	ACME	0.829	0.72	0.86	0.79	0.588
	NetMHCpan-4.1	0.822	0.722	0.864	0.793	0.593
HLA-B*07:02	CapsNet-MHC	0.9845	0.9522	0.967	0.9596	0.9194
	NetMHCpan-4.1	0.984	0.954	0.952	0.953	0.906
	NetMHCcons-1.1	0.984	0.958	0.962	0.959	0.919
	NetMHCstabpan-1.0	0.984	0.954	0.96	0.957	0.914
	HLAB	0.983	0.9375	0.9707	0.9541	0.9087
	MixMHCpred-2.0.2	0.982	0.949	0.946	0.947	0.894
	ACME	0.982	0.965	0.951	0.958	0.916
	Anthem	0.981	0.951	0.955	0.953	0.907
	MHCNetSeq	0.953	0.874	0.898	0.886	0.773
HLA-B*08:01	NetMHCcons-1.1	0.988	0.961	0.967	0.964	0.927
	NetMHCstabpan-1.0	0.988	0.961	0.967	0.964	0.928
	NetMHCpan-4.1	0.981	0.951	0.955	0.953	0.907
	ACME	0.973	0.915	0.93	0.923	0.846
	HLAB	0.9608	0.9697	0.8824	0.9254	0.8543
	CapsNet-MHC	0.9554	0.9697	0.8529	0.9104	0.8271
	Anthem	0.952	0.782	0.942	0.862	0.736
	MixMHCpred-2.0.2	0.936	0.882	0.873	0.877	0.757
	MHCNetSeq	0.727	0.751	0.67	0.711	0.425
HLA-B*15:01	CapsNet-MHC	0.9822	0.9552	0.9509	0.953	0.906
	Anthem	0.973	0.939	0.961	0.95	0.9
	NetMHCpan-4.1	0.973	0.95	0.96	0.955	0.91
	NetMHCstabpan-1.0	0.973	0.941	0.959	0.95	0.9
	ACME	0.972	0.94	0.96	0.95	0.901
	NetMHCcons-1.1	0.971	0.932	0.966	0.949	0.898
	MixMHCpred-2.0.2	0.968	0.928	0.963	0.946	0.892
	HLAB	0.956	0.8341	0.8973	0.8658	0.7329
	MHCNetSeq	0.916	0.987	0.351	0.669	0.437
HLA-B*27:01	HLAB	0.9987	0.9727	1	0.9864	0.9732
	CapsNet-MHC	0.9984	0.9818	0.973	0.9774	0.9548
	MixMHCpred-2.0.2	0.998	0.981	0.987	0.984	0.969
	NetMHCpan-4.1	0.995	0.983	0.978	0.98	0.961
	Anthem	0.993	0.962	0.989	0.975	0.951
	ACME	0.987	0.978	0.95	0.964	0.929
	NetMHCcons-1.1	0.983	0.942	0.953	0.947	0.895
	NetMHCstabpan-1.0	0.981	0.949	0.942	0.945	0.891
	MHCNetSeq	0.901	1	0.211	0.606	0.341

HLA-B*27:02	CapsNet-MHC	0.9999	0.9829	1	0.9915	0.9831
	HLAB	0.9997	1	0.9831	0.9915	0.9831
	MixMHCpred-2.0.2	0.999	1	0.991	0.995	0.991
	Anthem	0.998	0.981	0.985	0.983	0.966
	NetMHCstabpan-1.0	0.997	0.971	0.979	0.975	0.951
	NetMHCpan-4.1	0.994	0.985	0.976	0.98	0.961
	NetMHCcons-1.1	0.994	0.972	0.979	0.976	0.951
	ACME	0.987	0.965	0.968	0.967	0.934
	MHCNetSeq	0.966	1	0.297	0.649	0.417
HLA-B*27:03	CapsNet-MHC	1	1	1	1	1
	HLAB	1	1	1	1	1
	MixMHCpred-2.0.2	0.999	0.976	1	0.987	0.976
	NetMHCpan-4.1	0.997	0.978	0.987	0.982	0.965
	NetMHCcons-1.1	0.997	0.987	0.989	0.988	0.976
	NetMHCstabpan-1.0	0.997	0.984	0.989	0.986	0.973
	Anthem	0.994	0.973	0.981	0.977	0.954
	ACME	0.992	0.973	0.965	0.969	0.938
	MHCNetSeq	0.989	1	0.69	0.844	0.726
HLA-B*27:04	HLAB	0.9872	0.9615	0.963	0.9623	0.9245
	NetMHCcons-1.1	0.982	0.962	0.992	0.977	0.954
	NetMHCstabpan-1.0	0.982	0.962	0.996	0.979	0.958
	NetMHCpan-4.1	0.981	0.962	0.989	0.975	0.95
	ACME	0.977	0.954	0.989	0.972	0.943
	Anthem	0.974	0.954	0.978	0.966	0.932
	MHCNetSeq	0.973	1	0.419	0.71	0.515
	CapsNet-MHC	0.9701	0.9615	0.963	0.9623	0.9245
	MixMHCpred-2.0.2	0.967	0.962	1	0.981	0.962
HLA-B*27:05	CapsNet-MHC	0.8769	0.7277	0.8667	0.7973	0.6003
	HLAB	0.8716	0.7705	0.8479	0.8092	0.6203
	Anthem	0.827	0.642	0.838	0.74	0.49
	MixMHCpred-2.0.2	0.773	0.689	0.709	0.699	0.398
	NetMHCstabpan-1.0	0.74	0.619	0.762	0.69	0.385
	MHCNetSeq	0.735	0.885	0.299	0.592	0.228
	NetMHCcons-1.1	0.731	0.611	0.745	0.678	0.36
	ACME	0.719	0.596	0.736	0.666	0.336
	NetMHCpan-4.1	0.712	0.596	0.745	0.67	0.344
HLA-B*27:06	MixMHCpred-2.0.2	1	1	1	1	1
	CapsNet-MHC	1	1	0.9231	0.9608	0.9245
	Anthem	0.997	1	0.977	0.988	0.978
	ACME	0.996	0.992	0.98	0.986	0.972
	NetMHCpan-4.1	0.995	0.988	0.964	0.976	0.953
	NetMHCstabpan-1.0	0.993	0.976	0.976	0.976	0.953
	NetMHCcons-1.1	0.992	0.972	0.976	0.974	0.949

	HLAB	0.9862	1	0.9231	0.9608	0.9245
	MHCNetSeq	0.977	1	0.452	0.726	0.54
HLA-B*27:07	CapsNet-MHC	1	1	1	1	1
	HLAB	1	1	1	1	1
	MixMHCpred-2.0.2	0.999	1	0.997	0.998	0.997
	NetMHCpan-4.1	0.996	0.998	0.982	0.99	0.98
	Anthem	0.993	0.987	0.98	0.983	0.967
	ACME	0.992	0.988	0.965	0.977	0.954
	NetMHCstabpan-1.0	0.991	0.981	0.968	0.975	0.95
	NetMHCcons-1.1	0.989	0.985	0.96	0.972	0.946
	MHCNetSeq	0.987	1	0.295	0.648	0.416
HLA-B*27:08	HLAB	0.9991	0.9865	0.9733	0.9799	0.9598
	MixMHCpred-2.0.2	0.995	0.985	0.986	0.986	0.972
	CapsNet-MHC	0.9946	0.9865	1	0.9933	0.9867
	Anthem	0.989	0.986	0.974	0.98	0.961
	NetMHCstabpan-1.0	0.989	0.969	0.962	0.965	0.931
	NetMHCcons-1.1	0.988	0.969	0.957	0.963	0.926
	NetMHCpan-4.1	0.987	0.974	0.961	0.968	0.935
	ACME	0.977	0.978	0.951	0.965	0.93
HLA-B*27:09	Anthem	0.9715	0.9362	0.9507	0.9435	0.887
	CapsNet-MHC	0.9682	0.9078	0.9437	0.9258	0.8521
	Anthem	0.953	0.872	0.938	0.905	0.813
	NetMHCpan-4.1	0.947	0.889	0.919	0.904	0.809
	MixMHCpred-2.0.2	0.944	0.904	0.923	0.913	0.828
	NetMHCcons-1.1	0.937	0.866	0.937	0.901	0.805
	NetMHCstabpan-1.0	0.937	0.866	0.95	0.908	0.82
	ACME	0.93	0.862	0.923	0.893	0.787
	MHCNetSeq	0.928	0.979	0.286	0.632	0.366
HLA-B*35:01	CapsNet-MHC	0.9747	0.9457	0.9154	0.9305	0.8614
	Anthem	0.956	0.851	0.939	0.895	0.794
	HLAB	0.9465	0.8605	0.9154	0.888	0.7771
	NetMHCpan-4.1	0.94	0.871	0.926	0.898	0.797
	MixMHCpred-2.0.2	0.937	0.862	0.948	0.905	0.813
	ACME	0.93	0.861	0.929	0.895	0.793
	NetMHCstabpan-1.0	0.925	0.873	0.942	0.907	0.817
	NetMHCcons-1.1	0.924	0.875	0.938	0.907	0.815
	MHCNetSeq	0.819	0.691	0.806	0.748	0.5
HLA-B*35:03	HLAB	1	1	1	1	1
	NetMHCpan-4.1	0.998	1	0.997	0.998	0.997
	CapsNet-MHC	0.9977	1	0.9333	0.9661	0.9344
	ACME	0.996	0.986	0.976	0.981	0.962
	Anthem	0.985	0.931	0.98	0.956	0.913
	NetMHCcons-1.1	0.985	0.938	0.956	0.946	0.893

	MixMHCpred-2.0.2	0.982	0.935	0.966	0.95	0.901
	NetMHCstabpan-1.0	0.982	0.928	0.948	0.938	0.876
	MHCNetSeq	0.861	0.9	0.752	0.826	0.661
HLA-B*35:08	CapsNet-MHC	0.9947	0.963	1	0.9818	0.9642
	HLAB	0.9881	0.963	1	0.9818	0.9642
	MixMHCpred-2.0.2	0.975	0.933	0.93	0.931	0.864
	Anthem	0.964	0.941	0.933	0.937	0.875
	NetMHCpan-4.1	0.949	0.926	0.996	0.961	0.924
	NetMHCcons-1.1	0.946	0.889	0.967	0.928	0.858
	NetMHCstabpan-1.0	0.945	0.889	0.963	0.926	0.854
	ACME	0.943	0.908	0.948	0.928	0.857
	MHCNetSeq	0.932	1	0.771	0.885	0.792
HLA-B*37:01	CapsNet-MHC	0.9905	1	0.9333	0.9655	0.9333
	NetMHCpan-4.1	0.988	0.993	0.979	0.986	0.973
	HLAB	0.9857	0.9286	1	0.9655	0.933
	NetMHCstabpan-1.0	0.983	0.993	0.936	0.964	0.932
	NetMHCcons-1.1	0.98	0.986	0.936	0.961	0.924
	ACME	0.968	0.929	0.972	0.95	0.902
	Anthem	0.954	0.829	0.954	0.891	0.794
	MixMHCpred-2.0.2	0.954	0.936	0.922	0.929	0.86
	MHCNetSeq	0.902	0.943	0.572	0.757	0.554
HLA-B*39:01	CapsNet-MHC	1	1	0.9714	0.9855	0.9714
	HLAB	0.9992	1	0.9714	0.9855	0.9714
	NetMHCcons-1.1	0.999	1	0.994	0.997	0.994
	NetMHCstabpan-1.0	0.999	1	0.994	0.997	0.994
	NetMHCpan-4.1	0.997	0.991	0.991	0.991	0.982
	ACME	0.997	1	0.988	0.994	0.988
	MixMHCpred-2.0.2	0.994	0.965	0.977	0.97	0.943
	Anthem	0.993	0.935	0.991	0.963	0.928
	MHCNetSeq	0.992	1	0.918	0.959	0.922
HLA-B*40:01	CapsNet-MHC	1	1	0.982	0.991	0.9821
	Anthem	0.996	0.953	0.99	0.972	0.944
	NetMHCcons-1.1	0.996	0.976	0.981	0.978	0.957
	NetMHCstabpan-1.0	0.996	0.976	0.983	0.979	0.959
	ACME	0.996	0.975	0.986	0.98	0.96
	NetMHCpan-4.1	0.995	0.981	0.975	0.978	0.956
	HLAB	0.9921	0.9909	0.982	0.9864	0.9729
	MixMHCpred-2.0.2	0.992	0.957	0.965	0.96	0.921
	MHCNetSeq	0.985	0.936	0.944	0.94	0.881
HLA-B*40:02	MixMHCpred-2.0.2	0.996	0.969	0.98	0.975	0.95
	Anthem	0.993	0.96	0.974	0.967	0.934
	HLAB	0.9926	0.9862	0.9726	0.9794	0.9589
	CapsNet-MHC	0.99	0.9724	0.9589	0.9556	0.9314

	NetMHCpan-4.1	0.99	0.983	0.972	0.977	0.955
	ACME	0.987	0.974	0.966	0.97	0.94
	NetMHCstabpan-1.0	0.986	0.972	0.972	0.972	0.945
	NetMHCcons-1.1	0.984	0.969	0.964	0.966	0.933
	MHCNetSeq	0.98	0.986	0.818	0.902	0.816
HLA-B*44:02	NetMHCpan-4.1	0.99	0.953	0.969	0.961	0.922
	CapsNet-MHC	0.9898	0.9572	0.9628	0.96	0.92
	ACME	0.988	0.961	0.953	0.957	0.914
	HLAB	0.987	0.9626	0.9734	0.968	0.9361
	NetMHCstabpan-1.0	0.984	0.951	0.974	0.963	0.926
	Anthem	0.983	0.95	0.963	0.957	0.913
	NetMHCcons-1.1	0.983	0.952	0.971	0.961	0.922
	MixMHCpred-2.0.2	0.982	0.942	0.968	0.955	0.91
	MHCNetSeq	0.977	0.963	0.826	0.894	0.796
HLA-B*44:03	HLAB	0.9975	0.9728	0.9932	0.9831	0.9663
	CapsMCH	0.9966	0.9864	0.9797	0.9831	0.9661
	NetMHCcons-1.1	0.996	0.982	0.986	0.984	0.968
	NetMHCpan-4.1	0.995	0.984	0.986	0.985	0.97
	NetMHCstabpan-1.0	0.995	0.98	0.983	0.981	0.963
	ACME	0.995	0.983	0.98	0.982	0.963
	MixMHCpred-2.0.2	0.994	0.977	0.977	0.977	0.954
	MHCNetSeq	0.99	0.973	0.882	0.927	0.858
	Anthem	0.989	0.958	0.988	0.973	0.946
HLA-B*45:01	NetMHCpan-4.1	0.984	0.958	0.974	0.966	0.933
	HLAB	0.9788	0.9677	0.9688	0.9683	0.9365
	NetMHCcons-1.1	0.977	0.925	0.948	0.937	0.875
	ACME	0.977	0.932	0.958	0.945	0.891
	MixMHCpred-2.0.2	0.974	0.935	0.897	0.916	0.834
	CapsNet-MHC	0.9718	0.9355	0.9375	0.9365	0.873
	NetMHCstabpan-1.0	0.971	0.925	0.948	0.937	0.875
	Anthem	0.946	0.858	0.967	0.912	0.831
	MHCNetSeq	0.891	0.893	0.739	0.816	0.641
HLA-B*46:01	CapsNet-MHC	0.9532	0.8889	0.9474	0.9189	0.8387
	HLAB	0.9532	0.7222	0.9474	0.8378	0.6901
	Anthem	0.909	0.645	0.95	0.797	0.626
	MixMHCpred-2.0.2	0.905	0.833	0.9	0.867	0.737
	MHCNetSeq	0.889	1	0.428	0.714	0.52
	ACME	0.864	0.767	0.894	0.831	0.67
	NetMHCstabpan-1.0	0.852	0.8	0.894	0.847	0.699
	NetMHCpan-4.1	0.848	0.8	0.889	0.845	0.693
	NetMHCcons-1.1	0.838	0.789	0.939	0.864	0.739
HLA-B*49:01	CapsNet-MHC	1	1	1	1	1
	HLAB	1	1	1	1	1

	NetMHCpan-4.1	0.992	0.996	0.983	0.989	0.979
	NetMHCstabpan-1.0	0.991	1	0.979	0.989	0.979
	NetMHCcons-1.1	0.989	0.978	0.983	0.98	0.961
	ACME	0.988	0.987	0.978	0.983	0.966
	MixMHCpred-2.0.2	0.979	0.957	0.931	0.944	0.889
	Anthem	0.966	0.879	0.932	0.905	0.813
	MHCNetSeq	0.926	1	0.439	0.72	0.529
HLA-B*51:01	CapsNet-MHC	0.9921	0.9667	0.967	0.9669	0.9337
	HLAB	0.9807	0.9444	0.967	0.9558	0.9118
	Anthem	0.956	0.874	0.929	0.901	0.804
	NetMHCpan-4.1	0.866	0.765	0.786	0.776	0.552
	NetMHCcons-1.1	0.859	0.753	0.755	0.754	0.512
	NetMHCstabpan-1.0	0.855	0.777	0.727	0.752	0.505
	ACME	0.852	0.749	0.77	0.759	0.519
	MixMHCpred-2.0.2	0.845	0.75	0.798	0.774	0.551
	MHCNetSeq	0.716	0.613	0.728	0.671	0.345
HLA-B*54:01	HLAB	0.9892	0.9333	0.9355	0.9344	0.8688
	CapsNet-MHC	0.9839	0.8333	1	0.918	0.8471
	MixMHCpred-2.0.2	0.982	0.92	0.947	0.933	0.868
	Anthem	0.96	0.853	0.928	0.891	0.786
	NetMHCstabpan-1.0	0.957	0.91	0.927	0.918	0.838
	NetMHCcons-1.1	0.955	0.893	0.927	0.91	0.822
	ACME	0.955	0.884	0.94	0.912	0.826
	NetMHCpan-4.1	0.946	0.897	0.92	0.908	0.818
	MHCNetSeq	0.879	0.967	0.61	0.788	0.617
HLA-B*56:01	CapsNet-MHC	1	1	1	1	1
	HLAB	1	0.375	1	0.697	0.4859
	MixMHCpred-2.0.2	0.999	1	0.988	0.994	0.988
	NetMHCpan-4.1	0.999	1	0.994	0.997	0.994
	NetMHCstabpan-1.0	0.999	1	0.994	0.997	0.994
	NetMHCcons-1.1	0.997	1	0.988	0.994	0.988
	ACME	0.993	0.994	0.981	0.988	0.975
	Anthem	0.991	0.9	0.984	0.942	0.89
	MHCNetSeq	0.962	1	0.55	0.775	0.617
HLA-B*57:01	CapsNet-MHC	0.9808	0.9236	0.948	0.9358	0.8719
	HLAB	0.9683	0.9184	0.9584	0.9384	0.885
	Anthem	0.966	0.92	0.964	0.942	0.885
	MixMHCpred-2.0.2	0.963	0.921	0.953	0.937	0.875
	NetMHCcons-1.1	0.96	0.908	0.946	0.927	0.854
	NetMHCstabpan-1.0	0.96	0.906	0.943	0.925	0.851
	ACME	0.958	0.911	0.94	0.926	0.852
	NetMHCpan-4.1	0.957	0.907	0.953	0.93	0.861
	MHCNetSeq	0.953	0.957	0.684	0.821	0.666

HLA-B*57:03	CapsNet-MHC	0.9908	0.982	0.9464	0.9642	0.929
	HLAB	0.986	0.9581	0.9702	0.9642	0.9284
	Anthem	0.985	0.944	0.966	0.955	0.91
	NetMHCpan-4.1	0.983	0.947	0.952	0.949	0.899
	NetMHCstabpan-1.0	0.983	0.951	0.95	0.95	0.901
	NetMHCcons-1.1	0.982	0.951	0.946	0.949	0.898
	ACME	0.976	0.938	0.937	0.938	0.875
	MHCNetSeq	0.949	0.958	0.7	0.828	0.681
HLA-B*58:01	CapsNet-MHC	0.988	0.9612	0.9808	0.971	0.9422
	HLAB	0.9814	0.9709	0.9712	0.971	0.942
	ACME	0.978	0.945	0.961	0.953	0.907
	NetMHCstabpan-1.0	0.975	0.947	0.958	0.952	0.905
	NetMHCcons-1.1	0.974	0.945	0.96	0.953	0.906
	Anthem	0.972	0.936	0.975	0.955	0.912
	NetMHCpan-4.1	0.971	0.938	0.971	0.954	0.91
	MixMHCpred-2.0.2	0.968	0.938	0.954	0.946	0.892
	MHCNetSeq	0.965	0.971	0.684	0.828	0.684
HLA-C*01:02	CapsNet-MHC	0.9535	0.8636	0.9111	0.8876	0.7759
	HLAB	0.9056	0.7045	0.9556	0.8315	0.6833
	NetMHCstabpan-1.0	0.893	0.827	0.955	0.891	0.789
	NetMHCpan-4.1	0.882	0.827	0.9	0.864	0.731
	NetMHCcons-1.1	0.879	0.839	0.911	0.875	0.753
	Anthem	0.871	0.764	0.93	0.847	0.705
	MixMHCpred-2.0.2	0.866	0.816	0.762	0.789	0.582
	MHCNetSeq	0.569	0.818	0.271	0.544	0.108
HLA-C*02:02	HLAB	0.9929	0.8462	1	0.9245	0.8585
	CapsNet-MHC	0.9915	0.9231	1	0.9623	0.927
	MixMHCpred-2.0.2	0.99	0.958	0.969	0.964	0.927
	NetMHCpan-4.1	0.982	0.95	0.927	0.939	0.879
	NetMHCcons-1.1	0.977	0.935	0.919	0.927	0.857
	NetMHCstabpan-1.0	0.974	0.943	0.904	0.923	0.849
	Anthem	0.965	0.885	0.951	0.918	0.839
HLA-C*03:03	CapsNet-MHC	0.9881	0.9	1	0.9512	0.9065
	MixMHCpred-2.0.2	0.974	0.92	0.955	0.938	0.876
	HLAB	0.9548	0.9	0.9048	0.9024	0.8048
	NetMHCcons-1.1	0.943	0.865	0.97	0.918	0.842
	NetMHCpan-4.1	0.941	0.875	0.965	0.92	0.845
	NetMHCstabpan-1.0	0.94	0.87	0.965	0.918	0.841
	Anthem	0.939	0.8	0.92	0.86	0.728
	MHCNetSeq	0.884	0.85	0.735	0.792	0.59
HLA-C*03:04	HLAB	0.9923	0.92	1	0.9608	0.9243
	CapsNet-MHC	0.9892	0.92	0.9615	0.9412	0.8829
	MixMHCpred-2.0.2	0.981	0.94	0.956	0.948	0.897

	NetMHCcons-1.1	0.979	0.912	0.96	0.936	0.874
	NetMHCstabpan-1.0	0.978	0.912	0.96	0.936	0.874
	NetMHCpan-4.1	0.971	0.912	0.92	0.916	0.834
	Anthem	0.968	0.816	0.97	0.893	0.797
	MHCNetSeq	0.874	0.84	0.732	0.786	0.577
HLA-C*04:01	CapsNet-MHC	0.8668	0.6875	0.8769	0.7829	0.5753
	HLAB	0.8353	0.8438	0.7231	0.7829	0.5707
	Anthem	0.824	0.678	0.852	0.765	0.545
	NetMHCpan-4.1	0.772	0.659	0.837	0.749	0.507
	MixMHCpred-2.0.2	0.767	0.686	0.837	0.762	0.531
	NetMHCcons-1.1	0.762	0.63	0.831	0.731	0.474
	NetMHCstabpan-1.0	0.76	0.675	0.781	0.728	0.46
	MHCNetSeq	0.695	0.938	0.231	0.585	0.235
HLA-C*05:01	HLAB	0.9768	0.95	0.9836	0.9669	0.9344
	CapsNet-MHC	0.9669	0.9167	0.9672	0.9421	0.8853
	MixMHCpred-2.0.2	0.965	0.912	0.953	0.932	0.866
	Anthem	0.964	0.88	0.946	0.913	0.829
	NetMHCpan-4.1	0.963	0.94	0.925	0.932	0.865
	NetMHCcons-1.1	0.958	0.94	0.933	0.937	0.873
	NetMHCstabpan-1.0	0.957	0.925	0.948	0.937	0.873
	MHCNetSeq	0.887	1	0.078	0.539	0.196
HLA-C*06:02	HLAB	0.8837	0.8286	0.8333	0.831	0.6619
	CapsNet-MHC	0.8778	0.8	0.8333	0.8169	0.6338
	Anthem	0.856	0.789	0.819	0.804	0.611
	NetMHCpan-4.1	0.841	0.815	0.76	0.787	0.577
	NetMHCcons-1.1	0.829	0.774	0.806	0.79	0.581
	NetMHCstabpan-1.0	0.815	0.723	0.823	0.773	0.551
	MixMHCpred-2.0.2	0.812	0.74	0.874	0.807	0.622
	MHCNetSeq	0.707	0.974	0.1	0.537	0.149
HLA-C*07:01	CapsNet-MHC	0.991	0.9268	0.9286	0.9277	0.9554
	NetMHCstabpan-1.0	0.988	0.988	0.978	0.983	0.966
	NetMHCcons-1.1	0.987	0.983	0.959	0.971	0.942
	NetMHCpan-4.1	0.972	0.954	0.932	0.943	0.886
	Anthem	0.969	0.888	0.938	0.913	0.828
	HLAB	0.9657	0.8293	1	0.9157	0.8431
	MixMHCpred-2.0.2	0.912	0.883	0.844	0.863	0.728
	MHCNetSeq	0.63	0.549	0.68	0.615	0.232
HLA-C*07:02	MixMHCpred-2.0.2	0.964	0.94	0.97	0.955	0.911
	CapsNet-MHC	0.9548	0.75	0.9048	0.8293	0.6643
	HLAB	0.9333	0.95	0.7143	0.8293	0.6807
	NetMHCpan-4.1	0.933	0.935	0.895	0.915	0.832
	NetMHCcons-1.1	0.926	0.875	0.885	0.88	0.762
	NetMHCstabpan-1.0	0.923	0.895	0.87	0.882	0.766

		Anthem	0.906	0.82	0.956	0.888	0.785
		MHCNetSeq	0.856	0.91	0.52	0.715	0.466
	HLA-C*08:02	NetMHCpan-4.1	0.994	0.97	0.987	0.978	0.957
		CapsNet-MHC	0.9903	0.9333	0.9355	0.9344	0.8688
		NetMHCcons-1.1	0.99	0.967	0.953	0.96	0.921
		HLAB	0.9892	0.9333	0.9677	0.9508	0.902
		NetMHCstabpan-1.0	0.989	0.946	0.983	0.965	0.931
		MixMHCpred-2.0.2	0.988	0.943	0.967	0.955	0.911
		Anthem	0.98	0.913	0.977	0.945	0.893
		MHCNetSeq	0.957	1	0.417	0.708	0.512
	HLA-C*16:01	CapsNet-MHC	0.9905	1	0.9333	0.9655	0.9333
		MixMHCpred-2.0.2	0.984	0.936	0.972	0.954	0.908
		NetMHCpan-4.1	0.983	0.972	0.943	0.957	0.917
		NetMHCcons-1.1	0.962	0.993	0.9	0.947	0.898
		NetMHCstabpan-1.0	0.962	1	0.9	0.95	0.905
		HLAB	0.9452	1	0.8667	0.931	0.8708
		Anthem	0.943	0.786	0.952	0.869	0.749
		MHCNetSeq	0.816	0.95	0.25	0.6	0.28
12	HLA-A*01:01	MixMHCpred-2.0.2	0.991	0.956	0.963	0.96	0.919
		NetMHCpan-4.1	0.991	0.964	0.965	0.964	0.929
		Anthem	0.99	0.943	0.974	0.959	0.918
		NetMHCcons-1.1	0.987	0.956	0.942	0.949	0.898
		NetMHCstabpan-1.0	0.987	0.939	0.951	0.945	0.891
		ACME	0.987	0.956	0.941	0.948	0.897
		CapsNet-MHC	0.9865	0.9364	0.9483	0.9424	0.8848
		HLAB	0.9768	0.9538	0.954	0.9539	0.9078
		MHCNetSeq	0.88	0.994	0.128	0.561	0.243
	HLA-A*02:01	CapsNet-MHC	0.9444	0.886	0.8821	0.884	0.7681
		Anthem	0.938	0.862	0.895	0.878	0.757
		HLAB	0.9334	0.9211	0.8646	0.8928	0.7869
		NetMHCcons-1.1	0.931	0.847	0.931	0.889	0.781
		NetMHCstabpan-1.0	0.931	0.852	0.918	0.885	0.771
		MixMHCpred-2.0.2	0.926	0.852	0.881	0.866	0.733
		ACME	0.924	0.86	0.886	0.873	0.746
		NetMHCpan-4.1	0.919	0.852	0.906	0.879	0.759
		MHCNetSeq	0.823	0.736	0.775	0.755	0.512
	HLA-A*03:01	NetMHCstabpan-1.0	0.982	0.963	0.961	0.962	0.924
		NetMHCpan-4.1	0.981	0.97	0.951	0.96	0.921
		NetMHCcons-1.1	0.981	0.961	0.963	0.962	0.924
		CapsNet-MHC	0.9778	0.9425	0.9091	0.9257	0.852
		HLAB	0.9717	0.954	0.9432	0.9486	0.8972
		ACME	0.969	0.956	0.952	0.954	0.908
		Anthem	0.963	0.904	0.943	0.923	0.847

	MixMHCpred-2.0.2	0.961	0.911	0.913	0.912	0.825
	MHCNetSeq	0.892	0.862	0.71	0.786	0.579
HLA-A*11:01	HLAB	0.9844	0.9355	0.9683	0.952	0.9044
	CapsNet-MHC	0.9757	0.9355	0.9683	0.952	0.9044
	NetMHCpan-4.1	0.968	0.93	0.958	0.945	0.889
	NetMHCstabpan-1.0	0.961	0.949	0.973	0.961	0.921
	ACME	0.96	0.933	0.96	0.947	0.894
	NetMHCcons-1.1	0.959	0.95	0.974	0.962	0.925
	Anthem	0.956	0.913	0.964	0.938	0.878
	MixMHCpred-2.0.2	0.956	0.935	0.982	0.959	0.919
	MHCNetSeq	0.941	0.968	0.352	0.66	0.404
HLA-A*24:02	CapsNet-MHC	0.9793	0.8871	0.9683	0.928	0.8587
	HLAB	0.9759	0.9194	0.9683	0.944	0.889
	MixMHCpred-2.0.2	0.969	0.949	0.96	0.954	0.908
	Anthem	0.963	0.925	0.954	0.94	0.88
	NetMHCpan-4.1	0.958	0.922	0.945	0.934	0.868
	NetMHCcons-1.1	0.955	0.938	0.927	0.933	0.866
	NetMHCstabpan-1.0	0.951	0.945	0.924	0.935	0.87
	ACME	0.951	0.901	0.937	0.919	0.84
	MHCNetSeq	0.843	0.952	0.363	0.657	0.388
HLA-A*29:02	MixMHCpred-2.0.2	0.962	0.909	0.982	0.946	0.893
	Anthem	0.956	0.903	0.937	0.92	0.842
	CapsNet-MHC	0.9554	0.9091	0.8824	0.8955	0.7914
	ACME	0.953	0.909	0.955	0.9254	0.8512
	HLAB	0.9501	0.9394	0.9118	0.954	0.908
	NetMHCstabpan-1.0	0.947	0.885	0.954	0.92	0.842
	NetMHCcons-1.1	0.946	0.879	0.979	0.928	0.862
	NetMHCpan-4.1	0.93	0.879	0.967	0.922	0.849
	MHCNetSeq	0.927	1	0.155	0.577	0.285
HLA-A*31:01	HLAB	0.9692	0.96	0.8077	0.8824	0.7749
	MixMHCpred-2.0.2	0.957	0.892	0.968	0.93	0.863
	Anthem	0.953	0.872	0.909	0.89	0.784
	NetMHCpan-4.1	0.944	0.912	0.9	0.906	0.813
	CapsNet-MHC	0.94	0.88	0.8462	0.8627	0.7262
	NetMHCcons-1.1	0.93	0.864	0.908	0.886	0.774
	NetMHCstabpan-1.0	0.929	0.872	0.9	0.886	0.774
	ACME	0.903	0.82	0.892	0.856	0.715
	MHCNetSeq	0.86	1	0.14	0.57	0.27
HLA-A*68:01	NetMHCpan-4.1	1	1	1	1	1
	MixMHCpred-2.0.2	0.998	0.994	0.988	0.991	0.982
	ACME	0.997	1	0.994	0.997	0.994
	NetMHCcons-1.1	0.996	1	0.981	0.991	0.982
	NetMHCstabpan-1.0	0.995	1	0.981	0.991	0.982

	Anthem	0.989	0.875	0.978	0.926	0.861
	HLAB	0.9706	1	0.9412	0.9697	0.9412
	CapsNet-MHC	0.9669	0.9375	0.8824	0.9091	0.8199
HLA-A*68:02	CapsNet-MHC	0.9386	0.8158	0.8974	0.8571	0.7161
	HLAB	0.9207	0.9211	0.7949	0.8571	0.7208
	Anthem	0.877	0.8	0.87	0.835	0.673
	MixMHCpred-2.0.2	0.867	0.771	0.895	0.833	0.672
	NetMHCstabpan-1.0	0.853	0.75	0.897	0.824	0.656
	NetMHCcons-1.1	0.85	0.734	0.908	0.821	0.652
	ACME	0.836	0.758	0.845	0.801	0.606
	NetMHCpan-4.1	0.831	0.74	0.887	0.813	0.635
	MHCNetSeq	0.73	1	0.145	0.572	0.273
HLA-B*07:02	CapsNet-MHC	0.9871	0.945	0.9636	0.9543	0.9088
	NetMHCpan-4.1	0.987	0.962	0.972	0.967	0.935
	ACME	0.983	0.946	0.967	0.956	0.913
	Anthem	0.982	0.936	0.957	0.947	0.894
	NetMHCcons-1.1	0.98	0.931	0.963	0.947	0.895
	NetMHCstabpan-1.0	0.98	0.93	0.959	0.945	0.89
	MixMHCpred-2.0.2	0.979	0.946	0.951	0.948	0.896
	HLAB	0.9774	0.9541	0.9636	0.9589	0.9178
	MHCNetSeq	0.849	1	0.146	0.573	0.279
HLA-B*08:01	NetMHCstabpan-1.0	0.991	1	0.987	0.993	0.987
	NetMHCcons-1.1	0.99	0.995	0.981	0.988	0.976
	NetMHCpan-4.1	0.987	0.976	0.959	0.967	0.935
	CapsNet-MHC	0.9858	1	0.9211	0.96	0.923
	HLAB	0.9708	0.8919	0.9737	0.9333	0.8693
	ACME	0.968	0.924	0.914	0.919	0.839
	Anthem	0.957	0.8	0.956	0.878	0.767
	MixMHCpred-2.0.2	0.943	0.924	0.865	0.895	0.791
	MHCNetSeq	0.56	0.757	0.332	0.545	0.097
HLA-B*15:01	CapsNet-MHC	0.9744	0.8701	0.9615	0.9161	0.8356
	HLAB	0.9599	0.8701	0.9103	0.8903	0.7812
	MixMHCpred-2.0.2	0.94	0.867	0.919	0.894	0.789
	NetMHCstabpan-1.0	0.93	0.856	0.914	0.885	0.772
	Anthem	0.925	0.841	0.941	0.891	0.787
	NetMHCcons-1.1	0.925	0.836	0.928	0.883	0.769
	ACME	0.924	0.869	0.925	0.897	0.795
	NetMHCpan-4.1	0.915	0.847	0.922	0.885	0.772
	MHCNetSeq	0.878	1	0.047	0.523	0.15
HLA-B*27:01	CapsNet-MHC	1	1	1	1	1
	HLAB	1	1	1	1	1
	MixMHCpred-2.0.2	0.998	0.992	0.979	0.986	0.971
	NetMHCpan-4.1	0.998	1	0.989	0.995	0.99

	ACME	0.996	1	0.984	0.992	0.985
	NetMHCcons-1.1	0.99	0.973	0.969	0.971	0.942
	NetMHCstabpan-1.0	0.989	0.966	0.974	0.97	0.94
	Anthem	0.975	0.921	0.976	0.949	0.899
HLA-B*27:02	MixMHCpred-2.0.2	0.996	0.969	0.975	0.972	0.944
	CapsNet-MHC	0.9956	0.9492	0.9833	0.9664	0.9333
	NetMHCpan-4.1	0.993	0.968	0.978	0.973	0.946
	HLAB	0.9917	1	0.95	0.9748	0.9508
	Anthem	0.984	0.922	0.98	0.951	0.903
	NetMHCstabpan-1.0	0.984	0.934	0.973	0.954	0.908
	NetMHCcons-1.1	0.981	0.94	0.951	0.946	0.892
	ACME	0.968	0.94	0.944	0.942	0.885
HLA-B*27:03	CapsNet-MHC	1	1	1	1	1
	HLAB	1	1	1	1	1
	MixMHCpred-2.0.2	0.999	1	0.987	0.993	0.987
	NetMHCstabpan-1.0	0.999	1	0.993	0.997	0.994
	NetMHCcons-1.1	0.998	1	0.987	0.993	0.987
	NetMHCpan-4.1	0.997	1	0.993	0.997	0.994
	ACME	0.994	1	0.987	0.993	0.987
	Anthem	0.966	0.947	0.973	0.96	0.926
	MHCNetSeq	0.919	1	0.247	0.623	0.369
HLA-B*27:05	CapsNet-MHC	0.8423	0.734	0.7931	0.7635	0.5279
	Anthem	0.821	0.689	0.787	0.738	0.479
	HLAB	0.8016	0.877	0.6088	0.7428	0.5042
	MixMHCpred-2.0.2	0.735	0.606	0.74	0.673	0.349
	NetMHCstabpan-1.0	0.684	0.57	0.695	0.633	0.268
	NetMHCcons-1.1	0.682	0.592	0.667	0.629	0.259
	NetMHCpan-4.1	0.645	0.502	0.729	0.616	0.239
	ACME	0.642	0.492	0.751	0.622	0.252
	MHCNetSeq	0.642	0.971	0.064	0.517	0.081
HLA-B*27:07	CapsNet-MHC	1	1	1	1	1
	MixMHCpred-2.0.2	0.997	0.979	0.987	0.983	0.967
	HLAB	0.9833	0.9583	0.96	0.9592	0.9183
	NetMHCpan-4.1	0.986	0.962	0.967	0.964	0.93
	NetMHCstabpan-1.0	0.983	0.962	0.958	0.96	0.922
	NetMHCcons-1.1	0.981	0.962	0.958	0.96	0.922
	Anthem	0.968	0.958	0.965	0.962	0.924
	ACME	0.968	0.942	0.95	0.946	0.893
HLA-B*27:08	MixMHCpred-2.0.2	0.996	0.988	0.975	0.981	0.963
	CapsNet-MHC	0.9877	0.9062	0.9394	0.9231	0.8464
	NetMHCpan-4.1	0.983	0.963	0.969	0.965	0.932
	NetMHCstabpan-1.0	0.982	0.938	0.953	0.945	0.891
	Anthem	0.981	0.888	0.985	0.936	0.879

	NetMHCcons-1.1	0.981	0.941	0.966	0.953	0.907
	ACME	0.97	0.95	0.925	0.938	0.876
	HLAB	0.9328	0.9063	0.9394	0.9231	0.8464
HLA-B*27:09	HLAB	0.9474	0.9016	0.871	0.8862	0.7728
	CapsNet-MHC	0.9371	0.8197	0.9677	0.8943	0.797
	MixMHCpred-2.0.2	0.897	0.793	0.979	0.886	0.787
	Anthem	0.895	0.79	0.948	0.869	0.748
	NetMHCstabpan-1.0	0.869	0.798	0.948	0.873	0.755
	NetMHCcons-1.1	0.868	0.8	0.953	0.876	0.762
	ACME	0.864	0.797	0.942	0.87	0.748
	NetMHCpan-4.1	0.846	0.787	0.969	0.878	0.769
	MHCNetSeq	0.806	1	0.076	0.538	0.196
HLA-B*35:01	HLAB	0.9437	0.8333	0.8189	0.8767	0.7557
	Anthem	0.909	0.822	0.875	0.849	0.701
	CapsNet-MHC	0.9002	0.8889	0.8378	0.863	0.7273
	MixMHCpred-2.0.2	0.866	0.756	0.897	0.826	0.66
	ACME	0.824	0.761	0.847	0.804	0.613
	NetMHCstabpan-1.0	0.794	0.711	0.858	0.785	0.577
	NetMHCcons-1.1	0.792	0.722	0.881	0.802	0.612
	NetMHCpan-4.1	0.791	0.68	0.889	0.785	0.583
	MHCNetSeq	0.583	0.716	0.431	0.573	0.157
HLA-B*40:01	CapsNet-MHC	1	1	1	1	1
	HLAB	1	1	1	1	1
	NetMHCcons-1.1	0.998	0.997	0.994	0.995	0.99
	NetMHCstabpan-1.0	0.998	0.997	0.994	0.995	0.99
	ACME	0.998	1	0.997	0.998	0.997
	Anthem	0.997	1	0.983	0.991	0.983
	NetMHCpan-4.1	0.997	1	0.99	0.995	0.99
	MixMHCpred-2.0.2	0.995	0.971	0.974	0.973	0.946
	MHCNetSeq	0.973	1	0.565	0.782	0.628
HLA-B*40:02	MixMHCpred-2.0.2	0.997	0.973	0.99	0.982	0.964
	HLAB	0.993	0.9512	0.9762	0.9639	0.9279
	NetMHCcons-1.1	0.988	0.971	0.971	0.971	0.941
	CapsNet-MHC	0.9787	0.9756	0.9524	0.9639	0.928
	NetMHCstabpan-1.0	0.986	0.968	0.971	0.97	0.939
	Anthem	0.982	0.932	0.979	0.955	0.912
	NetMHCpan-4.1	0.979	0.963	0.983	0.973	0.947
	ACME	0.977	0.971	0.976	0.973	0.946
	MHCNetSeq	0.89	1	0.183	0.591	0.315
HLA-B*44:02	NetMHCpan-4.1	0.997	0.979	0.988	0.983	0.966
	HLAB	0.9925	0.9385	0.9848	0.9618	0.9246
	MixMHCpred-2.0.2	0.981	0.937	0.946	0.941	0.883
	ACME	0.981	0.92	0.963	0.942	0.884

	NetMHCcons-1.1	0.98	0.898	0.98	0.939	0.882
	NetMHCstabpan-1.0	0.978	0.898	0.98	0.939	0.882
	Anthem	0.974	0.895	0.979	0.937	0.878
	CapsNet-MHC	0.9725	0.9077	0.9545	0.9313	0.8634
	MHCNetSeq	0.897	1	0.071	0.535	0.186
HLA-B*44:03	HLAB	0.9979	0.9535	1	0.977	0.955
	CapsNet-MHC	0.9894	0.9535	1	0.977	0.955
	ACME	0.989	0.953	0.984	0.969	0.938
	NetMHCcons-1.1	0.987	0.963	0.981	0.972	0.945
	MixMHCpred-2.0.2	0.986	0.958	0.993	0.976	0.952
	NetMHCstabpan-1.0	0.986	0.958	0.974	0.966	0.933
	NetMHCpan-4.1	0.982	0.953	0.986	0.97	0.94
	Anthem	0.969	0.897	0.992	0.945	0.894
	MHCNetSeq	0.946	1	0.151	0.576	0.282
HLA-B*51:01	CapsNet-MHC	0.9317	0.831	0.875	0.8531	0.7068
	Anthem	0.916	0.792	0.919	0.855	0.717
	HLAB	0.8987	0.8732	0.8056	0.8392	0.6801
	NetMHCcons-1.1	0.886	0.774	0.922	0.848	0.704
	NetMHCstabpan-1.0	0.88	0.765	0.905	0.835	0.677
	NetMHCpan-4.1	0.875	0.774	0.876	0.825	0.653
	MixMHCpred-2.0.2	0.873	0.797	0.835	0.816	0.633
	ACME	0.866	0.806	0.813	0.809	0.619
	MHCNetSeq	0.576	0.795	0.268	0.531	0.072
HLA-B*57:01	CapsNet-MHC	0.9571	0.8927	0.9172	0.905	0.8102
	Anthem	0.949	0.855	0.956	0.905	0.815
	HLAB	0.9399	0.9066	0.9172	0.9119	0.8239
	MixMHCpred-2.0.2	0.936	0.867	0.965	0.916	0.835
	NetMHCstabpan-1.0	0.936	0.852	0.952	0.902	0.808
	NetMHCpan-4.1	0.934	0.885	0.943	0.914	0.83
	NetMHCcons-1.1	0.934	0.857	0.946	0.901	0.806
	ACME	0.931	0.869	0.946	0.908	0.818
	MHCNetSeq	0.856	0.993	0.055	0.524	0.137
HLA-B*57:03	HLAB	0.9722	0.9167	0.9459	0.9315	0.8633
	CapsNet-MHC	0.961	0.8611	1	0.9315	0.871
	Anthem	0.933	0.828	0.94	0.884	0.775
	NetMHCpan-4.1	0.925	0.881	0.939	0.91	0.821
	NetMHCcons-1.1	0.917	0.883	0.941	0.913	0.827
	NetMHCstabpan-1.0	0.917	0.886	0.93	0.909	0.818
	ACME	0.912	0.884	0.911	0.897	0.797
	MHCNetSeq	0.835	1	0.07	0.535	0.185
HLA-B*58:01	CapsNet-MHC	0.9825	0.9143	0.9444	0.9296	0.8594
	HLAB	0.981	0.9143	0.9444	0.9296	0.8594
	NetMHCcons-1.1	0.959	0.917	0.968	0.943	0.887

		NetMHCstabpan-1.0	0.959	0.923	0.957	0.94	0.881
		ACME	0.957	0.94	0.946	0.943	0.886
		NetMHCpan-4.1	0.954	0.908	0.96	0.934	0.87
		MixMHCpred-2.0.2	0.946	0.908	0.948	0.929	0.858
		Anthem	0.93	0.835	0.93	0.882	0.768
	HLA-C*01:02	CapsNet-MHC	0.9495	0.9268	0.881	0.9036	0.8083
		HLAB	0.9437	0.9512	0.8095	0.8795	0.7675
		NetMHCcons-1.1	0.935	0.892	0.924	0.908	0.818
		NetMHCpan-4.1	0.932	0.912	0.897	0.905	0.81
		NetMHCstabpan-1.0	0.929	0.902	0.978	0.94	0.883
		Anthem	0.928	0.849	0.92	0.885	0.773
		MixMHCpred-2.0.2	0.785	0.793	0.715	0.754	0.51
		MHCNetSeq	0.532	0.761	0.239	0.5	-0.005
	HLA-C*04:01	CapsNet-MHC	0.8	0.56	0.8846	0.7255	0.4715
		HLAB	0.76	0.68	0.7308	0.7059	0.4114
		Anthem	0.726	0.608	0.714	0.661	0.331
		NetMHCpan-4.1	0.614	0.5	0.724	0.612	0.239
		NetMHCstabpan-1.0	0.608	0.464	0.788	0.626	0.271
		NetMHCcons-1.1	0.606	0.576	0.672	0.624	0.253
		MixMHCpred-2.0.2	0.573	0.492	0.716	0.604	0.224
	HLA-C*05:01	HLAB	0.9786	1	0.9048	0.9512	0.9069
		Anthem	0.942	0.89	0.864	0.877	0.757
		MixMHCpred-2.0.2	0.934	0.855	0.94	0.897	0.799
		CapsNet-MHC	0.9262	0.8	0.8095	0.8049	0.6095
		NetMHCpan-4.1	0.876	0.82	0.875	0.847	0.698
		NetMHCstabpan-1.0	0.855	0.79	0.855	0.823	0.648
		NetMHCcons-1.1	0.85	0.775	0.88	0.828	0.662
	HLA-C*06:02	HLAB	0.9074	0.9615	0.6667	0.8113	0.6549
		Anthem	0.764	0.492	0.915	0.704	0.453
		CapsNet-MHC	0.7578	0.6154	0.6296	0.6228	0.245
		MixMHCpred-2.0.2	0.647	0.588	0.65	0.619	0.242
		NetMHCcons-1.1	0.557	0.443	0.754	0.598	0.215
		NetMHCpan-4.1	0.547	0.465	0.688	0.577	0.171
		NetMHCstabpan-1.0	0.521	0.454	0.631	0.542	0.087
	HLA-C*07:01	NetMHCstabpan-1.0	0.994	1	0.978	0.989	0.978
		CapsNet-MHC	0.9925	0.9444	0.9459	0.9452	0.8904
		NetMHCcons-1.1	0.992	0.997	0.969	0.983	0.968
		HLAB	0.985	0.9167	0.973	0.9452	0.8917
		NetMHCpan-4.1	0.981	0.942	0.939	0.94	0.881
		Anthem	0.948	0.811	0.938	0.875	0.757
		MixMHCpred-2.0.2	0.885	0.85	0.792	0.821	0.645
		MHCNetSeq	0.502	0.672	0.308	0.49	-0.024
13	HLA-A*01:01	CapsNet-MHC	0.9987	0.9818	0.982	0.9819	0.9638

	MixMHCpred-2.0.2	0.998	0.986	0.988	0.987	0.974
	Anthem	0.997	0.987	0.972	0.979	0.959
	NetMHCcons-1.1	0.995	0.968	0.977	0.972	0.945
	NetMHCstabpan-1.0	0.995	0.968	0.971	0.97	0.939
	ACME	0.994	0.979	0.976	0.977	0.955
	NetMHCpan-4.1	0.993	0.962	0.979	0.971	0.941
	HLAB	0.9768	0.9538	0.954	0.9539	0.9078
HLA-A*02:01	CapsNet-MHC	0.9547	0.8632	0.9583	0.9119	0.8256
	HLAB	0.9334	0.9211	0.8646	0.8928	0.7869
	Anthem	0.917	0.802	0.894	0.848	0.7
	MixMHCpred-2.0.2	0.9	0.798	0.939	0.868	0.746
	NetMHCpan-4.1	0.894	0.798	0.915	0.856	0.718
	ACME	0.886	0.787	0.911	0.849	0.705
	NetMHCstabpan-1.0	0.883	0.762	0.905	0.834	0.675
	NetMHCcons-1.1	0.881	0.763	0.905	0.834	0.676
	MHCNetSeq	0.84	0.758	0.742	0.75	0.5
HLA-A*03:01	NetMHCpan-4.1	0.989	0.966	0.961	0.964	0.929
	ACME	0.98	0.944	0.978	0.961	0.923
	NetMHCcons-1.1	0.977	0.916	0.966	0.941	0.885
	NetMHCstabpan-1.0	0.975	0.916	0.972	0.944	0.891
	HLAB	0.9717	0.954	0.9432	0.9486	0.8972
	CapsNet-MHC	0.9708	0.9444	0.7895	0.8649	0.7404
	MixMHCpred-2.0.2	0.95	0.938	0.978	0.958	0.918
	Anthem	0.94	0.889	0.919	0.904	0.812
	MHCNetSeq	0.898	0.944	0.7	0.822	0.668
HLA-A*11:01	HLAB	0.9844	0.9355	0.9683	0.952	0.9044
	CapsNet-MHC	0.9708	0.8889	0.8947	0.8919	0.7836
	NetMHCstabpan-1.0	0.926	0.833	0.9	0.867	0.737
	MixMHCpred-2.0.2	0.925	0.811	0.9	0.855	0.719
	NetMHCcons-1.1	0.923	0.85	0.878	0.864	0.731
	ACME	0.91	0.845	0.861	0.853	0.708
	NetMHCpan-4.1	0.894	0.795	0.933	0.864	0.738
	Anthem	0.888	0.744	0.9	0.822	0.655
HLA-A*24:02	MixMHCpred-2.0.2	0.994	0.967	0.969	0.968	0.936
	CapsNet-MHC	0.9878	0.8718	0.925	0.8987	0.7983
	Anthem	0.98	0.897	0.951	0.924	0.851
	NetMHCpan-4.1	0.979	0.936	0.951	0.944	0.887
	NetMHCstabpan-1.0	0.979	0.931	0.931	0.931	0.862
	NetMHCcons-1.1	0.978	0.928	0.933	0.931	0.862
	HLAB	0.9759	0.9194	0.9683	0.944	0.889
	ACME	0.942	0.872	0.915	0.894	0.789
HLA-A*29:02	MixMHCpred-2.0.2	0.985	0.937	0.971	0.954	0.909
	CapsNet-MHC	0.9804	0.9211	0.8974	0.9091	0.8185

	NetMHCcons-1.1	0.965	0.921	0.953	0.937	0.876
	NetMHCstabpan-1.0	0.964	0.931	0.934	0.933	0.867
	NetMHCpan-4.1	0.959	0.926	0.924	0.925	0.85
	ACME	0.955	0.895	0.937	0.916	0.833
	Anthem	0.952	0.863	0.98	0.921	0.85
	HLAB	0.9501	0.9394	0.9118	0.9254	0.8512
HLA-A*31:01	HLAB	0.9692	0.96	0.8077	0.8824	0.7749
	MixMHCpred-2.0.2	0.894	0.85	0.9	0.875	0.754
	Anthem	0.889	0.8	0.894	0.847	0.701
	CapsNet-MHC	0.881	0.7143	0.7333	0.7241	0.4476
	NetMHCstabpan-1.0	0.839	0.786	0.793	0.789	0.58
	NetMHCcons-1.1	0.837	0.786	0.821	0.803	0.61
	NetMHCpan-4.1	0.822	0.779	0.879	0.829	0.663
	ACME	0.789	0.771	0.743	0.757	0.521
HLA-A*68:02	HLAB	0.9207	0.9211	0.7949	0.8571	0.7208
	CapsNet-MHC	0.8315	0.6957	0.9167	0.8085	0.6297
	Anthem	0.692	0.417	0.887	0.652	0.35
	MixMHCpred-2.0.2	0.595	0.483	0.752	0.617	0.25
	ACME	0.559	0.474	0.691	0.582	0.171
	NetMHCstabpan-1.0	0.55	0.487	0.704	0.596	0.198
	NetMHCcons-1.1	0.532	0.504	0.67	0.587	0.178
	NetMHCpan-4.1	0.515	0.417	0.765	0.591	0.208
HLA-B*07:02	MixMHCpred-2.0.2	0.991	0.959	0.959	0.96	0.919
	CapsNet-MHC	0.9899	0.9219	0.9538	0.938	0.8764
	NetMHCpan-4.1	0.987	0.946	0.956	0.951	0.902
	ACME	0.985	0.947	0.958	0.952	0.905
	NetMHCcons-1.1	0.984	0.958	0.93	0.944	0.888
	NetMHCstabpan-1.0	0.984	0.955	0.931	0.943	0.886
	Anthem	0.981	0.897	0.969	0.933	0.869
	HLAB	0.9774	0.9541	0.9636	0.9589	0.9178
HLA-B*15:01	HLAB	0.9599	0.8701	0.9103	0.8903	0.7812
	CapsNet-MHC	0.9446	0.8431	0.9038	0.8738	0.7487
	Anthem	0.941	0.878	0.949	0.914	0.83
	MixMHCpred-2.0.2	0.938	0.898	0.955	0.927	0.855
	NetMHCpan-4.1	0.922	0.882	0.951	0.917	0.836
	NetMHCcons-1.1	0.92	0.859	0.951	0.905	0.814
	NetMHCstabpan-1.0	0.918	0.87	0.935	0.903	0.808
	ACME	0.905	0.863	0.939	0.901	0.805
	MHCNetSeq	0.802	1	0.065	0.532	0.18
HLA-B*27:01	HLAB	1	1	1	1	1
	MixMHCpred-2.0.2	0.987	0.955	0.994	0.975	0.951
	CapsNet-MHC	0.9825	0.9444	0.9474	0.9459	0.8918
	NetMHCpan-4.1	0.978	0.944	0.989	0.966	0.935

	ACME	0.977	0.966	0.944	0.955	0.913
	NetMHCcons-1.1	0.956	0.938	0.961	0.95	0.901
	NetMHCstabpan-1.0	0.955	0.938	0.955	0.947	0.896
	Anthem	0.943	0.822	0.982	0.902	0.817
HLA-B*27:02	CapsNet-MHC	1	1	1	1	1
	MixMHCpred-2.0.2	1	1	1	1	1
	NetMHCpan-4.1	0.995	0.993	0.978	0.985	0.971
	NetMHCcons-1.1	0.995	0.974	0.974	0.974	0.949
	NetMHCstabpan-1.0	0.995	0.97	0.97	0.97	0.941
	HLAB	0.9917	1	0.95	0.9748	0.9508
	ACME	0.991	0.989	0.967	0.978	0.956
	Anthem	0.99	0.904	0.965	0.934	0.871
HLA-B*27:05	CapsNet-MHC	0.8315	0.7549	0.7396	0.7473	0.4946
	Anthem	0.811	0.699	0.767	0.732	0.466
	HLAB	0.8016	0.877	0.6088	0.7428	0.5042
	MixMHCpred-2.0.2	0.695	0.596	0.681	0.638	0.279
	NetMHCstabpan-1.0	0.623	0.519	0.655	0.587	0.175
	NetMHCcons-1.1	0.619	0.517	0.645	0.581	0.164
	MHCNetSeq	0.611	0.96	0.064	0.513	0.055
	NetMHCpan-4.1	0.595	0.471	0.671	0.571	0.147
	ACME	0.583	0.502	0.63	0.566	0.135
HLA-B*27:08	CapsNet-MHC	1	1	1	1	1
	NetMHCpan-4.1	1	1	1	1	1
	NetMHCcons-1.1	1	1	0.993	0.997	0.994
	NetMHCstabpan-1.0	1	1	0.993	0.997	0.994
	ACME	0.996	1	0.987	0.993	0.987
	MixMHCpred-2.0.2	0.994	1	0.993	0.997	0.994
	Anthem	0.993	0.907	0.976	0.941	0.885
	HLAB	0.9328	0.9063	0.939	0.9231	0.8464
HLA-B*27:09	HLAB	0.9474	0.9016	0.871	0.8862	0.7728
	CapsNet-MHC	0.9114	0.75	0.9189	0.8356	0.6797
	Anthem	0.76	0.606	0.851	0.728	0.476
	MixMHCpred-2.0.2	0.679	0.584	0.85	0.716	0.453
	NetMHCcons-1.1	0.642	0.556	0.886	0.721	0.469
	NetMHCstabpan-1.0	0.642	0.556	0.892	0.723	0.476
	NetMHCpan-4.1	0.627	0.55	0.867	0.708	0.441
	ACME	0.593	0.514	0.842	0.678	0.378
HLA-B*35:01	HLAB	0.9437	0.8333	0.9189	0.8767	0.7557
	CapsNet-MHC	0.9412	0.9412	0.7222	0.8286	0.6768
	MixMHCpred-2.0.2	0.851	0.783	0.788	0.785	0.575
	Anthem	0.837	0.635	0.893	0.764	0.551
	MHCNetSeq	0.757	0.947	0.341	0.644	0.356
	NetMHCcons-1.1	0.701	0.677	0.688	0.682	0.372

	ACME	0.69	0.647	0.688	0.667	0.345
	NetMHCstabpan-1.0	0.681	0.623	0.706	0.665	0.338
	NetMHCpan-4.1	0.576	0.588	0.606	0.597	0.205
HLA-B*44:02	HLAB	0.9925	0.9385	0.9848	0.9618	0.9246
	Anthem	0.963	0.943	0.95	0.947	0.895
	NetMHCpan-4.1	0.955	0.952	0.986	0.969	0.939
	ACME	0.954	0.952	0.99	0.971	0.943
	MixMHCpred-2.0.2	0.953	0.952	0.995	0.974	0.948
	NetMHCcons-1.1	0.953	0.952	0.981	0.966	0.934
	NetMHCstabpan-1.0	0.953	0.952	0.976	0.964	0.929
	CapsNet-MHC	0.9502	0.9048	1	0.9535	0.9107
HLA-B*51:01	CapsNet-MHC	0.9571	0.8	0.9524	0.878	0.7634
	HLAB	0.8987	0.8732	0.8056	0.8392	0.6801
	MixMHCpred-2.0.2	0.859	0.775	0.835	0.805	0.615
	NetMHCcons-1.1	0.845	0.82	0.815	0.818	0.637
	NetMHCpan-4.1	0.838	0.775	0.855	0.815	0.633
	NetMHCstabpan-1.0	0.836	0.775	0.86	0.818	0.641
	Anthem	0.825	0.62	0.861	0.74	0.501
	ACME	0.77	0.77	0.7	0.735	0.476
HLA-B*57:01	CapsNet-MHC	0.9538	0.8827	0.9018	0.8923	0.7847
	HLAB	0.9399	0.9066	0.9172	0.9119	0.8239
	Anthem	0.903	0.714	0.948	0.831	0.682
	MixMHCpred-2.0.2	0.882	0.783	0.87	0.826	0.656
	NetMHCstabpan-1.0	0.873	0.754	0.831	0.793	0.591
	NetMHCcons-1.1	0.864	0.709	0.892	0.801	0.613
	ACME	0.862	0.731	0.878	0.804	0.617
	NetMHCpan-4.1	0.855	0.733	0.879	0.806	0.618
HLA-B*57:03	HLAB	0.9722	0.9167	0.9459	0.9315	0.8633
	Anthem	0.956	0.8	0.958	0.879	0.77
	CapsNet-MHC	0.9452	0.9	0.7619	0.8293	0.6667
	NetMHCstabpan-1.0	0.93	0.85	1	0.925	0.86
	NetMHCcons-1.1	0.929	0.855	0.99	0.923	0.854
	NetMHCpan-4.1	0.91	0.85	1	0.925	0.86
	ACME	0.896	0.85	0.985	0.918	0.844
HLA-B*58:01	HLAB	0.981	0.9143	0.9444	0.9296	0.8594
	CapsNet-MHC	0.9181	0.5556	0.9474	0.7568	0.55
	ACME	0.841	0.744	0.861	0.803	0.614
	NetMHCstabpan-1.0	0.837	0.7	0.878	0.789	0.591
	NetMHCcons-1.1	0.836	0.711	0.855	0.784	0.575
	NetMHCpan-4.1	0.828	0.7	0.861	0.781	0.576
	Anthem	0.778	0.545	0.95	0.747	0.543
	MixMHCpred-2.0.2	0.751	0.639	0.889	0.764	0.547
HLA-C*04:01	HLAB	0.76	0.68	0.7308	0.7059	0.4114

		Anthem	0.746	0.564	0.842	0.703	0.43
		CapsNet-MHC	0.583	0.5455	0.5217	0.5333	0.0672
		MixMHCpred-2.0.2	0.579	0.627	0.586	0.607	0.218
		NetMHCstabpan-1.0	0.554	0.405	0.609	0.507	0.015
		NetMHCpan-4.1	0.545	0.35	0.714	0.532	0.078
		NetMHCcons-1.1	0.521	0.464	0.586	0.525	0.051
	HLA-C*05:01	HLAB	0.9786	1	0.9048	0.9512	0.9069
		CapsNet-MHC	0.9125	0.8667	0.875	0.871	0.7417
		MixMHCpred-2.0.2	0.838	0.773	0.887	0.83	0.666
		Anthem	0.776	0.493	0.823	0.658	0.344
		NetMHCcons-1.1	0.776	0.807	0.727	0.767	0.542
		NetMHCpan-4.1	0.772	0.72	0.74	0.73	0.462
		NetMHCstabpan-1.0	0.761	0.78	0.7	0.74	0.484
	HLA-C*06:02	HLAB	0.9074	0.9615	0.6667	0.8113	0.6549
		CapsNet-MHC	0.786	0.8333	0.5806	0.7049	0.427
		Anthem	0.618	0.34	0.81	0.575	0.175
		NetMHCstabpan-1.0	0.59	0.577	0.62	0.598	0.198
		NetMHCcons-1.1	0.571	0.54	0.673	0.607	0.217
		NetMHCpan-4.1	0.565	0.637	0.507	0.572	0.145
		MixMHCpred-2.0.2	0.521	0.5	0.627	0.563	0.134
14	HLA-A*01:01	HLAB	0.9988	0.9727	0.991	0.9819	0.964
		MixMHCpred-2.0.2	0.995	0.98	0.984	0.982	0.965
		NetMHCpan-4.1	0.994	0.98	0.98	0.98	0.961
		NetMHCcons-1.1	0.993	0.967	0.98	0.974	0.947
		ACME	0.993	0.968	0.974	0.972	0.943
		NetMHCstabpan-1.0	0.992	0.969	0.972	0.971	0.941
		CapsNet-MHC	0.986	0.9608	0.9615	0.9612	0.9223
		Anthem	0.986	0.937	0.974	0.956	0.913
	HLA-A*02:01	HLAB	0.9567	0.9053	0.9167	0.911	0.822
		CapsNet-MHC	0.937	0.8824	0.8654	0.8737	0.7477
		MixMHCpred-2.0.2	0.935	0.843	0.918	0.88	0.764
		NetMHCcons-1.1	0.924	0.845	0.892	0.869	0.739
		NetMHCstabpan-1.0	0.923	0.839	0.894	0.866	0.735
		Anthem	0.91	0.8	0.899	0.85	0.705
		NetMHCpan-4.1	0.906	0.812	0.9	0.856	0.715
		ACME	0.898	0.816	0.857	0.836	0.674
		MHCNetSeq	0.866	0.82	0.767	0.793	0.589
	HLA-A*24:02	CapsNet-MHC	1	0.875	1	0.9394	0.8848
		HLAB	0.9872	0.9744	0.8	0.8861	0.785
		NetMHCcons-1.1	0.986	0.981	0.956	0.969	0.939
		MixMHCpred-2.0.2	0.982	0.925	0.981	0.953	0.908
		NetMHCstabpan-1.0	0.982	0.975	0.944	0.96	0.919
		NetMHCpan-4.1	0.98	0.994	0.925	0.96	0.922

	Anthem	0.94	0.9	0.938	0.919	0.841
	ACME	0.927	0.881	0.913	0.897	0.794
HLA-A*68:02	HLAB	0.8913	0.8261	0.6667	0.7447	0.4982
	CapsNet-MHC	0.8392	0.7778	0.7895	0.7838	0.5673
	Anthem	0.778	0.511	0.872	0.692	0.418
	ACME	0.71	0.695	0.695	0.695	0.394
	NetMHCstabpan-1.0	0.665	0.628	0.628	0.628	0.259
	NetMHCpan-4.1	0.654	0.656	0.617	0.636	0.281
	NetMHCcons-1.1	0.626	0.6	0.65	0.625	0.258
	MixMHCpred-2.0.2	0.62	0.633	0.6	0.617	0.237
HLA-B*07:02	HLAB	0.9964	0.8906	0.9846	0.938	0.8797
	CapsNet-MHC	0.9841	0.8857	0.9722	0.9296	0.8621
	NetMHCcons-1.1	0.976	0.963	0.943	0.953	0.906
	ACME	0.975	0.945	0.948	0.947	0.895
	NetMHCpan-4.1	0.973	0.971	0.971	0.971	0.943
	NetMHCstabpan-1.0	0.973	0.963	0.937	0.95	0.901
	MixMHCpred-2.0.2	0.968	0.928	0.951	0.94	0.881
	Anthem	0.943	0.852	0.954	0.903	0.811
HLA-B*15:01	MixMHCpred-2.0.2	0.95	0.919	0.922	0.92	0.841
	CapsNet-MHC	0.9413	0.9062	0.8485	0.8769	0.7555
	NetMHCcons-1.1	0.937	0.878	0.928	0.903	0.808
	NetMHCstabpan-1.0	0.937	0.872	0.91	0.891	0.782
	Anthem	0.936	0.85	0.943	0.897	0.798
	ACME	0.934	0.888	0.878	0.883	0.768
	NetMHCpan-4.1	0.933	0.894	0.922	0.908	0.816
	HLAB	0.9123	0.8824	0.8654	0.8738	0.7477
	MHCNetSeq	0.855	0.969	0.384	0.677	0.432
HLA-B*27:05	Anthem	0.824	0.753	0.756	0.754	0.509
	CapsNet-MHC	0.8231	0.8105	0.685	0.7477	0.4995
	HLAB	0.8225	0.7431	0.7416	0.7423	0.4847
	MixMHCpred-2.0.2	0.66	0.571	0.684	0.627	0.257
	NetMHCstabpan-1.0	0.533	0.486	0.587	0.536	0.073
	NetMHCcons-1.1	0.529	0.478	0.595	0.536	0.073
	MHCNetSeq	0.523	0.953	0.053	0.503	0.013
	NetMHCpan-4.1	0.52	0.456	0.532	0.494	-0.012
	ACME	0.518	0.433	0.569	0.501	0.002
HLA-B*27:09	HLAB	0.9182	0.8333	0.7838	0.8082	0.6176
	CapsNet-MHC	0.8954	0.72	0.9615	0.8431	0.7074
	Anthem	0.838	0.672	0.901	0.786	0.591
	MixMHCpred-2.0.2	0.76	0.68	0.808	0.744	0.497
	NetMHCcons-1.1	0.614	0.604	0.632	0.618	0.24
	NetMHCstabpan-1.0	0.613	0.616	0.624	0.62	0.245
	NetMHCpan-4.1	0.55	0.46	0.732	0.596	0.203

	ACME	0.549	0.556	0.588	0.572	0.145
HLA-B*35:01	CapsNet-MHC	1	1	1	1	1
	HLAB	0.9346	0.9412	0.7778	0.8571	0.7261
	Anthem	0.831	0.533	0.887	0.71	0.457
	MHCNetSeq	0.746	0.966	0.3	0.633	0.36
	MixMHCpred-2.0.2	0.586	0.773	0.533	0.653	0.319
	NetMHCstabpan-1.0	0.526	0.653	0.533	0.593	0.195
	ACME	0.524	0.44	0.78	0.61	0.238
	NetMHCpan-4.1	0.521	0.62	0.5	0.56	0.123
	NetMHCcons-1.1	0.502	0.76	0.447	0.603	0.225
HLA-B*57:01	HLAB	0.981	0.9	0.9524	0.9268	0.8544
	CapsNet-MHC	0.8422	0.7753	0.7111	0.743	0.4873
	Anthem	0.82	0.605	0.866	0.736	0.489
	MixMHCpred-2.0.2	0.749	0.631	0.819	0.725	0.46
	NetMHCpan-4.1	0.734	0.643	0.722	0.683	0.367
	NetMHCcons-1.1	0.706	0.567	0.771	0.669	0.347
	NetMHCstabpan-1.0	0.703	0.601	0.72	0.661	0.325
	ACME	0.703	0.528	0.848	0.688	0.398
HLA-C*04:01	HLAB	0.8123	0.5909	0.8696	0.7333	0.4809
	CapsNet-MHC	0.8105	0.8421	0.75	0.7949	0.5937
	Anthem	0.78	0.421	0.891	0.656	0.359
	NetMHCpan-4.1	0.604	0.453	0.6	0.526	0.078
	NetMHCcons-1.1	0.602	0.558	0.426	0.492	-0.016
	NetMHCstabpan-1.0	0.587	0.495	0.495	0.495	-0.011
	MixMHCpred-2.0.2	0.561	0.574	0.658	0.616	0.235
HLA-C*05:01	HLAB	0.9583	0.8667	0.875	0.871	0.7417
	CapsNet-MHC	0.7096	0.5	0.8824	0.697	0.4158
	Anthem	0.622	0.388	0.89	0.639	0.334
	NetMHCpan-4.1	0.547	0.481	0.738	0.609	0.238
	MixMHCpred-2.0.2	0.53	0.706	0.488	0.597	0.209
	NetMHCstabpan-1.0	0.525	0.432	0.769	0.6	0.219
	NetMHCcons-1.1	0.503	0.444	0.725	0.585	0.188
HLA-C*06:02	HLAB	0.8129	0.1333	1	0.5738	0.2693
	Anthem	0.734	0.517	0.802	0.659	0.336
	CapsNet-MHC	0.6547	0.7222	0.4324	0.5753	0.1615
	NetMHCpan-4.1	0.521	0.542	0.569	0.556	0.115
	NetMHCcons-1.1	0.521	0.5	0.542	0.521	0.043
	MixMHCpred-2.0.2	0.51	0.456	0.628	0.542	0.087
	MHCNetSeq	0.507	0.944	0.081	0.512	0.038
	NetMHCstabpan-1.0	0.505	0.553	0.53	0.542	0.084

Supplementary Note 4. The detailed results for transformers over IEDB's dataset

In this section, we present a detailed evaluation of CapsNet-MHC and its four transformer-based versions, including CapsNet-MHC-Pae, CapsNet-MHC-contact-map, CapsNet-MHC-ESM1, and CapsNet-MHC-ESM2, over IEDB's datasets. To compare these methods, we utilized AUC and SRCC metrics over IEDB's 61 benchmark datasets. Supplementary Table S6 provides a detailed comparison of these methods using AUC and SRCC metrics over the same 61 benchmark datasets. This evaluation provides insights into the performance of our method and its various transformer-based versions and highlights their effectiveness in peptide-MHC binding prediction.

Supplementary Table S6. Detailed results for pan-specific methods over IEDB's dataset

Measure					results									
					CapsNet-MHC		CapsNet-MHC-Pae		CapsNet-MHC-contact-map		CapsNet-MHC-ESM1		CapsNet-MHC-ESM2	
HLA	IEDB ref	Type	Length	Count	AUC	SRCC	AUC	SRCC	AUC	SRCC	AUC	SRCC	AUC	SRCC
A*02:01	1026371	t1/2	9	34	0.74	0.41	0.74	0.42	0.74	0.42	0.74	0.42	0.74	0.43
A*02:01	1026371	t1/2	10	10	0.54	0.1	0.5	0.09	0.5	0.02	0.54	0.14	0.54	0.06
B*07:02	1026371	t1/2	9	33	0.97	0.84	0.97	0.84	0.98	0.86	1.0	0.87	1	0.85
B*07:02	1026371	t1/2	10	19	0.9	0.7	0.87	0.64	0.88	0.68	0.93	0.67	0.88	0.69
A*02:01	1026371	binary	9	341	0.87	0.5	0.85	0.46	0.86	0.48	0.87	0.5	0.87	0.49
A*02:01	1026371	ic50	9	22	0.76	0.41	0.75	0.36	0.74	0.32	0.76	0.35	0.78	0.43
A*02:01	1026371	t1/2	9	22	0.78	0.49	0.79	0.51	0.81	0.46	0.82	0.5	0.8	0.5
A*24:02	1026371	binary	9	346	0.83	0.36	0.84	0.37	0.81	0.34	0.81	0.35	0.81	0.34
A*24:02	1026371	ic50	9	19	0.74	0.33	0.7	0.25	0.68	0.23	0.69	0.25	0.69	0.21
A*30:01	1026371	binary	9	347	0.85	0.18	0.85	0.18	0.81	0.16	0.86	0.19	0.81	0.14
A*30:02	1026371	binary	9	360	0.75	0.4	0.72	0.36	0.74	0.37	0.74	0.38	0.75	0.4
A*30:02	1026371	ic50	9	56	0.56	0.12	0.55	0.07	0.47	-0.03	0.51	-0.02	0.54	0.03
A*30:02	1026371	t1/2	9	56	0.56	0.1	0.49	0.02	0.49	0.04	0.52	0.03	0.53	0.08

A*68:01	1026371	binary	9	436	0.89	0.41	0.88	0.39	0.89	0.4	0.87	0.39	0.89	0.38
A*68:01	1026371	ic50	9	35	0.84	0.65	0.81	0.61	0.83	0.56	0.84	0.59	0.84	0.57
A*68:01	1026371	t1/2	9	35	0.37	-0.21	0.35	-0.26	0.38	-0.19	0.35	-0.24	0.39	-0.16
B*07:02	1026371	binary	9	288	0.85	0.31	0.84	0.3	0.84	0.3	0.85	0.31	0.86	0.32
A*02:01	1027079	binary	9	18	0.82	0.49	0.83	0.51	0.75	0.39	0.83	0.51	0.74	0.34
A*02:01	1027471	binary	9	43	0.84	0.37	0.78	0.32	0.83	0.37	0.82	0.36	0.84	0.33
A*02:01	1027588	binary	9	18	0.84	0.58	0.86	0.6	0.83	0.56	0.87	0.63	0.82	0.54
C*03:03	1028228	ic50	9	10	0.77	0.53	0.92	0.87	0.72	0.53	0.92	0.9	0.88	0.8
A*02:01	1028285	t1/2	9	135	0.82	0.71	0.74	0.49	0.71	0.49	0.74	0.5	0.78	0.54
A*02:01	1028285	t1/2	10	36	0.8	0.4	0.85	0.73	0.83	0.71	0.82	0.7	0.84	0.74
A*02:01	1028285	t1/2	11	35	0.88	0.71	0.61	0.18	0.75	0.33	0.57	0.18	0.73	0.35
A*11:01	1028287	t1/2	9	177	0.9	0.56	0.82	0.61	0.83	0.61	0.88	0.72	0.85	0.66
A*02:01	1028553	ic50	9	22	0.91	0.65	0.95	0.49	0.88	0.5	0.95	0.52	0.88	0.47
B*07:02	1028553	ic50	9	22	0.92	0.73	0.91	0.7	0.91	0.65	0.89	0.73	0.89	0.57
A*02:01	1028554	ic50	9	44	0.91	0.65	0.89	0.61	0.9	0.71	0.89	0.62	0.89	0.63
B*07:02	1028554	ic50	9	52	0.92	0.73	0.88	0.68	0.84	0.67	0.85	0.67	0.84	0.61
B*35:01	1028554	ic50	9	56	0.62	0.63	0.64	0.42	0.6	0.33	0.65	0.39	0.61	0.31
B*44:03	1028554	ic50	9	46	0.67	0.59	0.64	0.5	0.66	0.5	0.59	0. 5	0.68	0.55
B*57:01	1028554	ic50	9	53	0.84	0.47	0.77	0.43	0.83	0.54	0.8	0.44	0.84	0.48
B*27:03	315174	binary	9	10	0.79	0.5	0.79	0.5	0.79	0.5	0.88	0.64	0.83	0.57
A*02:01	1028790	ic50	10	2	-	1	-	1.0	-	1.0	-	1.0	-	1.0
A*02:03	1028790	ic50	10	2	-	-1	-	-1.0	-	-1.0	-	-1.0	-	-1.0
A*02:06	1028790	ic50	10	2	-	1	-	1.0	-	1.0	-	1.0	-	1.0
A*68:02	1028790	ic50	10	2	-	1	-	1.0	-	1.0	-	1.0	-	1.0
A*02:01	1028928	binary	9	11	1	0.67	1.0	0.67	1.0	0.67	1.0	0.67	1.0	0.67
B*07:02	1028928	binary	9	11	1	0.5	1.0	0.5	1.0	0.5	1.0	0.5	1.0	0.5
C*03:04	315209	t1/2	9	14	1	0.94	1.0	0.83	0.56	0.02	0.91	0.67	0.93	0.7
B*57:01	1029061	ic50	9	17	1	0.68	0.97	0.55	0.93	0.69	1.0	0.76	0.97	0.67

B*27:04	1029125	binary	9	21	0.95	0.73	0.99	0.8	0.97	0.77	0.97	0.77	0.97	0.77
B*27:05	1029125	binary	9	21	0.96	0.75	0.99	0.8	1.0	0.82	0.96	0.75	0.98	0.78
B*27:06	1029125	binary	9	21	0.81	0.52	0.77	0.45	0.86	0.6	0.77	0.45	0.77	0.45
B*15:02	1027131	binary	9	14	1	0.71	1.0	0.71	1.0	0.71	1.0	0.71	1.0	0.71
A*02:01	1029824	binary	9	77	0.53	0.05	0.54	0.06	0.53	0.05	0.55	0.07	0.55	0.07
B*38:01	1029957	ic50	9	4	-	0.8	-	0.4	-	0.6	-	0.8	-	1.0
B*39:06	1029957	ic50	9	33	0.71	0.42	0.74	0.47	0.33	-0.22	0.69	0.24	0.76	0.49
A*31:01	315312	binary	9	8	1	0.87	0.88	0.65	0.94	0.76	0.93	0.65	1.0	0.87
A*66:01	315312	binary	9	16	0.68	0.2	0.43	-0.08	0.54	0.04	0.54	0.04	0.71	0.25
A*03:01	1031253	ic50	9	14	0.89	0.73	0.91	0.76	0.93	0.8	0.93	0.76	0.91	0.77
B*07:02	1031253	ic50	9	13	1	0.97	1.0	0.95	1.0	0.97	1.0	0.94	1.0	0.95
B*27:05	1031253	ic50	9	12	0.57	0.47	0.63	0.57	0.57	0.46	0.6	0.5	0.57	0.44
B*27:05	1031959	binary	8	97	0.45	-0.09	0.44	-0.09	0.43	-0.11	0.44	-0.1	0.45	-0.08
B*27:05	1031959	binary	9	4810	0.61	0.17	0.6	0.16	0.6	0.16	0.61	0.17	0.61	0.17
A*02:01	1033071	ic50	9	102	0.87	0.82	0.86	0.81	0.85	0.79	0.86	0.8	0.86	0.81
A*02:01	1033071	ic50	10	65	0.89	0.81	0.9	0.83	0.86	0.79	0.88	0.8	0.9	0.83
A*02:01	1031072	ic50	9	24	1	0.83	1.0	0.87	1.0	0.85	1.0	0.88	1.0	0.84
A*02:01	1031072	ic50	10	16	0.9	0.82	0.87	0.76	0.92	0.85	0.9	0.88	0.87	0.82
A*02:01	1033576	binary	9	187	0.59	0.07	0.59	0.08	0.58	0.07	0.61	0.1	0.59	0.08
A*02:01	1031894	ic50	9	3	1	0.5	1.0	0.5	1.0	0.5	1.0	0.5	1.0	0.5

Supplementary Note 5. The detailed results for transformers over Anthem's dataset

In this section, we have provided detailed information about the evaluation of CapsNet-MHC and its four transformer-based versions using Anthem's datasets13. Results of the comparison study for various peptide lengths and MHC (HLA) alleles are presented in Supplementary Table S7, including AUC, sensitivity, specificity, accuracy, and MCC metrics.

Supplementary Table S7. Performance comparison of CapsNet-MHC, and its four transformer-based versions on the independent test dataset

Length	HLA-I	Tool	AUC	Sensitivity	Specificity	Accuracy	MCC
8	HLA-A*01:01	CapsNet-MHC-pae	0.9930	0.9104	0.9853	0.9481	0.8987
		CapsNet-MHC	0.9912	0.9851	0.9265	0.9556	0.9127
		CapsNet-MHC-esm2	0.9897	0.9403	0.9559	0.9481	0.8964
		CapsNet-MHC-esm1	0.984	0.9254	0.9269	0.9259	0.8518
		CapsNet-MHC- contact	0.9765	0.8955	0.9559	0.9259	0.8533
	HLA-A*02:01	CapsNet-MHC-contact	0.9259	0.8917	0.8354	0.8635	0.7282
		CapsNet-MHC-esm2	0.9251	0.8471	0.8987	0.873	0.747
		CapsNet-MHC	0.9245	0.8599	0.8924	0.8762	0.7527
		CapsNet-MHC-pae	0.9200	0.8726	0.8671	0.8698	0.7397
		CapsNet-MHC-esm1	0.918	0.8535	0.8686	0.8571	0.7143
	HLA-A*03:01	CapsNet-MHC-esm1	0.9797	0.9123	0.9655	0.9391	0.8794
		CapsNet-MHC-pae	0.9767	0.9289	0.9310	0.9304	0.8609
		CapsNet-MHC	0.9749	0.9298	0.9138	0.9217	0.8436
		CapsNet-MHC-esm2	0.9743	0.9123	0.9483	0.9304	0.8613
		CapsNet-MHC-contact	0.9688	0.8947	0.9483	0.9217	0.8445
	HLA-A*11:01	CapsNet-MHC-pae	0. 9542	0.8235	0.8889	0.8571	0.7148
		CapsNet-MHC	0.9542	0.7647	0.8889	0.8286	0.6601
		CapsNet-MHC-esm2	0.9542	0.7647	0.8889	0.8286	0.6601
		CapsNet-MHC-esm1	0.9477	0.7647	0.8889	0.8286	0.6601
		CapsNet-MHC-contact	0.9248	0.8235	0.8889	0.8571	0.7148
	HLA-A*24:02	CapsNet-MHC-esm2	0.9692	0.9773	0.8889	0.9326	0.8688
		CapsNet-MHC	0.9641	0.9091	0.8667	0.8871	0.7761
		CapsNet-MHC-esm1	0.9611	0.9318	0.9111	0.9213	0.8429
		CapsNet-MHC-contact	0.9596	0.9545	0.8889	0.9213	0.8447
		CapsNet-MHC-pae	0.9591	0.9318	0.8889	0.8698	0.8211
	HLA-A*29:02	CapsNet-MHC	0.989	0.975	0.9268	0.9506	0.9024

	CapsNet-MHC-esm1	0.9866	1	0.9075	0.9532	0.9058
	CapsNet-MHC-esm2	0.984	0.9254	0.9269	0.9259	0.8518
	CapsNet-MHC-pae	0.9817	1	0.9512	0.9753	0.9518
	CapsNet-MHC-contact	0.9780	0.9750	0.9268	0.9506	0.9024
HLA-B*07:02	CapsNet-MHC-pae	0. 9846	0.9227	0.9670	0.9449	0.8907
	CapsNet-MHC-esm2	0.9829	1	0.878	0.9383	0.8835
	CapsNet-MHC	0.9829	0.9337	0.956	0.9449	0.89
	CapsNet-MHC-esm1	0.9811	0.917	0.9689	0.9366	0.8743
	CapsNet-MHC-contact	0.9788	0.9061	0.9670	0.9366	0.8749
HLA-B*08:01	CapsNet-MHC	0.9952	0.9697	0.9612	0.9654	0.9309
	CapsNet-MHC-contact	0.9948	0.9827	0.9483	0.9654	0.9314
	CapsNet-MHC-pae	0.9945	0.9697	0.9612	0.9654	0.9309
	CapsNet-MHC-esm1	0.9941	0.6647	0.9397	0.9525	0.9053
	CapsNet-MHC-esm2	0.9941	0.9827	0.9569	0.9698	0.9398
HLA-B*13:02	CapsNet-MHC	1	1	1	1	1
	CapsNet-MHC-contact	1	1	1	1	1
	CapsNet-MHC-esm1	1	1	1	1	1
	CapsNet-MHC-esm2	1	1	1	1	1
	CapsNet-MHC-pae	0.9954	1	0.9333	0.9655	0.9333
HLA-B*14:02	CapsNet-MHC-esm1	0.9936	0.9909	0.9439	0.968	0.9353
	CapsNet-MHC-contact	0.9918	0.9906	0.9533	0.9718	0.9443
	CapsNet-MHC	0.9913	0.9811	0.9533	0.9671	0.9347
	CapsNet-MHC-esm2	0.9891	0.9906	0.9439	0.9671	0.9353
	CapsNet-MHC-pae	0.9862	0.9717	0.9533	0.9624	0.9251
HLA-B*15:01	CapsNet-MHC	0.978	0.9643	0.9381	0.9511	0.9026
	CapsNet-MHC-contact	0.9779	0.9286	0.8850	0.9067	0.8142
	CapsNet-MHC-pae	0.9771	0.8839	0.9381	0.9111	0.8233
	CapsNet-MHC-esm2	0.9763	0.9196	0.9381	0.9289	0.8579
	CapsNet-MHC-esm1	0.9747	0.9464	0.9115	0.9289	0.8583
HLA-B*18:01	CapsNet-MHC	0.9877	0.981	0.9434	0.9621	0.9248
	CapsNet-MHC-esm2	0.9814	0.9714	0.9623	0.9668	0.9337
	CapsNet-MHC-esm1	0.981	0.9619	0.9717	0.9667	0.9337
	CapsNet-MHC-contact	0.9805	0.9714	0.9528	0.9621	0.9243
	CapsNet-MHC-pae	0.9753	0.9810	0.9623	0.9716	0.9433
HLA-B*18:03	CapsNet-MHC	1	1	0.9545	0.9767	0.9545
	CapsNet-MHC-pae	1	1	0.9545	0.9767	0.9545
	CapsNet-MHC-contact	1	1	0.9545	0.9767	0.9545
	CapsNet-MHC-esm1	1	1	0.9545	0.9767	0.9545
	CapsNet-MHC-esm2	0.9892	1	0.9545	0.9767	0.9545

HLA-B*27:05	CapsNet-MHC-esm2	0.8708	0.7179	0.8025	0.7604	0.5224
	CapsNet-MHC	0.8673	0.7821	0.7962	0.7891	0.5783
	CapsNet-MHC-pae	0.8632	0.7564	0.7771	0.7668	0.5336
	CapsNet-MHC-esm1	0.8551	0.7244	0.8025	0.7637	0.5286
	CapsNet-MHC-contact	0.8541	0.6090	0.8535	0.7316	0.4771
HLA-B*27:09	CapsNet-MHC-esm2	0.9548	0.75	0.9524	0.8537	0.7197
	CapsNet-MHC-esm1	0.9452	0.7	0.9524	0.8293	0.677
	CapsNet-MHC	0.9333	0.85	0.8095	0.8293	0.6595
	CapsNet-MHC-contact	0.9333	0.85	0.8095	0.8293	0.6595
	CapsNet-MHC-pae	0.9143	0.75	0.9081	0.8296	0.6647
HLA-B*35:01	CapsNet-MHC	0.95	0.8261	0.8936	0.8602	0.7217
	CapsNet-MHC-contact	0.9222	0.7826	0.9362	0.8602	0.7283
	CapsNet-MHC-pae	0.9163	0.7826	0.9362	0.8602	0.7283
	CapsNet-MHC-esm1	0.9015	0.8043	0.9362	0.871	0.7478
	CapsNet-MHC-esm2	0.8978	0.8043	0.9574	0.8817	0.7719
HLA-B*37:01	CapsNet-MHC	0.9993	0.9918	0.9756	0.9837	0.9675
	CapsNet-MHC-contact	0.9961	0.9918	0.9593	0.9755	0.9515
	CapsNet-MHC-esm1	0.996	0.9918	0.9593	0.9755	0.9515
	CapsNet-MHC-esm2	0.996	0.9918	0.9675	0.9796	0.9595
	CapsNet-MHC-pae	0.9954	0.9918	0.9675	0.9796	0.9595
HLA-B*39:01	CapsNet-MHC	0.9987	0.8889	1	0.9455	0.896
	CapsNet-MHC-pae	0.9987	0.8519	1	0.9273	0.8633
	CapsNet-MHC-esm1	0.9921	0.8519	1	0.9273	0.8633
	CapsNet-MHC-contact	0.9894	0.8519	0.9643	0.9091	0.8227
	CapsNet-MHC-esm2	0.9881	0.8889	1	0.9455	0.896
HLA-B*39:24	CapsNet-MHC-esm1	1	0.96	1	0.9806	0.9615
	CapsNet-MHC	1	0.92	1	0.9608	0.9243
	CapsNet-MHC-pae	1	0.92	1	0.9608	0.9243
	CapsNet-MHC-contact	0.9954	1	0.9618	0.9894	0.9618
HLA-B*40:01	CapsNet-MHC-esm2	0.9985	0.96	1	0.9804	0.9615
	CapsNet-MHC	0.9935	1	0.9545	0.9767	0.9545
	CapsNet-MHC-pae	0.9935	1	0.9545	0.9767	0.9545
	CapsNet-MHC-contact	0.9892	1	0.9545	0.9767	0.9545
	CapsNet-MHC-esm1	0.9892	1	0.9545	0.9767	0.9545
HLA-B*40:02	CapsNet-MHC	0.9923	0.9632	0.96	0.9616	0.9232
	CapsNet-MHC-esm2	0.9923	0.9632	0.96	0.9616	0.9232
	CapsNet-MHC-esm1	0.9906	0.9565	0.96	0.9583	0.9165
	CapsNet-MHC-pae	0.9901	0.9599	0.9567	0.9583	0.9165
	CapsNet-MHC-contact	0.9885	0.9532	0.9567	0.9549	0.9099

HLA-B*44:02	CapsNet-MHC-esm1	0.9989	0.9643	1	0.9492	0.9028
	CapsNet-MHC-pae	0.9977	0.8966	1	0.9492	0.9028
	CapsNet-MHC	0.9966	0.931	0.9667	0.9492	0.8987
	CapsNet-MHC-contact	0.9931	0.8966	1	0.9492	0.9028
	CapsNet-MHC-esm2	0.992	0.8621	0.9667	0.9153	0.8345
HLA-B*44:03	CapsNet-MHC	0.9815	0.9643	0.9310	0.9474	0.8953
	CapsNet-MHC-pae	0.9754	0.9643	0.9310	0.9474	0.8953
	CapsNet-MHC-esm1	0.9741	0.9643	0.931	0.9474	0.8953
	CapsNet-MHC-esm2	0.9729	0.9643	0.931	0.9474	0.8953
	CapsNet-MHC-contact	0.9581	0.9643	0.9310	0.9474	0.8953
HLA-B*46:01	CapsNet-MHC-contact	0.9833	0.9500	0.8095	0.8789	0.7650
	CapsNet-MHC	0.9762	1	0.8571	0.9268	0.8633
	CapsNet-MHC-esm2	0.9476	1	0.8571	0.9268	0.8633
	CapsNet-MHC-pae	0.9476	0.9500	0. 8571	0.9024	0.8091
	CapsNet-MHC-esm1	0.9353	1	0.8094	0.9024	0.8213
HLA-B*49:01	CapsNet-MHC	0.9985	0.9766	0.9845	0.9805	0.9611
	CapsNet-MHC-contact	0.9983	0.9844	0.97	0.9767	0.9534
	CapsNet-MHC-esm1	0.9981	0.9844	0.9845	0.9844	0.9689
	CapsNet-MHC-pae	0.9976	0.9844	0.97	0.9767	0.9534
	CapsNet-MHC-esm2	0.9965	0.9844	0.969	0.9767	0.9534
HLA-B*51:01	CapsNet-MHC	0.9856	1	0.9444	0.9714	0.9444
	CapsNet-MHC-esm2	0.9833	0.9502	0.9279	0.9391	0.8783
	CapsNet-MHC-pae	0.9820	0.9457	0.9099	0.9278	0.8561
	CapsNet-MHC-esm1	0.9814	0.9321	0.9324	0.9323	0.8646
	CapsNet-MHC-contact	0.9800	0.9548	0.9009	0.9278	0.8568
HLA-B*51:08	CapsNet-MHC	1	1	0.9444	0.9714	0.9444
	CapsNet-MHC-pae	1	1	0.9444	0.9714	0.9444
	CapsNet-MHC-esm2	0.9967	1	0.9444	0.9714	0.9444
	CapsNet-MHC-contact	0.9967	1	0.8333	0.9171	0.8432
	CapsNet-MHC-esm1	0.9821	1	0.9444	0.9714	0.9444
HLA-B*52:01	CapsNet-MHC-contact	0.9776	0.9342	0.9481	0.9412	0.8824
	CapsNet-MHC-pae	0.9711	0.9474	0.9481	0.9477	0.8954
	CapsNet-MHC-esm2	0.9708	0.9342	0.9221	0.9281	0.8563
	CapsNet-MHC-esm1	0.9701	0.9081	0.9481	0.9281	0.8568
	CapsNet-MHC	0.9694	0.9605	0.961	0.9608	0.9216
HLA-B*54:01	CapsNet-MHC-pae	0.9600	0.8400	1	0.9216	0.8532
	CapsNet-MHC-contact	0.9538	0.8400	1	0.9216	0.8532
	CapsNet-MHC	0.9508	0.84	1	0.9216	0.8532
	CapsNet-MHC-esm1	0.9308	0.8	1	0.902	0.8191

HLA-B*57:01	CapsNet-MHC-contact	0.9500	0.8519	0.9634	0.9080	0.8208
	CapsNet-MHC	0.9429	0.8765	0.939	0.9018	0.8046
	CapsNet-MHC-pae	0.9425	0.8148	0.9634	0.8896	0.7875
	CapsNet-MHC-esm2	0.9369	0.84	1	0.9216	0.8532
	CapsNet-MHC-esm1	0.9357	0.8321	0.9524	0.8957	0.796
HLA-B*57:03	CapsNet-MHC-pae	0.9734	0.8246	0.9655	0.8957	0.7988
	CapsNet-MHC	0.9722	0.9298	0.9483	0.9391	0.8784
	CapsNet-MHC-esm2	0.9643	0.8772	0.9655	0.9217	0.8466
	CapsNet-MHC-esm1	0.9607	0.8596	0.9655	0.913	0.8305
	CapsNet-MHC-contact	0.9595	0.8421	0.8966	0.8696	0.7400
HLA-B*58:01	CapsNet-MHC	0.9875	0.961	0.9359	0.9484	0.8971
	CapsNet-MHC-contact	0.9873	0.9740	0.9232	0.9484	0.8980
	CapsNet-MHC-pae	0.9868	0.9610	0.9487	0.9548	0.9098
	CapsNet-MHC-esm1	0.9868	0.961	0.9231	0.9419	0.8846
	CapsNet-MHC-esm2	0.9855	0.974	0.8974	0.9355	0.8736
HLA-C*01:02	CapsNet-MHC	0.9758	0.963	0.8909	0.9266	0.8556
	CapsNet-MHC-esm1	0.9614	0.8519	0.9091	0.8801	0.7625
	CapsNet-MHC-contact	0.9589	0.8888	0.9095	0.8991	0.7983
	CapsNet-MHC-esm2	0.9579	0.8333	0.9273	0.8807	0.7645
	CapsNet-MHC-pae	0.9451	0.9073	0.8791	0.8898	0.7829
HLA-C*02:02	CapsNet-MHC-pae	0.9933	0.9583	0.92	0.9385	0.8786
	CapsNet-MHC-esm2	0.99	0.9583	0.92	0.9388	0.8783
	CapsNet-MHC-esm1	0.9867	0.9167	0.92	0.9184	0.8367
	CapsNet-MHC-contact	0.985	0.9583	0.88	0.9184	0.8397
	CapsNet-MHC	0.9833	0.9167	0.92	0.9184	0.8367
HLA-C*03:03	CapsNet-MHC	0.9723	0.9412	0.9143	0.9275	0.8555
	CapsNet-MHC-contact	0.9714	0.9118	0.8857	0.8986	0.7975
	CapsNet-MHC-esm1	0.9714	0.9118	0.8857	0.8986	0.7975
	CapsNet-MHC-esm2	0.9664	0.9118	0.8857	0.8986	0.7975
	CapsNet-MHC-pae	0.9647	0.8529	0.9143	0.8841	0.7692
HLA-C*03:04	CapsNet-MHC	0.997	0.9825	0.9655	0.9739	0.948
	CapsNet-MHC-pae	0.9943	0.9474	0.9828	0.9652	0.9310
	CapsNet-MHC-esm1	0.9933	0.9474	0.9655	0.9565	0.9132
	CapsNet-MHC-contact	0.9930	0.9474	0.9828	0.9652	0.9310
	CapsNet-MHC-esm2	0.9918	0.9474	0.9828	0.9652	0.9310
HLA-C*04:01	CapsNet-MHC	0.9746	0.8671	0.9497	0.9085	0.8197
	CapsNet-MHC-pae	0.9695	0.8671	0.9434	0.9054	0.8130
	CapsNet-MHC-contact	0.9691	0.8671	0.9623	0.9148	0.8333
	CapsNet-MHC-esm2	0.969	0.8734	0.9623	0.918	0.8392

	CapsNet-MHC-esm1	0.9656	0.8481	0.9623	0.9054	0.8159
HLA-C*05:01	CapsNet-MHC	0.9981	0.9931	0.9448	0.9689	0.9388
	CapsNet-MHC-pae	0.9968	0.9861	0.9793	0.9827	0.9654
	CapsNet-MHC-esm1	0.9961	0.9861	0.9448	0.9654	0.9316
	CapsNet-MHC-esm2	0.9947	0.9861	0.9448	0.9654	0.9316
	CapsNet-MHC-contact	0.9943	0.9861	0.9581	0.9723	0.9450
HLA-C*06:02	CapsNet-MHC-pae	0.9889	0.9143	0.9722	0.9437	0.8886
	CapsNet-MHC-esm2	0.9825	0.8857	0.9444	0.9155	0.8321
	CapsNet-MHC	0.9762	0.8286	0.9167	0.8732	0.7488
	CapsNet-MHC-contact	0.9738	0.7143	0.9722	0.8451	0.7124
	CapsNet-MHC-esm1	0.9563	0.6571	0.9444	0.8028	0.6278
HLA-C*07:01	CapsNet-MHC-esm1	0.9961	0.9778	0.9565	0.967	0.9343
	CapsNet-MHC	0.9947	0.9778	0.9384	0.956	0.913
	CapsNet-MHC-esm2	0.9942	1	0.9565	0.978	0.957
	CapsNet-MHC-pae	0.9928	0.9778	0.9565	0.9670	0.9343
	CapsNet-MHC-contact	0.9918	0.9778	0.9348	0.9560	0.9130
HLA-C*07:02	CapsNet-MHC	0.9986	0.9811	0.963	0.972	0.9441
	CapsNet-MHC-pae	0.9986	0.9811	0.9444	0.9626	0.9259
	CapsNet-MHC-contact	0.9979	0.9811	0.963	0.972	0.9441
	CapsNet-MHC-esm2	0.9979	0.9811	0.9259	0.9533	0.908
	CapsNet-MHC-esm1	0.9948	0.9811	0.9444	0.9626	0.9259
HLA-C*07:04	CapsNet-MHC-pae	0.9947	1	0.95	0.9744	0.95
	CapsNet-MHC-contact	0.993	1	0.95	0.9744	0.95
	CapsNet-MHC	0.9816	1	0.95	0.9744	0.95
	CapsNet-MHC-esm1	0.9763	1	0.95	0.744	0.95
	CapsNet-MHC-esm2	0.9763	1	0.95	0.744	0.95
HLA-C*08:02	CapsNet-MHC	1	1	0.9939	0.9969	0.9939
	CapsNet-MHC-pae	1	0.9938	1	0.9969	0.9939
	CapsNet-MHC-esm2	0.9999	1	0.9816	0.9908	0.9817
	CapsNet-MHC-esm1	0.9997	0.9938	0.9877	0.9908	0.9816
	CapsNet-MHC-contact	0.9994	0.9938	0.9755	0.9846	0.9694
HLA-C*12:03	CapsNet-MHC-pae	0.9903	1	0.9355	0.9672	0.9365
	CapsNet-MHC	0.9871	1	0.9032	0.9508	0.9062
	CapsNet-MHC-esm2	0.9828	1	0.871	0.9344	0.8766
	CapsNet-MHC-esm1	0.9817	1	0.871	0.9344	0.8766
	CapsNet-MHC-contact	0.9806	0.9333	0.9032	0.9180	0.8366
HLA-C*14:02	CapsNet-MHC	0.9998	1	0.9694	0.9846	0.9697
	CapsNet-MHC-pae	0.9998	1	0.9490	0.9744	0.9500
	CapsNet-MHC-esm2	0.9997	1	0.949	0.9744	0.95

		CapsNet-MHC-esm1	0.9996	1	0.9592	0.9795	0.9598
		CapsNet-MHC-contact	0.9972	0.9897	0.9594	0.9744	0.9492
	HLA-C*15:02	CapsNet-MHC-contact	0.9974	0.9487	0.975	0.962	0.9243
		CapsNet-MHC-pae	0.9968	1	0.9750	0.9873	0.9750
		CapsNet-MHC	0.9936	1	0.925	0.962	0.9268
		CapsNet-MHC-esm1	0.9917	1	0.9750	0.9873	0.9750
		CapsNet-MHC-esm2	0.9917	1	0.95	0.9747	0.9506
	HLA-C*16:01	CapsNet-MHC-esm2	0.9924	0.9742	0.9295	0.9518	0.9045
		CapsNet-MHC	0.9892	0.9806	0.9103	0.9453	0.8929
		CapsNet-MHC-esm1	0.9892	0.9613	0.9295	0.9453	0.8911
		CapsNet-MHC-pae	0.9835	0.9806	0.9163	0.9483	0.899
		CapsNet-MHC-contact	0.9825	0.9482	0.9158	0.9365	0.8659
	HLA-C*17:01	CapsNet-MHC-esm1	0.9615	0.88	0.8462	0.8627	0.7262
		CapsNet-MHC-pae	0.9509	0.968	0.8077	0.8823	0.7749
		CapsNet-MHC	0.9499	1	0.8077	0.902	0.8204
		CapsNet-MHC-contact	0.9477	0.8424	0.8476	0.8442	0.6841
		CapsNet-MHC-esm2	0.9415	0.95	0.8077	0.8824	0.7749
9	HLA-A*01:01	CapsNet-MHC	0.9872	0.9406	0.9589	0.9497	0.8996
		CapsNet-MHC-pae	0.9872	0.9368	0.9569	0.9469	0.8939
		CapsNet-MHC-esm2	0.9867	0.9377	0.956	0.9469	0.8939
		CapsNet-MHC-esm1	0.9849	0.9387	0.9502	0.9445	0.889
		CapsNet-MHC-contact	0.9841	0.9387	0.9531	0.9459	0.8919
	HLA-A*02:01	CapsNet-MHC	0.964	0.9088	0.9085	0.9086	0.8173
		CapsNet-MHC-pae	0.9631	0.9000	0.9147	0.9094	0.8188
		CapsNet-MHC-contact	0.9841	0.9082	0.9106	0.9459	0.8919
		CapsNet-MHC-esm2	0.9631	0.9023	0.9142	0.9083	0.8166
		CapsNet-MHC-esm1	0.9629	0.903	0.9132	0.9083	0.8165
	HLA-A*02:02	CapsNet-MHC	0.9659	0.9106	0.901	0.9058	0.8116
		CapsNet-MHC-esm1	0.964	0.9106	0.9241	0.9174	0.8348
		CapsNet-MHC-esm2	0.9629	0.8974	0.9175	0.9074	0.815
		CapsNet-MHC-contact	0.9594	0.8954	0.9348	0.9129	0.8208
		CapsNet-MHC-pae	0.9593	0.8450	0.9408	0.8976	0.7865
	HLA-A*02:03	CapsNet-MHC-esm1	0.9778	0.9522	0.9138	0.933	0.8667
		CapsNet-MHC	0.9778	0.9522	0.9046	0.9284	0.8578
		CapsNet-MHC-esm2	0.9776	0.9553	0.9123	0.9338	0.8684
		CapsNet-MHC-contact	0.9770	0.9354	0.9048	0.9129	0.8434
		CapsNet-MHC-pae	0.9769	0.9340	0.9234	0.9245	0.8544
	HLA-A*02:04	CapsNet-MHC	0.9881	0.9493	0.9386	0.9439	0.8879
		CapsNet-MHC-esm1	0.9841	0.9674	0.917	0.9421	0.8854

	CapsNet-MHC-esm2	0.984	0.9674	0.9314	0.9494	0.8993
	CapsNet-MHC-contact	0.9762	0.9654	0.9048	0.9389	0.8754
	CapsNet-MHC-pae	0.9846	0.9740	0.9134	0.9485	0.8894
HLA-B*07:02	CapsNet-MHC	0.9888	0.9576	0.9532	0.9554	0.9108
	CapsNet-MHC-pae	0.9885	0.9518	0.9569	0.9569	0.9039
	CapsNet-MHC-esm2	0.9875	0.9537	0.9518	0.9527	0.9055
	CapsNet-MHC-contact	0.9874	0.9587	0.9531	0.9559	0.9019
	CapsNet-MHC-esm1	0.9866	0.9508	0.9581	0.9544	0.9089
HLA-B*08:01	CapsNet-MHC	0.9881	0.947	0.9489	0.9479	0.8959
	CapsNet-MHC-pae	0.9872	0.9468	0.9379	0.9449	0.8872
	CapsNet-MHC-esm1	0.9852	0.9508	0.9309	0.9408	0.8819
	CapsNet-MHC-contact	0.9847	0.9587	0.9531	0.9559	0.9019
	CapsNet-MHC-esm2	0.9844	0.9498	0.9396	0.9432	0.8865
HLA-B*13:02	CapsNet-MHC	0.9965	0.9851	0.9735	0.9793	0.9586
	CapsNet-MHC-esm2	0.995	0.9801	0.9454	0.9627	0.926
	CapsNet-MHC-pae	0.9929	0.9382	0.9678	0.9538	0.9076
	CapsNet-MHC-esm1	0.992	0.9834	0.9371	0.9602	0.9215
	CapsNet-MHC-contact	0.9886	0.9751	0.9118	0.9485	0.8969
HLA-B*14:01	CapsNet-MHC	1	1	1	1	1
	CapsNet-MHC-pae	1	1	1	1	1
	CapsNet-MHC-esm1	1	1	1	1	1
	CapsNet-MHC-esm2	1	1	1	1	1
	CapsNet-MHC-contact	1	1	0.9771	0.9554	0.9761
HLA-B*14:02	CapsNet-MHC	0.9914	0.974	0.9497	0.9619	0.924
	CapsNet-MHC-esm2	0.99	0.9771	0.9345	0.9558	0.9124
	CapsNet-MHC-pae	0.9896	0.974	0.9397	0.9559	0.9124
	CapsNet-MHC-contact	0.9895	0.9855	0.9235	0.9565	0.9133
	CapsNet-MHC-esm1	0.9871	0.9771	0.9314	0.9542	0.9094
HLA-B*15:01	CapsNet-MHC-pae	0.9876	0.9582	0.9585	0.9548	0.9056
	CapsNet-MHC	0.9876	0.9552	0.9485	0.9518	0.9036
	CapsNet-MHC-esm2	0.9864	0.9564	0.9422	0.9493	0.8987
	CapsNet-MHC-contact	0.9861	0.965	0.9215	0.9465	0.8983
	CapsNet-MHC-esm1	0.9848	0.9608	0.9326	0.9466	0.8935
HLA-B*15:02	CapsNet-MHC-esm2	0.985	0.9623	0.9259	0.9439	0.8885
	CapsNet-MHC-esm1	0.9818	0.9434	0.9444	0.9439	0.8878
	CapsNet-MHC-contact	0.979	0.9822	0.9454	0.9612	0.9247
	CapsNet-MHC	0.979	0.9623	0.9444	0.9533	0.9067
	CapsNet-MHC-pae	0.9766	0.9628	0.9489	0.9590	0.9064
HLA-B*15:03	CapsNet-MHC	0.9821	0.9878	0.8675	0.9273	0.861

	CapsNet-MHC-pae	0.9728	0.8268	0.9674	0.9980	0.8003
	CapsNet-MHC-contact	0.9608	0.7907	0.9212	0.8642	0.7316
	CapsNet-MHC-esm2	0.9594	0.8049	0.9277	0.8667	0.7386
	CapsNet-MHC-esm1	0.9354	0.7317	0.9036	0.8182	0.6454
HLA-B*15:09	CapsNet-MHC-esm2	1	0.8571	1	0.9302	0.8685
	CapsNet-MHC	0.9978	0.7619	1	0.8837	0.7879
	CapsNet-MHC-esm1	0.9957	0.7619	1	0.8837	0.7879
	CapsNet-MHC-contact	0.9935	0.8065	1	0.9086	0.8276
	CapsNet-MHC-pae	0.9589	0.7120	0.9564	0.8345	0.6917
HLA-B*15:11	CapsNet-MHC	0.9773	0.9514	0.914	0.9326	0.8659
	CapsNet-MHC-pae	0.9771	0.9342	0.9126	0.9234	0.8498
	CapsNet-MHC-esm2	0.9686	0.9135	0.9194	0.9164	0.8329
	CapsNet-MHC-esm1	0.9651	0.9189	0.8978	0.9084	0.8169
	CapsNet-MHC-contact	0.9565	0.9214	0.8514	0.8926	0.7869
HLA-B*15:17	CapsNet-MHC	0.9979	0.9954	0.987	0.988	0.976
	CapsNet-MHC-pae	0.9438	0.8978	0.8701	0.883	0.7698
	CapsNet-MHC-esm1	0.933	0.8586	0.87	0.8642	0.7287
	CapsNet-MHC-contact	0.9329	0.8564	0.8976	0.7821	0.7438
	CapsNet-MHC-esm2	0.9306	0.8788	0.87	0.8744	0.7488
HLA-B*15:18	CapsNet-MHC	0.9979	0.9954	0.987	0.988	0.976
	CapsNet-MHC-esm2	0.9943	0.9907	0.9677	0.9792	0.9587
	CapsNet-MHC-esm1	0.9927	0.9722	0.9585	0.9654	0.9308
	CapsNet-MHC-contact	0.9885	0.9878	0.9301	0.9523	0.9138
	CapsNet-MHC-pae	0.9869	0.9564	0.9376	0.9421	0.8838
HLA-B*15:42	CapsNet-MHC	0.9816	0.9437	0.8472	0.8951	0.7942
	CapsNet-MHC-contact	0.9603	0.9067	0.8333	0.8651	0.7372
	CapsNet-MHC-esm2	0.9591	0.8732	0.8889	0.8811	0.7623
	CapsNet-MHC-pae	0.9577	0.9078	0.8451	0.8765	0.7495
	CapsNet-MHC-esm1	0.9417	0.831	0.8611	0.8462	0.6925
HLA-B*18:01	CapsNet-MHC	0.989	0.9615	0.9472	0.9544	0.9089
	CapsNet-MHC-esm2	0.987	0.9303	0.964	0.9474	0.8949
	CapsNet-MHC-pae	0.9861	0.9115	0.9772	0.9444	0.8959
	CapsNet-MHC-contact	0.9859	0.9337	0.9541	0.9453	0.8806
	CapsNet-MHC-esm1	0.9856	0.9207	0.964	0.9424	0.8856
HLA-B*18:03	CapsNet-MHC	0.965	0.8438	0.9091	0.8769	0.755
	CapsNet-MHC-esm1	0.9611	0.75	0.9697	0.8615	0.7396
	CapsNet-MHC-esm2	0.9545	0.75	0.9394	0.8462	0.7035
	CapsNet-MHC-pae	0.9299	0.6872	0.9385	0.8131	0.6496
	CapsNet-MHC-contact	0.9186	0.75	0.909	0.8306	0.6686

HLA-B*27:01	CapsNet-MHC-contact	0.9949	0.9944	0.9676	0.9845	0.9687
	CapsNet-MHC	0.9945	0.9974	0.9683	0.9828	0.9661
	CapsNet-MHC-pae	0.9936	0.9998	0.9376	0.9684	0.9369
	CapsNet-MHC-esm2	0.9926	0.9947	0.9631	0.9789	0.9582
	CapsNet-MHC-esm1	0.9924	0.9947	0.9683	0.9815	0.9633
HLA-B*27:02	CapsNet-MHC-pae	0.9982	0.9844	0.9822	0.9876	0.9651
	CapsNet-MHC-contact	0.9978	0.9937	0.9641	0.9753	0.9506
	CapsNet-MHC	0.9976	0.9883	0.9922	0.9903	0.9806
	CapsNet-MHC-esm2	0.9975	0.9844	0.9884	0.9864	0.9728
	CapsNet-MHC-esm1	0.9974	0.9844	0.9845	0.9845	0.9689
HLA-B*27:03	CapsNet-MHC	0.993	0.9608	0.9903	0.9756	0.9516
	CapsNet-MHC-pae	0.9913	0.9854	0.9456	0.9635	0.9251
	CapsNet-MHC-contact	0.9910	0.9814	0.9036	0.945	0.8858
	CapsNet-MHC-esm1	0.9888	0.9706	0.9417	0.9561	0.9126
	CapsNet-MHC-esm2	0.989	0.9804	0.9515	0.9659	0.9321
HLA-B*27:04	CapsNet-MHC	0.9958	0.9758	0.9783	0.9771	0.9542
	CapsNet-MHC-pae	0.9955	0.9769	0.9642	0.9762	0.9436
	CapsNet-MHC-contact	0.9948	0.9803	0.9579	0.9676	0.9358
	CapsNet-MHC-esm2	0.9942	0.9783	0.9687	0.9735	0.947
	CapsNet-MHC-esm1	0.9941	0.9783	0.9663	0.9723	0.9446
HLA-B*27:05	CapsNet-MHC-esm1	0.9393	0.8475	0.8889	0.8687	0.7381
	CapsNet-MHC-esm2	0.9379	0.8513	0.8881	0.8697	0.7397
	CapsNet-MHC-pae	0.9368	0.8475	0.8763	0.8655	0.7266
	CapsNet-MHC	0.9367	0.8485	0.8815	0.865	0.7303
	CapsNet-MHC-contact	0.9341	0.8165	0.8956	0.8533	0.7167
HLA-B*27:06	CapsNet-MHC	0.9947	0.9691	0.9755	0.9723	0.9446
	CapsNet-MHC-esm1	0.9947	0.9753	0.9632	0.9692	0.9385
	CapsNet-MHC-pae	0.9945	0.9769	0.9642	0.9762	0.9436
	CapsNet-MHC-esm2	0.9933	0.9753	0.9693	0.9723	0.9443
	CapsNet-MHC-contact	0.9918	0.9713	0.9213	0.9591	0.9082
HLA-B*27:07	CapsNet-MHC-contact	0.997	1	0.9389	0.9689	0.9379
	CapsNet-MHC	0.9964	1	0.9865	0.9932	0.9865
	CapsNet-MHC-esm1	0.9962	1	0.9775	0.9887	0.9777
	CapsNet-MHC-esm2	0.9957	1	0.9775	0.9885	0.9777
	CapsNet-MHC-pae	0.9954	1	0.9595	0.9797	0.9602
HLA-B*27:08	CapsNet-MHC	0.9989	0.9947	0.9947	0.9947	0.9894
	CapsNet-MHC-esm1	0.9989	0.9947	0.9894	0.992	0.9846
	CapsNet-MHC-esm2	0.9987	0.9894	0.9894	0.9894	0.9788
	CapsNet-MHC-pae	0.9979	0.9747	0.9747	0.9747	0.9594

	CapsNet-MHC-contact	0.9935	0.9689	0.9258	0.9485	0.8892
HLA-B*27:09	CapsNet-MHC	0.9849	0.923	0.9476	0.9353	0.8709
	CapsNet-MHC-esm2	0.9836	0.9197	0.946	0.9328	0.866
	CapsNet-MHC-esm1	0.9822	0.918	0.9508	0.9343	0.8694
	CapsNet-MHC-pae	0.981	0.9023	0.9576	0.9253	0.8599
	CapsNet-MHC-contact	0.978	0.9394	0.9027	0.921	0.8412
HLA-B*27:20	CapsNet-MHC	0.9921	0.8421	0.95	0.8974	0.7984
	CapsNet-MHC-contact	0.9895	0.9467	0.9513	0.9451	0.8972
	CapsNet-MHC-esm2	0.9842	0.7895	0.95	0.8718	0.7514
	CapsNet-MHC-pae	0.9816	0.8978	0.95	0.9241	0.8465
	CapsNet-MHC-esm1	0.9763	0.7895	0.95	0.8718	0.7514
HLA-B*35:01	CapsNet-MHC	0.9839	0.9479	0.94	0.944	0.8879
	CapsNet-MHC-pae	0.9822	0.954	0.9461	0.9359	0.8951
	CapsNet-MHC-esm1	0.9814	0.9473	0.9375	0.9424	0.8849
	CapsNet-MHC-esm2	0.9813	0.9468	0.9406	0.9436	0.8873
	CapsNet-MHC-contact	0.9805	0.9514	0.936	0.945	0.8832
HLA-B*35:03	CapsNet-MHC	0.997	0.9836	0.97	0.9768	0.9537
	CapsNet-MHC-pae	0.9951	0.972	0.9464	0.9682	0.9236
	CapsNet-MHC-esm2	0.995	0.9768	0.955	0.9659	0.9321
	CapsNet-MHC-esm1	0.9946	0.9713	0.9591	0.9652	0.9305
	CapsNet-MHC-contact	0.9945	0.9823	0.9455	0.9689	0.9324
HLA-B*35:08	CapsNet-MHC-esm1	0.9864	0.9536	0.9538	0.9537	0.9075
	CapsNet-MHC	0.9853	0.9433	0.9538	0.9486	0.8972
	CapsNet-MHC-esm2	0.982	0.9433	0.9538	0.9489	0.8972
	CapsNet-MHC-contact	0.9808	0.952	0.9675	0.9537	0.9176
	CapsNet-MHC-pae	0.9795	0.9564	0.9452	0.958	0.9017
HLA-B*37:01	CapsNet-MHC	0.9964	0.984	0.9753	0.9797	0.9594
	CapsNet-MHC-esm2	0.9948	0.9827	0.9681	0.9753	0.9507
	CapsNet-MHC-contact	0.9946	0.9843	0.9709	0.9776	0.9552
	CapsNet-MHC-pae	0.9942	0.9803	0.9653	0.9753	0.942
	CapsNet-MHC-esm1	0.9938	0.9869	0.9637	0.9753	0.9509
HLA-B*38:01	CapsNet-MHC	0.9971	0.991	0.9821	0.9865	0.9731
	CapsNet-MHC-esm2	0.9963	0.9885	0.977	0.9829	0.9655
	CapsNet-MHC-contact	0.9963	0.9882	0.9689	0.9789	0.9578
	CapsNet-MHC-pae	0.9963	0.9891	0.9721	0.9805	0.9601
	CapsNet-MHC-esm1	0.9953	0.9846	0.9693	0.9769	0.954
HLA-B*39:01	CapsNet-MHC-pae	0.9952	0.9582	0.9627	0.9624	0.9239
	CapsNet-MHC	0.9949	0.9672	0.9717	0.9694	0.9389
	CapsNet-MHC-contact	0.9945	0.9717	0.9627	0.9697	0.939

	CapsNet-MHC-esm1	0.9942	0.9626	0.9683	0.9655	0.931
	CapsNet-MHC-esm2	0.9941	0.957	0.9638	0.9604	0.9208
HLA-B*39:06	CapsNet-MHC	0.9974	0.9905	0.9779	0.9842	0.9685
	CapsNet-MHC-contact	0.997	0.9937	0.9716	0.9826	0.9655
	CapsNet-MHC-pae	0.9949	0.984	0.9562	0.9637	0.9327
	CapsNet-MHC-esm2	0.9924	0.9717	0.9621	0.9668	0.9337
	CapsNet-MHC-esm1	0.9923	0.9652	0.959	0.9623	0.9242
HLA-B*39:24	CapsNet-MHC-esm1	0.9932	1	0.9082	0.9537	0.9116
	CapsNet-MHC	0.9925	0.9794	0.9184	0.9487	0.8992
	CapsNet-MHC-esm2	0.9876	1	0.9081	0.9538	0.9116
	CapsNet-MHC-contact	0.9817	0.9858	0.8975	0.9467	0.8913
	CapsNet-MHC-pae	0.981	1	0.9158	0.9562	0.9214
HLA-C*01:02	CapsNet-MHC	0.9975	0.9885	0.9696	0.979	0.9583
	CapsNet-MHC-esm1	0.9902	0.9885	0.9582	0.9733	0.9471
	CapsNet-MHC-esm2	0.9902	0.9697	0.962	0.9657	0.9315
	CapsNet-MHC-pae	0.9884	0.9466	0.9571	0.9479	0.8981
	CapsNet-MHC-contact	0.9795	0.9371	0.9201	0.9276	0.8592
HLA-C*02:02	CapsNet-MHC	0.9872	0.9748	0.9313	0.953	0.9069
	CapsNet-MHC-esm2	0.9862	0.9777	0.9284	0.953	0.9072
	CapsNet-MHC-contact	0.9843	0.9759	0.9	0.9356	0.8734
	CapsNet-MHC-esm1	0.9836	0.969	0.9245	0.9467	0.8944
	CapsNet-MHC-pae	0.9832	0.9768	0.9061	0.9401	0.8846
HLA-C*03:03	CapsNet-MHC-contact	0.9849	0.9737	0.9216	0.9426	0.8999
	CapsNet-MHC	0.9842	0.962	0.9399	0.951	0.9022
	CapsNet-MHC-esm1	0.9842	0.9673	0.9336	0.9504	0.9014
	CapsNet-MHC-esm2	0.9841	0.9694	0.9347	0.952	0.9046
	CapsNet-MHC-pae	0.9838	0.9784	0.9282	0.9437	0.9001
HLA-C*03:04	CapsNet-MHC	0.9931	0.987	0.9479	0.9674	0.9356
	CapsNet-MHC-contact	0.9930	0.9737	0.9516	0.9626	0.9355
	CapsNet-MHC-pae	0.9929	0.984	0.9572	0.9617	0.9347
	CapsNet-MHC-esm2	0.9926	0.9846	0.9361	0.9603	0.9281
	CapsNet-MHC-esm1	0.9926	0.987	0.9337	0.9603	0.922
HLA-C*04:01	CapsNet-MHC-contact	0.9767	0.9341	0.923	0.9385	0.8625
	CapsNet-MHC	0.9761	0.9315	0.9414	0.9365	0.873
	CapsNet-MHC-esm2	0.9759	0.9321	0.9328	0.9325	0.8649
	CapsNet-MHC-pae	0.9756	0.9367	0.947	0.9319	0.8741
	CapsNet-MHC-esm1	0.9746	0.9266	0.9383	0.9325	0.865
HLA-C*05:01	CapsNet-MHC	0.9928	0.9714	0.9578	0.9646	0.9292
	CapsNet-MHC-pae	0.9911	0.9592	0.9511	0.955	0.9101

	CapsNet-MHC-esm2	0.9909	0.9645	0.9469	0.9469	0.9115
	CapsNet-MHC-esm1	0.9906	0.9632	0.9469	0.955	0.9101
	CapsNet-MHC-contact	0.9905	0.97	0.9405	0.9504	0.9108
HLA-C*06:02	CapsNet-MHC	0.9829	0.9738	0.9223	0.948	0.8973
	CapsNet-MHC-contact	0.9898	0.9649	0.9127	0.948	0.8842
	CapsNet-MHC-pae	0.9796	0.9611	0.9242	0.9467	0.8837
	CapsNet-MHC-esm2	0.9795	0.9747	0.9133	0.944	0.8898
	CapsNet-MHC-esm1	0.9793	0.9683	0.9143	0.9413	0.8838
HLA-C*07:01	CapsNet-MHC	0.9847	0.9564	0.9537	0.955	0.9101
	CapsNet-MHC-contact	0.9824	0.9603	0.9324	0.9429	0.8957
	CapsNet-MHC-pae	0.982	0.9606	0.9368	0.9587	0.9017
	CapsNet-MHC-esm1	0.9804	0.9648	0.9326	0.9487	0.8979
	CapsNet-MHC-esm2	0.9796	0.9705	0.9312	0.9508	0.9023
HLA-C*07:02	CapsNet-MHC	0.9857	0.9695	0.9197	0.9446	0.8903
	CapsNet-MHC-esm2	0.9831	0.963	0.9085	0.9357	0.8726
	CapsNet-MHC-esm1	0.982	0.963	0.9085	0.9357	0.8728
	CapsNet-MHC-contact	0.9816	0.9653	0.9121	0.9437	0.8825
	CapsNet-MHC-pae	0.9810	0.9637	0.916	0.9426	0.8853
HLA-C*07:04	CapsNet-MHC-esm1	0.9899	0.9706	0.9396	0.9551	0.9106
	CapsNet-MHC-esm2	0.989	0.9633	0.9469	0.9551	0.9106
	CapsNet-MHC	0.9887	0.9615	0.9524	0.9569	0.9139
	CapsNet-MHC-pae	0.9886	0.9836	0.9339	0.9573	0.9166
	CapsNet-MHC-contact	0.9872	0.9715	0.9052	0.9383	0.8793
HLA-C*08:02	CapsNet-MHC	0.9916	0.9796	0.9542	0.9669	0.9341
	CapsNet-MHC-pae	0.9914	0.9787	0.948	0.9652	0.9275
	CapsNet-MHC-contact	0.9913	0.9893	0.9413	0.9633	0.9286
	CapsNet-MHC-esm1	0.9911	0.9796	0.9481	0.9639	0.9282
	CapsNet-MHC-esm2	0.9909	0.9817	0.9471	0.9644	0.9293
HLA-C*12:03	CapsNet-MHC	0.9913	0.976	0.9468	0.9614	0.9232
	CapsNet-MHC-esm2	0.9882	0.9653	0.9388	0.9521	0.9045
	CapsNet-MHC-esm1	0.9881	0.9733	0.9309	0.9523	0.905
	CapsNet-MHC-pae	0.9858	0.9643	0.9438	0.9534	0.9098
	CapsNet-MHC-contact	0.9840	0.9567	0.9369	0.9467	0.8854
HLA-C*14:02	CapsNet-MHC	0.995	0.9891	0.9673	0.9782	0.9566
	CapsNet-MHC-esm2	0.9943	0.9945	0.9482	0.9714	0.9437
	CapsNet-MHC-esm1	0.9934	0.9781	0.9373	0.9577	0.9162
	CapsNet-MHC-pae	0.9918	0.9808	0.9473	0.9632	0.9269
	CapsNet-MHC-contact	0.9893	0.9898	0.9367	0.9668	0.9235
HLA-C*15:02	CapsNet-MHC	0.9902	0.9806	0.9543	0.9624	0.9313

		CapsNet-MHC-pae	0.9869	0.9656	0.9396	0.9585	0.9087
		CapsNet-MHC-esm2	0.9818	0.9557	0.9199	0.9378	0.8761
		CapsNet-MHC-esm1	0.9802	0.9583	0.9171	0.9378	0.8763
		CapsNet-MHC-contact	0.9792	0.9359	0.92	0.9238	0.8515
	HLA-C*16:01	CapsNet-MHC	0.9907	0.9847	0.9266	0.9556	0.9128
		CapsNet-MHC-esm1	0.9868	0.9801	0.9312	0.9556	0.9123
		CapsNet-MHC-esm2	0.9865	0.977	0.9419	0.9594	0.9195
		CapsNet-MHC-pae	0.9847	0.9872	0.9233	0.9513	0.9057
		CapsNet-MHC-contact	0.9796	0.9549	0.9172	0.9351	0.8751
	HLA-C*17:01	CapsNet-MHC	0.9849	0.9286	0.9529	0.9408	0.8819
		CapsNet-MHC-esm2	0.9791	0.9405	0.9176	0.929	0.8582
		CapsNet-MHC-esm1	0.9779	0.9167	0.9176	0.9172	0.8343
		CapsNet-MHC-pae	0.9732	0.905	0.9298	0.9171	0.8344
		CapsNet-MHC-contact	0.962	0.8931	0.9059	0.8993	0.7989
10	HLA-A*01:01	CapsNet-MHC	0.9924	0.9575	0.9621	0.9598	0.9196
		CapsNet-MHC-pae	0.9917	0.9508	0.9573	0.9532	0.9069
		CapsNet-MHC-esm2	0.9908	0.9597	0.9442	0.952	0.904
		CapsNet-MHC-esm1	0.9906	0.9575	0.9554	0.9564	0.9129
		CapsNet-MHC-contact	0.9905	0.9698	0.9567	0.9568	0.9195
	HLA-A*02:01	CapsNet-MHC-contact	0.9605	0.9188	0.8825	0.9033	0.8069
		CapsNet-MHC-pae	0.9585	0.9018	0.9025	0.9051	0.8046
		CapsNet-MHC	0.9584	0.9178	0.8925	0.9051	0.8106
		CapsNet-MHC-esm1	0.9568	0.9141	0.8925	0.9033	0.8067
		CapsNet-MHC-esm2	0.9549	0.916	0.8925	0.9042	0.8086
	HLA-A*02:02	CapsNet-MHC-contact	0.9677	0.92	0.9154	0.9177	0.8354
		CapsNet-MHC	0.9675	0.94	0.9104	0.9252	0.8508
		CapsNet-MHC-esm2	0.9674	0.92	0.9154	0.9177	0.8354
		CapsNet-MHC-esm1	0.9655	0.95	0.9055	0.9277	0.8562
		CapsNet-MHC-pae	0.9640	0.905	0.9206	0.9127	0.8255
	HLA-A*02:03	CapsNet-MHC-contact	0.9639	0.9281	0.9283	0.9282	0.8565
		CapsNet-MHC	0.9737	0.9469	0.919	0.9392	0.8662
		CapsNet-MHC-esm1	0.9724	0.9467	0.919	0.9329	0.8662
		CapsNet-MHC-pae	0.9718	0.9125	0.9377	0.9251	0.8505
		CapsNet-MHC-esm2	0.9715	0.9594	0.9221	0.9407	0.8821
	HLA-A*02:04	CapsNet-MHC-contact	1	0.9677	1	0.9841	0.8687
		CapsNet-MHC-pae	0.999	1	0.9688	0.9841	0.9688
		CapsNet-MHC-esm1	0.998	0.9677	0.9688	0.9683	0.9365
		CapsNet-MHC-esm2	0.998	0.9677	1	0.9841	0.9687
		CapsNet-MHC	0.995	0.9677	1	0.9841	0.9687

HLA-A*02:05	CapsNet-MHC-contact	0.9753	0.96	0.9816	0.9205	0.8438
	CapsNet-MHC	0.9747	0.9733	0.8684	0.9205	0.8459
	CapsNet-MHC-esm2	0.9737	0.9733	0.9816	0.9272	0.8581
	CapsNet-MHC-pae	0.9735	0.96	0.8816	0.9205	0.8438
	CapsNet-MHC-esm1	0.9721	0.9733	0.8421	0.9072	0.822
HLA-A*02:06	CapsNet-MHC-esm2	0.9737	0.9733	0.8816	0.9279	0.8581
	CapsNet-MHC-esm1	0.9681	0.9671	0.8785	0.9227	0.8488
	CapsNet-MHC-contact	0.9648	0.9343	0.8832	0.9087	0.8184
	CapsNet-MHC-pae	0.9645	0.9202	0.8879	0.904	0.8084
	CapsNet-MHC	0.9615	0.9437	0.8645	0.9274	0.8106
HLA-A*02:07	CapsNet-MHC	0.9712	0.9022	0.957	0.9297	0.8607
	CapsNet-MHC-esm2	0.9622	0.9022	0.957	0.9297	0.8607
	CapsNet-MHC-esm1	0.9576	0.8804	0.9247	0.9027	0.8068
	CapsNet-MHC-contact	0.9554	0.9022	0.9355	0.9189	0.8382
	CapsNet-MHC-pae	0.9552	0.8913	0.9355	0.9155	0.8283
HLA-A*02:17	CapsNet-MHC	1	1	0.9355	0.9672	0.9365
	CapsNet-MHC-pae	1	1	0.9032	0.9508	0.9065
	CapsNet-MHC-esm1	1	1	0.9032	0.9508	0.9065
	CapsNet-MHC-esm2	0.9989	1	0.9355	0.9672	0.9365
	CapsNet-MHC-contact	0.9957	1	0.9032	0.9508	0.9065
HLA-A*03:01	CapsNet-MHC-esm2	0.9908	0.9597	0.9442	0.952	0.904
	CapsNet-MHC-contact	0.9784	0.9657	0.9215	0.9436	0.8881
	CapsNet-MHC-esm1	0.9773	0.9597	0.9276	0.9436	0.8877
	CapsNet-MHC-pae	0.9765	0.9516	0.9315	0.9416	0.8828
	CapsNet-MHC	0.9753	0.9597	0.9215	0.9406	0.8818
HLA-A*11:01	CapsNet-MHC	0.9848	0.9694	0.9202	0.9448	0.8906
	CapsNet-MHC-contact	0.9845	0.9711	0.9236	0.9473	0.8957
	CapsNet-MHC-pae	0.9839	0.9694	0.9280	0.9483	0.8972
	CapsNet-MHC-esm1	0.9835	0.9677	0.9185	0.9431	0.8872
	CapsNet-MHC-esm2	0.9829	0.9711	0.9287	0.9499	0.9006
HLA-A*23:01	CapsNet-MHC	0.9868	0.9322	0.9496	0.9409	0.882
	CapsNet-MHC-pae	0.9837	0.9068	0.9328	0.9198	0.8399
	CapsNet-MHC-esm2	0.9832	0.9407	0.9076	0.9241	0.8486
	CapsNet-MHC-esm1	0.9803	0.9068	0.9328	0.9189	0.8399
	CapsNet-MHC-contact	0.9793	0.9138	0.9467	0.9288	0.8568
HLA-A*24:02	CapsNet-MHC	0.9924	0.9651	0.9594	0.9623	0.9245
	CapsNet-MHC-pae	0.9923	0.9522	0.9528	0.9578	0.9159
	CapsNet-MHC-esm1	0.9923	0.9593	0.9536	0.9565	0.9129
	CapsNet-MHC-esm2	0.992	0.9709	0.9391	0.955	0.9105

	CapsNet-MHC-contact	0.9893	0.9538	0.9467	0.9508	0.9014
HLA-A*24:06	CapsNet-MHC	1	1	1	1	1
	CapsNet-MHC-esm2	1	1	1	1	1
	CapsNet-MHC-contact	1	1	0.9525	0.9757	0.9525
	CapsNet-MHC-esm1	1	0.95	1	0.9756	0.9523
	CapsNet-MHC-pae	0.9857	0.9	0.9525	0.9267	0.8545
HLA-A*26:01	CapsNet-MHC	0.986	0.9385	0.9697	0.9542	0.9088
	CapsNet-MHC-esm1	0.9816	0.9538	0.9394	0.9467	0.8939
	CapsNet-MHC-esm2	0.979	0.9538	0.9545	0.9542	0.9082
	CapsNet-MHC-pae	0.9765	0.9381	0.9542	0.9466	0.8932
	CapsNet-MHC-contact	0.9676	0.9385	0.8788	0.9084	0.8184
HLA-A*29:02	CapsNet-MHC	0.9837	0.9592	0.9472	0.9532	0.9064
	CapsNet-MHC-esm2	0.983	0.9592	0.939	0.9491	0.8984
	CapsNet-MHC-pae	0.9820	0.9265	0.9431	0.9348	0.8698
	CapsNet-MHC-esm1	0.9796	0.9551	0.935	0.945	0.8902
	CapsNet-MHC-contact	0.9790	0.9510	0.9310	0.9450	0.8901
HLA-A*30:01	CapsNet-MHC	0.8442	0.7949	0.775	0.7848	0.5699
	CapsNet-MHC-contact	0.7615	0.5641	0.725	0.6426	0.2931
	CapsNet-MHC-esm2	0.759	0.5898	0.795	0.6589	0.3178
	CapsNet-MHC-pae	0.7481	0.5128	0.75	0.6329	0.2707
	CapsNet-MHC-esm1	0.7423	0.6667	0.725	0.6962	0.3924
HLA-A*30:02	CapsNet-MHC	0.9781	1	0.9167	0.958	0.9194
	CapsNet-MHC-contact	0.9767	0.9859	0.9028	0.9441	0.8913
	CapsNet-MHC-pae	0.9737	0.9577	0.8889	0.9273	0.8484
	CapsNet-MHC-esm2	0.9734	0.9577	0.8611	0.9091	0.8222
	CapsNet-MHC-esm1	0.9676	0.9437	0.9027	0.9231	0.8468
HLA-A*31:01	CapsNet-MHC	0.9769	0.9532	0.9025	0.9278	0.8568
	CapsNet-MHC-pae	0.9764	0.9362	0.9154	0.9257	0.8516
	CapsNet-MHC-esm2	0.9726	0.9447	0.9153	0.9299	0.8603
	CapsNet-MHC-contact	0.9713	0.9262	0.9234	0.9257	0.8514
	CapsNet-MHC-esm1	0.9676	0.9447	0.9153	0.9299	0.8603
HLA-A*32:01	CapsNet-MHC-pae	0.9540	0.8783	0.9397	0.9091	0.8196
	CapsNet-MHC	0.9518	0.8783	0.8966	0.8874	0.775
	CapsNet-MHC-contact	0.9484	0.8696	0.9397	0.9048	0.8114
	CapsNet-MHC-esm1	0.9391	0.8609	0.9397	0.9004	0.8032
	CapsNet-MHC-esm2	0.9391	0.8783	0.9052	0.8918	0.7838
HLA-A*33:01	CapsNet-MHC-contact	0.9802	0.9924	0.9015	0.9466	0.8973
	CapsNet-MHC-pae	0.9761	0.9847	0.9091	0.9468	0.8962
	CapsNet-MHC	0.9749	0.9924	0.8939	0.943	0.8903

	CapsNet-MHC-esm2	0.9731	0.9925	0.8939	0.943	0.8903
	CapsNet-MHC-esm1	0.9696	0.9847	0.9015	0.943	0.8891
HLA-A*68:01	CapsNet-MHC-contact	0.9873	0.9722	0.928	0.9501	0.901
	CapsNet-MHC-pae	0.9855	0.975	0.9197	0.9473	0.8960
	CapsNet-MHC-esm1	0.9848	0.975	0.928	0.9515	0.9039
	CapsNet-MHC	0.9838	0.9833	0.9086	0.9459	0.8943
	CapsNet-MHC-esm2	0.9832	0.9611	0.9114	0.9362	0.8735
HLA-A*68:02	CapsNet-MHC	0.9512	0.8944	0.898	0.8962	0.7924
	CapsNet-MHC-contact	0.9493	0.8713	0.9046	0.888	0.7764
	CapsNet-MHC-pae	0.9485	0.8845	0.9211	0.9028	0.8068
	CapsNet-MHC-esm1	0.9474	0.901	0.8947	0.8979	0.7957
	CapsNet-MHC-esm2	0.9457	0.8944	0.9013	0.8979	0.7975
HLA-A*69:01	CapsNet-MHC	0.9954	0.96	0.9231	0.9412	0.8831
	CapsNet-MHC-contact	0.9892	1	0.8849	0.9428	0.8887
	CapsNet-MHC-esm1	0.9877	0.96	0.9231	0.9412	0.8831
	CapsNet-MHC-esm2	0.9769	0.92	0.9231	0.9216	0.8431
	CapsNet-MHC-pae	0.9754	0.84	0.8849	0.8646	0.7257
HLA-B*07:02	CapsNet-MHC	0.9924	0.972	0.9572	0.9646	0.9293
	CapsNet-MHC-pae	0.9916	0.9608	0.9504	0.9548	0.916
	CapsNet-MHC-contact	0.9905	0.9562	0.9634	0.9607	0.9208
	CapsNet-MHC-esm1	0.9904	0.9552	0.9534	0.9543	0.9068
	CapsNet-MHC-esm2	0.9904	0.9608	0.9572	0.959	0.918
HLA-B*08:01	CapsNet-MHC-esm2	0.9571	0.9275	0.9143	0.9209	0.8418
	CapsNet-MHC-pae	0.9559	0.8986	0.8857	0.8921	0.7843
	CapsNet-MHC	0.9559	0.8986	0.8857	0.8921	0.7843
	CapsNet-MHC-contact	0.9495	0.8406	0.9143	0.8777	0.7572
	CapsNet-MHC-esm1	0.9431	0.8551	0.8857	0.8705	0.7412
HLA-B*13:02	CapsNet-MHC	1	0.9524	1	0.9767	0.9544
	CapsNet-MHC-pae	1	0.8048	1	0.9072	0.8272
	CapsNet-MHC-esm2	0.9848	0.9048	0.9091	0.907	0.8139
	CapsNet-MHC-contact	0.9913	0.9576	0.9543	0.959	0.9063
	CapsNet-MHC-esm1	0.9913	0.9524	0.9091	0.9302	0.8615
HLA-B*14:02	CapsNet-MHC	0.991	0.9744	0.925	0.9494	0.8999
	CapsNet-MHC-pae	0.9791	0.9744	0.9	0.9364	0.876
	CapsNet-MHC-contact	0.99	0.9744	0.9	0.9364	0.876
	CapsNet-MHC-esm1	0.9859	0.9744	0.875	0.9241	0.8526
	CapsNet-MHC-esm2	0.975	0.9744	0.85	0.9114	0.8297
HLA-B*15:01	CapsNet-MHC	0.9892	0.9757	0.9462	0.9609	0.9222
	CapsNet-MHC-pae	0.9892	0.9634	0.9543	0.9584	0.9168

	CapsNet-MHC-contact	0.9884	0.9806	0.934	0.9576	0.9159
	CapsNet-MHC-esm2	0.9879	0.9617	0.941	0.9513	0.9029
	CapsNet-MHC-esm1	0.9868	0.9635	0.9444	0.954	0.9081
HLA-B*18:01	CapsNet-MHC	0.9939	0.9762	0.9302	0.9529	0.9069
	CapsNet-MHC-esm1	0.9934	0.9524	0.9302	0.9412	0.8828
	CapsNet-MHC-pae	0.9929	0.9562	0.9582	0.9529	0.9059
	CapsNet-MHC-contact	0.99	0.9562	0.9582	0.9529	0.9059
	CapsNet-MHC-esm2	0.9889	0.9286	0.9535	0.9412	0.8825
HLA-B*27:01	CapsNet-MHC-pae	0.9991	1	0.9688	0.9894	0.9679
	CapsNet-MHC-esm1	0.9991	1	0.9683	0.9841	0.9687
	CapsNet-MHC	0.9988	1	0.9788	0.9894	0.979
	CapsNet-MHC-contact	0.9987	1	0.9777	0.9888	0.9784
	CapsNet-MHC-esm2	0.9987	1	0.9683	0.9841	0.9687
HLA-B*27:02	CapsNet-MHC	0.9978	0.9779	0.989	0.9835	0.967
	CapsNet-MHC-pae	0.995	0.9773	0.9845	0.9808	0.9614
	CapsNet-MHC-esm1	0.9935	0.9724	0.9835	0.978	0.956
	CapsNet-MHC-esm2	0.9933	0.9779	0.9835	0.9807	0.9614
	CapsNet-MHC-contact	0.9925	0.9782	0.9829	0.981	0.9613
HLA-B*27:03	CapsNet-MHC	0.9951	0.9787	0.9792	0.9789	0.9579
	CapsNet-MHC-pae	0.9934	0. 9787	0.9572	0.9673	0.937
	CapsNet-MHC-esm1	0.9925	0.9787	0.9583	0.9684	0.9371
	CapsNet-MHC-esm2	0.9911	0.9787	0.9583	0.9684	0.9371
	CapsNet-MHC-contact	0.9908	0.9787	0.8953	0.9364	0.8767
HLA-B*27:04	CapsNet-MHC	0.9883	0.9333	1	0.9669	0.9359
	CapsNet-MHC-pae	0.9869	0.9333	1	0.9669	0.9359
	CapsNet-MHC-esm2	0.9869	0.9333	1	0.9669	0.9359
	CapsNet-MHC-esm1	0.9861	0.9333	1	0.9699	0.9359
	CapsNet-MHC-contact	0.985	0.9333	0.967	0.9502	0.9014
HLA-B*27:05	CapsNet-MHC-esm2	0.9099	0.7928	0.8756	0.8342	0.6706
	CapsNet-MHC-esm1	0.9086	0.786	0.8812	0.8336	0.6703
	CapsNet-MHC	0.9064	0.7882	0.8665	0.8274	0.6568
	CapsNet-MHC-pae	0.9063	0.786	0.8731	0.8296	0.6615
	CapsNet-MHC-contact	0.9037	0.7397	0.8916	0.8156	0.6385
HLA-B*27:06	CapsNet-MHC-esm2	0.998	0.96	0.9804	0.9703	0.9408
	CapsNet-MHC-esm1	0.9976	0.96	1	0.9802	0.9611
	CapsNet-MHC-pae	0.9973	0.94	0.9804	0.9604	0.9215
	CapsNet-MHC-contact	0.9973	0.96	0.9604	0.9604	0.9208
	CapsNet-MHC	0.9969	0.94	0.9804	0.9604	0.9215
HLA-B*27:07	CapsNet-MHC-esm1	0.9972	0.9904	0.9905	0.9904	0.9809

	CapsNet-MHC-esm2	0.9969	0.9904	0.981	0.9856	0.9713
	CapsNet-MHC	0.9963	0.9904	0.9905	0.9904	0.9809
	CapsNet-MHC-pae	0.995	0.9987	0.9487	0.9688	0.99345
	CapsNet-MHC-contact	0.9915	0.9904	0.9905	0.9904	0.9809
HLA-B*27:08	CapsNet-MHC-esm2	1	1	0.972	0.9859	0.9722
	CapsNet-MHC-esm1	0.9997	1	0.972	0.9859	0.9722
	CapsNet-MHC-pae	0.9996	0.9906	0.972	0.9812	0.9631
	CapsNet-MHC	0.9994	0.9906	0.972	0.9812	0.9631
	CapsNet-MHC-contact	0.9938	0.9793	0.8788	0.9249	0.8538
HLA-B*27:09	CapsNet-MHC	0.9879	0.9273	0.9502	0.9388	0.8778
	CapsNet-MHC-esm2	0.9827	0.9364	0.9548	0.9456	0.8913
	CapsNet-MHC-esm1	0.982	0.9364	0.9457	0.941	0.8821
	CapsNet-MHC-pae	0.98	0.9157	0.9574	0.9314	0.8629
	CapsNet-MHC-contact	0.9706	0.9187	0.8926	0.907	0.8145
HLA-B*35:01	CapsNet-MHC	0.9695	0.9355	0.8964	0.9159	0.8325
	CapsNet-MHC-pae	0.9679	0.9319	0.9	0.9153	0.8321
	CapsNet-MHC-esm2	0.9673	0.9211	0.8893	0.9052	0.8108
	CapsNet-MHC-esm1	0.9665	0.9247	0.9	0.9123	0.825
	CapsNet-MHC-contact	0.9663	0.9175	0.8967	0.9074	0.8148
HLA-B*35:03	CapsNet-MHC-contact	0.9963	0.9593	0.94	0.9542	0.8992
	CapsNet-MHC	0.9927	0.9388	0.94	0.9394	0.8788
	CapsNet-MHC-pae	0.9922	0.9591	0.96	0.9594	0.9195
	CapsNet-MHC-esm1	0.9906	0.9592	0.96	0.9596	0.9192
	CapsNet-MHC-esm2	0.9902	0.9592	0.94	0.9495	0.8992
HLA-B*35:08	CapsNet-MHC	0.9769	0.9565	0.9787	0.9677	0.9357
	CapsNet-MHC-esm1	0.9769	0.9565	0.9787	0.9677	0.9357
	CapsNet-MHC-pae	0.9759	0.9567	0.9578	0.9578	0.9147
	CapsNet-MHC-contact	0.9746	0.9567	0.9578	0.9578	0.9147
	CapsNet-MHC-esm2	0.9727	0.9348	0.9787	0.957	0.9148
HLA-B*37:01	CapsNet-MHC	0.9983	0.9877	0.939	0.9632	0.9275
	CapsNet-MHC-esm2	0.9935	0.963	0.9512	0.9571	0.9142
	CapsNet-MHC-esm1	0.9905	0.9383	0.9634	0.9507	0.9021
	CapsNet-MHC-pae	0.9903	0.9639	0.9518	0.9511	0.9158
	CapsNet-MHC-contact	0.9893	0.9506	0.9519	0.9508	0.9016
HLA-B*39:01	CapsNet-MHC	0.9989	0.9804	0.9615	0.9709	0.9419
	CapsNet-MHC-pae	0.9985	0.9804	0.9805	0.9809	0.9619
	CapsNet-MHC-esm1	0.9981	0.9804	0.9615	0.9709	0.9419
	CapsNet-MHC-esm2	0.9977	0.9804	0.9808	0.9806	0.9612
	CapsNet-MHC-contact	0.9962	0.9804	0.9805	0.9809	0.9619

HLA-B*40:01	CapsNet-MHC	0.9977	0.9823	0.9648	0.9735	0.9472
	CapsNet-MHC-esm2	0.997	0.9779	0.9648	0.9713	0.9427
	CapsNet-MHC-pae	0.9963	0.9692	0.9738	0.9715	0.9451
	CapsNet-MHC-esm1	0.996	0.9779	0.9604	0.9691	0.9383
	CapsNet-MHC-contact	0.9953	0.9698	0.9694	0.9698	0.9386
HLA-B*40:02	CapsNet-MHC	0.995	0.978	0.9615	0.9697	0.9396
	CapsNet-MHC-pae	0.9949	0.978	0.9613	0.9693	0.9396
	CapsNet-MHC-contact	0.9947	0.9757	0.9647	0.9696	0.9398
	CapsNet-MHC-esm2	0.9947	0.9835	0.956	0.9697	0.9398
	CapsNet-MHC-esm1	0.994	0.978	0.956	0.967	0.9342
HLA-B*41:01	CapsNet-MHC	0.989	0.9375	1	0.9697	0.941
	CapsNet-MHC-pae	0.9853	0.8175	1	0.9097	0.831
	CapsNet-MHC-esm2	0.9853	0.9375	1	0.9697	0.941
	CapsNet-MHC-esm1	0.9769	0.9375	1	0.9697	0.941
	CapsNet-MHC-contact	0.9485	0.9375	1	0.9697	0.941
HLA-B*44:02	CapsNet-MHC-pae	0.9903	0.9616	0.9547	0.9571	0.9152
	CapsNet-MHC-esm1	0.9894	0.9563	0.954	0.9552	0.9103
	CapsNet-MHC	0.9892	0.9684	0.9419	0.9552	0.9106
	CapsNet-MHC-esm2	0.9892	0.9636	0.9467	0.9552	0.9104
	CapsNet-MHC-contact	0.9874	0.9445	0.9566	0.9155	0.9007
HLA-B*44:03	CapsNet-MHC-esm2	0.9942	0.9725	0.9699	0.9712	0.9424
	CapsNet-MHC	0.994	0.9835	0.9671	0.9753	0.9507
	CapsNet-MHC-pae	0.994	0.9698	0.9694	0.9697	0.9384
	CapsNet-MHC-contact	0.9934	0.9588	0.9697	0.9645	0.9285
	CapsNet-MHC-esm1	0.9932	0.978	0.9699	0.9739	0.9479
HLA-B*44:27	CapsNet-MHC	1	1	1	1	1
	CapsNet-MHC-pae	1	1	1	1	1
	CapsNet-MHC-esm1	1	1	1	1	1
	CapsNet-MHC-esm2	1	1	1	1	1
	CapsNet-MHC-contact	0.9905	0.9283	0.9333	0.9316	0.8619
HLA-B*45:01	CapsNet-MHC-contact	0.9803	0.9354	0.9814	0.9582	0.9183
	CapsNet-MHC	0.9774	0.9537	0.9174	0.9355	0.8716
	CapsNet-MHC-pae	0.9767	0.9441	0.9632	0.9531	0.9084
	CapsNet-MHC-esm1	0.9763	0.9352	0.945	0.9401	0.8802
	CapsNet-MHC-esm2	0.9752	0.9444	0.9541	0.9493	0.8986
HLA-B*46:01	CapsNet-MHC	0.9498	0.9079	0.8961	0.902	0.804
	CapsNet-MHC-pae	0.9395	0.8813	0.8832	0.8826	0.7647
	CapsNet-MHC-esm1	0.9357	0.8947	0.9091	0.902	0.804
	CapsNet-MHC-esm2	0.9327	0.8684	0.8961	0.8824	0.7649

	CapsNet-MHC-contact	0.9315	0.8683	0.8442	0.8566	0.717
HLA-B*49:01	CapsNet-MHC-esm2	0.9861	0.9439	0.9352	0.9395	0.8791
	CapsNet-MHC	0.9828	0.9346	0.9259	0.9302	0.8605
	CapsNet-MHC-esm1	0.9813	0.9439	0.9354	0.9395	0.8791
	CapsNet-MHC-contact	0.9771	0.9446	0.8709	0.9072	0.8165
	CapsNet-MHC-pae	0.976	0.9253	0.9534	0.9398	0.8762
HLA-B*50:01	CapsNet-MHC	0.9974	0.9767	0.9773	0.977	0.954
	CapsNet-MHC-esm1	0.9699	0.9535	0.9445	0.954	0.908
	CapsNet-MHC-contact	0.985	0.975	0.947	0.96	0.922
	CapsNet-MHC-pae	0.981	0.963	0.958	0.96	0.921
	CapsNet-MHC-esm2	0.9709	0.9302	0.9545	0.9425	0.8852
HLA-B*51:01	CapsNet-MHC-pae	0.9701	0.878	0.9396	0.9088	0.8191
	CapsNet-MHC-contact	0.9683	0.8902	0.9515	0.921	0.8436
	CapsNet-MHC	0.9668	0.9207	0.9333	0.9271	0.8542
	CapsNet-MHC-esm2	0.9656	0.8841	0.9576	0.921	0.8442
	CapsNet-MHC-esm1	0.9628	0.8841	0.9455	0.9149	0.8313
HLA-B*53:01	CapsNet-MHC	0.9794	0.9661	0.9167	0.9412	0.8835
	CapsNet-MHC-pae	0.9791	0.9661	0.9167	0.9412	0.8835
	CapsNet-MHC-esm1	0.9751	0.8983	0.9333	0.916	0.8323
	CapsNet-MHC-esm2	0.9726	0.8983	0.9	0.8992	0.7983
	CapsNet-MHC-contact	0.9661	0.8983	0.9	0.9	0.7983
HLA-B*54:01	CapsNet-MHC	0.9711	0.8485	0.9851	0.9173	0.8422
	CapsNet-MHC-contact	0.9688	0.8485	0.9701	0.9025	0.8176
	CapsNet-MHC-pae	0.9647	0.8333	0.9844	0.9096	0.8284
	CapsNet-MHC-esm2	0.9622	0.8333	1	0.9173	0.8461
	CapsNet-MHC-esm1	0.9516	0.8182	1	0.9098	0.833
HLA-B*56:01	CapsNet-MHC	0.9985	1	0.9189	0.9589	0.921
	CapsNet-MHC-esm2	0.9932	1	0.9189	0.9589	0.921
	CapsNet-MHC-esm1	0.9887	1	0.9189	0.9589	0.921
	CapsNet-MHC-contact	0.9827	1	0.9189	0.9589	0.921
	CapsNet-MHC-pae	0.9767	0.9165	0.9456	0.9313	0.8633
HLA-B*57:01	CapsNet-MHC-pae	0.977	0.9135	0.9752	0.9445	0.8912
	CapsNet-MHC-contact	0.9767	0.9227	0.9568	0.9391	0.8786
	CapsNet-MHC	0.9764	0.9171	0.9659	0.9415	0.8841
	CapsNet-MHC-esm1	0.9735	0.9008	0.9627	0.9318	0.8652
	CapsNet-MHC-esm2	0.9727	0.9041	0.9578	0.931	0.8631
HLA-B*57:03	CapsNet-MHC-contact	0.9822	0.9535	0.9451	0.9498	0.8986
	CapsNet-MHC	0.9818	0.9539	0.9541	0.954	0.908
	CapsNet-MHC-pae	0.9792	0.9535	0.9451	0.9498	0.8986

	CapsNet-MHC-esm1	0.977	0.9493	0.945	0.9471	0.8943
	CapsNet-MHC-esm2	0.9769	0.9447	0.9358	0.9402	0.8805
HLA-B*58:01	CapsNet-MHC-pae	0.9751	0.9094	0.9346	0.9213	0.8441
	CapsNet-MHC	0.9737	0.9293	0.9296	0.9295	0.8589
	CapsNet-MHC-esm1	0.973	0.904	0.9447	0.9244	0.8495
	CapsNet-MHC-contact	0.9729	0.9196	0.9199	0.9193	0.8386
	CapsNet-MHC-esm2	0.9728	0.9141	0.9447	0.9295	0.8591
HLA-C*01:02	CapsNet-MHC	0.9813	0.9512	0.8795	0.9152	0.8326
	CapsNet-MHC-esm1	0.9706	0.9146	0.8916	0.903	0.8063
	CapsNet-MHC-esm2	0.9702	0.939	0.9038	0.9212	0.843
	CapsNet-MHC-pae	0.9686	0.9262	0.8795	0.9032	0.8071
	CapsNet-MHC-contact	0.9584	0.9122	0.8795	0.897	0.7945
HLA-C*02:02	CapsNet-MHC	0.9875	0.9675	0.9113	0.9393	0.88
	CapsNet-MHC-esm2	0.9855	0.9593	0.9032	0.9312	0.8638
	CapsNet-MHC-esm1	0.9788	0.9593	0.8952	0.9271	0.8561
	CapsNet-MHC-pae	0.9783	0.9674	0.8463	0.9069	0.8198
	CapsNet-MHC-contact	0.9771	0.9674	0.8463	0.9069	0.8198
HLA-C*03:03	CapsNet-MHC-pae	0.9566	0.8592	0.9304	0.8591	0.7921
	CapsNet-MHC-contact	0.9554	0.8454	0.9162	0.8811	0.7662
	CapsNet-MHC-esm2	0.9525	0.8592	0.9583	0.9091	0.822
	CapsNet-MHC-esm1	0.946	0.8451	0.9444	0.8951	0.7939
	CapsNet-MHC	0.9435	0.8732	0.9444	0.9091	0.8201
HLA-C*03:04	CapsNet-MHC	0.9872	0.939	0.9036	0.9212	0.843
	CapsNet-MHC-esm1	0.9841	0.9512	0.9398	0.9455	0.891
	CapsNet-MHC-esm2	0.9837	0.939	0.9038	0.9212	0.843
	CapsNet-MHC-pae	0.9808	0.9264	0.9633	0.9451	0.8913
	CapsNet-MHC-contact	0.9784	0.9394	0.9392	0.9393	0.8784
HLA-C*04:01	CapsNet-MHC	0.9695	0.8961	0.9397	0.9179	0.8366
	CapsNet-MHC-esm2	0.9662	0.8788	0.9526	0.9158	0.8338
	CapsNet-MHC-pae	0.963	0.8749	0.9484	0.9119	0.8252
	CapsNet-MHC-contact	0.9624	0.8657	0.9442	0.9051	0.8126
	CapsNet-MHC-esm1	0.9607	0.8788	0.9483	0.9136	0.8292
HLA-C*05:01	CapsNet-MHC-esm2	0.9811	0.8983	0.958	0.9283	0.858
	CapsNet-MHC	0.9784	0.9068	0.9748	0.9409	0.8838
	CapsNet-MHC-pae	0.9773	0.8983	0.9742	0.9369	0.8755
	CapsNet-MHC-contact	0.9768	0.8894	0.9586	0.9241	0.85
	CapsNet-MHC-esm1	0.9732	0.8814	0.958	0.9198	0.842
HLA-C*06:02	CapsNet-MHC	0.9491	0.866	0.9184	0.8923	0.7856
	CapsNet-MHC-contact	0.9424	0.8552	0.8778	0.8664	0.7331

		CapsNet-MHC-pae	0.9411	0.8452	0.9385	0.8928	0.7874
		CapsNet-MHC-esm1	0.9404	0.866	0.9388	0.9026	0.8071
		CapsNet-MHC-esm2	0.9391	0.8557	0.9388	0.8974	0.7975
	HLA-C*07:01	CapsNet-MHC-esm1	0.9909	0.9375	0.9753	0.9656	0.9137
		CapsNet-MHC	0.99	0.975	0.963	0.9689	0.938
		CapsNet-MHC-esm2	0.9887	0.95	0.9753	0.9627	0.9257
		CapsNet-MHC-pae	0.9863	0.9374	0.9634	0.9504	0.9008
		CapsNet-MHC-contact	0.9858	0.925	0.963	0.9447	0.8888
	HLA-C*07:02	CapsNet-MHC-pae	0.9756	0.8902	0.9464	0.9182	0.8395
		CapsNet-MHC	0.9744	0.9455	0.9107	0.9279	0.8564
		CapsNet-MHC-esm2	0.9714	0.9091	0.9286	0.9189	0.8379
		CapsNet-MHC-esm1	0.9682	0.8909	0.9464	0.9189	0.839
		CapsNet-MHC-contact	0.9627	0.8909	0.9104	0.9009	0.8019
	HLA-C*07:04	CapsNet-MHC	0.9735	1	0.8788	0.9385	0.8838
		CapsNet-MHC-pae	0.9659	1	0.8485	0.9238	0.8577
		CapsNet-MHC-esm2	0.965	1	0.8485	0.9231	0.8566
		CapsNet-MHC-esm1	0.9536	0.9688	0.8485	0.9077	0.8219
		CapsNet-MHC-contact	0.9328	0.9365	0.7878	0.8615	0.7323
	HLA-C*08:02	CapsNet-MHC-contact	0.9936	0.9643	0.9646	0.9644	0.9289
		CapsNet-MHC-pae	0.9933	0.9642	0.9552	0.96	0.92
		CapsNet-MHC	0.9927	0.9643	0.9646	0.9644	0.9289
		CapsNet-MHC-esm1	0.9915	0.9554	0.9646	0.96	0.92
		CapsNet-MHC-esm2	0.9905	0.9732	0.9558	0.9644	0.929
	HLA-C*14:02	CapsNet-MHC	0.9925	1	0.9538	0.9767	0.9545
		CapsNet-MHC-pae	0.9923	1	0.9538	0.9767	0.9545
		CapsNet-MHC-esm2	0.9901	1	0.9385	0.969	0.9398
		CapsNet-MHC-contact	0.9887	1	0.9538	0.9767	0.9545
		CapsNet-MHC-esm1	0.9885	1	0.9538	0.9767	0.9545
	HLA-C*16:01	CapsNet-MHC-pae	0.9718	0.9342	0.8932	0.914	0.8288
		CapsNet-MHC-esm1	0.9718	0.8913	0.9149	0.9032	0.8066
		CapsNet-MHC-esm2	0.9695	0.913	0.8936	0.9031	0.8067
		CapsNet-MHC-contact	0.9653	0.913	0.957	0.9355	0.8717
		CapsNet-MHC	0.9598	0.913	0.8298	0.871	0.7449
11	HLA-A*01:01	CapsNet-MHC	0.9928	0.974	0.9397	0.9568	0.9142
		CapsNet-MHC-pae	0.9911	0.9697	0.9397	0.9546	0.9097
		CapsNet-MHC-contact	0.991	0.9567	0.9267	0.9417	0.8838
		CapsNet-MHC-esm1	0.9902	0.9567	0.931	0.9438	0.888
		CapsNet-MHC-esm2	0.9896	0.961	0.9267	0.9438	0.8882
	HLA-A*02:01	CapsNet-MHC-contact	0.9642	0.9243	0.8907	0.9085	0.8154

	CapsNet-MHC-pae	0.9623	0.9044	0.9066	0.9055	0.8109
	CapsNet-MHC-esm1	0.9619	0.9223	0.8926	0.9075	0.8153
	CapsNet-MHC	0.9612	0.9163	0.8807	0.8985	0.7975
	CapsNet-MHC-esm2	0.9575	0.9243	0.8867	0.9055	0.8115
HLA-A*02:03	CapsNet-MHC-esm2	0.9601	0.8462	0.9259	0.8868	0.7754
	CapsNet-MHC-esm1	0.9587	0.8077	0.9259	0.8679	0.74
	CapsNet-MHC-pae	0.9487	0.8462	0.9630	0.9057	0.8161
	CapsNet-MHC-contact	0.9444	0.8077	0.9259	0.8679	0.74
	CapsNet-MHC	0.9387	0.8077	0.9259	0.8679	0.74
HLA-A*02:04	CapsNet-MHC-esm1	0.9816	0.931	0.9667	0.9492	0.8987
	CapsNet-MHC-contact	0.9793	0.9655	0.9667	0.9661	0.9332
	CapsNet-MHC	0.977	0.8966	0.9667	0.9322	0.8662
	CapsNet-MHC-pae	0.9759	0.931	0.9667	0.9492	0.8987
	CapsNet-MHC-esm2	0.9759	0.931	0.9333	0.9322	0.8644
HLA-A*02:05	CapsNet-MHC	0.9837	1	0.9444	0.9714	0.9444
	CapsNet-MHC-pae	0.9804	1	0.9444	0.9714	0.9444
	CapsNet-MHC-contact	0.9804	1	0.9444	0.9714	0.9444
	CapsNet-MHC-esm1	0.9904	1	0.8889	0.9429	0.8918
	CapsNet-MHC-esm2	0.9641	1	0.8889	0.9429	0.8913
HLA-A*02:07	CapsNet-MHC	0.9771	0.9211	0.9481	0.9346	0.8696
	CapsNet-MHC-contact	0.9689	0.9211	0.9351	0.9214	0.854
	CapsNet-MHC-esm2	0.9645	0.9211	0.9221	0.9216	0.8431
	CapsNet-MHC-esm1	0.9614	0.9211	0.9481	0.9346	0.8696
	CapsNet-MHC-pae	0.9586	0.9474	0.9221	0.9346	0.8696
HLA-A*03:01	CapsNet-MHC-contact	0.9813	0.9576	0.9458	0.9517	0.9035
	CapsNet-MHC-esm2	0.9811	0.9515	0.9398	0.9456	0.8913
	CapsNet-MHC-pae	0.9797	0.9567	0.9699	0.9637	0.9276
	CapsNet-MHC	0.9786	0.9636	0.9639	0.9637	0.9275
	CapsNet-MHC-esm1	0.978	0.9349	0.9518	0.9456	0.8913
HLA-A*11:01	CapsNet-MHC	0.9822	0.9614	0.9359	0.9486	0.8975
	CapsNet-MHC-pae	0.981	0.9571	0.9386	0.9479	0.8936
	CapsNet-MHC-esm1	0.9803	0.9657	0.9231	0.9443	0.8896
	CapsNet-MHC-esm2	0.978	0.9657	0.9231	0.9443	0.8895
	CapsNet-MHC-contact	0.9771	0.9553	0.9313	0.9429	0.8845
HLA-A*23:01	CapsNet-MHC	1	1	1	1	1
	CapsNet-MHC-pae	1	1	1	1	1
	CapsNet-MHC-contact	1	1	1	1	1
	CapsNet-MHC-esm1	1	1	1	1	1
	CapsNet-MHC-esm2	1	1	1	1	1

HLA-A*24:02	CapsNet-MHC	0.9949	0.9624	0.984	0.9732	0.9466
	CapsNet-MHC-pae	0.9945	0.9677	0.9733	0.9705	0.941
	CapsNet-MHC-esm2	0.994	0.9677	0.9626	0.9651	0.9303
	CapsNet-MHC-esm1	0.9913	0.9677	0.9626	0.9651	0.9303
	CapsNet-MHC-contact	0.9937	0.9638	0.9662	0.9598	0.9197
HLA-A*24:06	CapsNet-MHC-contact	1	1	1	1	1
	CapsNet-MHC-esm2	0.9868	0.8421	0.95	0.8974	0.7984
	CapsNet-MHC-pae	0.9785	0.7821	1	0.8974	0.8111
	CapsNet-MHC-esm1	0.9763	0.7368	0.95	0.8463	0.7057
	CapsNet-MHC	0.9553	0.7895	0.95	0.8718	0.7514
HLA-A*29:02	CapsNet-MHC	0.9914	0.9468	0.9579	0.9524	0.9048
	CapsNet-MHC-esm1	0.9877	0.9468	0.9579	0.9524	0.9048
	CapsNet-MHC-pae	0.987	0.9255	0.9363	0.9312	0.8641
	CapsNet-MHC-esm2	0.9859	0.9268	0.9684	0.9577	0.9155
	CapsNet-MHC-contact	0.9828	0.9438	0.9262	0.9398	0.8737
HLA-A*31:01	CapsNet-MHC	0.988	0.9848	0.8209	0.9023	0.816
	CapsNet-MHC-pae	0.9837	0.9697	0.8954	0.9394	0.8672
	CapsNet-MHC-contact	0.9842	0.9697	0.8504	0.9094	0.8257
	CapsNet-MHC-esm1	0.9796	0.9697	0.8358	0.9023	0.8122
	CapsNet-MHC-esm2	0.9754	0.9697	0.8657	0.9173	0.8394
HLA-A*32:01	CapsNet-MHC-esm2	0.9894	0.96	0.9412	0.9505	0.9012
	CapsNet-MHC	0.9878	0.94	0.9608	0.9505	0.9011
	CapsNet-MHC-esm1	0.9875	0.98	0.9216	0.9505	0.9026
	CapsNet-MHC-pae	0.9867	0.96	0.9418	0.9505	0.9012
	CapsNet-MHC	0.9855	0.98	0.9412	0.965	0.9215
HLA-A*68:01	CapsNet-MHC	1	1	0.9273	0.9633	0.9292
	CapsNet-MHC-contact	1	1	0.8903	0.945	0.8976
	CapsNet-MHC-pae	0.9973	1	0.9091	0.953	0.9145
	CapsNet-MHC-esm2	0.9923	1	0.8909	0.945	0.8954
	CapsNet-MHC-esm1	0.9896	1	0.9273	0.9633	0.9292
HLA-A*68:02	CapsNet-MHC	0.9294	0.8429	0.9155	0.8794	0.7606
	CapsNet-MHC-esm2	0.9197	0.8	0.9155	0.8582	0.7207
	CapsNet-MHC-contact	0.9167	0.8414	0.9285	0.8852	0.7557
	CapsNet-MHC-pae	0.9133	0.7575	0.8723	0.8227	0.6504
	CapsNet-MHC-esm1	0.9115	0.7857	0.8873	0.8369	0.6769
HLA-B*07:02	CapsNet-MHC-contact	0.9866	0.9596	0.9634	0.9619	0.9231
	CapsNet-MHC	0.9845	0.9522	0.967	0.9596	0.9194
	CapsNet-MHC-pae	0.9841	0.9528	0.9702	0.9619	0.9231
	CapsNet-MHC-esm2	0.9834	0.9522	0.9634	0.9578	0.9156

	CapsNet-MHC-esm1	0.9825	0.9522	0.9524	0.9523	0.9046
HLA-B*08:01	CapsNet-MHC	0.9554	0.9697	0.8529	0.9104	0.8271
	CapsNet-MHC-esm1	0.951	0.9394	0.8529	0.8955	0.7945
	CapsNet-MHC-pae	0.9447	0.9394	0.8824	0.9106	0.8225
	CapsNet-MHC-contact	0.934	0.8182	0.8824	0.8505	0.7024
	CapsNet-MHC-esm2	0.9332	0.9394	0.7941	0.8657	0.7401
HLA-B*15:01	CapsNet-MHC-pae	0.9846	0.9596	0.9554	0.9575	0.915
	CapsNet-MHC	0.9822	0.9552	0.9509	0.953	0.906
	CapsNet-MHC-esm2	0.9815	0.9552	0.933	0.9441	0.8884
	CapsNet-MHC-contact	0.9809	0.9552	0.933	0.9441	0.8884
	CapsNet-MHC-esm1	0.9776	0.9596	0.9375	0.9485	0.8973
HLA-B*27:01	CapsNet-MHC-esm1	0.9993	0.9818	0.991	0.9864	0.9729
	CapsNet-MHC-esm2	0.9989	0.9818	0.973	0.9774	0.9584
	CapsNet-MHC-pae	0.9987	0.9909	0.982	0.9864	0.9732
	CapsNet-MHC	0.9984	0.9818	0.973	0.9774	0.9548
	CapsNet-MHC-contact	0.9962	0.981	1	0.9984	0.9869
HLA-B*27:02	CapsNet-MHC	0.9999	0.9829	1	0.9915	0.9831
	CapsNet-MHC-esm2	0.9999	0.9829	1	0.9915	0.9831
	CapsNet-MHC-esm1	0.9993	0.9744	0.9915	0.9830	0.9661
	CapsNet-MHC-pae	0.9992	0.983	1	0.9916	0.9831
	CapsNet-MHC-contact	0.9972	0.9915	0.9824	0.9875	0.9745
HLA-B*27:03	CapsNet-MHC	1	1	1	1	1
	CapsNet-MHC-esm1	1	1	1	1	1
	CapsNet-MHC-esm2	1	1	1	1	1
	CapsNet-MHC-pae	1	1	0.9734	0.9865	0.9737
	CapsNet-MHC-contact	1	1	0.9653	0.987	0.976
HLA-B*27:04	CapsNet-MHC-esm2	0.9729	0.9615	0.963	0.9623	0.9245
	CapsNet-MHC	0.9701	0.9615	0.963	0.9623	0.9245
	CapsNet-MHC-esm1	0.9687	0.9615	0.963	0.9623	0.9245
	CapsNet-MHC-pae	0.9671	0.9615	0.9263	0.9456	0.8875
	CapsNet-MHC-contact	0.9657	0.962	0.8518	0.9081	0.8162
HLA-B*27:05	CapsNet-MHC-esm1	0.8783	0.7192	0.8735	0.7964	0.5999
	CapsNet-MHC-esm2	0.8772	0.7329	0.8598	0.7964	0.5976
	CapsNet-MHC	0.8769	0.7277	0.8667	0.7973	0.6003
	CapsNet-MHC-pae	0.8749	0.726	0.865	0.7956	0.5968
	CapsNet-MHC-contact	0.8685	0.6742	0.8838	0.7814	0.5761
HLA-B*27:06	CapsNet-MHC-esm1	1	1	0.9613	0.9804	0.9615
	CapsNet-MHC-esm2	1	1	0.9613	0.9804	0.9615
	CapsNet-MHC	1	1	0.9231	0.9608	0.9245

	CapsNet-MHC-pae	1	1	0.8897	0.9425	0.8885
	CapsNet-MHC-contact	0.9985	1	0.9277	0.9688	0.9278
HLA-B*27:07	CapsNet-MHC	1	1	1	1	1
	CapsNet-MHC-esm1	1	1	1	1	1
	CapsNet-MHC-esm2	1	1	0.9878	0.9931	0.9831
	CapsNet-MHC-pae	0.9995	1	0.9654	0.9867	0.9675
	CapsNet-MHC-contact	0.9989	0.9874	0.9677	0.9758	0.9507
HLA-B*27:08	CapsNet-MHC-contact	0.9964	0.9567	0.9468	0.952	0.9062
	CapsNet-MHC-esm2	0.9959	0.9865	0.9867	0.9866	0.9731
	CapsNet-MHC-pae	0.9957	0.9865	0.9846	0.9872	0.9732
	CapsNet-MHC-esm1	0.9951	0.9865	0.9867	0.9866	0.9732
	CapsNet-MHC	0.9946	0.9865	1	0.9933	0.9867
HLA-B*27:09	CapsNet-MHC	0.9682	0.9078	0.9437	0.9258	0.8521
	CapsNet-MHC-contact	0.9619	0.9178	0.9017	0.908	0.8161
	CapsNet-MHC-pae	0.961	0.8872	0.9385	0.9115	0.8243
	CapsNet-MHC-esm2	0.9607	0.922	0.9155	0.9187	0.8375
	CapsNet-MHC-esm1	0.959	0.9	0.9085	0.9046	0.8092
HLA-B*35:01	CapsNet-MHC-pae	0.9775	0.9351	0.8964	0.9151	0.8314
	CapsNet-MHC	0.9747	0.9457	0.9154	0.9305	0.8614
	CapsNet-MHC-esm1	0.9741	0.907	0.9077	0.9073	0.8147
	CapsNet-MHC-esm2	0.9732	0.8915	0.9385	0.9151	0.831
	CapsNet-MHC-contact	0.9709	0.907	0.9154	0.9112	0.8224
HLA-B*35:03	CapsNet-MHC	0.9977	1	0.9333	0.9661	0.9344
	CapsNet-MHC-esm1	0.9977	1	0.9	0.9492	0.9031
	CapsNet-MHC-pae	0.9943	1	0.8654	0.9321	0.8723
	CapsNet-MHC-contact	0.9943	1	0.8654	0.9321	0.8723
	CapsNet-MHC-esm2	0.9931	1	0.9	0.9492	0.9031
HLA-B*35:08	CapsNet-MHC	0.9947	0.963	1	0.9818	0.9642
	CapsNet-MHC-contact	0.9907	0.963	0.9234	0.9456	0.8916
	CapsNet-MHC-esm1	0.9894	0.963	0.9286	0.9455	0.8915
	CapsNet-MHC-esm2	0.9881	0.963	0.9286	0.9455	0.8915
	CapsNet-MHC-pae	0.9762	0.9633	0.9283	0.9431	0.8964
HLA-B*37:01	CapsNet-MHC-esm1	0.9915	0.8571	1	0.931	0.8691
	CapsNet-MHC-contact	0.9905	0.9286	1	0.9655	0.933
	CapsNet-MHC	0.9905	1	0.9333	0.9655	0.9333
	CapsNet-MHC-esm2	0.9905	0.8571	1	0.931	0.8697
	CapsNet-MHC-pae	0.9857	0.8564	1	0.9356	0.8697
HLA-B*39:01	CapsNet-MHC-esm1	1	1	1	1	1
	CapsNet-MHC	1	1	0.9714	0.9855	0.9714

	CapsNet-MHC-contact	1	1	0.9414	0.9752	0.9437
	CapsNet-MHC-pae	0.9992	1	0.9794	0.9837	0.9714
	CapsNet-MHC-esm2	0.9966	1	0.9429	0.971	0.9431
HLA-B*40:01	CapsNet-MHC	1	1	0.982	0.991	0.9821
	CapsNet-MHC-esm1	0.9997	0.9909	0.973	0.9819	0.964
	CapsNet-MHC-esm2	0.9996	1	0.982	0.991	0.9821
	CapsNet-MHC-pae	0.9991	0.9953	0.9729	0.9822	0.9644
	CapsNet-MHC-contact	0.999	0.9909	0.973	0.9818	0.964
HLA-B*40:02	CapsNet-MHC-pae	0.9948	0.9655	0.9589	0.9629	0.9244
	CapsNet-MHC-esm1	0.9936	0.9655	0.9589	0.9622	0.9244
	CapsNet-MHC-esm2	0.9934	0.9655	0.9658	0.9656	0.9313
	CapsNet-MHC-contact	0.9933	0.9867	0.9599	0.9637	0.9376
	CapsNet-MHC	0.99	0.9724	0.9589	0.9556	0.9314
HLA-B*44:02	CapsNet-MHC-esm2	0.9911	0.9412	0.984	0.9627	0.9262
	CapsNet-MHC-esm1	0.9904	0.9305	0.984	0.9573	0.916
	CapsNet-MHC	0.9898	0.9572	0.9628	0.96	0.92
	CapsNet-MHC-pae	0.9895	0.9467	0.9738	0.96	0.9203
	CapsNet-MHC-contact	0.9854	0.9461	0.9753	0.9657	0.9254
HLA-B*44:03	CapsNet-MHC-contact	0.9969	0.9896	0.979	0.9791	0.9594
	CapsNet-MHC	0.9966	0.9864	0.9797	0.9831	0.9661
	CapsNet-MHC-esm1	0.9955	0.9864	0.9865	0.9864	0.9729
	CapsNet-MHC-pae	0.9938	0.9796	0.9797	0.9791	0.9597
	CapsNet-MHC-esm2	0.992	0.9864	0.9865	0.9864	0.9729
HLA-B*45:01	CapsNet-MHC	0.9718	0.9035	0.9688	0.9365	0.8473
	CapsNet-MHC-contact	0.9718	0.9355	0.9375	0.9365	0.873
	CapsNet-MHC-esm1	0.9627	0.9032	0.9688	0.9365	0.8746
	CapsNet-MHC-esm2	0.9617	0.9032	0.9378	0.9206	0.8416
	CapsNet-MHC-pae	0.9597	0.9325	0.9348	0.9367	0.873
HLA-B*46:01	CapsNet-MHC	0.9532	0.8889	0.9474	0.9189	0.8387
	CapsNet-MHC-contact	0.9269	0.6122	1	0.8178	0.6681
	CapsNet-MHC-pae	0.924	0.6645	1	0.8397	0.7126
	CapsNet-MHC-esm2	0.9064	0.6111	0.9474	0.7838	0.5963
	CapsNet-MHC-esm1	0.8977	0.6667	1	0.8378	0.7118
HLA-B*49:01	CapsNet-MHC	1	1	1	1	1
	CapsNet-MHC-pae	1	1	1	1	1
	CapsNet-MHC-contact	1	1	1	1	1
	CapsNet-MHC-esm1	1	1	1	1	1
	CapsNet-MHC-esm2	1	1	1	1	1
HLA-B*51:01	CapsNet-MHC-pae	0.9945	0.9333	0.989	0.9667	0.9243

	CapsNet-MHC-esm2	0.9941	0.9778	0.978	0.9779	0.9558
	CapsNet-MHC-esm1	0.9933	0.9333	0.978	0.9558	0.9125
	CapsNet-MHC	0.9921	0.9667	0.967	0.9669	0.9337
	CapsNet-MHC-contact	0.988	0.9222	0.9529	0.9391	0.8784
HLA-B*54:01	CapsNet-MHC	0.9839	0.8333	1	0.918	0.8471
	CapsNet-MHC-pae	0.9774	0.8	0.9653	0.8865	0.7804
	CapsNet-MHC-esm1	0.971	0.7667	0.9677	0.8689	0.7516
	CapsNet-MHC-contact	0.9613	0.7667	0.9347	0.8525	0.7138
	CapsNet-MHC-esm2	0.9591	0.8333	0.9032	0.8689	0.7389
HLA-B*56:01	CapsNet-MHC	1	1	1	1	1
	CapsNet-MHC-esm1	1	1	1	1	1
	CapsNet-MHC-esm2	1	1	1	1	1
	CapsNet-MHC-pae	1	0.9376	1	0.9697	0.9409
	CapsNet-MHC-contact	1	0.9376	1	0.9697	0.9409
HLA-B*57:01	CapsNet-MHC	0.9808	0.9236	0.948	0.9358	0.8719
	CapsNet-MHC-pae	0.9768	0.9184	0.9684	0.9484	0.8815
	CapsNet-MHC-contact	0.9766	0.9263	0.9464	0.9342	0.8785
	CapsNet-MHC-esm1	0.976	0.9203	0.9532	0.9367	0.8737
	CapsNet-MHC-esm2	0.9745	0.9184	0.9515	0.935	0.8704
HLA-B*57:03	CapsNet-MHC	0.9908	0.982	0.9464	0.9642	0.929
	CapsNet-MHC-contact	0.9899	0.982	0.9264	0.9592	0.9069
	CapsNet-MHC-pae	0.9897	0.9581	0.9452	0.9522	0.9044
	CapsNet-MHC-esm1	0.9876	0.9701	0.9286	0.9493	0.8993
	CapsNet-MHC-esm2	0.987	0.9701	0.9345	0.9522	0.9051
HLA-B*58:01	CapsNet-MHC	0.988	0.9612	0.9808	0.971	0.9422
	CapsNet-MHC-pae	0.9867	0.9709	0.9612	0.9671	0.9342
	CapsNet-MHC-contact	0.9841	0.9709	0.9761	0.9753	0.942
	CapsNet-MHC-esm1	0.9835	0.9515	0.9808	0.9662	0.9327
	CapsNet-MHC-esm2	0.981	0.9612	0.9712	0.9662	0.9324
HLA-C*01:02	CapsNet-MHC	0.9535	0.8636	0.9111	0.8876	0.7759
	CapsNet-MHC-esm2	0.9444	0.8636	0.9333	0.8989	0.7994
	CapsNet-MHC-esm1	0.9414	0.8182	0.9556	0.8876	0.7821
	CapsNet-MHC-contact	0.9348	0.7745	0.8888	0.8315	0.6663
	CapsNet-MHC-pae	0.9293	0.776	0.9355	0.8551	0.7169
HLA-C*02:02	CapsNet-MHC-contact	0.9986	0.8862	1	0.9435	0.8925
	CapsNet-MHC-esm1	0.9957	0.9231	1	0.9623	0.927
	CapsNet-MHC-pae	0.9929	0.9631	0.9653	0.9623	0.9247
	CapsNet-MHC	0.9925	0.9231	1	0.9623	0.927
	CapsNet-MHC-esm2	0.9915	0.9615	1	0.9811	0.9629

HLA-C*03:03	CapsNet-MHC	0.9881	0.9	1	0.9512	0.9065
	CapsNet-MHC-esm2	0.9833	0.95	0.9524	0.9512	0.9024
	CapsNet-MHC-pae	0.9784	0.90	0.955	0.9238	0.8576
	CapsNet-MHC-contact	0.969	0.95	0.9048	0.9224	0.8548
	CapsNet-MHC-esm1	0.969	0.95	0.9048	0.9224	0.8548
HLA-C*03:04	CapsNet-MHC-esm1	0.9969	0.96	0.9615	0.9608	0.9215
	CapsNet-MHC-contact	0.9969	0.92	0.9654	0.9408	0.8829
	CapsNet-MHC-esm2	0.9969	0.92	0.9616	0.9412	0.8829
	CapsNet-MHC	0.9892	0.92	0.9615	0.9412	0.8829
	CapsNet-MHC-pae	0.9877	0.924	0.9656	0.9408	0.8897
HLA-C*04:01	CapsNet-MHC-contact	0.8825	0.6719	0.9077	0.7907	0.5973
	CapsNet-MHC	0.8668	0.6875	0.8769	0.7829	0.5753
	CapsNet-MHC-esm2	0.863	0.7031	0.8923	0.7984	0.6069
	CapsNet-MHC-esm1	0.8611	0.6406	0.8923	0.7674	0.5513
	CapsNet-MHC-pae	0.8576	0.678	0.8726	0.7765	0.5645
HLA-C*05:01	CapsNet-MHC-contact	0.9749	0.9165	0.9336	0.9259	0.854
	CapsNet-MHC-pae	0.9732	0.9167	0.9508	0.9339	0.8683
	CapsNet-MHC-esm1	0.9699	0.9	0.9508	0.9256	0.8522
	CapsNet-MHC	0.9669	0.9167	0.9672	0.9421	0.8853
	CapsNet-MHC-esm2	0.962	0.9333	0.918	0.9256	0.8514
HLA-C*06:02	CapsNet-MHC-contact	0.9097	0.8	0.8633	0.831	0.6627
	CapsNet-MHC-pae	0.9032	0.7718	0.7611	0.8169	0.6356
	CapsNet-MHC-esm1	0.8944	0.8286	0.8611	0.8451	0.6902
	CapsNet-MHC-esm2	0.8857	0.7714	0.8611	0.8169	0.6359
	CapsNet-MHC	0.8778	0.8	0.8333	0.8169	0.6338
HLA-C*07:01	CapsNet-MHC	0.991	0.9268	0.9286	0.9277	0.9554
	CapsNet-MHC-esm1	0.9872	0.878	0.9524	0.9157	0.8333
	CapsNet-MHC-esm2	0.9821	0.9512	0.9048	0.9277	0.8565
	CapsNet-MHC-pae	0.982	0.9024	0.9278	0.9183	0.8315
	CapsNet-MHC-contact	0.9779	0.9512	0.9524	0.9521	0.9042
HLA-C*07:02	CapsNet-MHC	0.9548	0.75	0.9048	0.8293	0.6643
	CapsNet-MHC-contact	0.9548	0.75	0.9048	0.8043	0.6193
	CapsNet-MHC-esm1	0.9333	0.75	0.9048	0.8293	0.6643
	CapsNet-MHC-esm2	0.9333	0.9	0.9048	0.8537	0.7099
	CapsNet-MHC-pae	0.9286	0.75	0.9048	0.8293	0.6643
HLA-C*08:02	CapsNet-MHC-pae	0.9946	0.9667	0.9387	0.9508	0.9022
	CapsNet-MHC-esm1	0.9935	0.9333	0.9032	0.918	0.8366
	CapsNet-MHC	0.9903	0.9333	0.9355	0.9344	0.8688
	CapsNet-MHC-contact	0.9882	0.9667	0.9387	0.9508	0.9022

		CapsNet-MHC-esm2	0.9849	0.9667	0.9355	0.9508	0.9022
	HLA-C*16:01	CapsNet-MHC-pae	1	1	0.9333	0.9655	0.9333
		CapsNet-MHC	0.9905	1	0.9333	0.9655	0.9333
		CapsNet-MHC-contact	0.9810	0.9265	0.8665	0.8966	0.7952
		CapsNet-MHC-esm2	0.9762	1	0.9333	0.9655	0.9333
		CapsNet-MHC-esm1	0.9714	1	0.9333	0.9655	0.9333
12	HLA-A*01:01	CapsNet-MHC	0.9865	0.9364	0.9483	0.9424	0.8848
		CapsNet-MHC-contact	0.9858	0.9364	0.9425	0.9395	0.878
		CapsNet-MHC-pae	0.9853	0.9364	0.954	0.9452	0.8906
		CapsNet-MHC-esm2	0.9852	0.9364	0.9368	0.9366	0.8732
		CapsNet-MHC-esm1	0.9836	0.9364	0.9483	0.9424	0.8848
	HLA-A*02:01	CapsNet-MHC-contact	0.954	0.916	0.8714	0.895	0.7907
		CapsNet-MHC-pae	0.9480	0.8947	0.8865	0.8906	0.7812
		CapsNet-MHC-esm1	0.9466	0.9035	0.8734	0.8884	0.7772
		CapsNet-MHC-esm2	0.9462	0.9079	0.8777	0.8928	0.7859
		CapsNet-MHC	0.9444	0.886	0.8821	0.884	0.7681
	HLA-A*03:01	CapsNet-MHC-pae	0.9778	0.8852	0.8941	0.8947	0.7829
		CapsNet-MHC	0.9794	0.9425	0.9432	0.9429	0.8852
		CapsNet-MHC-esm1	0.9771	0.9425	0.9318	0.9371	0.8743
		CapsNet-MHC-esm2	0.9752	0.931	0.9205	0.9257	0.8515
		CapsNet-MHC-contact	0.9616	0.9425	0.9091	0.9257	0.852
	HLA-A*11:01	CapsNet-MHC-contact	0.9844	0.9516	0.9883	0.9652	0.9344
		CapsNet-MHC	0.9757	0.9355	0.9683	0.952	0.9044
		CapsNet-MHC-pae	0.9749	0.9525	0.9841	0.9677	0.9365
		CapsNet-MHC-esm1	0.9729	0.9355	0.9841	0.96	0.921
		CapsNet-MHC-esm2	0.969	0.9516	0.9841	0.968	0.9365
	HLA-A*24:02	CapsNet-MHC-contact	0.9854	0.9032	0.938	0.92	0.8407
		CapsNet-MHC-esm2	0.9808	0.9194	0.9683	0.944	0.889
		CapsNet-MHC-pae	0.9803	0.9194	0.9583	0.9344	0.8789
		CapsNet-MHC	0.9793	0.8871	0.9683	0.928	0.8587
		CapsNet-MHC-esm1	0.9782	0.9032	0.9683	0.936	0.8737
	HLA-A*29:02	CapsNet-MHC-contact	0.9599	0.932	0.9138	0.9253	0.851
		CapsNet-MHC-pae	0.9554	0.9394	0.9404	0.9405	0.8806
		CapsNet-MHC	0.9554	0.9091	0.8824	0.8955	0.7914
		CapsNet-MHC-esm1	0.9528	0.9091	0.9706	0.9403	0.882
		CapsNet-MHC-esm2	0.9492	0.9091	0.9706	0.9403	0.882
	HLA-A*31:01	CapsNet-MHC-contact	0.96	0.88	0.9245	0.902	0.8063
		CapsNet-MHC-esm1	0.9492	0.92	0.9231	0.9216	0.8431
		CapsNet-MHC-pae	0.9462	0.88	0.9245	0.902	0.8063

	CapsNet-MHC	0.94	0.88	0.8462	0.8627	0.7262
	CapsNet-MHC-esm2	0.9354	0.84	0.8462	0.8431	0.6862
HLA-A*68:01	CapsNet-MHC	0.9669	0.9375	0.8824	0.9091	0.8199
	CapsNet-MHC-pae	0.9559	0.9374	0.8834	0.9091	0.8192
	CapsNet-MHC-esm1	0.9485	0.9375	0.8235	0.8788	0.7638
	CapsNet-MHC-contact	0.9412	0.9375	0.8834	0.9091	0.8192
	CapsNet-MHC-esm2	0.9412	0.9375	0.8834	0.9091	0.8192
HLA-A*68:02	CapsNet-MHC	0.9386	0.8158	0.8974	0.8571	0.7161
	CapsNet-MHC-esm1	0.9265	0.8684	0.8717	0.8701	0.7402
	CapsNet-MHC-contact	0.9231	0.8141	0.8249	0.8171	0.6368
	CapsNet-MHC-esm2	0.9157	0.8158	0.8205	0.8182	0.6363
	CapsNet-MHC-pae	0.9062	0.8424	0.8467	0.8435	0.6883
HLA-B*07:02	CapsNet-MHC-contact	0.988	0.9358	0.9636	0.9489	0.8999
	CapsNet-MHC-pae	0.9874	0.945	0.9636	0. 9543	0.9088
	CapsNet-MHC	0.9871	0.945	0.9636	0.9543	0.9088
	CapsNet-MHC-esm2	0.986	0.9541	0.9636	0.9589	0.9178
	CapsNet-MHC-esm1	0.9835	0.9358	0.9636	0.9498	0.8999
HLA-B*08:01	CapsNet-MHC-contact	0.995	0.973	0.9737	0.9733	0.9467
	CapsNet-MHC-pae	0.9893	0.9459	0.9737	0. 96	0.9203
	CapsNet-MHC-esm2	0.9936	0.973	0.9474	0.96	0.9203
	CapsNet-MHC	0.9858	1	0.9211	0.96	0.923
	CapsNet-MHC-esm1	0.9851	0.9459	0.9211	0.9333	0.867
HLA-B*15:01	CapsNet-MHC-pae	0.9764	0.8441	0.9872	0.9161	0.8406
	CapsNet-MHC-contact	0.9747	0.8701	0.9615	0.9161	0.8356
	CapsNet-MHC	0.9744	0.8701	0.9615	0.9161	0.8356
	CapsNet-MHC-esm1	0.9684	0.8701	0.9744	0.9226	0.8496
	CapsNet-MHC-esm2	0.9549	0.8571	0.9487	0.9032	0.8096
HLA-B*27:01	CapsNet-MHC	1	1	1	1	1
	CapsNet-MHC-pae	1	1	1	1	1
	CapsNet-MHC-contact	1	1	1	1	1
	CapsNet-MHC-esm1	1	1	1	1	1
	CapsNet-MHC-esm2	1	1	1	1	1
HLA-B*27:02	CapsNet-MHC-contact	0.9969	0.9369	0.9675	0.9472	0.9896
	CapsNet-MHC	0.9956	0.9492	0.9833	0.9664	0.9333
	CapsNet-MHC-pae	0.9946	0.9368	0.9878	0.9573	0.9176
	CapsNet-MHC-esm1	0.9932	0.9153	0.9833	0.9496	0.9011
	CapsNet-MHC-esm2	0.9927	0.9492	0.95	0.9496	0.8992
HLA-B*27:03	CapsNet-MHC	1	1	1	1	1
	CapsNet-MHC-pae	1	1	1	1	1

	CapsNet-MHC-esm1	1	1	1	1	1
	CapsNet-MHC-esm2	1	1	1	1	1
	CapsNet-MHC-contact	1	1	0.8754	0.9355	0.8787
HLA-B*27:05	CapsNet-MHC-esm2	0.8559	0.742	0.7944	0.7682	0.5372
	CapsNet-MHC-pae	0.8537	0.7634	0.777	0.7702	0.5405
	CapsNet-MHC-esm1	0.8528	0.7567	0.7997	0.7782	0.5569
	CapsNet-MHC-contact	0.8482	0.7166	0.8104	0.7635	0.5293
	CapsNet-MHC	0.8423	0.734	0.7931	0.7635	0.5279
HLA-B*27:07	CapsNet-MHC	1	1	1	1	1
	CapsNet-MHC-esm1	1	1	1	1	1
	CapsNet-MHC-esm2	0.9967	0.9583	0.96	0.9592	0.9183
	CapsNet-MHC-pae	0.9917	0.9583	0.92	0.9383	0.8787
	CapsNet-MHC-contact	0.99	0.9583	0.92	0.9383	0.8787
HLA-B*27:08	CapsNet-MHC	0.9877	0.9062	0.9394	0.9231	0.8464
	CapsNet-MHC-esm2	0.9848	0.9375	0.9394	0.9385	0.7869
	CapsNet-MHC-esm1	0.9848	0.9062	0.9394	0.9231	0.8464
	CapsNet-MHC-pae	0.983	0.9062	0.9394	0.9231	0.8464
	CapsNet-MHC-contact	0.9683	0.9063	0.8169	0.865	0.7232
HLA-B*27:09	CapsNet-MHC-esm1	0.9471	0.8033	0.9677	0.8862	0.7825
	CapsNet-MHC-contact	0.941	0.8197	0.9677	0.8943	0.797
	CapsNet-MHC	0.9371	0.8197	0.9677	0.8943	0.797
	CapsNet-MHC-esm2	0.9276	0.8033	0.9516	0.878	0.764
	CapsNet-MHC-pae	0.9197	0.8393	0.9679	0.9086	0.8117
HLA-B*35:01	CapsNet-MHC-pae	0.9137	0.8889	0.8649	0.8767	0.7557
	CapsNet-MHC-esm2	0.9032	0.8611	0.8108	0.8356	0.6724
	CapsNet-MHC	0.9002	0.8889	0.8378	0.863	0.7273
	CapsNet-MHC-contact	0.8986	0.8889	0.8918	0.8903	0.7803
	CapsNet-MHC-esm1	0.8934	0.8611	0.8648	0.863	0.726
HLA-B*40:01	CapsNet-MHC	1	1	1	1	1
	CapsNet-MHC-esm1	1	1	1	1	1
	CapsNet-MHC-esm2	1	1	1	1	1
	CapsNet-MHC-pae	1	1	0.9654	0.9855	0.9687
	CapsNet-MHC-contact	1	1	0.9654	0.9355	0.9687
HLA-B*40:02	CapsNet-MHC-pae	0.9983	0.9512	0.9762	0.9639	0.9279
	CapsNet-MHC-contact	0.9948	0.9512	0.9662	0.9639	0.9279
	CapsNet-MHC-esm2	0.9936	0.9756	0.9524	0.9639	0.928
	CapsNet-MHC-esm1	0.9895	0.9512	0.9762	0.9639	0.9279
	CapsNet-MHC	0.9787	0.9756	0.9524	0.9639	0.928
HLA-B*44:02	CapsNet-MHC-esm1	0.9804	0.8923	0.9848	0.9389	0.8815

	CapsNet-MHC-pae	0.9783	0.9077	0.9697	0.9389	0.8794
	CapsNet-MHC-contact	0.9779	0.9077	0.9848	0.9425	0.8934
	CapsNet-MHC	0.9725	0.9077	0.9545	0.9313	0.8634
	CapsNet-MHC-esm2	0.9695	0.8923	0.9545	0.9237	0.8488
HLA-B*44:03	CapsNet-MHC-pae	0.99	0.9535	1	0.977	0.955
	CapsNet-MHC	0.9894	0.9535	1	0.977	0.955
	CapsNet-MHC-esm1	0.9889	0.9535	0.9773	0.9655	0.9313
	CapsNet-MHC-esm2	0.9868	0.9535	1	0.977	0.955
	CapsNet-MHC-contact	0.9804	0.9535	1	0.977	0.955
HLA-B*51:01	CapsNet-MHC	0.9317	0.831	0.875	0.8531	0.7068
	CapsNet-MHC-esm1	0.9286	0.8592	0.8472	0.8531	0.7064
	CapsNet-MHC-pae	0.9227	0.831	0.8889	0.8601	0.7213
	CapsNet-MHC-contact	0.9157	0.9014	0.8194	0.8601	0.723
	CapsNet-MHC-esm2	0.9128	0.8873	0.8333	0.8601	0.7215
HLA-B*57:01	CapsNet-MHC	0.9571	0.8927	0.9172	0.905	0.8102
	CapsNet-MHC-pae	0.9559	0.8785	0.9276	0.9033	0.8075
	CapsNet-MHC-contact	0.9558	0.8858	0.9172	0.9019	0.8035
	CapsNet-MHC-esm2	0.9508	0.8858	0.9207	0.9033	0.807
	CapsNet-MHC-esm1	0.9497	0.8858	0.9241	0.925	0.8106
HLA-B*57:03	CapsNet-MHC-esm2	0.982	0.9167	1	0.9589	0.9208
	CapsNet-MHC-pae	0.9782	0.9167	1	0.9589	0.9208
	CapsNet-MHC-contact	0.9761	0.8889	0.973	0.9315	0.8651
	CapsNet-MHC-esm1	0.9741	0.9167	1	0.9589	0.9208
	CapsNet-MHC	0.961	0.8611	1	0.9315	0.871
HLA-B*58:01	CapsNet-MHC	0.9825	0.9143	0.9444	0.9296	0.8594
	CapsNet-MHC-esm2	0.9778	0.9714	0.9444	0.9577	0.9159
	CapsNet-MHC-pae	0.9778	0.9429	0.9444	0.9437	0.8873
	CapsNet-MHC-contact	0.9778	0.9429	0.9444	0.9437	0.8873
	CapsNet-MHC-esm1	0.9746	0.9714	0.9444	0.9577	0.9159
HLA-C*01:02	CapsNet-MHC-esm2	0.9623	0.9512	0.7619	0.8554	0.725
	CapsNet-MHC	0.9495	0.9268	0.881	0.9036	0.8083
	CapsNet-MHC-esm1	0.9448	0.8537	0.8571	0.8554	0.7108
	CapsNet-MHC-pae	0.9447	0.8212	0.8595	0.8435	0.6875
	CapsNet-MHC-contact	0.9365	0.9092	0.9024	0.9068	0.8078
HLA-C*04:01	CapsNet-MHC	0.8	0.56	0.8846	0.7255	0.4715
	CapsNet-MHC-contact	0.7756	0.64	0.7688	0.7059	0.413
	CapsNet-MHC-pae	0.7706	0.608	0.7514	0.7061	0.4031
	CapsNet-MHC-esm2	0.7615	0.64	0.6923	0.6667	0.3328
	CapsNet-MHC-esm1	0.76	0.6	0.8077	0.7059	0.4174

	HLA-C*05:01	CapsNet-MHC-esm2	0.9429	0.85	0.9048	0.878	0.7566
		CapsNet-MHC-esm1	0.9333	0.8	0.9048	0.8537	0.7099
		CapsNet-MHC-contact	0.9333	0.75	0.8095	0.8049	0.6095
		CapsNet-MHC	0.9262	0.8	0.8095	0.8049	0.6095
		CapsNet-MHC-pae	0.9143	0.75	0.8094	0.7847	0.5698
	HLA-C*06:02	CapsNet-MHC	0.7578	0.6154	0.6296	0.6228	0.245
		CapsNet-MHC-contact	0.735	0.5	0.8115	0.6604	0.3223
		CapsNet-MHC-pae	0.7178	0.4224	0.8196	0.6228	0.2591
		CapsNet-MHC-esm2	0.7051	0.4615	0.7778	0.6226	0.2526
		CapsNet-MHC-esm1	0.6681	0.4231	0.7407	0.5849	0.1729
	HLA-C*07:01	CapsNet-MHC	0.9925	0.9444	0.9459	0.9452	0.8904
		CapsNet-MHC-esm2	0.982	0.9444	0.8649	0.9041	0.8111
		CapsNet-MHC-pae	0.979	0.889	0.973	0.9315	0.8658
		CapsNet-MHC-contact	0.9692	0.889	0.8649	0.8767	0.7538
		CapsNet-MHC-esm1	0.9655	0.9167	0.8919	0.9041	0.8086
13	HLA-A*01:01	CapsNet-MHC	0.9987	0.9818	0.982	0.9819	0.9638
		CapsNet-MHC-pae	0.998	0.9816	0.9828	0.9817	0.9634
		CapsNet-MHC-esm2	0.998	0.9727	0.9731	0.9729	0.9457
		CapsNet-MHC-esm1	0.9975	0.9727	0.973	0.9729	0.9457
		CapsNet-MHC-contact	0.997	0.9816	0.9828	0.9817	0.9634
	HLA-A*02:01	CapsNet-MHC-esm2	0.9595	0.8632	0.9271	0.8953	0.7921
		CapsNet-MHC-pae	0.9589	0.8832	0.9688	0.9264	0.856
		CapsNet-MHC-contact	0.9669	0.9011	0.9246	0.9128	0.8369
		CapsNet-MHC-esm1	0.9636	0.8947	0.9583	0.9267	0.855
		CapsNet-MHC	0.9547	0.8632	0.9583	0.9119	0.8256
	HLA-A*03:01	CapsNet-MHC-pae	0.9883	1	0.8943	0.9459	0.8974
		CapsNet-MHC-esm2	0.9795	0.9444	0.7895	0.8649	0.7404
		CapsNet-MHC	0.9708	0.9444	0.7895	0.8649	0.7404
		CapsNet-MHC-esm1	0.9708	0.9444	0.7368	0.8378	0.6936
		CapsNet-MHC-contact	0.9649	1	0.8451	0.9189	0.8496
	HLA-A*11:01	CapsNet-MHC-esm2	0.9766	0.7222	0.9474	0.8378	0.6901
		CapsNet-MHC	0.9708	0.8889	0.8947	0.8919	0.7836
		CapsNet-MHC-contact	0.9708	0.8333	1	0.9123	0.8432
		CapsNet-MHC-esm1	0.9591	0.8333	0.9474	0.8919	0.7876
		CapsNet-MHC-pae	0.9474	0.7222	0.9492	0.8384	0.6905
	HLA-A*24:02	CapsNet-MHC-esm1	0.9885	0.9487	0.925	0.9367	0.8736
		CapsNet-MHC-esm2	0.9885	0.9231	0.925	0.9241	0.8481
		CapsNet-MHC	0.9878	0.8718	0.925	0.8987	0.7983
		CapsNet-MHC-pae	0.9859	0.8974	0.925	0.9134	0.823

	CapsNet-MHC-contact	0.975	0.9231	0.95	0.9367	0.8736
HLA-A*29:02	CapsNet-MHC	0.9804	0.9211	0.8974	0.9091	0.8185
	CapsNet-MHC-pae	0.975	0.9474	0.8974	0.9221	0.8485
	CapsNet-MHC-contact	0.9642	0.9211	0.9231	0.9221	0.8441
	CapsNet-MHC-esm1	0.9602	0.9211	0.8974	0.9091	0.8186
	CapsNet-MHC-esm2	0.9582	0.9211	0.8974	0.9091	0.8186
HLA-A*31:01	CapsNet-MHC-esm1	0.8952	0.7857	0.8	0.7931	0.5857
	CapsNet-MHC-esm2	0.8952	0.7857	0.9333	0.8621	0.7295
	CapsNet-MHC	0.881	0.7143	0.7333	0.7241	0.4476
	CapsNet-MHC-pae	0.8762	0.7143	0.8667	0.7931	0.5894
	CapsNet-MHC-contact	0.8667	0.7143	0.8667	0.7931	0.5894
HLA-A*68:02	CapsNet-MHC	0.8315	0.6957	0.9167	0.8085	0.6297
	CapsNet-MHC-pae	0.7971	0.6957	0.8333	0.766	0.5348
	CapsNet-MHC-contact	0.7772	0.6522	0.875	0.766	0.5421
	CapsNet-MHC-esm1	0.7754	0.6522	0.7919	0.7234	0.4487
	CapsNet-MHC-esm2	0.7736	0.6957	0.857	0.7872	0.5813
HLA-B*07:02	CapsNet-MHC-pae	0.993	0.9062	0.9692	0.938	0.8776
	CapsNet-MHC	0.9899	0.9219	0.9538	0.938	0.8764
	CapsNet-MHC-esm1	0.9885	0.8906	0.9846	0.938	0.8797
	CapsNet-MHC-contact	0.9867	0.9219	0.9538	0.938	0.8764
	CapsNet-MHC-esm2	0.9858	0.9062	0.9692	0.938	0.8776
HLA-B*15:01	CapsNet-MHC-esm2	0.9498	0.8824	0.9231	0.9029	0.8063
	CapsNet-MHC-esm1	0.9487	0.8824	0.8462	0.8641	0.7288
	CapsNet-MHC	0.9446	0.8431	0.9038	0.8738	0.7487
	CapsNet-MHC-contact	0.9408	0.902	0.8264	0.8641	0.7305
	CapsNet-MHC-pae	0.9363	0.8431	0.9038	0.8738	0.7487
HLA-B*27:01	CapsNet-MHC-contact	0.9854	0.9444	0.9474	0.9459	0.8918
	CapsNet-MHC-pae	0.9825	0.9444	0.9474	0.9459	0.8918
	CapsNet-MHC	0.9825	0.9444	0.9474	0.9459	0.8918
	CapsNet-MHC-esm2	0.9708	0.9444	0.9474	0.9459	0.8918
	CapsNet-MHC-esm1	0.9591	0.9444	0.9474	0.9459	0.8918
HLA-B*27:02	CapsNet-MHC	1	1	1	1	1
	CapsNet-MHC-pae	1	1	1	1	1
	CapsNet-MHC-contact	1	1	1	1	1
	CapsNet-MHC-esm2	1	1	1	1	1
	CapsNet-MHC-esm1	1	0.963	1	0.9818	0.9642
HLA-B*27:05	CapsNet-MHC-esm2	0.8497	0.7648	0.7968	0.7808	0.562
	CapsNet-MHC-esm1	0.8437	0.7312	0.783	0.7572	0.515
	CapsNet-MHC-pae	0.8378	0.7727	0.7456	0.759	0.5185

	CapsNet-MHC-contact	0.8375	0.7174	0.7909	0.7542	0.5097
	CapsNet-MHC	0.8315	0.7549	0.7396	0.7473	0.4946
HLA-B*27:08	CapsNet-MHC	1	1	1	1	1
	CapsNet-MHC-pae	1	1	1	1	1
	CapsNet-MHC-esm1	1	1	1	1	1
	CapsNet-MHC-esm2	1	1	1	1	1
	CapsNet-MHC-contact	1	1	0.8793	0.9397	0.8794
HLA-B*27:09	CapsNet-MHC	0.9114	0.75	0.9189	0.8356	0.6797
	CapsNet-MHC-esm1	0.9017	0.8056	0.8649	0.8356	0.6719
	CapsNet-MHC-contact	0.8971	0.7775	0.8649	0.8256	0.6457
	CapsNet-MHC-esm2	0.8791	0.75	0.8919	0.8219	0.6492
	CapsNet-MHC-pae	0.8686	0.6944	0.8374	0.7671	0.5384
HLA-B*35:01	CapsNet-MHC	0.9412	0.9412	0.7222	0.8286	0.6768
	CapsNet-MHC-pae	0.9346	0.8827	0.7772	0.8286	0.6628
	CapsNet-MHC-esm2	0.9248	0.9412	0.7222	0.8286	0.6768
	CapsNet-MHC-contact	0.9183	0.8827	0.7772	0.885	0.7266
	CapsNet-MHC-esm1	0.915	0.8235	0.7778	0.8	0.6013
HLA-B*44:02	CapsNet-MHC-esm1	0.987	0.9044	1	0.9535	0.9107
	CapsNet-MHC-esm2	0.9632	0.9047	1	0.9535	0.9107
	CapsNet-MHC-pae	0.9610	0.9045	1	0.9518	0.9146
	CapsNet-MHC-contact	0.9567	0.9048	1	0.9547	0.9107
	CapsNet-MHC	0.9502	0.9048	1	0.9535	0.9107
HLA-B*51:01	CapsNet-MHC	0.9571	0.8	0.9524	0.878	0.7634
	CapsNet-MHC-esm2	0.9548	0.85	1	0.9268	0.8624
	CapsNet-MHC-esm1	0.9524	0.75	0.9524	0.8537	0.7197
	CapsNet-MHC-pae	0.9405	0.8	0.8571	0.8293	0.6541
	CapsNet-MHC-contact	0.9119	0.7	0.9048	0.805	0.6197
HLA-B*57:01	CapsNet-MHC	0.9538	0.8827	0.9018	0.8923	0.7847
	CapsNet-MHC-pae	0.9478	0.7654	0.9571	0.8615	0.7339
	CapsNet-MHC-contact	0.9458	0.856	0.8948	0.8731	0.7482
	CapsNet-MHC-esm1	0.9422	0.8395	0.9202	0.88	0.7624
	CapsNet-MHC-esm2	0.9395	0.8333	0.9141	0.8738	0.75
HLA-B*57:03	CapsNet-MHC-pae	0.9714	0.95	0.7619	0.8551	0.7224
	CapsNet-MHC-esm1	0.969	0.95	0.8095	0.878	0.765
	CapsNet-MHC-esm2	0.9667	0.9	0.7619	0.8293	0.6667
	CapsNet-MHC-contact	0.9643	0.9	0.8054	0.8537	0.7117
	CapsNet-MHC	0.9452	0.9	0.7619	0.8293	0.6667
HLA-B*58:01	CapsNet-MHC	0.9181	0.5556	0.9474	0.7568	0.55
	CapsNet-MHC-esm1	0.9181	0.7222	0.9474	0.8387	0.6901

		CapsNet-MHC-esm2	0.9181	0.7778	0.8947	0.8378	0.6785
		CapsNet-MHC-pae	0.9123	0.7222	0.8954	0.8108	0.6281
		CapsNet-MHC-contact	0.883	0.6665	0.8961	0.8803	0.5714
	HLA-C*04:01	CapsNet-MHC-esm1	0.6937	0.5	0.6522	0.5778	0.154
		CapsNet-MHC-contact	0.6621	0.6364	0.6974	0.6658	0.3327
		CapsNet-MHC-esm2	0.6581	0.6364	0.5652	0.6	0.202
		CapsNet-MHC-pae	0.6542	0.6342	0.5217	0.5778	0.159
		CapsNet-MHC	0.583	0.5455	0.5217	0.5333	0.0672
	HLA-C*05:01	CapsNet-MHC-contact	0.9125	0.7353	0.875	0.871	0.7417
		CapsNet-MHC	0.9125	0.8667	0.875	0.871	0.7417
		CapsNet-MHC-esm1	0.9041	0.8	0.875	0.8387	0.6778
		CapsNet-MHC-pae	0.8857	0.7373	0.8187	0.7783	0.5466
		CapsNet-MHC-esm2	0.8792	0.8667	0.8125	0.8387	0.6792
	HLA-C*06:02	CapsNet-MHC-pae	0.7935	0.7	0.7741	0.7385	0.4757
		CapsNet-MHC	0.786	0.8333	0.5806	0.7049	0.427
		CapsNet-MHC-esm1	0.7849	0.7333	0.7419	0.7377	0.4753
		CapsNet-MHC-esm2	0.7828	0.6	0.6774	0.6885	0.3774
		CapsNet-MHC-contact	0.7624	0.5624	0.8027	0.6863	0.3834
14	HLA-A*01:01	CapsNet-MHC-contact	0.9951	0.9608	0.9423	0.9515	0.9031
		CapsNet-MHC-esm2	0.9947	0.9804	0.9231	0.9515	0.9045
		CapsNet-MHC-esm1	0.9932	0.9608	0.9231	0.9417	0.8842
		CapsNet-MHC-pae	0.9898	0.9608	0.9615	0.9612	0.9223
		CapsNet-MHC	0.986	0.9608	0.9615	0.9612	0.9223
	HLA-A*02:01	CapsNet-MHC-pae	0.9442	0.8824	0.8654	0.8738	0.7477
		CapsNet-MHC	0.937	0.8824	0.8654	0.8737	0.7477
		CapsNet-MHC-esm2	0.9302	0.902	0.8462	0.8738	0.749
		CapsNet-MHC-contact	0.9246	0.9020	0.825	0.8606	0.7325
		CapsNet-MHC-esm1	0.917	0.8431	0.8462	0.8446	0.6893
	HLA-A*24:02	CapsNet-MHC	1	0.875	1	0.9394	0.8848
		CapsNet-MHC-pae	0.9926	0.875	0.9412	0.9091	0.8192
		CapsNet-MHC-esm2	0.9743	0.75	0.9412	0.8485	0.7069
		CapsNet-MHC-contact	0.9743	0.875	0.8824	0.8788	0.7574
		CapsNet-MHC-esm1	0.9632	0.8125	0.9412	0.8788	0.7621
	HLA-A*68:02	CapsNet-MHC-esm2	0.924	0.8889	0.7367	0.8108	0.6313
		CapsNet-MHC-pae	0.9123	0.8889	0.8947	0.8919	0.7838
		CapsNet-MHC-esm1	0.8655	0.8333	0.8421	0.8378	0.6754
		CapsNet-MHC-contact	0.8509	0.8333	0.7368	0.7838	0.5718
		CapsNet-MHC	0.8392	0.7778	0.7895	0.7838	0.5673
	HLA-B*07:02	CapsNet-MHC-esm2	0.9952	0.9143	1	0.9577	0.9178

	CapsNet-MHC-contact	0.9921	0.9406	0.9746	0.9538	0.9157
	CapsNet-MHC-pae	0.9913	0.9154	0.9706	0.9468	0.8886
	CapsNet-MHC	0.9841	0.8857	0.9722	0.9296	0.8621
	CapsNet-MHC-esm1	0.9802	0.9144	1	0.9577	0.9187
HLA-B*15:01	CapsNet-MHC-contact	0.9498	0.9688	0.8182	0.8965	0.795
	CapsNet-MHC-esm1	0.9498	0.9375	0.8182	0.8769	0.76
	CapsNet-MHC-pae	0.9479	0.8725	0.9054	0.8923	0.7846
	CapsNet-MHC	0.9413	0.9062	0.8485	0.8769	0.7555
	CapsNet-MHC-esm2	0.9384	0.9062	0.8182	0.8615	0.7265
HLA-B*27:05	CapsNet-MHC-esm2	0.8533	0.7579	0.8031	0.7806	0.5616
	CapsNet-MHC-contact	0.8503	0.7895	0.7795	0.7823	0.5647
	CapsNet-MHC-esm1	0.839	0.7658	0.7428	0.7543	0.5087
	CapsNet-MHC-pae	0.833	0.8	0.6798	0.7377	0.4805
	CapsNet-MHC	0.8231	0.8105	0.685	0.7477	0.4995
HLA-B*27:09	CapsNet-MHC-esm2	0.9062	0.76	0.8432	0.8039	0.609
	CapsNet-MHC	0.8954	0.72	0.9615	0.8431	0.7074
	CapsNet-MHC-contact	0.8899	0.8	0.8415	0.8231	0.6474
	CapsNet-MHC-esm1	0.8754	0.64	0.9231	0.7843	0.589
	CapsNet-MHC-pae	0.8462	0.8	0.7311	0.7686	0.5391
HLA-B*35:01	CapsNet-MHC	1	1	1	1	1
	CapsNet-MHC-pae	0.9883	0.9333	0.8138	0.871	0.749
	CapsNet-MHC-esm1	0.9625	0.8667	0.9374	0.9032	0.8075
	CapsNet-MHC-contact	0.9583	0.9333	0.875	0.9082	0.8083
	CapsNet-MHC-esm2	0.9542	0.9333	0.8125	0.871	0.749
HLA-B*57:01	CapsNet-MHC-pae	0.9549	0.6854	0.8667	0.7765	0.5671
	CapsNet-MHC	0.8422	0.7753	0.7111	0.743	0.4873
	CapsNet-MHC-contact	0.8286	0.6745	0.8444	0.7536	0.5269
	CapsNet-MHC-esm2	0.8285	0.6629	0.8333	0.7486	0.5039
	CapsNet-MHC-esm1	0.8262	0.6517	0.8444	0.7486	0.5059
HLA-C*04:01	CapsNet-MHC-pae	0.8447	0.8905	0.7	0.7947	0.6044
	CapsNet-MHC-contact	0.8342	0.7897	0.7	0.7456	0.49
	CapsNet-MHC	0.8105	0.8421	0.75	0.7949	0.5937
	CapsNet-MHC-esm1	0.7895	0.7895	0.65	0.7179	0.443
	CapsNet-MHC-esm2	0.7816	0.7895	0.7	0.7436	0.4908
HLA-C*05:01	CapsNet-MHC-contact	0.8309	0.5625	0.8824	0.7216	0.4716
	CapsNet-MHC-esm2	0.7868	0.4375	0.8824	0.6667	0.3589
	CapsNet-MHC-esm1	0.7574	0.4375	0.8235	0.6364	0.2839
	CapsNet-MHC-pae	0.7537	0.375	0.8865	0.6364	0.3
	CapsNet-MHC	0.7096	0.5	0.8824	0.697	0.4158

HLA-C*06:02	CapsNet-MHC-esm2	0.753	0.6389	0.7297	0.6849	0.3703
	CapsNet-MHC-esm1	0.7185	0.6111	0.6757	0.6438	0.2874
	CapsNet-MHC-contact	0.705	0.5825	0.7027	0.6425	0.2882
	CapsNet-MHC-pae	0.7027	0.75	0.6245	0.6854	0.3744
	CapsNet-MHC	0.6547	0.7222	0.4324	0.5753	0.1615

Supplementary Note 6. The summarized information for benchmark datasets

We utilized two primary datasets, the IEDB and Anthem datasets, for training and evaluating our method, as stated in the main manuscript. To remove duplicate data from the IEDB datasets, we adopted the approach proposed by DeepAttentionPan5, which resulted in 157247 and 8855 records for the training and testing data, respectively.

As mentioned in HLAB17, the Anthem datasets were obtained from three sources, and the positive binding data were curated from the IEDB, EPIMHC27, MHCBN28, and SYFPEITHI29 databases. The training and testing data include 539019 and 172580 binding data, respectively, as detailed in Supplementary Table S8, which provides comprehensive information on the IEDB and Anthem datasets, including the number of data samples for each peptide length. It is worth noting that the method's validation was performed using five-fold cross-validation, as explained in the main manuscript.

Supplementary Table S8. More information for IEDB and Anthem datasets

Peptide Length	IEDB Datasets		Anthem Datasets	
	Training	Test	Training	Test
8-mer	2,792	97	22,643	7,564
9-mer	122,089	8,569	360,248	116,363
10-mer	28,731	154	87,462	27,126
11-mer	2,565	35	39,423	11,659
12-mer	203	0	16,198	5,471
13-mer	376	0	8,373	2,818
14-mer	219	0	4,672	1,579
15-mer	272	0	0	0

Supplementary Note 7. Network parameter settings

In this section, we provide the summarized parameters information of our model. We considered three hyperparameters for our model including batch size, dropout, and learning rate. For optimizing the hyperparameters, we conducted a hyperparameter search using the widely-used Ray tune framework. Various parameters and the corresponding ranges for CapsNet-MHC are illustrated in Supplementary Table S9. In this table, the best combinations of hyperparameters are shown in bold.

Supplementary Table S9. Parameter settings for CapsNet-MHC

Parameter	Range
Total number of parameters	372992
Number of epochs	100
Batch size	256, 512, **1024**
Dropout FCs30	0.1, 0.2, **0.5**
Optimizer	Adam
Learning rate (lr)	0.01, **0.001**, 0.0001
Number of filters (peptide)	15, 32, 64
Number of filter (HLA)	34, 64, 128, 256
Filter length (peptide)	[1,3,3]
Filter length (HLA)	[3,4,3,3]
Hidden neurons (FCs)	220, 200, 1

Supplementary Figure S1. Full distribution of AUC values of HLAB and CapsNet-MHC, for various sizes of peptide k-mer (8≤k≤14)

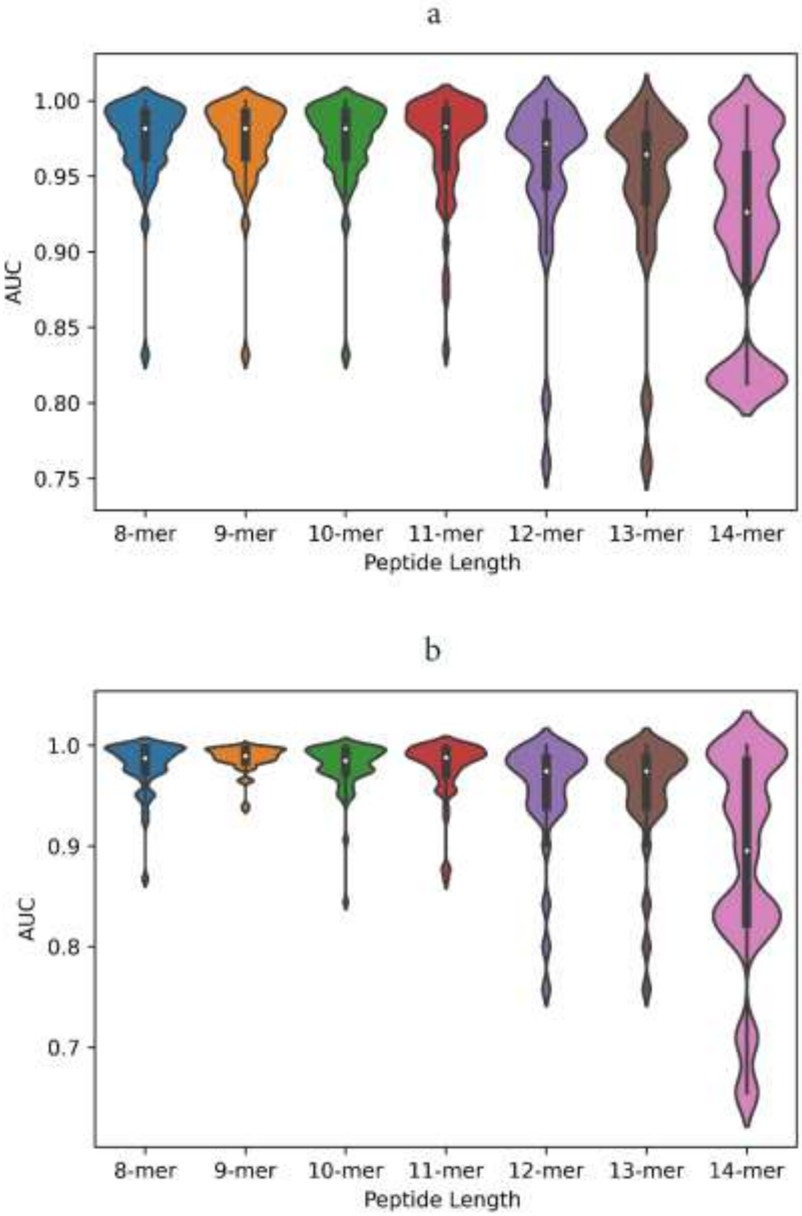

Supplementary Figure S1. Full distribution of AUC values of (a) HLAB and (b) CapsNet-MHC, for various sizes of peptide k-mer (8≤k≤14)

Supplementary Figure S2. Full distribution of ACC, MCC, Specificity, and Sensitivity values of CapsNet-MHC, for various sizes of peptide k-mer (8≤k≤14)

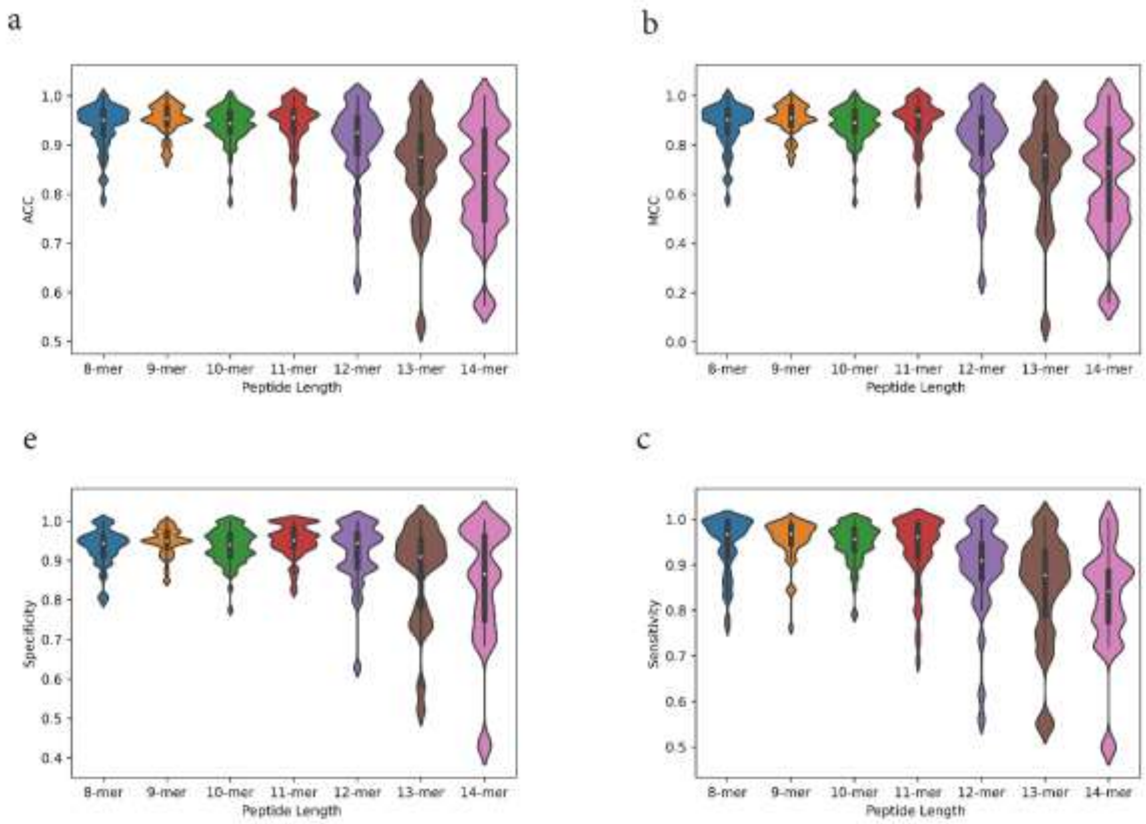

Supplementary Figure S2. Full distribution of (a) ACC, (b) MCC, (c) Specificity, and (d) Sensitivity values of CapsNet-MHC, for various sizes of peptide k-mer (8≤k≤14)

Supplementary Figure S3. Comparing prediction performance of CapsNet-MHC and TranspHLA

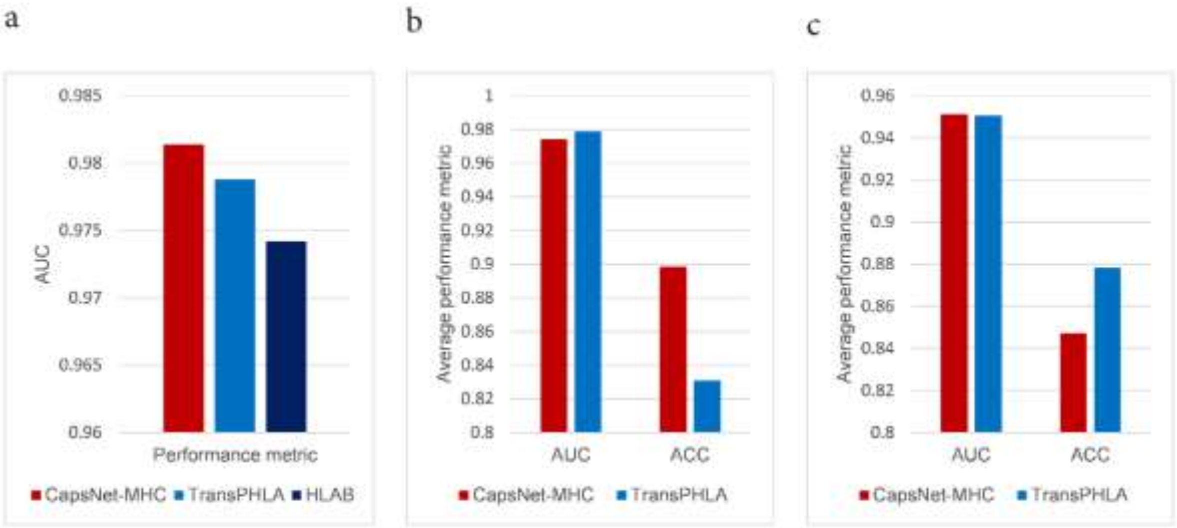

Supplementary Figure S3. Comparing prediction performance of CapsNet-MHC and TranspHLA. (a) in terms of AUC, (b) in terms of AUC and ACC for the independent datasets, and (c) in terms of AUC and ACC for the external datasets

Supplementary Figure S4. Contribution of Capsules for learning the binding features

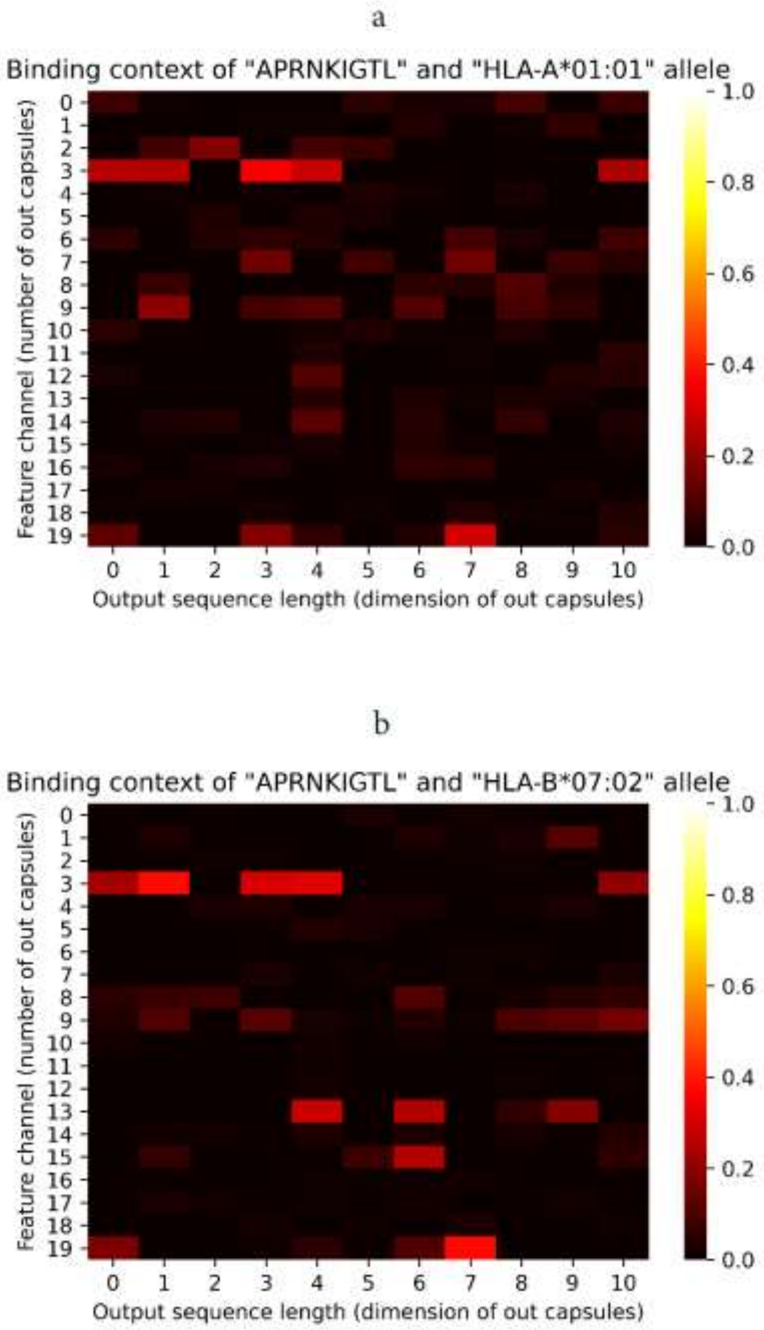

Supplementary Figure S4. Contribution of Capsules for learning the binding features. (a) Heatmap for the peptide APRNKIGTL and the HLA-A*01:01 allele. (b) Heatmap for the peptide APRNKIGTL and the HLA-A*07:02 allele

Supplementary References

1. Nielsen, M. & Andreatta, M. NetMHCpan-3.0: improved prediction of binding to MHC class I molecules integrating information from multiple receptor and peptide length datasets. *Genome Med.* **8**, 33 (2016).
2. Wu, J. et al. DeepHLApan: a deep learning approach for neoantigen prediction considering both HLA-peptide binding and immunogenicity. *Front. Immunol.* **10**, 2559 (2019).
3. Phloyphisut, P., Pornputtapong, N., Sriswasdi, S. & Chuangsuwanich, E. MHCSeqNet: a deep neural network model for universal MHC binding prediction. *BMC Bioinformatics* **20**, 270 (2019).
4. Mikolov, T., Sutskever, I., Chen, K., Corrado, G. & Dean, J. Distributed representations of words and phrases and their compositionality. Preprint at https://arxiv.org/abs/1310.4546 (2013).
5. Hu, Y. et al. ACME: pan-specific peptide-MHC class I binding prediction through attention-based deep neural networks. *Bioinformatics* **35**, 4946–4954 (2019).
6. Jin, J. et al. Deep learning pan-specific model for interpretable MHC-I peptide binding prediction with improved attention mechanism. *Proteins* **89**, 866–883 (2021).
7. Liu, Z. et al. DeepSeqPan, a novel deep convolutional neural network model for pan-specific class I HLA-peptide binding affinity prediction. *Sci. Rep.* **9**, 794 (2019).
8. Zhao, T., Cheng, L., Zang, T. & Hu, Y. Peptide-major histocompatibility complex class I binding prediction based on deep learning with novel feature. *Front. Genet.* **10**, 1191 (2019).
9. Pei, B. & Hsu, Y. H. IConMHC: a deep learning convolutional neural network model to predict peptide and MHC-I binding affinity. *Immunogenetics.* **72**, 295-304 (2020).
10. Xie, X., Han, Y. & Zhang, K. MHCherryPan. a novel model to predict the binding affinity of pan-specific class I HLA-peptide. In *2019 IEEE International Conference on Bioinformatics and Biomedicine (BIBM).* 548-554 (2019).
11. Saxena, S., Animesh, S., Fullwood, M. J. & Mu, Y. OnionMHC: A deep learning model for peptide—HLA-A* 02: 01 binding predictions using both structure and sequence feature sets. *J. micromechanics mol. phys.* **5**, 2050009 (2020).
12. Heng, Y. et al. A Pan-specific GRU-based recurrent neural network for predicting HLA-I-binding peptides. *ACS omega.* **5**, 18321-18330 (2020).
13. Mei, S. et al. Anthem: a user customised tool for fast and accurate prediction of binding between peptides and HLA class I molecules. *Brief. Bioinform.* **22**, bbaa415 (2021).
14. Webb, G. I., Boughton, J. R. & Wang, Z. Not so naive Bayes: aggregating one-dependence estimators. *Mach. Learn.* **58**, 5-24 (2005).
15. Crooks, G. E., Hon, G., Chandonia, J. M. & Brenner, S. E. WebLogo: a sequence logo generator. *Genome Res.* **14**, 1188-1190 (2004).
16. Chu, Y. et al. A transformer-based model to predict peptide–HLA class I binding and optimize mutated peptides for vaccine design. *Nat. Mach. Intell.* **4**, 300-311 (2022).
17. Zhang, Y. et al. HLAB: learning the BiLSTM features from the ProtBert-encoded proteins for the class I HLA-peptide binding prediction. *Brief. Bioinform.* **23**, bbac173 (2022).
18. Jurtz, V. et al. NetMHCpan-4.0: improved peptide-MHC class I interaction predictions integrating eluted ligand and peptide binding affinity data. *J. Immunol.* **199**, 3360–3368 (2017).
19. Zhang, H., Lund, O. & Nielsen, M. The PickPocket method for predicting binding specificities for receptors based on receptor pocket similarities: application to MHC-peptide binding. *Bioinformatics* **25**, 1293–1299 (2009).
20. Peters, B. & Sette, A. Generating quantitative models describing the sequence specificity of biological processes with the stabilized matrix method. *BMC bioinformatics* **6**, 1-9 (2005).
21. Andreatta, M. & Nielsen, M. Gapped sequence alignment using artificial neural networks: application to the MHC class I system. *Bioinformatics* **32**, 511–517 (2016).
22. Trolle, T. et al. Automated benchmarking of peptide-MHC class I binding predictions. *Bioinformatics* **31**, 2174-2181 (2015).

23. Karosiene, E., Lundegaard, C., Lund, O. & Nielsen, M. NetMHCcons: a consensus method for the major histocompatibility complex class I predictions. *Immunogenetics.* **64**, 177-186 (2012).
24. Reynisson, B., Alvarez, B., Paul, S., Peters, B. & Nielsen, M. NetMHCpan-4.1 and NetMHCIIpan-4.0: improved predictions of MHC antigen presentation by concurrent motif deconvolution and integration of MS MHC eluted ligand data. *Nucleic Acids Res.* **48** (W1), W449–W454 (2020).
25. Rasmussen, M. et al. Pan-specific prediction of peptide-MHC class I complex stability, a correlate of T cell immunogenicity. *J. Immunol.* **197**, 1517–1524 (2016).
26. Bassani-Sternberg, M. et al. Deciphering HLA-I motifs across HLA peptidomes improves neo-antigen predictions and identifies allostery regulating HLA specificity. *PLoS Comput. Biol.* **13**, e1005725 (2017).
27. Reche, P. A., Zhang, H., Glutting, J.-P. & Reinherz, E. L. EPIMHC: a curated database of MHC-binding peptides for customized computational vaccinology. *Bioinformatics* **21**, 2140–2141 (2005).
28. Bhasin, M., Singh, H. & Raghava, G. P. S. MHCBN: a comprehensive database of MHC binding and non-binding peptides. *Bioinformatics* **19**, 665-666 (2003).
29. Rammensee, H.-G., Bachmann, J., Emmerich, N. P. N., Bachor, O. A. & Stevanović, S. SYFPEITHI: database for MHC ligands and peptide motifs. *Immunogenetics* **50**, 213–219 (1999).
30. Srivastava, N., Hinton, G., Krizhevsky, A., Sutskever, I. & Salakhutdinov, R. Dropout: a simple way to prevent neural networks from overfitting. *J. Mach. Learn. Res*. **15**, 1929–1958 (2014).
