# Supplementary material for: CapsNet-MHC predicts peptide-MHC class I binding based on capsule neural networks
Source: Commun Biol. 2023 May 5;6:492. doi: 10.1038/s42003-023-04867-2 (PMC10162658; doi:10.1038/s42003-023-04867-2)
Supplement: Supplementary file 2 — Description of Additional Supplementary Files [file 42003_2023_4867_MOESM2_ESM.pdf]

Description of Additional Supplementary Files

File name: Supplementary Data 1

Description: The source data behind the Fig 1 in the paper

File name: Supplementary Data 2

Description: The source data behind the Fig 2 in the paper

File name: Supplementary Data 3

Description: The source data behind the Fig 3 in the paper

File name: Supplementary Data 4

Description: The source data behind the Fig 7 in the paper

File name: Supplementary Data 5

Description: The source data behind the Fig 8 in the paper

File name: Supplementary Data 6

Description: The source data behind the Fig 9 in the paper
