# Supplementary material for: CapsNet-MHC predicts peptide-MHC class I binding based on capsule neural networks
Source: Commun Biol. 2023 May 5;6:492. doi: 10.1038/s42003-023-04867-2 (PMC10162658; doi:10.1038/s42003-023-04867-2)
Supplement: Supplementary file 9 — Reporting Summary [file 42003_2023_4867_MOESM9_ESM.pdf]

Corresponding author(s): DBPR and COMMSBIO-22-3642-T

Last updated by author(s): 2023-04-09

Reporting Summary

Nature Portfolio wishes to improve the reproducibility of the work that we publish. This form provides structure for consistency and transparency in reporting. For further information on Nature Portfolio policies, see our Editorial Policies and the Editorial Policy Checklist.

Statistics

For all statistical analyses, confirm that the following items are present in the figure legend, table legend, main text, or Methods section.

n/a	Confirmed	
☐	☒	The exact sample size (n) for each experimental group/condition, given as a discrete number and unit of measurement
☐	☒	A statement on whether measurements were taken from distinct samples or whether the same sample was measured repeatedly
☐	☒	The statistical test(s) used AND whether they are one- or two-sided<br>*Only common tests should be described solely by name; describe more complex techniques in the Methods section.*
☐	☒	A description of all covariates tested
☒	☐	A description of any assumptions or corrections, such as tests of normality and adjustment for multiple comparisons
☐	☒	A full description of the statistical parameters including central tendency (e.g. means) or other basic estimates (e.g. regression coefficient) AND variation (e.g. standard deviation) or associated estimates of uncertainty (e.g. confidence intervals)
☒	☐	For null hypothesis testing, the test statistic (e.g. F, t, r) with confidence intervals, effect sizes, degrees of freedom and P value noted<br>*Give P values as exact values whenever suitable.*
☒	☐	For Bayesian analysis, information on the choice of priors and Markov chain Monte Carlo settings
☒	☐	For hierarchical and complex designs, identification of the appropriate level for tests and full reporting of outcomes
☒	☐	Estimates of effect sizes (e.g. Cohen's d, Pearson's r), indicating how they were calculated

Our web collection on statistics for biologists contains articles on many of the points above.

Software and code

Policy information about availability of computer code

Data collection	Data processing frameworks and libraries include python, pandas, and numpy.<br>The datasets are available at https://github.com/s7776d/CapsNet-MHC/tree/main/dataset.<br>The code is available at https://github.com/s7776d/CapsNet-MHC.
Data analysis	Data analysis frameworks and libraries include python, pandas, numpy, matplotlib, scipy, seaborn, math, and scikit-learn.<br>The method was implemented using the popular python library PyTorch. The code is available at https://github.com/s7776d/CapsNet-MHC. It should be noted, for our implementation, the DeepAttentionPan source code (https://github.com/jjin49/DeepAttentionPan) were adopted with some required modification and customization.

For manuscripts utilizing custom algorithms or software that are central to the research but not yet described in published literature, software must be made available to editors and reviewers. We strongly encourage code deposition in a community repository (e.g. GitHub). See the Nature Portfolio guidelines for submitting code & software for further information.

Data

Policy information about availability of data

All manuscripts must include a data availability statement. This statement should provide the following information, where applicable:

- Accession codes, unique identifiers, or web links for publicly available datasets
- A description of any restrictions on data availability
- For clinical datasets or third party data, please ensure that the statement adheres to our policy

Several public datasets were used in this research study including the Immune Epitope Database and Analysis Resource (IEDB) [1], and Anthem's datasets [2]. For evaluating our method and comparing it with the alternative methods, using IEDB's dataset, the training datasets called BD2013 and the test datasets called IEDB's weekly benchmark dataset are downloaded from http://tools.iedb.org/main/datasets/ and http://tools.iedb.org/auto_bench/mhci/weekly/, respectively. For a more comprehensive evaluation of our method, Anthem's datasets are downloaded from https://github.com/17shutao/Anthem/tree/master/Dataset.
The datasets are available at https://github.com/s7776d/CapsNet-MHC/tree/main/dataset.
[1] https://www.iedb.org/
[2] Mei S, Li F, Xiang D, Ayala R, Faridi P, Webb GI, Illing PT, Rossjohn J, Akutsu T, Croft NP, Purcell AW. Anthem: a user customised tool for fast and accurate prediction of binding between peptides and HLA class I molecules. Briefings in Bioinformatics. 2021 Sep;22(5):bbaa41

Human research participants

Policy information about studies involving human research participants and Sex and Gender in Research.

Reporting on sex and gender	*Use the terms sex (biological attribute) and gender (shaped by social and cultural circumstances) carefully in order to avoid confusing both terms. Indicate if findings apply to only one sex or gender; describe whether sex and gender were considered in study design whether sex and/or gender was determined based on self-reporting or assigned and methods used. Provide in the source data disaggregated sex and gender data where this information has been collected, and consent has been obtained for sharing of individual-level data; provide overall numbers in this Reporting Summary. Please state if this information has not been collected. Report sex- and gender-based analyses where performed, justify reasons for lack of sex- and gender-based analysis.*
Population characteristics	*Describe the covariate-relevant population characteristics of the human research participants (e.g. age, genotypic information, past and current diagnosis and treatment categories). If you filled out the behavioural & social sciences study design questions and have nothing to add here, write "See above."*
Recruitment	*Describe how participants were recruited. Outline any potential self-selection bias or other biases that may be present and how these are likely to impact results.*
Ethics oversight	*Identify the organization(s) that approved the study protocol.*

Note that full information on the approval of the study protocol must also be provided in the manuscript.

Field-specific reporting

Please select the one below that is the best fit for your research. If you are not sure, read the appropriate sections before making your selection.

☒ Life sciences ☐ Behavioural & social sciences ☐ Ecological, evolutionary & environmental sciences

For a reference copy of the document with all sections, see nature.com/documents/nr-reporting-summary-flat.pdf

Life sciences study design

All studies must disclose on these points even when the disclosure is negative.

Sample size	All the sequence and binding data were collected from IEDB and Anthem datasets. The ratio for IEDB and Anthem datasets are close to that of DeepAttentionPan [1], and HLAB [2], respectively. In this manner, for IEDB datasets, the training and testing sets include 157247 and 8855 records, respectively. For Anthem's dataets, the training and testing datasets include 539019 and 172580 binding data.<br>[1]Jin J, Liu Z, Nasiri A, Cui Y, Louis SY, Zhang A, Zhao Y, Hu J. Deep learning pan-specific model for interpretable MHC-I peptide binding prediction with improved attention mechanism. Proteins: Structure, Function, and Bioinformatics. 2021 Jul;89(7):866-83<br>[2]Zhang Y, Zhu G, Li K, Li F, Huang L, Duan M, Zhou F. HLAB: learning the BiLSTM features from the ProtBert-encoded proteins for the class I HLA-peptide binding prediction. Briefings in Bioinformatics. 2022 May 5.
Data exclusions	No data were excluded from the analysis. For considering the datasets, the duplicated data are removed.
Replication	All the prediction and analysis experiments can be reproduced by cloning our GitHub repository at https://github.com/s7776d/CapsNet-MHC
Randomization	No randomization was needed. We used the widely-used benchmark datasets including IEDB benchmark datasets and the Anthem's datasets.
Blinding	For a fair performance evaluation of the method, the benchmark datasets were blinded to the predictor during the model training steps.

Reporting for specific materials, systems and methods

We require information from authors about some types of materials, experimental systems and methods used in many studies. Here, indicate whether each material, system or method listed is relevant to your study. If you are not sure if a list item applies to your research, read the appropriate section before selecting a response.

Materials & experimental systems

n/a	Involved in the study
☒	☐ Antibodies
☒	☐ Eukaryotic cell lines
☒	☐ Palaeontology and archaeology
☒	☐ Animals and other organisms
☒	☐ Clinical data
☒	☐ Dual use research of concern

Methods

n/a	Involved in the study
☒	☐ ChIP-seq
☒	☐ Flow cytometry
☒	☐ MRI-based neuroimaging
